# RNA-Seq SSRs of Moth Orchid and Screening for Molecular Markers across Genus *Phalaenopsis* (Orchidaceae)

**DOI:** 10.1371/journal.pone.0141761

**Published:** 2015-11-02

**Authors:** Chi-Chu Tsai, Huei-Chuan Shih, Hao-Ven Wang, Yu-Shium Lin, Chia-Hung Chang, Yu-Chung Chiang, Chang-Hung Chou

**Affiliations:** 1 Kaohsiung District Agricultural Research and Extension Station, Pingtung 900, Taiwan; 2 National Pingtung University of Science and Technology, Pingtung 912, Taiwan; 3 Department of Nursing, Meiho University, Pingtung 912, Taiwan; 4 Department of Life Sciences, National Cheng Kung University, Tainan 701, Taiwan; 5 Department of Biological Sciences, National Sun Yat-sen University, Kaohsiung 804, Taiwan; 6 Department of Biomedical Science and Environment Biology, Kaohsiung Medical University, Kaohsiung 807, Taiwan; 7 Research Center for Biodiversity, China Medical University, Taichung 404, Taiwan; Beijing Forestry University, CHINA

## Abstract

**Background:**

The moth orchid (*Phalaenopsis* species) is an ornamental crop that is highly commercialized worldwide. Over 30,000 cultivars of moth orchids have been registered at the Royal Horticultural Society (RHS). These cultivars were obtained by artificial pollination of interspecific hybridization. Therefore, the identification of different cultivars is highly important in the worldwide market.

**Methods/Results:**

We used Illumina sequencing technology to analyze an important species for breeding, *Phalaenopsis aphrodite* subsp. *formosana* and develop the expressed sequence tag (EST)-simple sequence repeat (SSR) markers. After *de novo* assembly, the obtained sequence covered 29.1 Mb, approximately 2.2% of the *P*. *aphrodite* subsp. *formosana* genome (1,300 Mb), and a total of 1,439 EST-SSR loci were detected. SSR occurs in the exon region, including the 5’ untranslated region (UTR), coding region (CDS), and 3’UTR, on average every 20.22 kb. The di- and tri-nucleotide motifs (51.49% and 35.23%, respectively) were the two most frequent motifs in the *P*. *aphrodite* subsp. *formosana*. To validate the developed EST-SSR loci and to evaluate the transferability to the genus *Phalaenopsis*, thirty tri-nucleotide motifs of the EST-SSR loci were randomly selected to design EST-SSR primers and to evaluate the polymorphism and transferability across 22 native *Phalaenopsis* species that are usually used as parents for moth orchid breeding. Of the 30 EST-SSR loci, ten polymorphic and transferable SSR loci across the 22 native taxa can be obtained. The validated EST-SSR markers were further proven to discriminate 12 closely related *Phalaenopsis* cultivars. The results show that it is not difficult to obtain universal SSR markers by transcriptome deep sequencing in *Phalaenopsis* species.

**Conclusions:**

This study supported that transcriptome analysis based on deep sequencing is a powerful tool to develop SSR loci in non-model species. A large number of EST-SSR loci can be isolated, and about 33.33% EST-SSR loci are universal markers across the *Phalaenopsis* breeding germplasm after preliminary validation. The potential universal EST-SSR markers are highly valuable for identifying all of *Phalaenopsis* cultivars.

## Introduction

Moth orchids (*Phalaenopsis* spp.) are among the most graceful and popular plants. They consist of approximately 66 natural species worldwide, fifty-six of which are extant [1]. Based on the classification of Christenson [1], *Phalaenopsis* is divided into five subgenera, *Proboscidioides*, *Aphyllae*, *Parishianae*, *Polychilos*, and *Phalaenopsis* that are determined mainly by plant size and floral morphology (including callus, lip structure, pollinium number, and other characters). The subgenus *Polychilos* was further subdivided into four sections, including *Polychilos*, *Fuscatae*, *Amboinenses*, and *Zebrinae*. Additionally, the subgenus *Phalaenopsis* was also subdivided into four sections, *Phalaenopsis*, *Deliciosae*, *Esmeralda*, and *Stauroglottis*. *Phalaenopsis* species are found throughout tropical Asia and the larger islands of the Pacific Ocean. All *Phalaenopsis* species, excluding the natural tetraploid species *P*. *buyssoniana* Rchb.f., have 38 (2n = 38) chromosomes [1,2]. Recently, the plastid genome of *P*. *aphrodite* has been completely sequenced [3], and molecular phylogenies of *Phalaenopsis* species also have been constructed based on the internal transcribed spacer (ITS) of the ribosomal (rDNA) and plastid DNA [4,5,6,7]. Additionally, molecular data were used to determine the inheritance of the natural hybrid, *P*. *x intermedia*, showing that *P*. *aphrodite* was the maternal parent and *P*. *equestris* was the paternal parent [8]. More recently, complete genome sequencing has been conducted in *P*. *equestris* [9].

Random amplified polymorphic DNA (RAPD) has been conducted to reveal the phylogenetic relationship of 16 *Phalaenopsis* species [10]. Three-hundred-eighty-one RAPD makers derived from 20 primers were obtained. Chuang [11] examined several accessions of *Phalaenopsis aphrodite* subsp. *formosana* and several related *Phalaenopsis* species from the Philippines based on RAPD and inter-simple sequence repeat (ISSR) molecular markers. The results showed that these two molecular techniques could offer informative markers to separate those from samples that are closely related. Another RAPD analysis was conducted by Goh *et al*. [12]. They examined 149 accessions representing 46 species of genus *Phalaenopsis*, and four *Paraphalaenopsis* species were used as outgroups. Six out of twenty random primers were selected for analysis and 123 polymorphic bands have been obtained. Cluster analysis derived from the RAPD molecular markers showed that *Phalaenopsis* form seven groups and are basically congruent with previous studies derived from morphological characters

Generally, the high repeated motifs of microsatellites are prone to mutation through slipped-strand mispairing [13]. The relatively rapid mutation rate, and high frequency in genome have made SSRs to be popular markers for population genetics [14,15,16], hybrid detection [17], linkage mapping, genetic fingerprinting [18,19], evolutionary history [20,21], and taxonomy [22,23]. Young [24] examined DNA fingerprinting of 89 accessions of *Phalaenopsis amabilis* based on microsatellite DNA (simple sequence repeats, SSRs). Three SSR loci were cloned and evaluated from *P*. *amabilis* accessions. The results indicated that these loci are good molecular markers to identify intraspecific variation of *Phalaenopsis*. EST-SSRs separately developed from the *Phalaenopsis* ESTs database have obtained 42 [25] and 261 EST-SSR loci [26]. Nine-hundred-fifty potential SSRs in *Phalaenopsis equestris* were discovered by large-scale BAC end sequencing [27]. Deep sequencing technologies offer the possibility of generating numerous SSR markers much faster and at a lower cost compared to library-based methods [28,29,30,31,32].

Here, we performed *de novo* transcriptome deep sequencing of *P*. *aphrodite* subsp. *formosana* to analyze EST-SSR, develop molecular markers, and test the transferability between most members of *Phalaenopsis* that are used as parents for moth orchid breeding. To our knowledge, this is the first study to develop EST-SSRs by deep sequencing of transcriptomes in *Phalaenopsis* species. Furthermore, the developed EST-SSR markers in the present study can be applied for genetic diversity analysis, gene mapping, linkage map development, marker-assisted selection breeding, and cultivar identification in *Phalaenopsis* species/cultivars.

## Results

### Sequencing and *de novo* assembly

**Fig 1:**
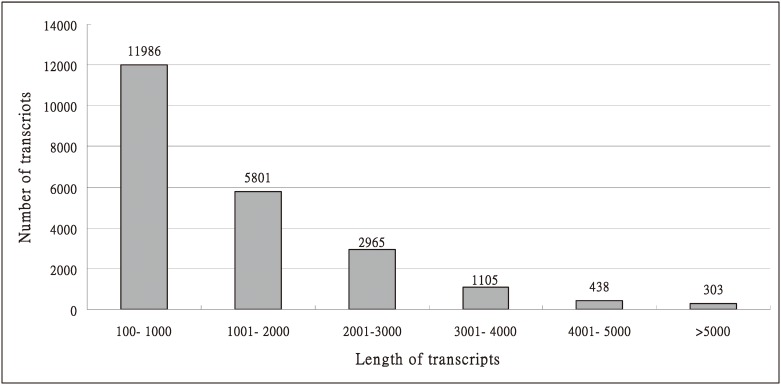
Summary distribution of the lengths of the 22,598 unigenes from raw reads (>100 bp, mean length = 1,286 bp, N50 = 2,047 bp, Min = 113 bp, Max = 16,771 bp).

A total of 21,396,423 (30–76 base, 46.5% GC) high-quality PE reads were generated from Sanger/Illumina 1.9 sequencing, approximately 4Gb of sequence data was obtained from leaves of *P*. *aphrodite* subsp. *formosona*. These short sequence reads have been deposited at NCBI as SRA accession number SRX1253908 and SRX1253909. The reads with high quality bases above Q20 were more than 90% indicated a very good quality calls bases (Q20 means 1 error per 100 sequenced bases) and with per sequence quality score above 38 (if the most frequently observed mean quality below 27 equates to a 0.2% error rate) mean a good quality. The high-quality PE reads were used for *de novo* assembly to join into scaffolds step-by-step, based on paired-end information. Finally, 22,598 unigenes (≥ 100 bp) were generated, with a final unigene N50 length of 2,047 bp and a total length of 29,062,410 bp (Table 1) (Fig 1).

**Table 1:** Summary of sequence assembly after Illumina sequence.

Total number of unigenes	22,598
Sum of scaffolds	29,062,410 bp
Max scaffolds size	16,771 bp
Min scaffolds size	113 bp
Average scaffolds size	1,286 bp
N50	2,047

### Frequency and distribution of different types of EST-SSR loci

**Fig 2:**
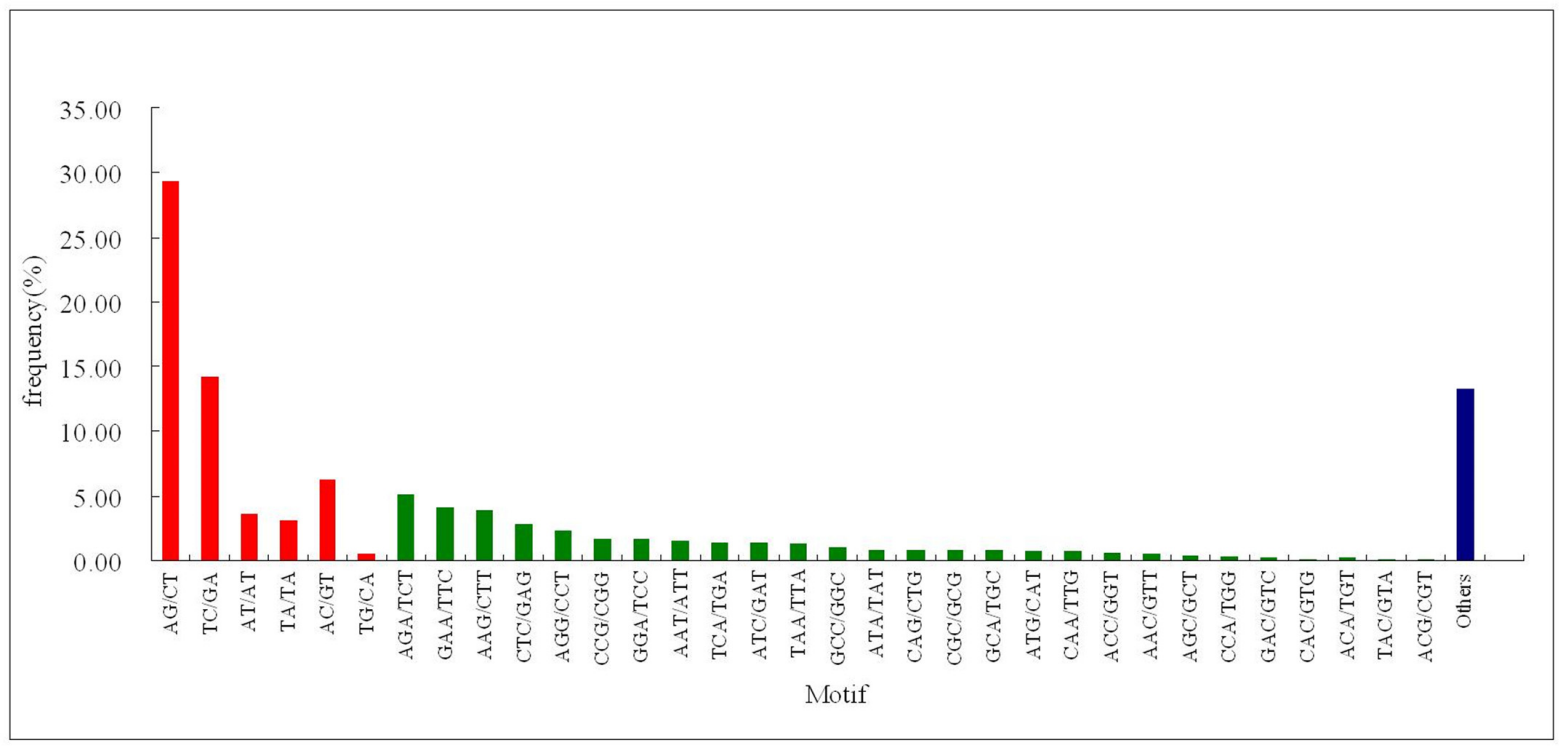
Summary distribution of EST-SSR loci repeat motifs. The number of EST-SSR loci repeat motifs derived from Solexa transcriptome *de novo* sequencing. Di-, tri-nucleotide motifs, and others were highlighted in red, green and blue boxes, respectively.

The 22,598 unigenes generated in this study were used to search potential microsatellites that were defined as perfect di-, tri-, tetra-, penta-, and hexa-nucleotide motifs with a minimum of 9, 6, 5, 5, and 4 repeats, respectively. There are potentially 1,439 EST-SSRs that can be found after SSR mining. The potential 1,439 EST-SSRs were further classified and shown in Table 2, the di-nucleotide repeats were shown to be the most abundant (741, 51.49%), followed by tri- (507, 35.23%), hexa- (121, 8.41%), tetra- (59, 4.10%), and penta- nucleotide repeats (11, 0.76%). Of di-nucleotide repeat motifs, the AG/CT di-nucleotide repeat was the most abundant motif (422, 29.33%), followed by TC/GA (205, 14.25%), and GC/GC was the rarest motif (0, 0%). Of tri-nucleotide repeat motifs, AGA/TCT was the most abundant motif (73, 5.07%), followed by GAA/TTC (59, 4.10%), AAG/CTT (56, 3.89%), and both ACG/CGT and TAC/GTA were the rarest motif (1, 0.07%) (Fig 2).

**Table 2:** Numbers of EST-SSRs identified and primer designation in *Phalaenopsis aphrodite* subsp. *formosana* in the study.

	Numbers of SSRs
Total number of unigenes	22598
Total number of identified EST-SSRs	1,439 (1,051)*
Dinucleotide	741 (507)
Trinucleotide	507 (421)
Tetranucleotide	59 (29)
Pentanucleotide	11 (6)
Hexanucleotide	121 (88)

*The number of suitable primer designation for PCR is shown in parentheses.

### Preliminary validation for the developed EST-SSR loci

**Fig 3:**
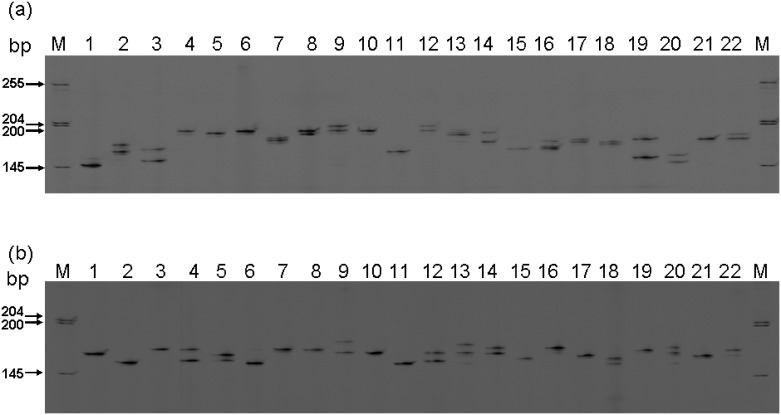
The polymorphism of 22 *Phalaenopsis* species detect by SSR-PCR analysis. (a) The polymorphism of *Phalaenopsis* taxa at Pap-3222 SSR locus. Lanes 1~22 represent 22 *Phalaenopsis* species listed in Table 5. (b) The polymorphism of *Phalaenopsis* taxa at Pap-4825 SSR locus. Lanes 1~22 represent 22 Phalaenopsis species listed in Table 5.

**Fig 4:**
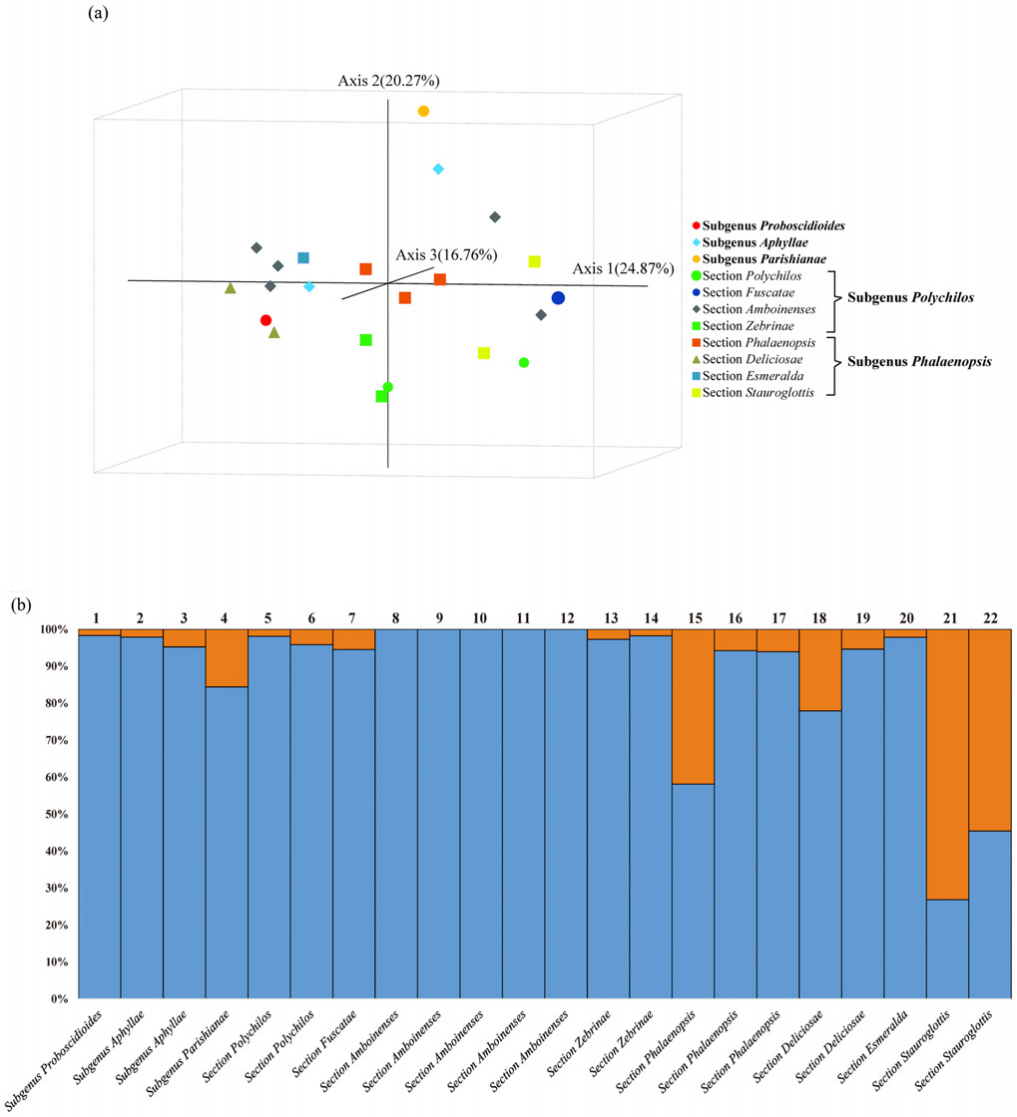
Estimated genotypic group structure for 22 moth orchids. (a) Using the first three axes in principle coordinate analysis (PCoA). (b) Using the assignment test with Bayesian clustering analysis on the best fit numbers (K = 2) of grouping based on 10 polymorphic microsatellite loci.

**Fig 5:**
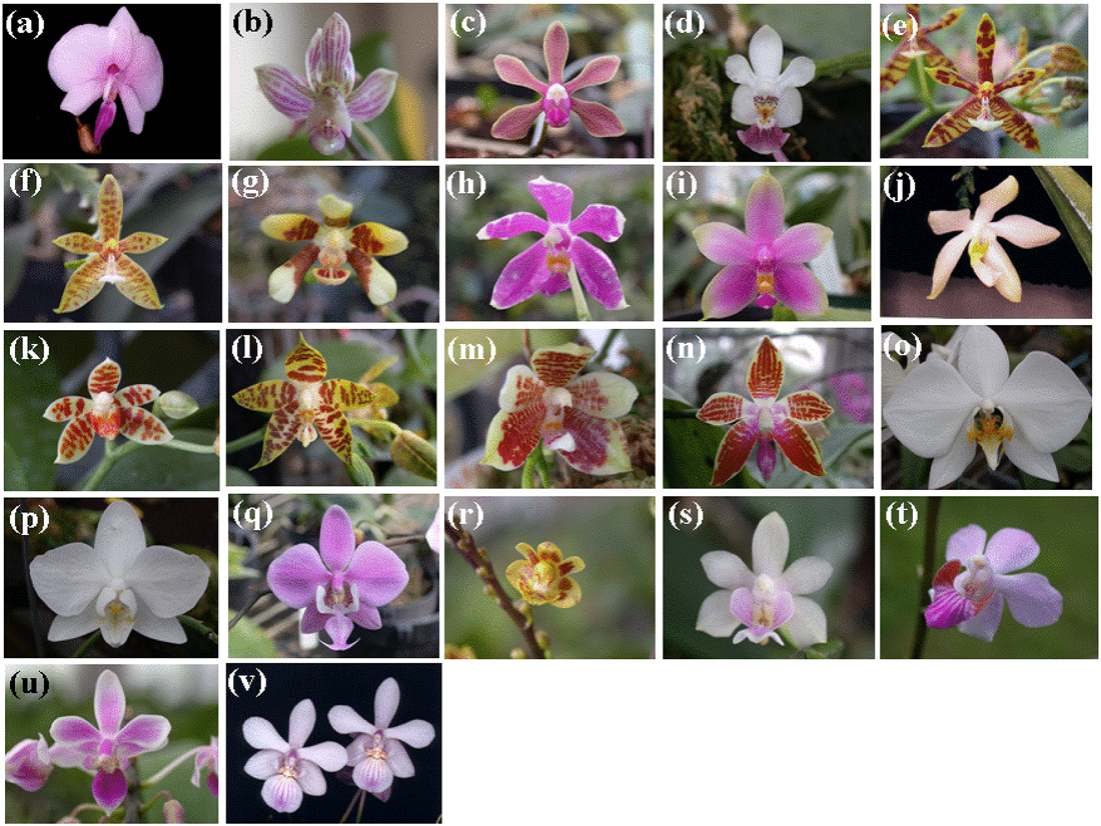
Images of 22 *Phalaenopsis* species. Images (a)-(v) represent samples 1–22 shown in Table 5.

Most EST-SSRs are either di- (51.49%) or tri-nucleotide motifs (35.23%). The average number of potential EST-SSRs per unigene is 0.064. Of the detected EST-SSRs, 1,051 EST-SSRs were obtained for suitable primer designation by BatchPrimer3 (Table 2). The information of EST-SSR primers in this study is shown in S1 Table. Of the 421 primer pairs for tri-nucleotide motifs, 30 EST-SSR loci were randomly selected to evaluate the polymorphism and transferability across 22 native *Phalaenopsis* species that are representative germplasms for breeding most *Phalaenopsis* cultivars. Of these, ten EST-SSR loci were stably amplified, polymorphic, and transferrable SSR loci across 22 native *Phalaenopsis* species (S1 Fig). In total, 70 amplifying bands were detected by 10 primer pairs across 22 native *Phalaenopsis* species, and the number of amplifying bands per primer pairs ranged from 3 to 16, with an average of 7. The polymorphism information content (PIC) value across 22 native *Phalaenopsis* species ranged from 0.163 to 0.889, with an average of 0.588 (Table 3). The amplified products derived from EST-SSR PCR across 22 native *Phalaenopsis* species are shown to be one or two bands for most of SSR loci, such as the loci Pap-3222 (Fig 3a) and Pap-4825 (Fig 3b). Genetic similarity between 22 native moth orchids was evaluated by principal coordinate analysis (PCoA), and the three-dimensional representation provided by the plot shows a certain degree of separation between different species (Fig 4a). The resolution of the first, second and third axes show 24.87%, 20.27%, and 16.76% of the variance, respectively. Compared to 22 native taxa (Fig 5) by 10 polymorphic EST-SSR loci, genetic compositions among different species are obviously scattered between taxa but can be grouped at the three axes on Sections *Zebrinae*, *Phalaenopsis*, *Deliciosae*, and *Stauroglottis* (Fig 4a).

**Table 3:** The evaluation of the polymorphism and transferability of 10 EST-SSR primers across 22 native *Phalaenopsis* species.

SSR loci	Motif	Primer sequence (5’→3’)	Size	No. of sample	No. of alleles	Heterozygosity (H)	PIC	Ta (°C)
Pap-1059	(CGC)_8_	F:AGAAGTTCGATTCTGCTATGA	150–135	22	3	0.091	0.163	55
		R:GGGAAGGAAAGAGAGATGTAA						
Pap-1358	(TCA)_8_	F:CTGACGGAAGATTGAAAATTA	180–135	22	9	0.682	0.818	55
		R:TGGTCTTCGGTAAGAAGTATG						
Pap-1520	(GCT)_8_	F:ATCAGCCTTCATGATCTTCTT	152–128	22	4	1.000	0.491	55
		R:AACTCTACCACCATCAGCAG						
Pap-1904	(GCG)_8_	F:GGTTGCATTTGAACTTGAATA	183–180	22	3	0.000	0.163	55
		R:CCCCAATTCTCAAATTTCTAT						
Pap-3222	(GAG)_8_	F:GAGTATTGAATCCCCAAGTTT	180–126	22	16	0.546	0.889	55
		R:TTCAGAATCATCTTTCTCCTG						
Pap-3268	(AAC)_9_	F:TAACTCGCCTTCTCGTCTTA	160–145	22	6	0.182	0.601	55
		R:TTTTTCCATTACTGTTTGATGA						
Pap-3754	(TCC)_8_	F:AGTCTGAAGCTTCTTCTTGCT	151–136	22	6	0.182	0.597	55
		R:CAATATAGAGGAGGAGCAGGT						
Pap-4282	(AGA)_8_	F:CTATGCTTCCCACAGAAACC	213–185	22	11	0.818	0.862	55
		R:CTGTGATCCACCATCCTTAC						
Pap-4356	(AAG)_8_	F:CTATTGTGAAGAAGGAGGTGA	158–310	22	4	0.182	0.496	55
		R:CTGTTACTAACCTGCGTTGAT						
Pap-4825	(CTC)_8_	F:ACCAGCTTCTACATTTCCAAT	162–141	22	8	0.318	0.803	55
		R:AAGATCTTCATTGATCCTTTTG						
			Average		7	0.400	0.588	

The best fit numbers of grouping is inferred as two by the ΔK evaluation (ΔK = 64.57 when K = 2) in the Bayesian clustering analysis. Two genetic components were estimated using assignment test and each taxon was either high percentage of component 1 (Blue color on Fig 4b) or component 2 (Orange color on Fig 4b), except three taxa including *Phalaenopsis amabilis* (Taxon abb. 15) belong to Section *Phalaenopsis*, *Phalaenopsis equestris* (Taxon abb. 21), and *Phalaenopsis lindenii* (Taxon abb. 22) belong to Section *Stauroglottis*, revealed an admixture genetic components. Based on the best fit number of grouping, 22 taxa of *Phalaenopsis* were divided into two groups (Fig 4b). The first group with high percentage of component 1 are included 20 taxa of *Phalaenopsis*, except 2 taxa, *Phalaenopsis equestris* and *P*. *lindenii* of Section *Stauroglottis* were grouped into the second group.

### Application of validated EST-SSR loci for *Phalaenopsis* cultivars identification

**Fig 6:**
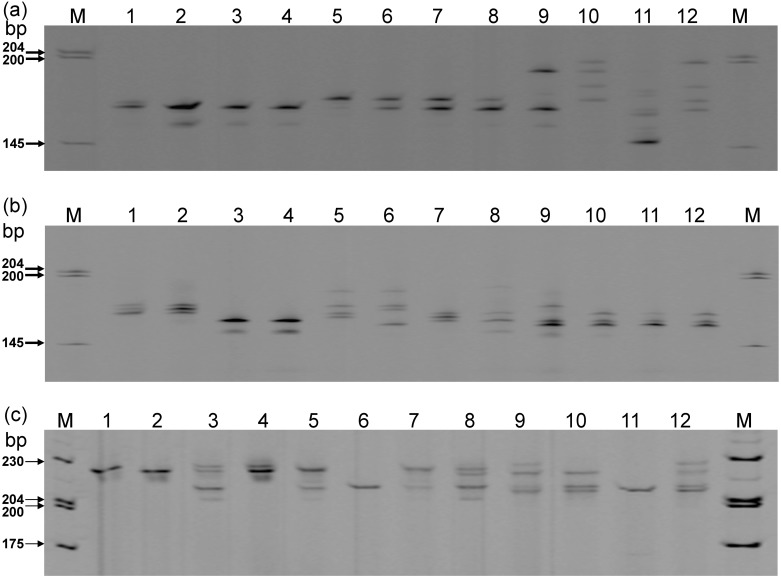
The polymorphism of 12 *Phalaenopsis* varieties by SSR-PCR analysis. The polymorphism of 12 *Phalaenopsis* varieties at (a) Pap-3222 SSR, (b) Pap-4825 SSR, and (c) Pap-4282 SSR loci. Lanes 1–12 represent 12 *Phalaenopsis* varieties/lines listed in Table 4. Lanes 1–4 represent four similar commercialized cultivars with white floral color; Lanes 5–8 represent four similar commercialized cultivars with yellow floral color; Lanes 9–12 represent four similar commercialized cultivars with red floral color.

**Fig 7:**
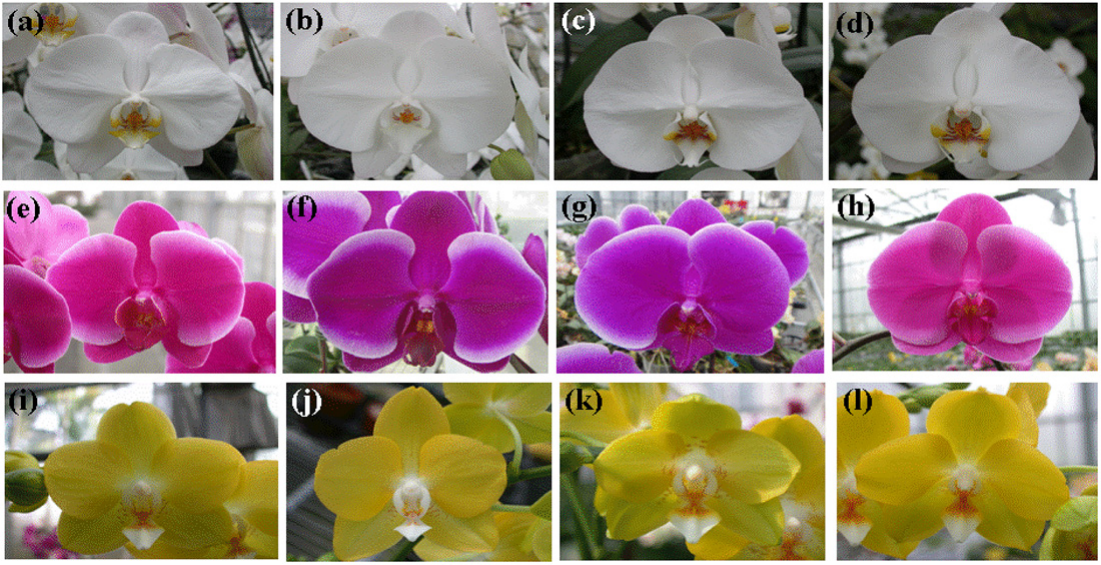
Images of 12 *Phalaenopsis* varieties. Images (a)-(l) represent samples 1–12 shown in Table 4.

The EST-SSRs studied were further used to identify 12 commercialized *Phalaenopsis* cultivars, including white, red and yellow floral color groups (Table 4). The morphological characters of the same floral color of plant materials studied are very similar. It is not easy to identify them based on either vegetative or reproductive characters (such as floral color, size, and morphology). Three validated polymorphic and transferable primer pairs (i.e., SSR loci Pap-3222, Pap-4825, and Pap-4282) for EST-SSR were selected to discriminate 12 commercialized *Phalaenopsis* cultivars. According to the amplified PCR products, more than two bands can be found within an individual (Fig 6). In white floral color group (Fig 7a–7d), each of cultivars can be identified according to both SSR loci Pap-3222 and Pap-4282 (Fig 6a). In red floral color group (Fig 7e–7h), each of cultivars can be identified by using SSR locus Pap-4825 (Fig 6b). In yellow floral color group (Fig 7i–7l), each of cultivars can be identified by using SSR locus Pap-3222 (Fig 6c). Using the aforementioned three EST-SSR markers, each of the 12 commercialized *Phalaenopsis* cultivars can be discriminated.

**Table 4:** The twelve commercialized *Phalaenopsis* cultivars studied.

Abb.	Cultivars name	Floral color	Flower size in diameter (cm)
1	*P*. Sogo Yukidian ‘V3’	white	12–13
2	*P*. Sogo Musadian	white	12–13
3	*P*. I-Hsin Diamond	white	12–13
4	*P*. Chainport Dorothy	white	12–13
5	*P*. Ruey Lih Beauty	red	8–10
6	*P*. Shiuh-Dong Red Rose ‘Fantasy Rose’	red	8–10
7	*P*. Ruey-Lih Red Rose	red	8–10
8	OX 1172	red	8–10
9	*P*. Sogo Meili	Yellow	6–7.5
10	Sogo F3005	Yellow	6–7.5
11	*P*. Sogo Shito ‘Sogo F2999’	Yellow	6–7.5
12	*P*. Sogo Sweet	Yellow	6–7.5

## Discussion

The genome size of *Phalaenopsis aphrodite* subsp. *formosana* was estimated to be approximately 1,300 Mb (2.81 pg/diploid genome), which is relatively small compared to other *Phalaenopsis* species [33,34]. After *de novo* assembly of deep sequencing data, the obtained sequence covered 29.1 Mb, or approximately 2.2% of the *P*. *aphrodite* subsp. *formosana* genome. Excluding mono-nucleotide repeats that are not nearly useful for molecular markers [35], a total of 1,439 EST-SSR loci, including di-, tri-, tetra-, penta-, and hexa-nucleotide motifs, were detected across the transcriptome of 29.1 Mb in *P*. *aphrodite* subsp. *formosana*. Excluding mono-nucleotide repeat motifs, di-nucleotide repeat motifs of EST-SSRs were the most abundant type (51.49%) of microsatellites in the study. This result is consistent with EST-SSR studies in loblolly pine and spruce [36]. *Pinus contorta* [37], blueberry [38], rubber tree [39]. In contrast, tri-nucleotide motifs are also revealed to be the most abundant type of EST-SSRs in several studies [40,41,42,43], which are consistent with the maintenance of the open reading frame (ORF) coding. The untranslated regions (UTRs) are richer in SSRs than coding regions, particularly the 5'-UTRs [43,44,45]. Thus, the most abundant type of di-nucleotide repeats in the *P*. *ahrodite* subsp. *formosana* and some plants may result from their high proportion of 5’UTR-SSRs.

SSR occurs in the exon region, including a 5’ UTR, CDS, and 3’UTR, on average every 20.22 kb in *P*. *aphrodite* subsp. *formosana*. The average density of EST-SSRs was likely shown to be relatively low frequency in *P*. *aphrodite* subsp. *formosana* compared to most other species; such as one EST-SSR is found every 14 kb in *Arabidopsis* [46], every 19 kb in rice [47], every 19.4 kb averaged across maize, rice, soybean, and wheat [48], and every 1.77 kb in castor bean [42]. Although the frequencies of EST-SSRs were shown to vary in different species, these results may not be accurate because of the different strategies that were used to mine the EST-SSRs. In this study, the EST-SSR mining parameters were set to search perfect di-, tri-, tetra-, penta-, and hexa-nucleotide motifs with a minimum of 9, 6, 5, 5, and 4 repeats, respectively. The parameters are relatively stringent compared to other studies; for example, the EST-SSR mining parameter in castor bean was set to identify perfect mono-, di-, tri-, tetra-, penta- and hexa-nucleotide motifs with a minimum of 10, 5, 4, 4, 4, and 4 repeat subunits, respectively. The study included mono-nucleotide repeats and a parameter with low repeat number (a cut-off value of 5) on di-nucleotide SSR mining resulting of the high average density of EST-SSRs (an EST-SSR every 1.77 kb) in castor bean [42]. Using the same SSR mining parameters, the frequency of SSR loci in *P*. *aphrodite* subsp. *formosana* is higher than that of loblolly pine and spruce, which on average have an EST-SSR every 49.8 kb [36]. Therefore, the average density of EST-SSRs cannot be compared among different species if the mining parameters are not identical. Because both di-nucleotide and tri-nucleotide repeats are the two most abundant types of microsatellites [35], the average density of SSRs is highly dependent on the parameter for the minimum repeat units of di- and tri-nucleotide repeat types of microsatellites.

Di-nucleotide repeat units in *P*. *aphrodite* subsp. *formosana*, AG motifs were the most frequent SSR motifs, about 29.33% of the total isolated loci and the lowest frequency (0%) in GC repeat di-nucleotide repeat units in the study. Similar results also can be found in SSR loci of other plants derived from either EST-SSR [39,42,43] or genome-wide SSR [42,43,45,49,50,51]. In tri-nucleotide repeat units, AGA, GAA, and AAG are the three highest microsatellite frequencies in *P*. *aphrodite* subsp. *formosana*. These results are consistent with those found in SSR loci of other plants derived from either EST-SSRs [39,42,43] or genome-wide SSRs [42,43,45,47,50,51]. According to this study and previous studies, both AG and AGA/GAA/AAG repeat units are shown to be high frequency SSR motifs in most of the clarified plants. AG or AGA/GAA/AAG repeat motifs in the 5’UTR upstream region of genes were thought to play significant roles in regulating gene expression and translation in *Arabidopsis* [52,53], and positive selection of AG and AGA/GAA/AAG repeat motifs respectively can be found in the 5’UTR and 5’coding region of *Arabidopsis* [43].

GC repeat units in low frequency of SSR motifs are shown in most SSR transcriptome-wide studies as previously described. GC-rich regions might be relatively stable, resulting in less replication slippage [54,55]. Furthermore, GC-rich and AT-rich motifs are respectively found in exon and intron regions for the splice site recognition in plant genes [56,57]. Additionally, the coding region of di-nucleotide SSR motif repeats occurs less frequently because of functional constraints, therefore di-nucleotide SSRs were preferentially concentrated in 5’-and 3’ untranslated regions (UTR) [39,58,59], as well as both 5’ and 3’UTR regions usually show AT-rich motifs, which is implicated in mediating RNA stability [60]. Overall, these patterns might lead to the low efficiency of GC repeat unit SSR motifs in plants. The low frequency of GC repeat units of EST-SSR motifs has been revealed in various species, from yeast, plants, and vertebrates [35].

Deep sequencing clearly offers a rapid strategy of acquiring the sequences required to discover SSRs and to design specific primers to obtain useful SSR markers. Additionally, EST-SSR markers usually have a higher amplification efficiency, and are more likely to be transferable across species than SSR markers derived from non-coding regions of the genome [61,62,63,64].

To the present, there is no universal SSR markers across genus *Phalaenopsis*, even though several studies have focused on SSR mining in *Phalaenopsis* with transferable evaluation across part germplasm of the genus [24,25,27], or with transferable evaluation by using several cultivars [26]. To develop universal SSR markers for all commercialized *Phalaenopsis* cultivars, the transferability and polymorphisms of SSR loci cloned form EST-SSRs require validation. According to the molecular phylogeny of *Phalaenopsis* [6] and the orchid hybrid database at the Royal Horticultural Society (RHS), twenty-two native *Phalaenopsis* species were selected from the representatives of all subgenera and sections, which include most of the breeding parents for historical *Phalaenopsis* breeding programs. To develop universal SSR markers for all commercialized *Phalaenopsis* cultivars, EST-SSR markers were isolated and screened, resulting from having a higher amplification efficiency and are more likely to be transferable across species than those derived from non-coding regions of the genome [62].

Di-nucleotide repeat unit microsatellites always have larger repeat numbers and high levels of polymorphism in diverse plants [51,65,66,67]. Although the performance of higher polymorphism derived from di-nucleotide repeat units implied that these markers could be used efficiently, the higher efficiency of SSR-PCR stutter products will be easily found in di-nucleotide repeat units with larger repeat numbers [68,69]. Thus, tri-nucleotide, tetra-nucleotide or penta-nucleotide repeats usually amplify more faithfully by PCR than di-nucleotide repeats [70]. The stutter products result from the slipped-strand mispairings as a natural process of SSR mutation *in vivo* [71]. SSR markers will interfere with the high ratio of stutter products, especially in polyploidy plants [72]. Because *Phalaenopsis* cultivars have different ploids, including diploids, triploids and tetraploids [73], di-nucleotide repeat units of SSR motifs might not be suitable for plant identification. In a study of transcriptome-wide EST-SSR searching, 4–6 nucleotide repeat units of SSR motifs are at too low of a frequency to develop transferable and polymorphic SSR markers across the diversified breeding germplasm of *Phalaenopsis* cultivars; thus, tri-nucleotide repeat unit microsatellites are considered to be suitable SSR markers to identify *Phalaenopsis* cultivars.

Of 507 potential tri-nucleotide microsatellites, 421 were suitable for primer designation. Of these, 30 tri-nucleotide microsatellites were randomly selected to evaluate the transferability and polymorphism across the 22 native *Phalaenopsis* species which are usually used as parents for moth orchid breeding. The result showed that the transferability of the EST-SSRs across the 22 native *Phalaenopsis* species is approximately 33.33% (10/30). According to the PCoA results, the resolution of the first three axes explain 61.91% of the variation between species EST-SSR multilocus genotypes. The distribution of results in the three dimensional plot were clearly scattered between taxa, but can be grouped on cross related species of Sections *Zebrinae*, *Phalaenopsis*, *Deliciosae*, and *Stauroglottis*. Based on the Bayesian clustering analysis, 22 moth orchid divided into two groups and the Section *Stauroglottis* was separated out of others. This provides evidence of genetically distinct units in native moth orchids and potential molecular tools to identify commercialized cultivars/lines. The data were sampled from across five subgenera of the genus *Phalaenopsis* [1]. Previous systematic studies have indicated that this genus included members of two genera, *Phalaenopsis* and *Doritis* [74]. In the Orchidaceae, hybrids derived not only from different species but also different genera are continually crossed, and F1 hybrids from more advanced generations have been produced on a large scale [75]. In moth orchids, there are over 30,000 *Phalaenopsis* cultivars registered in the RHS orchid hybrid database. The results show that the crossing barriers within orchidaceous plant genera are relatively low. Additionally, the amplified products derived from EST-SSR PCR for 22 native *Phalaenopsis* species are shown to be one or two bands, respectively, showing homozygosity and heterozygosity as described by several studies [76,77].

In the analysis of 12 commercialized *Phalaenopsis* cultivars, more than two bands may exit for an individual, as shown on Fig 6. The plant materials could be explained as polyploids as described by Diwan et al. [78]. This result is consistent with the chromosome karyotype of commercialized *Phalaenopsis* cultivars that are often triploid or tetraploid [79]. Additionally, 12 *Phalaenopsis* cultivars can be differentiated from one another based on the analysis of three SSR loci (Fig 6). Thus, the EST-SSR markers developed in this study could be efficiently used to differentiate closely related *Phalaenopsis* cultivars.

## Conclusions

The study shows that transcriptome analysis based on deep sequencing is a powerful tool to develop EST-SSR loci in non-model species. A total of 1,439 EST-SSR loci from *Phalaenopsis* species were obtained. After the preliminary validation of EST-SSR loci, about 33.33% EST-SSR markers are transferable across the *Phalaenopsis* breeding gremplasm. These characterized and uncharacterized universal EST-SSR markers can be potentially applied to identify all of *Phalaenopsis* cultivars in the future.

## Materials and Methods

### Plant materials

Twenty-two *Phalaenopsis* taxa were obtained from wild populations and cultivated in the greenhouse at Kaohsiung District Agricultural Research and Extension Station in Taiwan by C. C. Tsai. Voucher specimens were deposited at the herbarium of the National Museum of Natural Science, Taiwan (TNM) and are listed in Table 5 and Fig 5. To test the transferability for commercialized *Phalaenopsis* cultivars, twelve commercialized varieties were collected for study and are listed in Table 4 and Fig 7.

**Table 5:** Names of the specimens, geographical distributions, and sources for the plant material used in the study.

Taxon abb.	Taxa	Systematics^a^	Geographical distribution	Source^b^
1	*Phalaenopsis lowii* Rchb.f. ^b^	Subgenus *Proboscidioides*	Myanmar and adjacent western Thailand	KDAIS KC-88
2	*Phalaenopsis minus* (Seidenf.) E. A. Christ.	Subgenus *Aphyllae*	Endemic to Thailand	KDAIS KC-227
3	*Phalaenopsis braceana* (J. D. Hook.) E. A. Christ.	Subgenus *Aphyllae*	Bhutan and China	KDAIS KC-289
4	*Phalaenopsis parishii* Rchb.f.	Subgenus *Parishianae*	Eastern Himalayas, India, Myanmar, and Thailand	KDAIS KC-316
5	*Phalaenopsis mannii* Rchb.f.	Subgenus *Polychilos* Section *Polychilos*	Northeast India, Nepal, and China to Vietnam	KDAIS KC-22
6	*Phalaenopsis cornu-cervi* (Breda) Bl. & Rchb.f.	Section *Polychilos*	Northeast India and the Nicobar Islands to Java and Borneo	KDAIS KC-23
7	*Phalaenopsis fuscata* Rchb.f.	Section *Fuscatae*	Malaysia (Malay Peninsula), Borneo (West Koetai)	KDAIS KC-115
8	*Phalaenopsis pulchra* (Rchb.f.) Sweet	Section *Amboinenses*	Endemic to the Philippines (Luzon and Leyte)	KDAIS KC-17
9	*Phalaenopsis violacea* Witte	Section *Amboinenses*	Indonesia (Sumatra) and Malaysia (Malay Peninsula)	KDAIS KC-153
10	*Phalaenopsis micholitzii* Rolfe	Section *Amboinenses*	Philippines (Mindanao)	KDAIS KC-382
11	*Phalaenopsis maculata* Rchb.f.	Section *Amboinenses*	Malaysia (Pahang), East Malaysia (Sabah and Sarawak), and Indonesia (Kalimantan Timur)	KDAIS KC-49
12	*Phalaenopsis amboinensis* J. J. Sm.	Section *Amboinenses*	Indonesia (Molucca Archipelago and Sulawesi)	KDAIS KC-157
13	*Phalaenopsis inscriptiosinensis* Fowlie	Section *Zebrinae*	Endemic to Indonesia (Sumatra)	KDAIS KC-298
14	*Phalaenopsis corningiana* Rchb.f.	Section *Zebrinae*	Borneo (Sarawak and elsewhere on the island)	KDAIS KC-346
15	*Phalaenopsis amabilis* (L.) Blume	Subgenus *Phalaenopsis* Section *Phalaenopsis*	Widespread from Sumatra and Java to the southern Philippines, and east to New Guinea and Queensland, Australia	KDAIS KC-96
16	*Phalaenopsis aphrodite* subsp. *formosana*	Section *Phalaenopsis*	Taiwan	
17	*Phalaenopsis schilleriana* Rchb.f.	Section *Phalaenopsis*	Endemic to the Philippines	KDAIS KC-429
18	*Phalaenopsis chibae* Yukawa	Section *Deliciosae*	Endemic to Vietnam	KDAIS KC-488
19	*Phalaenopsis deliciosa* Rchb.f.	Section *Deliciosae*	Widespread from Sri Lanka and India to the Philippines and Sulawesi	KDAIS KC-255
20	*Phalaenopsis pulcherrima* (Lindl.) J. J. Sm.	Section *Esmeralda*	Widespread from northeast India and southern China throughout Indochina to Malaysia (Malay Peninsula), Indonesia (Sumatra), and East Malaysia (Sabah)	KDAIS KC-256
21	*Phalaenopsis equestris* (Schauer) Rchb.f.	Section *Stauroglottis*	Philippines and Taiwan	KDAIS KC-203
22	*Phalaenopsis lindenii* Loher	Section *Stauroglottis*	Endemic to the Philippines	KDAIS KC-119

^a^ The systematic characterizations of *Phalaenopsis* are based on Christenson (2001).

^b^ Plant materials were cultivated at the Kaohsiung District Agricultural Improvement Station (KDAIS), Taiwan; their voucher specimens were deposited at the herbarium of the National Museum of Natural Science, Taiwan (TNM).

### RNA extraction, cDNA library construction, sequencing, Data filtering, *de novo* assembly

For Illumina transcriptome deep sequencing, total RNA was extracted from the fresh leaves of *P*. *aphrodite* subsp. *formosana* using the RNeasy Plant Mini Kit (Qiagen, Germany) according to the manufacturer’s protocol. RNA quantity and quality was verified using NanoDrop ND 1000 (Thermo Scientific, Hudson, NH, USA) and 2100 Bioanalyzer (Agilent Technologies), respectively. The cDNA library was constructed according to the manufacturer’s instructions for the mRNA-Seq Sample Preparation Kit (Illumina Inc., San Diego, CA). The constructed paired-end library was prepared by using the Genomic Sample Preparation Kit (Illumina) according to the manufacturer’s instructions. After validation on an Agilent Technologies 2100 Bioanalyzer, the library was sequenced using Illumina HiSeq^™^ 2000 (Illumina Inc., San Diego, CA, USA) according to the manufacturer’s instructions. *De novo* transcriptome assembly for the high quality reads (Q < 20) was performed using Trinity software [79].

### Isolation of EST-SSR loci and Primer Designation

SSR loci were isolated in all of the unigenes from *P*. *aphrodite* subsp. *formosana* with SciRoKo 3.4 software [80]. The searching parameters of SSR loci were set to identify perfect di-, tri-, tetra-, penta-, and hexa-nucleotide motifs with a minimum of 9, 6, 5, 5, and 4 repeats, respectively. SSR motifs and their complementary SSR motifs were considered the same type of SSR motifs, such as those that were subsequently classified according to theoretically possible combinations, such as an AAG equivalent to CTT on a complementary strand. The specific primers for each of EST-SSR loci were separately designed using BatchPrimer3 developed by You et al. [81]

### DNA extraction and EST-SSRs PCR amplification

To validate the polymorphism and transferability of the EST-SSR markers derived from transcriptome deep sequencing, 22 native *Phalaenopsis* species and 12 commercialized cultivars were the plant materials respectively examined. One hundred milligrams of fresh leaves was ground in liquid nitrogen, and genomic DNA was extracted using the CTAB method [82]. For economic validation purposes, the designed forward primers for each of the SSR loci were elongated from the M13 (-21) 18 bp sequence (5’-TGTAAAACGACGGCCAGT-3’) to inexpensively label PCR products as described by Schuelke [83]. PCR conditions and IRDye label procedure were referenced from Tsai et al [19]. The labeled PCR products were denatured in loading dye (10 mg/ml blue dextran in formamide), and separated by 6.5% polyacrylamide gel (19:1, 7 M urea) electrophoresis using LI-COR 4300 DNA analyzer (LI-COR, Lincoln, Nebraska USA). Allele sizes were determined using IRDye 700 size standards (50–350 bp, LI-COR). The experiments were repeated three times and only the target bands consisted with three separate experiment were used for genotyping.

### Statistical analysis

The degree of polymorphism, including the number of amplifying bands per primer pairs with an average and the polymorphism information content (PIC) value were calculated using PowerMarker version 3.25 [84]. A principle coordinate analysis (PCoA) was performed to evaluate the degree of separation between different species. The PCoA was conducted with GenAlEx ver. 6.4 [85]. To evaluate the assistance of genotyping group information, the genetic clustering algorithms based on Bayesian-clustering approach were using by using the program STRUCTURE ver. 2.3.4 [86]. The posterior probability of the genetic groups from 1 to 22 was estimated using the Markov chain Monte Carlo (MCMC) approach based on the admixture model with 20 separate runs for each possible group to estimate the stability. Each run contained of 1,000,000 burn-in steps followed by 10,000,000MCMCsteps. To evaluate the best fit number of grouping, the ΔK method [87] by STRUCTURE HARVESTER v. 0.6.8 [88] was using.

## Supporting Information

S1 Fig The polymorphism of 22 *Phalaenopsis* species at ten characterized EST-SSR loci in the study. Lanes 1~22 represent 22 *Phalaenopsis* species listed in Table 4.
(PDF)

S1 Table The information of EST-SSR primers of the study. (PDF)
