# Supplementary material for: RNA-Seq SSRs of Moth Orchid and Screening for Molecular Markers across Genus Phalaenopsis (Orchidaceae)
Source: PLoS One. 2015 Nov 2;10(11):e0141761. doi: 10.1371/journal.pone.0141761 (PMC4629892; doi:10.1371/journal.pone.0141761)

S1 Fig. The polymorphism of 22 Phalaenopsis species at ten characterized EST-SSR loci in the study. Lanes 1~22 represent 22 *Phalaenopsis* species listed in Table 4.

(A) Locus Pap-1059

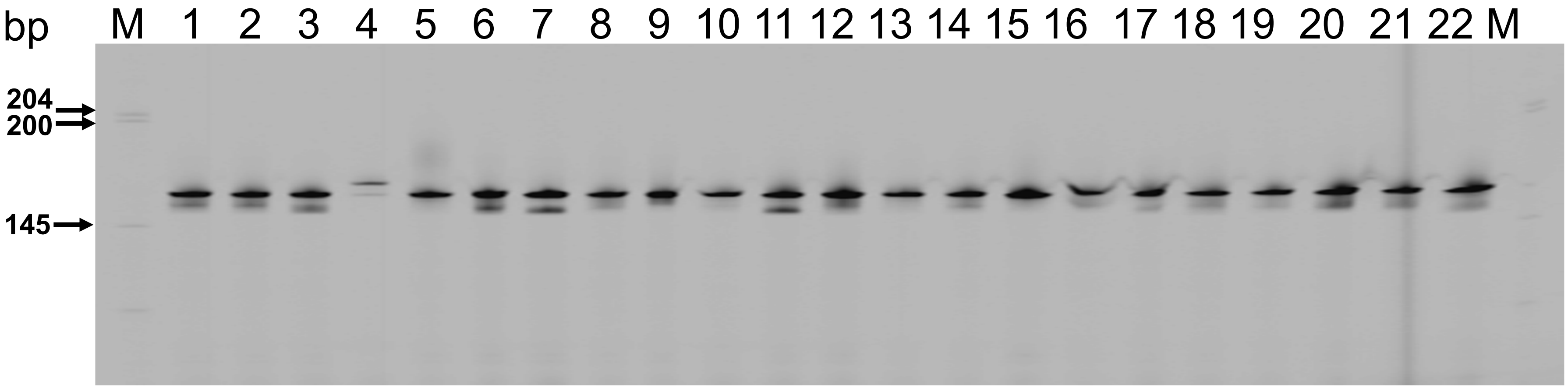

(B) Locus Pap-1358

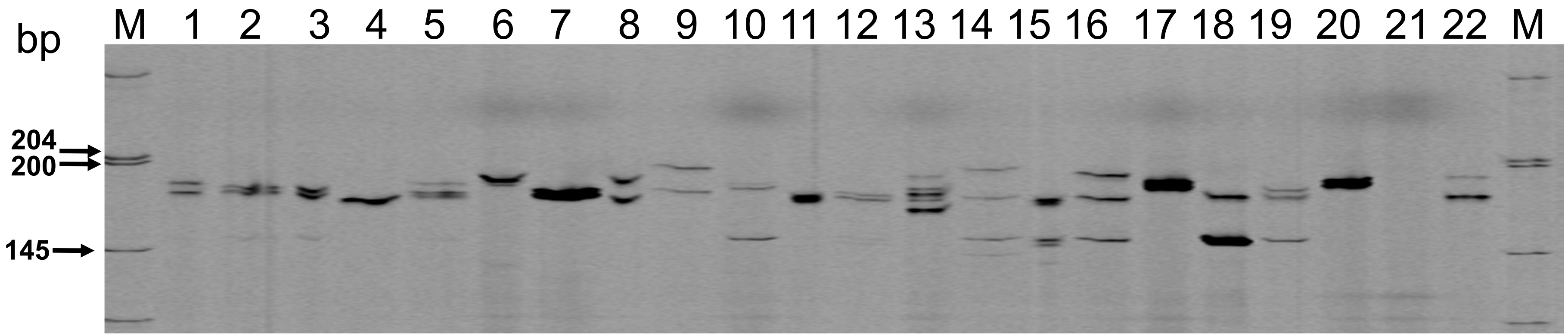

(C) Locus Pap-1520

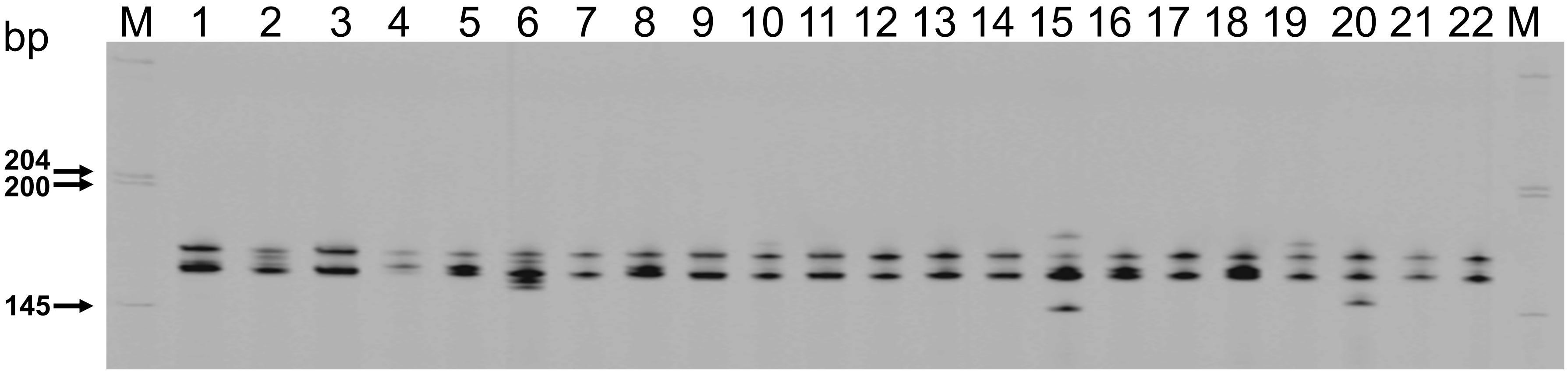

(D) Locus Pap-1904

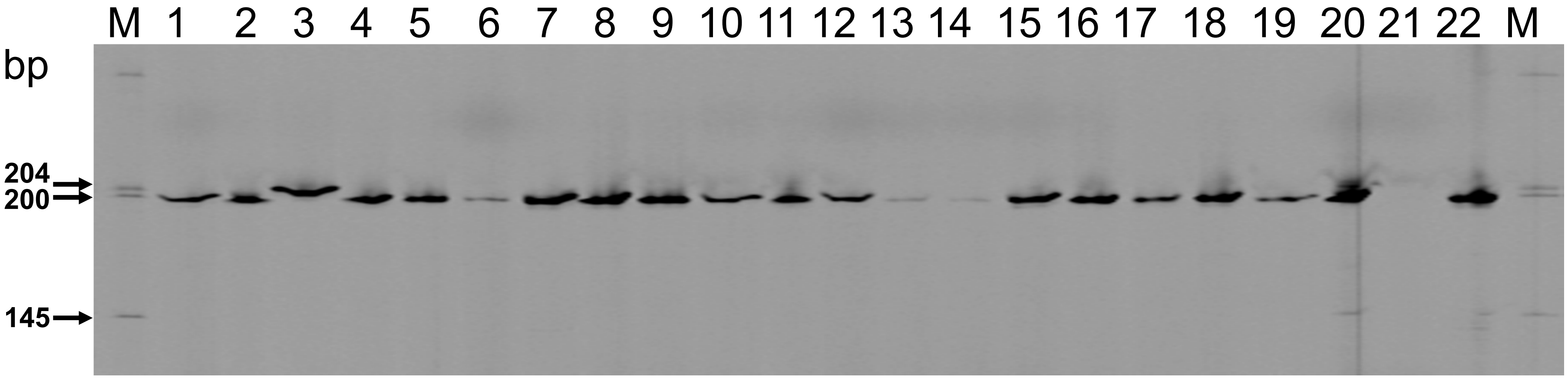

(E) Locus Pap-3222

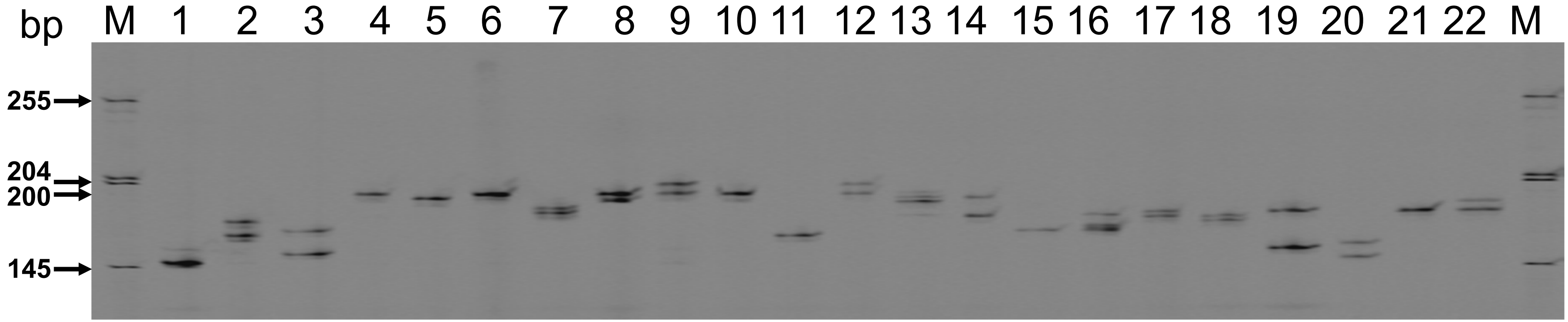

(F) Locus Pap-3268

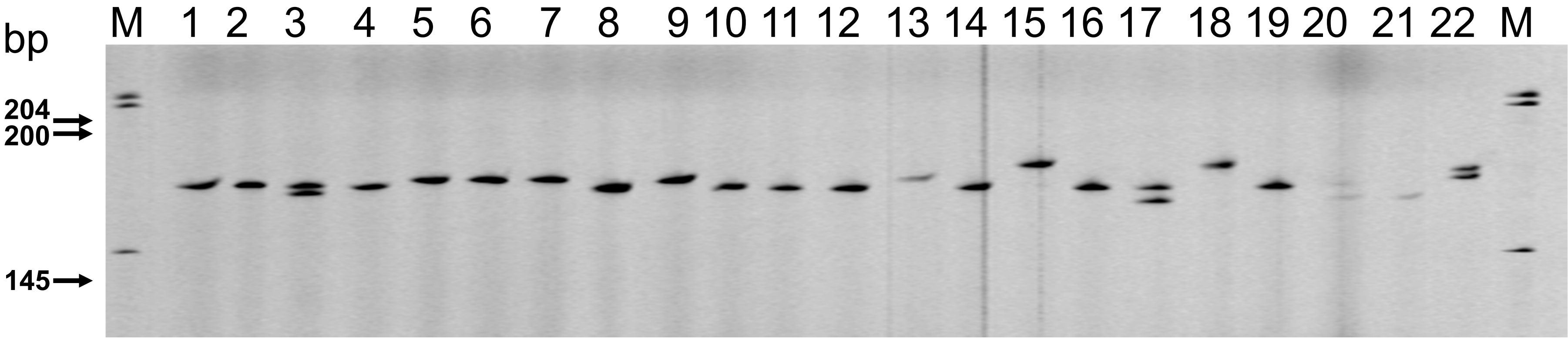

(G) Locus Pap-3754

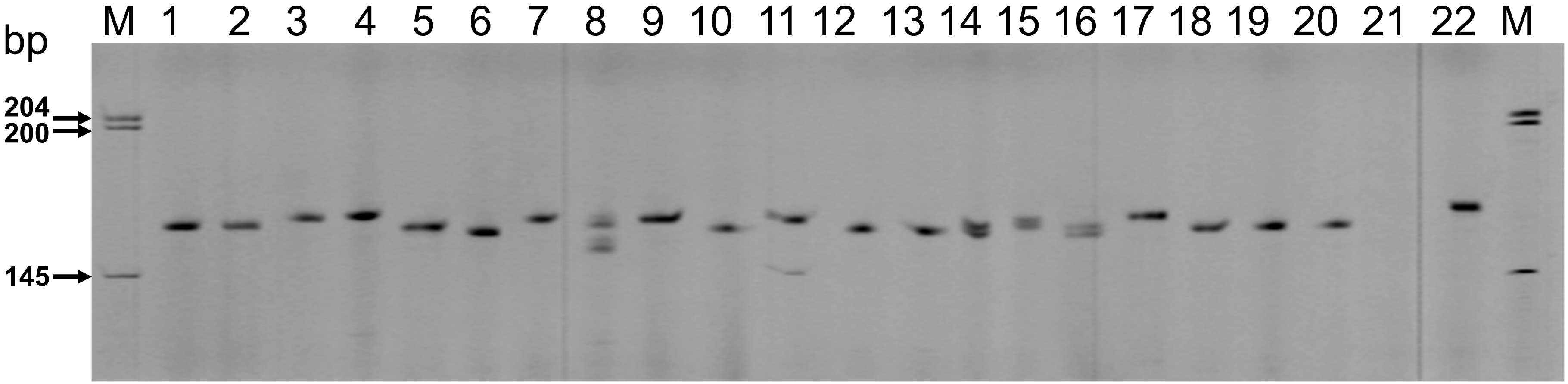

(H) Locus Pap-4282

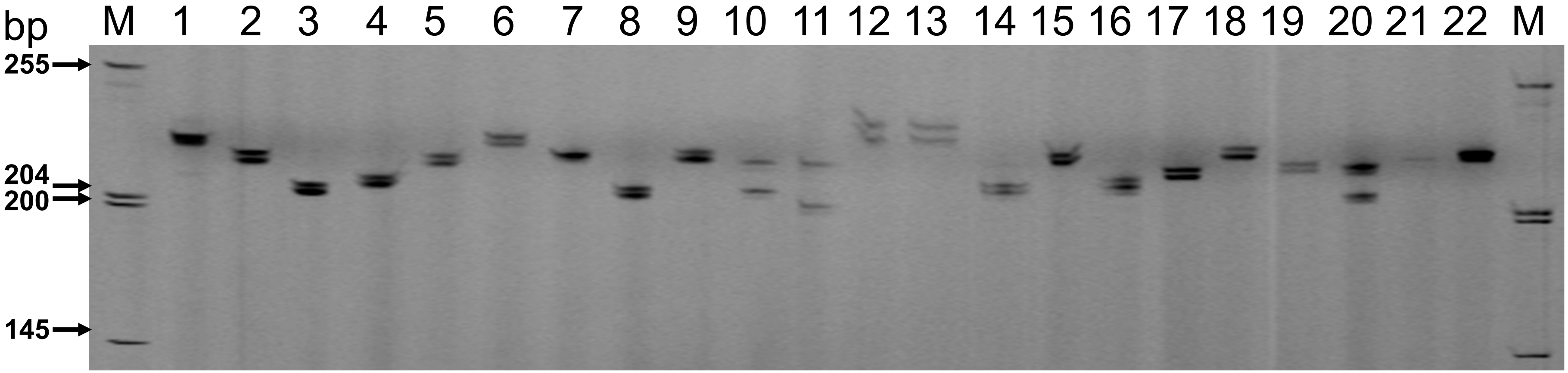

(I) Locus Pap-4356

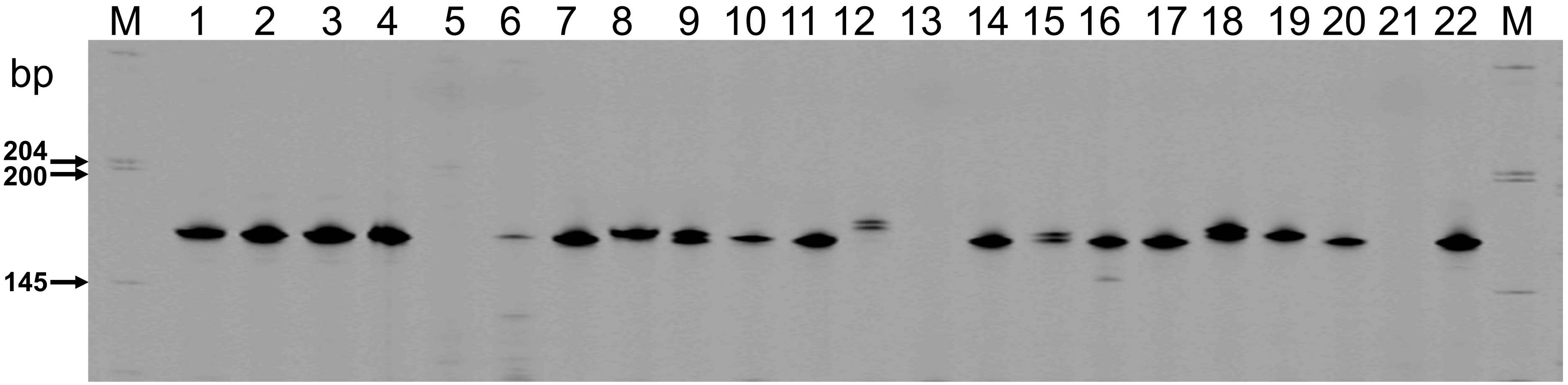

(J) Locus Pap-4825

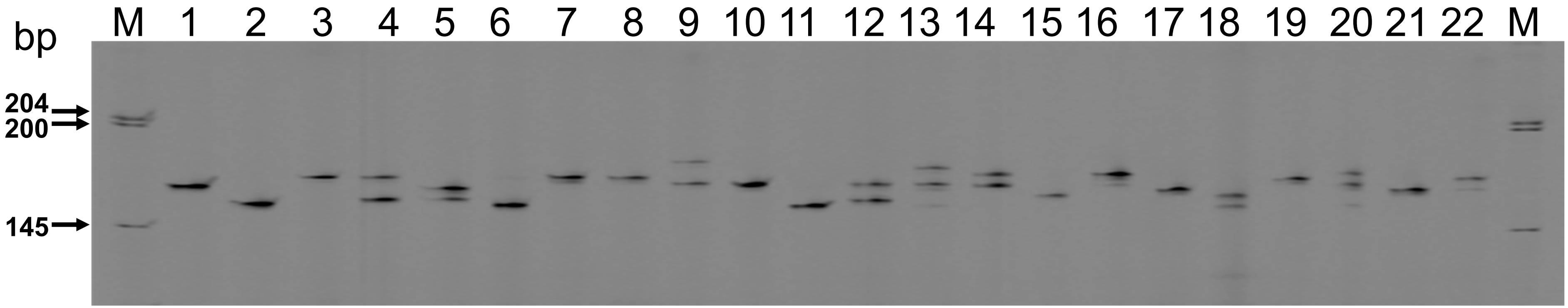

Supplement: S1 Fig — Lanes 1~22 represent 22 Phalaenopsis species listed in Table 4. (PDF) [file pone.0141761.s001.pdf]
