# Supplementary material for: RNA-Seq SSRs of Moth Orchid and Screening for Molecular Markers across Genus Phalaenopsis (Orchidaceae)
Source: PLoS One. 2015 Nov 2;10(11):e0141761. doi: 10.1371/journal.pone.0141761 (PMC4629892; doi:10.1371/journal.pone.0141761)
Supplement: S1 Table — (PDF) [file pone.0141761.s002.pdf]

S1 Table. The information of EST-SSR primers of the study.

(A) Dinucleotide repeat more than 9 times.

Seq ID	Orientation	tm	GC%	Seq	Prod size	Seq len	Included l	Motif	SSR	SSRLen
Locus_60_Transcript_55/59_Confidence_0.345_Length_3395	FORWARD	54.64	38.1	TTCTATGTTTGTGGTGAGGAT	149	420	420	CA	CACACACACACACACACACA	20
Locus_60_Transcript_55/59_Confidence_0.345_Length_3395	REVERSE	54.91	33.33	AAGTATCAAATTGCCTGATGA						
Locus_62_Transcript_8/14_Confidence_0.609_Length_2013	FORWARD	55.01	38.1	CTATAGCTTCCAGCATTTGAA	162	420	420	AT	ATATATATATATATATATAT	20
Locus_62_Transcript_8/14_Confidence_0.609_Length_2013	REVERSE	54.56	38.1	GACATAAGCACTCAAAATCGT						
Locus_91_Transcript_7/37_Confidence_0.514_Length_1796	FORWARD	55.29	45	ACCGGATATCGGTTAGTTCT	149	419	419	TC	TCTCTCTCTCTCTCTCTC	18
Locus_91_Transcript_7/37_Confidence_0.514_Length_1796	REVERSE	54.34	31.82	AGCACAAATACTTGAATTCTCA						
Locus_92_Transcript_52/102_Confidence_0.511_Length_3385	FORWARD	54.78	42.86	TGATGAGAAGAAGAGACTTGC	159	422	422	AG	AGAGAGAGAGAGAGAGAGAGAG	22
Locus_92_Transcript_52/102_Confidence_0.511_Length_3385	REVERSE	54.67	42.86	GCTTCAGTATCTCTCCAAACA						
Locus_92_Transcript_52/102_Confidence_0.511_Length_3385	FORWARD	55.41	42.86	CGAGGGGGATTTGTATAATAG	145	422	422	CT	CTCTCTCTCTCTCTCTCTCTCT	22
Locus_92_Transcript_52/102_Confidence_0.511_Length_3385	REVERSE	55.24	52.38	AAGTGGTGGAGAGAGAGAGAG						
Locus_113_Transcript_53/68_Confidence_0.525_Length_2354	FORWARD	56.13	42.86	GCCCGACAATATTTAGAGAGA	140	423	423	GA	GAGAGAGAGAGAGAGAGAGAGA	22
Locus_113_Transcript_53/68_Confidence_0.525_Length_2354	REVERSE	54.75	33.33	ATCAAACAAATTAAGCACAGC						
Locus_113_Transcript_53/68_Confidence_0.525_Length_2354	FORWARD	54.77	52.38	AGGCTTGAGAGAGAGAGAGAG	139	423	423	AG	AGAGAGAGAGAGAGAGAGAGAG	22
Locus_113_Transcript_53/68_Confidence_0.525_Length_2354	REVERSE	54	38.1	CATCGTGAACCCTAATTAAAC						
Locus_136_Transcript_15/18_Confidence_0.509_Length_1077	FORWARD	54.75	28.57	GAGAAATTGCGATTCATTTTA	152	424	424	AG	AGAGAGAGAGAGAGAGAG	18
Locus_136_Transcript_15/18_Confidence_0.509_Length_1077	REVERSE	55	47.62	CGCCTATTTATCTCCTCTCTC						
Locus_136_Transcript_15/18_Confidence_0.509_Length_1077	FORWARD	54.63	33.33	GCGATTCATTTTAAGGACATA	154	424	424	AG	AGAGAGAGAGAGAGAGAGAGAG	22
Locus_136_Transcript_15/18_Confidence_0.509_Length_1077	REVERSE	55.31	38.1	GCCAAATCTACGCCTATTTAT						
Locus_139_Transcript_63/70_Confidence_0.359_Length_1053	FORWARD	54.95	50	CGAGAAAGTCCCATACTGAG	202	421	421	TC	TCTCTCTCTCTCTCTCTCTC	20
Locus_139_Transcript_63/70_Confidence_0.359_Length_1053	REVERSE	55.2	42.86	ATAGGGGATTGGTATCAGAGA						
Locus_139_Transcript_63/70_Confidence_0.359_Length_1053	FORWARD	55.66	52.38	CGCTCATCTCTCTCTCTCTCT	105	421	421	CT	CTCTCTCTCTCTCTCTCTCTCTCT	24
Locus_139_Transcript_63/70_Confidence_0.359_Length_1053	REVERSE	54.83	38.1	AAAAGATGGTATAGGGGATTG						
Locus_165_Transcript_47/93_Confidence_0.268_Length_2277	FORWARD	55.29	40	ACGATGAAGAAACCAAACTG	126	421	421	TC	TCTCTCTCTCTCTCTCTCTC	20
Locus_165_Transcript_47/93_Confidence_0.268_Length_2277	REVERSE	54.83	38.1	AAAAGATGGTATAGGGGATTG						
Locus_190_Transcript_53/63_Confidence_0.522_Length_5794	FORWARD	55.39	47.62	GAGGATCTGGAGTGGATTAAG	171	424	424	AG	AGAGAGAGAGAGAGAGAGAGAGAG	24
Locus_190_Transcript_53/63_Confidence_0.522_Length_5794	REVERSE	55.39	50	TCTCTCTCTCTCTCTTGTGACC						
Locus_190_Transcript_53/63_Confidence_0.522_Length_5794	FORWARD	54.77	52.17	AGAGAGAGAGAGAGAGAGAGAGC	150	424	424	AG	AGAGAGAGAGAGAGAGAG	18
Locus_190_Transcript_53/63_Confidence_0.522_Length_5794	REVERSE	54.84	38.1	AATACCAAAGAGGTCAAATCC						
Locus_190_Transcript_53/63_Confidence_0.522_Length_5794	FORWARD	54.67	33.33	GAAAAGAGAAAATCGTTGACA	162	424	424	AG	AGAGAGAGAGAGAGAGAGAGAG	22
Locus_190_Transcript_53/63_Confidence_0.522_Length_5794	REVERSE	55.35	38.1	GAGTTCTGACGATTCATTTCA						
Locus_190_Transcript_53/63_Confidence_0.522_Length_5794	FORWARD	54.67	33.33	GAAAAGAGAAAATCGTTGACA	168	422	422	AG	AGAGAGAGAGAGAGAGAGAGAG	22
Locus_190_Transcript_53/63_Confidence_0.522_Length_5794	REVERSE	55	42.86	CTTTGTGAGTTCTGACGATTC						
Locus_194_Transcript_42/46_Confidence_0.556_Length_2713	FORWARD	55.11	42.86	CAGTATTGGTCATCAGGGTTA	149	421	421	CT	CTCTCTCTCTCTCTCTCTCT	20
Locus_194_Transcript_42/46_Confidence_0.556_Length_2713	REVERSE	55.07	36.36	TGTATCAGCAAGCTTATTATCG						
Locus_220_Transcript_25/51_Confidence_0.552_Length_2237	FORWARD	55.15	42.86	AGATCTGATCGAGGTTAAAGG	153	418	418	AG	AGAGAGAGAGAGAGAGAG	18
Locus_220_Transcript_25/51_Confidence_0.552_Length_2237	REVERSE	55.5	47.62	TGCCTTTTCTCTCTCCTCTAC						
Locus_288_Transcript_13/24_Confidence_0.527_Length_2899	FORWARD	55.2	38.1	GCAGTTGAATGATGAGATTGT	150	418	418	GA	GAGAGAGAGAGAGAGAGA	18
Locus_288_Transcript_13/24_Confidence_0.527_Length_2899	REVERSE	54.55	33.33	TGTAAATGCCCCTTATATTTG						
Locus_314_Transcript_38/53_Confidence_0.440_Length_3188	FORWARD	54.93	47.62	TTCTCTCCCTCGTTCTCTACT	148	426	426	TC	TCTCTCTCTCTCTCTCTCTCTCTCTC	26
Locus_314_Transcript_38/53_Confidence_0.440_Length_3188	REVERSE	55.09	42.86	AGATGGTATAGGGGATTGGTA						
Locus_329_Transcript_38/56_Confidence_0.520_Length_2789	FORWARD	54.97	38.1	ATGCCTTGAAGGGTAATAATC	142	428	428	AC	ACACACACACACACACACACACACACAC	28
Locus_329_Transcript_38/56_Confidence_0.520_Length_2789	REVERSE	55.44	50	ACTTCATGCAGAGGACTGAG						

Locus_336_Transcript_10/11_Confidence_0.565_Length_774	FORWARD	55.53	52.38	GTGGTAGTATACGAGGCTTCC	152	421	421	TC	TCTCTCTCTCTCTCTCTCTC	20
Locus_336_Transcript_10/11_Confidence_0.565_Length_774	REVERSE	55.33	33.33	TATCGACCTAAAAGCACAAAA						
Locus_346_Transcript_190/203_Confidence_0.165_Length_3056	FORWARD	54.81	40	AGCTTATGAGCAAGAATTGG	155	424	424	GA	GAGAGAGAGAGAGAGAGAGAGAGA	24
Locus_346_Transcript_190/203_Confidence_0.165_Length_3056	REVERSE	54.59	42.86	TCTGTACGTGTTTCTCTTTCC						
Locus_347_Transcript_16/29_Confidence_0.280_Length_4723	FORWARD	54.89	45	GATTGCTCTGGAACAACTTC	147	423	423	TC	TCTCTCTCTCTCTCTCTCTCTC	22
Locus_347_Transcript_16/29_Confidence_0.280_Length_4723	REVERSE	55.1	42.86	AGATTGAAGGATTCTGGAGAG						
Locus_380_Transcript_80/90_Confidence_0.471_Length_3431	FORWARD	55.47	38.1	CATCAGAAGTTGTGCTTCAAT	146	424	424	GA	GAGAGAGAGAGAGAGAGAGAGAGA	24
Locus_380_Transcript_80/90_Confidence_0.471_Length_3431	REVERSE	55.36	57.14	CGCTCTCTCTCTCTCTCTCTC						
Locus_380_Transcript_80/90_Confidence_0.471_Length_3431	FORWARD	55.18	50	TGGAGAGAGAGAGAGAGAGAGA	150	424	424	AG	AGAGAGAGAGAGAGAGAGAGAGAG	24
Locus_380_Transcript_80/90_Confidence_0.471_Length_3431	REVERSE	54.45	42.86	AGTGAATACTGCTTCCACAAC						
Locus_453_Transcript_95/260_Confidence_0.220_Length_2392	FORWARD	54.5	38.1	TGTATTGCATTGTGACTCATC	134	418	418	TG	TGTGTGTGTGTGTGTGTG	18
Locus_453_Transcript_95/260_Confidence_0.220_Length_2392	REVERSE	55.9	27.27	TGAATCTATTTCAAATGCATGA						
Locus_454_Transcript_27/50_Confidence_0.278_Length_2439	FORWARD	55.62	33.33	AAAATGCCTTCAATGAACTCT	150	423	423	TC	TCTCTCTCTCTCTCTCTCTCTC	22
Locus_454_Transcript_27/50_Confidence_0.278_Length_2439	REVERSE	54.83	38.1	TGTTGAAGATTGAGAACCAGT						
Locus_468_Transcript_159/168_Confidence_0.420_Length_3850	FORWARD	55.03	57.14	CTCTCCCTCTCTCTCTCTCTG	166	424	424	CT	CTCTCTCTCTCTCTCTCTCTCTCT	24
Locus_468_Transcript_159/168_Confidence_0.420_Length_3850	REVERSE	54.97	42.86	GCAACTTACTGATTGATGGAG						
Locus_566_Transcript_10/17_Confidence_0.658_Length_6711	FORWARD	55.69	47.62	GTACCAAGAGCTCCAAAACTC	150	420	420	CT	CTCTCTCTCTCTCTCTCTCT	20
Locus_566_Transcript_10/17_Confidence_0.658_Length_6711	REVERSE	55.48	38.1	TTACAGGATTTGCAGAAGCTA						
Locus_588_Transcript_139/151_Confidence_0.216_Length_2310	FORWARD	55.31	42.86	AAACCTGAAACCTTAGAGACG	157	423	423	TC	TCTCTCTCTCTCTCTCTCTCTC	22
Locus_588_Transcript_139/151_Confidence_0.216_Length_2310	REVERSE	54.94	52.38	AGAAGAGCGAGAGAGAGAGAG						
Locus_597_Transcript_640/643_Confidence_0.075_Length_3808	FORWARD	55.12	42.86	CTTTATAGAACGGGTTGGAGT	153	421	421	GA	GAGAGAGAGAGAGAGAGAGA	20
Locus_597_Transcript_640/643_Confidence_0.075_Length_3808	REVERSE	55.36	42.86	TAAGGTGGAGGAAAAAGAGAG						
Locus_623_Transcript_90/105_Confidence_0.396_Length_2799	FORWARD	55	33.33	GAACATGATTTGAATTGGGTA	151	423	423	GA	GAGAGAGAGAGAGAGAGAGAGA	22
Locus_623_Transcript_90/105_Confidence_0.396_Length_2799	REVERSE	55.36	57.14	CGCTCTCTCTCTCTCTCTCTC						
Locus_623_Transcript_90/105_Confidence_0.396_Length_2799	FORWARD	55.13	47.62	TCTCTGTGTGTGTGTGAGAGA	151	423	423	AG	AGAGAGAGAGAGAGAGAGAGAGAG	24
Locus_623_Transcript_90/105_Confidence_0.396_Length_2799	REVERSE	54.67	33.33	TGTCAACGATTTTCTCTTTTC						
Locus_623_Transcript_90/105_Confidence_0.396_Length_2799	FORWARD	55.13	47.62	TGTGTGTGTGTGAGAGAGAGA	200	423	423	AG	AGAGAGAGAGAGAGAGAGAGAG	22
Locus_623_Transcript_90/105_Confidence_0.396_Length_2799	REVERSE	55.31	38.1	GCCAAATCTACGCCTATTTAT						
Locus_623_Transcript_90/105_Confidence_0.396_Length_2799	FORWARD	55.13	47.62	TCTCTGTGTGTGTGTGAGAGA	152	423	423	AG	AGAGAGAGAGAGAGAGAG	18
Locus_623_Transcript_90/105_Confidence_0.396_Length_2799	REVERSE	53.88	33.33	CTGTCAACGATTTTCTCTTTT						
Locus_694_Transcript_18/125_Confidence_0.251_Length_1340	FORWARD	55.3	42.86	GCTTCTATCGTTGATTCCTCT	180	418	418	TC	TCTCTCTCTCTCTCTCTC	18
Locus_694_Transcript_18/125_Confidence_0.251_Length_1340	REVERSE	54.14	38.1	CAGCTTGTAACCTTTTTAGGA						
Locus_694_Transcript_18/125_Confidence_0.251_Length_1340	FORWARD	55.02	57.14	CCGTCTCTCTCTCTCTCTCTC	149	418	418	CT	CTCTCTCTCTCTCTCTCTCTCTCT	24
Locus_694_Transcript_18/125_Confidence_0.251_Length_1340	REVERSE	54.83	38.1	AAAAGATGGTATAGGGGATTG						
Locus_694_Transcript_18/125_Confidence_0.251_Length_1340	FORWARD	54.61	45	CTATCGTTGATTCCTCTGCT	176	424	424	TC	TCTCTCTCTCTCTCTCTC	18
Locus_694_Transcript_18/125_Confidence_0.251_Length_1340	REVERSE	54.14	38.1	CAGCTTGTAACCTTTTTAGGA						
Locus_700_Transcript_59/63_Confidence_0.286_Length_2042	FORWARD	55.28	33.33	TGACATTTTCAACAACTTTCC	150	419	419	GA	GAGAGAGAGAGAGAGAGA	18
Locus_700_Transcript_59/63_Confidence_0.286_Length_2042	REVERSE	54.31	40.91	CTGTTCCTGATCAGTATGTGTT						
Locus_721_Transcript_7/28_Confidence_0.618_Length_1364	FORWARD	54.62	33.33	GGAGCAAAAAGGTTTAGTTTT	134	419	419	TA	TATATATATATATATATA	18
Locus_721_Transcript_7/28_Confidence_0.618_Length_1364	REVERSE	55.04	28.57	TTTGCAAATAATCCCATCTAA						
Locus_725_Transcript_67/131_Confidence_0.450_Length_4072	FORWARD	54.48	42.86	GAGAGGAGCTGGTTTTCTATT	142	424	424	AG	AGAGAGAGAGAGAGAGAGAGAGAG	24
Locus_725_Transcript_67/131_Confidence_0.450_Length_4072	REVERSE	55	47.62	CGCCTATTTATCTCCTCTCTC						
Locus_725_Transcript_67/131_Confidence_0.450_Length_4072	FORWARD	54.67	33.33	GAAAAGAGAAAATCGTTGACA	143	424	424	AG	AGAGAGAGAGAGAGAGAGAGAG	22
Locus_725_Transcript_67/131_Confidence_0.450_Length_4072	REVERSE	54.8	42.86	GTAAAATAGCACGAGACCGTA						
Locus_743_Transcript_41/52_Confidence_0.489_Length_1468	FORWARD	55.37	33.33	TGATCGTAAATAAGGGGATTT	164	419	419	TC	TCTCTCTCTCTCTCTCTC	18

Locus_743_Transcript_41/52_Confidence_0.489_Length_1468	REVERSE	54.87	42.86	ACTATTGGGTAGCGACTCTTT						
Locus_743_Transcript_41/52_Confidence_0.489_Length_1468	FORWARD	52.71	22.73	AATTTTTATAATTGCACCTTGT	145	424	424	TC	TCTCTCTCTCTCTCTCTC	18
Locus_743_Transcript_41/52_Confidence_0.489_Length_1468	REVERSE	54.6	47.62	GTAGTTGTTTGTGGAGACACC						
Locus_743_Transcript_41/52_Confidence_0.489_Length_1468	FORWARD	54.6	47.62	GGTGTCTCCACAAACAACTAC	151	424	424	CT	CTCTCTCTCTCTCTCTCTCTCTCT	24
Locus_743_Transcript_41/52_Confidence_0.489_Length_1468	REVERSE	55.09	42.86	AGATGGTATAGGGGATTGGTA						
Locus_771_Transcript_43/152_Confidence_0.329_Length_1559	FORWARD	54.82	40	GCAAAGCATCTTCTTCAAGT	136	423	423	TC	TCTCTCTCTCTCTCTCTCTCTC	22
Locus_771_Transcript_43/152_Confidence_0.329_Length_1559	REVERSE	55.01	42.86	ACCTCGTATGAATGAGTAGCA						
Locus_827_Transcript_9/19_Confidence_0.613_Length_1562	FORWARD	55.62	42.86	GTTGAGATGGTAAGCCTTTTC	159	423	423	TA	TATATATATATATATATATATA	22
Locus_827_Transcript_9/19_Confidence_0.613_Length_1562	REVERSE	55.08	38.1	TGGCAGGAGAATTCAGTATAA						
Locus_862_Transcript_66/186_Confidence_0.287_Length_3509	FORWARD	56.33	42.86	GCAGGAGAAGAAAGATTCTGA	141	424	424	CT	CTCTCTCTCTCTCTCTCTCTCTCT	24
Locus_862_Transcript_66/186_Confidence_0.287_Length_3509	REVERSE	54.83	38.1	AAAAGATGGTATAGGGGATTG						
Locus_862_Transcript_66/186_Confidence_0.287_Length_3509	FORWARD	55.54	38.1	AATTCTGAAACGAGGACAACT	159	424	424	CT	CTCTCTCTCTCTCTCTCTCTCTCT	24
Locus_862_Transcript_66/186_Confidence_0.287_Length_3509	REVERSE	55.5	47.62	GAGAGAGGAGCTGGTTTTCTA						
Locus_871_Transcript_10/52_Confidence_0.508_Length_3304	FORWARD	53.5	42.86	GGGTGTACGAAATCCTAGATA	161	425	425	AG	AGAGAGAGAGAGAGAGAGAGAGAG	24
Locus_871_Transcript_10/52_Confidence_0.508_Length_3304	REVERSE	54.25	38.1	CTGTCAACGATTTTCTCTTTC						
Locus_871_Transcript_10/52_Confidence_0.508_Length_3304	FORWARD	54.25	38.1	GAAAGAGAAAATCGTTGACAG	188	424	424	GA	GAGAGAGAGAGAGAGAGAGAGAGA	24
Locus_871_Transcript_10/52_Confidence_0.508_Length_3304	REVERSE	54.62	52.17	CTCCTCTCTCTCTCTCTCTCTCT						
Locus_871_Transcript_10/52_Confidence_0.508_Length_3304	FORWARD	53.5	42.86	GGGTGTACGAAATCCTAGATA	139	422	422	GA	GAGAGAGAGAGAGAGAGAGAGAGA	24
Locus_871_Transcript_10/52_Confidence_0.508_Length_3304	REVERSE	55.36	57.14	CGCTCTCTCTCTCTCTCTCTC						
Locus_871_Transcript_10/52_Confidence_0.508_Length_3304	FORWARD	53.75	36.36	AGATATATAACGGGAAGAAAGC	152	422	422	AG	AGAGAGAGAGAGAGAGAGAGAGAG	24
Locus_871_Transcript_10/52_Confidence_0.508_Length_3304	REVERSE	55.39	50	TCTCTCTCTCTCTCTTGTGACC						
Locus_871_Transcript_10/52_Confidence_0.508_Length_3304	FORWARD	54.62	52.17	AGAGAGAGAGAGAGAGAGGAGAG	165	425	425	AG	AGAGAGAGAGAGAGAGAGAGAGAG	24
Locus_871_Transcript_10/52_Confidence_0.508_Length_3304	REVERSE	55.01	33.33	TTCCCGTTATATATCTAGGATTTC						
Locus_871_Transcript_10/52_Confidence_0.508_Length_3304	FORWARD	54.67	33.33	GAAAAGAGAAAATCGTTGACA	147	419	419	AG	AGAGAGAGAGAGAGAGAGAGAG	22
Locus_871_Transcript_10/52_Confidence_0.508_Length_3304	REVERSE	55.42	42.86	GGAGAAGGAAAACAGATCAAC						
Locus_894_Transcript_36/57_Confidence_0.457_Length_2644	FORWARD	55.3	42.86	AACAAAACCTAGAGCCACTTC	150	421	421	CT	CTCTCTCTCTCTCTCTCTCTCTCT	24
Locus_894_Transcript_36/57_Confidence_0.457_Length_2644	REVERSE	54.09	50	GAGGAATGAGAGAGAGAGAGAG						
Locus_894_Transcript_36/57_Confidence_0.457_Length_2644	FORWARD	54.19	57.14	GCCTCTCTCTCTCTCTCTCTC	173	421	421	CT	CTCTCTCTCTCTCTCTCTCTCT	22
Locus_894_Transcript_36/57_Confidence_0.457_Length_2644	REVERSE	55.09	47.62	GGTATAGGGGATTGGTATCAG						
Locus_894_Transcript_36/57_Confidence_0.457_Length_2644	FORWARD	54.48	50	TCTGCTCTCTCTCTCTCTCTCT	150	421	421	CT	CTCTCTCTCTCTCTCTCTCTCT	22
Locus_894_Transcript_36/57_Confidence_0.457_Length_2644	REVERSE	55.09	47.62	GGTATAGGGGATTGGTATCAG						
Locus_894_Transcript_36/57_Confidence_0.457_Length_2644	FORWARD	54.48	50	TGCTCTCTCTCTCTCTCTCTCT	152	424	424	CT	CTCTCTCTCTCTCTCTCTCTCTCT	24
Locus_894_Transcript_36/57_Confidence_0.457_Length_2644	REVERSE	55.09	42.86	AGATGGTATAGGGGATTGGTA						
Locus_894_Transcript_36/57_Confidence_0.457_Length_2644	FORWARD	54.92	50	TCTCTCTCTCTCTCTCTCTTGC	154	423	423	CT	CTCTCTCTCTCTCTCTCTCTCTCT	24
Locus_894_Transcript_36/57_Confidence_0.457_Length_2644	REVERSE	55.04	33.33	GAGTCCAAATTTCACCTTTTT						
Locus_894_Transcript_36/57_Confidence_0.457_Length_2644	FORWARD	54.78	52.38	CCTTCCATCTCTCTCTCTCTC	228	424	424	CT	CTCTCTCTCTCTCTCTCTCTCTCT	24
Locus_894_Transcript_36/57_Confidence_0.457_Length_2644	REVERSE	55.09	47.62	GGTATAGGGGATTGGTATCAG						
Locus_977_Transcript_43/65_Confidence_0.481_Length_2237	FORWARD	54.99	33.33	ACTCAAAATTGTCCTGCAATA	156	424	424	CT	CTCTCTCTCTCTCTCTCTCTCTCT	24
Locus_977_Transcript_43/65_Confidence_0.481_Length_2237	REVERSE	55.24	47.62	GGAAGACGAAGAAGAGAGTGT						
Locus_977_Transcript_43/65_Confidence_0.481_Length_2237	FORWARD	54.97	52.38	ATCCACCACTCTCTCTCTCTC	158	424	424	CT	CTCTCTCTCTCTCTCTCTCTCTCT	24
Locus_977_Transcript_43/65_Confidence_0.481_Length_2237	REVERSE	55.06	38.1	GTGATTGGTGGTAATTGAAGA						
Locus_999_Transcript_23/24_Confidence_0.606_Length_905	FORWARD	53.96	28.57	GAAAGCAAATTATTGAAGGAA	129	423	423	GA	GAGAGAGAGAGAGAGAGAGAGA	22
Locus_999_Transcript_23/24_Confidence_0.606_Length_905	REVERSE	54.67	33.33	TGTCAACGATTTTCTCTTTTC						
Locus_999_Transcript_23/24_Confidence_0.606_Length_905	FORWARD	53.96	28.57	GAAAGCAAATTATTGAAGGAA	176	423	423	AG	AGAGAGAGAGAGAGAGAG	18
Locus_999_Transcript_23/24_Confidence_0.606_Length_905	REVERSE	55.59	52.38	CCATCTCTGTCTCTTCTCCTC						

Locus_999_Transcript_23/24_Confidence_0.606_Length_905	FORWARD	55.14	55	CGGTCACAAGAGAGAGAGAG	141	423	423	AG	AGAGAGAGAGAGAGAGAGAGAG	22
Locus_999_Transcript_23/24_Confidence_0.606_Length_905	REVERSE	55.55	47.62	TGCTGCTACTGGAGAAGTCTA						
Locus_1000_Transcript_8/39_Confidence_0.605_Length_2175	FORWARD	55.22	33.33	TATATTTAAACGCTCCGATCA	145	425	425	GA	GAGAGAGAGAGAGAGAGAGAGAGA	24
Locus_1000_Transcript_8/39_Confidence_0.605_Length_2175	REVERSE	55	47.62	CGCCTATTTATCTCCTCTCTC						
Locus_1000_Transcript_8/39_Confidence_0.605_Length_2175	FORWARD	55.02	57.14	GGTCGAGAGAGAGAGAGAGAG	150	425	425	AG	AGAGAGAGAGAGAGAGAG	18
Locus_1000_Transcript_8/39_Confidence_0.605_Length_2175	REVERSE	54.84	38.1	AATACCAAAGAGGTCAAATCC						
Locus_1003_Transcript_18/20_Confidence_0.642_Length_2519	FORWARD	55.72	42.86	TGTAACCTTCTTGAGCTTGTG	152	418	418	TC	TCTCTCTCTCTCTCTCTC	18
Locus_1003_Transcript_18/20_Confidence_0.642_Length_2519	REVERSE	55.34	42.86	ACAGAGGCCATAGATTTTAGC						
Locus_1025_Transcript_34/46_Confidence_0.464_Length_1343	FORWARD	55.34	47.62	GGTGCCAGTACTATTCTGTGA	140	424	424	CT	CTCTCTCTCTCTCTCTCTCTCTCT	24
Locus_1025_Transcript_34/46_Confidence_0.464_Length_1343	REVERSE	54.83	38.1	AAAAGATGGTATAGGGGATTG						
Locus_1030_Transcript_29/64_Confidence_0.335_Length_2552	FORWARD	54.29	33.33	CTTTCATTTCGAAAGTTGTTC	144	422	422	CT	CTCTCTCTCTCTCTCTCTCTCT	22
Locus_1030_Transcript_29/64_Confidence_0.335_Length_2552	REVERSE	54.75	42.86	TATCGTTCAGAATGAGAGAGC						
Locus_1069_Transcript_41/64_Confidence_0.406_Length_2993	FORWARD	55.93	52.38	GCAGATGAGAGAGAGAGAAGG	150	423	423	TC	TCTCTCTCTCTCTCTCTCTCTC	22
Locus_1069_Transcript_41/64_Confidence_0.406_Length_2993	REVERSE	54.76	33.33	TTGCCTAGAGAAAGCAAATTA						
Locus_1100_Transcript_24/51_Confidence_0.515_Length_1753	FORWARD	55.08	38.1	TGACACAACGAAGAAGAAGTT	142	420	420	AG	AGAGAGAGAGAGAGAGAGAG	20
Locus_1100_Transcript_24/51_Confidence_0.515_Length_1753	REVERSE	55	42.86	TAAAGGCTGGACCTTTAGAGT						
Locus_1139_Transcript_15/20_Confidence_0.702_Length_3598	FORWARD	54.89	42.86	CAGTCACAGATCGTTAAAACC	183	419	419	CT	CTCTCTCTCTCTCTCTCT	18
Locus_1139_Transcript_15/20_Confidence_0.702_Length_3598	REVERSE	54.69	47.62	GCCGTTAGAGAGAGAGAAAGT						
Locus_1156_Transcript_76/96_Confidence_0.242_Length_1523	FORWARD	54.94	47.62	GTACAGTTGCAGAAACCAGAC	153	424	424	AG	AGAGAGAGAGAGAGAGAGAGAGAG	24
Locus_1156_Transcript_76/96_Confidence_0.242_Length_1523	REVERSE	55.36	57.14	CGCTCTCTCTCTCTCTCTCTC						
Locus_1156_Transcript_76/96_Confidence_0.242_Length_1523	FORWARD	54.03	57.14	GAGAGGAGAGAGGAGAGAGAG	150	424	424	AG	AGAGAGAGAGAGAGAGAGAGAGAG	24
Locus_1156_Transcript_76/96_Confidence_0.242_Length_1523	REVERSE	55.01	33.33	CACCATCACTTTTATTTTTGC						
Locus_1184_Transcript_24/68_Confidence_0.468_Length_2356	FORWARD	54.84	42.86	TGAATAACAGAGAGCGAGTTC	153	425	425	GA	GAGAGAGAGAGAGAGAGAGAGAGA	24
Locus_1184_Transcript_24/68_Confidence_0.468_Length_2356	REVERSE	55.43	45	CTTCATCATCCCCATACAAG						
Locus_1184_Transcript_24/68_Confidence_0.468_Length_2356	FORWARD	54.84	42.86	TGAATAACAGAGAGCGAGTTC	153	421	421	GA	GAGAGAGAGAGAGAGAGAGAGAGA	24
Locus_1184_Transcript_24/68_Confidence_0.468_Length_2356	REVERSE	55.43	45	CTTCATCATCCCCATACAAG						
Locus_1210_Transcript_12/25_Confidence_0.653_Length_1225	FORWARD	55.21	33.33	CTTGCTTCTTTTATTTGCTGA	150	418	418	AG	AGAGAGAGAGAGAGAGAG	18
Locus_1210_Transcript_12/25_Confidence_0.653_Length_1225	REVERSE	55.37	42.86	GCTCTTCGATTGTTACCTCTT						
Locus_1239_Transcript_30/64_Confidence_0.378_Length_2745	FORWARD	55.51	45	CCTTCTTCCTCTCCAAAATC	164	419	419	AG	AGAGAGAGAGAGAGAGAG	18
Locus_1239_Transcript_30/64_Confidence_0.378_Length_2745	REVERSE	54.79	45.83	TCTCTTTCTCTCTCTCTCTCTCTC						
Locus_1260_Transcript_25/44_Confidence_0.630_Length_3073	FORWARD	54.58	38.1	ACAGCATCAGTAATTCCAAAG	145	422	422	TA	TATATATATATATATATATATA	22
Locus_1260_Transcript_25/44_Confidence_0.630_Length_3073	REVERSE	55.14	33.33	TTCACATTGTTGCATGATAGA						
Locus_1260_Transcript_25/44_Confidence_0.630_Length_3073	FORWARD	55.64	47.62	CCTTCGAGAAGAAGAGAGAGA	151	422	422	CT	CTCTCTCTCTCTCTCTCTCTCT	22
Locus_1260_Transcript_25/44_Confidence_0.630_Length_3073	REVERSE	54.34	52.38	GAGGAGTACACAGAGGTGAGA						
Locus_1307_Transcript_93/117_Confidence_0.311_Length_2543	FORWARD	54.67	38.1	AGATATCTGAAGAAGGCCAAT	165	421	421	TC	TCTCTCTCTCTCTCTCTCTC	20
Locus_1307_Transcript_93/117_Confidence_0.311_Length_2543	REVERSE	54.46	42.86	TCCTACTTCCTTTCTCTTTCC						
Locus_1359_Transcript_6/47_Confidence_0.486_Length_1386	FORWARD	54.91	40	ACAAAAGCGAAGAGAACTTG	168	420	420	TA	TATATATATATATATATATA	20
Locus_1359_Transcript_6/47_Confidence_0.486_Length_1386	REVERSE	55.18	45	AGTTGGATAGGAAGGGGATA						
Locus_1359_Transcript_6/47_Confidence_0.486_Length_1386	FORWARD	54.91	40	ACAAAAGCGAAGAGAACTTG	194	420	420	TA	TATATATATATATATATATATATA	24
Locus_1359_Transcript_6/47_Confidence_0.486_Length_1386	REVERSE	55.98	35	TTTTTCCGGTTGCTACAATA						
Locus_1516_Transcript_52/110_Confidence_0.531_Length_2879	FORWARD	54.44	38.1	GGAAGGATGTATATTTGCTCA	154	424	424	CT	CTCTCTCTCTCTCTCTCTCTCTCT	24
Locus_1516_Transcript_52/110_Confidence_0.531_Length_2879	REVERSE	54.34	38.1	CAACACTCATCAATTTCTCCT						
Locus_1598_Transcript_49/127_Confidence_0.358_Length_1956	FORWARD	54.33	47.62	ATTCAGGTACCACACTCTACG	161	419	419	AC	ACACACACACACACACAC	18
Locus_1598_Transcript_49/127_Confidence_0.358_Length_1956	REVERSE	54.49	42.86	TCTAAGGATTGAGTGGTATGC						
Locus_1609_Transcript_17/42_Confidence_0.582_Length_820	FORWARD	55.49	42.86	ATCTGTACTGAGATGGGCTTT	148	423	423	GA	GAGAGAGAGAGAGAGAGAGAGA	22

Locus_1609_Transcript_17/42_Confidence_0.582_Length_820	REVERSE	54.48	52.38	TCTCTCTCTCTCTCCTTCTCC						
Locus_1609_Transcript_17/42_Confidence_0.582_Length_820	FORWARD	55.49	42.86	ATCTGTACTGAGATGGGCTTT	191	423	423	GA	GAGAGAGAGAGAGAGAGAGAGA	22
Locus_1609_Transcript_17/42_Confidence_0.582_Length_820	REVERSE	55.36	57.14	CGCTCTCTCTCTCTCTCTCTC						
Locus_1633_Transcript_29/35_Confidence_0.365_Length_2130	FORWARD	55.31	42.86	AATAGCAGGATCAGAAGGAAG	155	425	425	GA	GAGAGAGAGAGAGAGAGAGAGAGA	24
Locus_1633_Transcript_29/35_Confidence_0.365_Length_2130	REVERSE	55.36	57.14	CGCTCTCTCTCTCTCTCTCTC						
Locus_1633_Transcript_29/35_Confidence_0.365_Length_2130	FORWARD	54.03	57.14	GGGAGAGAGAGAGAGAGAGAG	153	425	425	AG	AGAGAGAGAGAGAGAGAG	18
Locus_1633_Transcript_29/35_Confidence_0.365_Length_2130	REVERSE	53.88	33.33	CTGTCAACGATTTTCTCTTTT						
Locus_1633_Transcript_29/35_Confidence_0.365_Length_2130	FORWARD	54.67	33.33	GAAAAGAGAAAATCGTTGACA	155	425	425	AG	AGAGAGAGAGAGAGAGAG	18
Locus_1633_Transcript_29/35_Confidence_0.365_Length_2130	REVERSE	54.8	42.86	ACAAGCAAGAAGATGAGTACG						
Locus_1633_Transcript_29/35_Confidence_0.365_Length_2130	FORWARD	55.02	50	GAGAGAGATTGCCGATAGTG	155	422	422	GA	GAGAGAGAGAGAGAGAGAGAGAGA	24
Locus_1633_Transcript_29/35_Confidence_0.365_Length_2130	REVERSE	55.39	50	TCTCTCTCTCTCTCTTGTGACC						
Locus_1644_Transcript_32/35_Confidence_0.608_Length_3704	FORWARD	55.68	38.1	GATACACATTGAAAGCCATTG	155	424	424	AG	AGAGAGAGAGAGAGAGAGAGAGAG	24
Locus_1644_Transcript_32/35_Confidence_0.608_Length_3704	REVERSE	54.67	33.33	TGTCAACGATTTTCTCTTTTC						
Locus_1644_Transcript_32/35_Confidence_0.608_Length_3704	FORWARD	54.67	33.33	GAAAAGAGAAAATCGTTGACA	146	424	424	AG	AGAGAGAGAGAGAGAGAGAGAG	22
Locus_1644_Transcript_32/35_Confidence_0.608_Length_3704	REVERSE	54.71	47.62	CTTCCCCTAACTTCATCTCTC						
Locus_1644_Transcript_32/35_Confidence_0.608_Length_3704	FORWARD	54.67	33.33	GAAAAGAGAAAATCGTTGACA	146	419	419	AG	AGAGAGAGAGAGAGAGAG	18
Locus_1644_Transcript_32/35_Confidence_0.608_Length_3704	REVERSE	54.71	47.62	CTTCCCCTAACTTCATCTCTC						
Locus_1656_Transcript_56/66_Confidence_0.457_Length_1455	FORWARD	54.34	38.1	AGAGTTCTTGTGAATTGGATG	149	420	420	TC	TCTCTCTCTCTCTCTCTCTC	20
Locus_1656_Transcript_56/66_Confidence_0.457_Length_1455	REVERSE	55.09	47.62	GGTATAGGGGATTGGTATCAG						
Locus_1656_Transcript_56/66_Confidence_0.457_Length_1455	FORWARD	54.34	38.1	AGAGTTCTTGTGAATTGGATG	153	420	420	CT	CTCTCTCTCTCTCTCTCTCTCTCT	24
Locus_1656_Transcript_56/66_Confidence_0.457_Length_1455	REVERSE	55.09	42.86	AGATGGTATAGGGGATTGGTA						
Locus_1675_Transcript_29/41_Confidence_0.505_Length_2360	FORWARD	54.85	28.57	TCAATTTATTGCCTTCATCAT	140	424	424	TA	TATATATATATATATATATATATA	24
Locus_1675_Transcript_29/41_Confidence_0.505_Length_2360	REVERSE	55.23	33.33	AATTCCTCCTCATCATTTGTT						
Locus_1681_Transcript_75/91_Confidence_0.392_Length_2256	FORWARD	54.3	33.33	TTGGGAGTTTAGTTTCTTTCA	155	418	418	AT	ATATATATATATATATAT	18
Locus_1681_Transcript_75/91_Confidence_0.392_Length_2256	REVERSE	54.7	38.1	TCCTAAATGGCTCATACTTTG						
Locus_1783_Transcript_105/138_Confidence_0.339_Length_5150	FORWARD	54.45	28.57	TTAAATGTTAAATGGGGTTTG	159	424	424	AG	AGAGAGAGAGAGAGAGAGAGAGAG	24
Locus_1783_Transcript_105/138_Confidence_0.339_Length_5150	REVERSE	54.03	52.38	TCTCTCTCCCTCTCTCTCTCT						
Locus_1806_Transcript_28/33_Confidence_0.521_Length_1557	FORWARD	55	42.86	AGCCTCTCTGTTTCTCTGTTT	143	421	421	TC	TCTCTCTCTCTCTCTCTCTC	20
Locus_1806_Transcript_28/33_Confidence_0.521_Length_1557	REVERSE	54.29	47.62	ACAGAGTCTCCTTCTTCCTTC						
Locus_1861_Transcript_32/77_Confidence_0.481_Length_2754	FORWARD	54.86	38.1	AGAATCTCAGGCTCTTTTGAT	143	424	424	AG	AGAGAGAGAGAGAGAGAGAGAGAGAGAG	28
Locus_1861_Transcript_32/77_Confidence_0.481_Length_2754	REVERSE	55.36	57.14	CGCTCTCTCTCTCTCTCTCTC						
Locus_1912_Transcript_21/23_Confidence_0.333_Length_536	FORWARD	54.14	38.1	TCCTAAAAAGGTTACAAGCTG	153	424	424	CT	CTCTCTCTCTCTCTCTCTCTCTCT	24
Locus_1912_Transcript_21/23_Confidence_0.333_Length_536	REVERSE	54.77	28.57	TTTCTTCATTGCTTTTGATTC						
Locus_1930_Transcript_47/74_Confidence_0.127_Length_2941	FORWARD	54.64	42.86	CCATTGGTTGATAGAGAACAC	174	421	421	GA	GAGAGAGAGAGAGAGAGAGA	20
Locus_1930_Transcript_47/74_Confidence_0.127_Length_2941	REVERSE	55.37	50	GAGGAGGGTTTCTTCGATAC						
Locus_1956_Transcript_14/26_Confidence_0.583_Length_1507	FORWARD	55.67	47.62	GGTATGCACAGCTTGAGTCTA	178	420	420	CT	CTCTCTCTCTCTCTCTCTCT	20
Locus_1956_Transcript_14/26_Confidence_0.583_Length_1507	REVERSE	54.79	28.57	ACCAAAATCATCAGTTCAAAA						
Locus_1964_Transcript_30/53_Confidence_0.329_Length_1309	FORWARD	54.44	45	GTTTTCAGGTCCATCATCTC	149	418	418	AG	AGAGAGAGAGAGAGAGAG	18
Locus_1964_Transcript_30/53_Confidence_0.329_Length_1309	REVERSE	54.03	57.14	CTCTCTCTCTCTCCTCTCTCC						
Locus_1964_Transcript_30/53_Confidence_0.329_Length_1309	FORWARD	54.94	52.38	AGCGAGAGAGAGAAGAGAGAGAG	149	418	418	GA	GAGAGAGAGAGAGAGAGAGAGA	22
Locus_1964_Transcript_30/53_Confidence_0.329_Length_1309	REVERSE	53.93	33.33	CTCTCTTTCGAAATGGAATAA						
Locus_1991_Transcript_29/47_Confidence_0.347_Length_4472	FORWARD	54.41	38.1	CATAGAAAAGAGAAGCAATGG	150	422	422	TC	TCTCTCTCTCTCTCTCTCTCTC	22
Locus_1991_Transcript_29/47_Confidence_0.347_Length_4472	REVERSE	55.09	42.86	AGATGGTATAGGGGATTGGTA						
Locus_2003_Transcript_7/9_Confidence_0.621_Length_1227	FORWARD	54.51	33.33	TTCTTGTTGCTGATGTTGTTA	150	420	420	TA	TATATATATATATATATATA	20
Locus_2003_Transcript_7/9_Confidence_0.621_Length_1227	REVERSE	55.31	38.1	AATTGTGGGGTGTGTATGTAA						

Locus_2012_Transcript_51/68_Confidence_0.562_Length_2744	FORWARD	55.49	40	AAGCAAAGCTGGCTTATACA	194	420	420	CT	CTCTCTCTCTCTCTCTCTCT	20
Locus_2012_Transcript_51/68_Confidence_0.562_Length_2744	REVERSE	55.68	38.1	GCTCGCAAATTTAACTCTCTT						
Locus_2035_Transcript_4/7_Confidence_0.600_Length_2778	FORWARD	55.76	38.1	GTTGCAATTATTCCACTGTTG	155	424	424	CT	CTCTCTCTCTCTCTCTCTCTCTCT	24
Locus_2035_Transcript_4/7_Confidence_0.600_Length_2778	REVERSE	54.99	47.62	CATGAGAGACTGAAGAAGGTG						
Locus_2036_Transcript_23/34_Confidence_0.563_Length_2233	FORWARD	54.81	38.1	TTTGTCCTTGTACCACTTGTT	151	425	425	AT	ATATATATATATATATATATATAT	24
Locus_2036_Transcript_23/34_Confidence_0.563_Length_2233	REVERSE	54.88	31.82	GGTGGATCAGTTAAATTTTCAT						
Locus_2068_Transcript_23/61_Confidence_0.418_Length_1152	FORWARD	55.07	36.36	GCAAATTATTGAAGGAAGAGAG	162	419	419	GA	GAGAGAGAGAGAGAGAGAGAGA	22
Locus_2068_Transcript_23/61_Confidence_0.418_Length_1152	REVERSE	55.1	33.33	TCCTAGTATTCCAAATGCAAA						
Locus_2068_Transcript_23/61_Confidence_0.418_Length_1152	FORWARD	54.81	42.86	ATTGAAGGAAGAGAGAGGAGA	155	419	419	AG	AGAGAGAGAGAGAGAGAG	18
Locus_2068_Transcript_23/61_Confidence_0.418_Length_1152	REVERSE	55.1	33.33	TCCTAGTATTCCAAATGCAAA						
Locus_2109_Transcript_33/44_Confidence_0.581_Length_3294	FORWARD	55.09	33.33	AATCCAATCCTTATCAAAAGC	150	418	418	CT	CTCTCTCTCTCTCTCTCT	18
Locus_2109_Transcript_33/44_Confidence_0.581_Length_3294	REVERSE	54.88	47.62	CCTGGGAAATTAGAGAGAGAG						
Locus_2109_Transcript_33/44_Confidence_0.581_Length_3294	FORWARD	55.67	38.1	TAATTTCCCAGGAACAATACC	169	418	418	CT	CTCTCTCTCTCTCTCTCT	18
Locus_2109_Transcript_33/44_Confidence_0.581_Length_3294	REVERSE	55.05	38.1	TCTTCTCCCCTCAATTTTATC						
Locus_2116_Transcript_18/31_Confidence_0.597_Length_2082	FORWARD	55.41	28.57	TGCCTTATTAAATGAGCAAAA	151	421	421	CT	CTCTCTCTCTCTCTCTCTCT	20
Locus_2116_Transcript_18/31_Confidence_0.597_Length_2082	REVERSE	55.14	55	CTCTCTGTTCTCCACTCTCG						
Locus_2200_Transcript_12/21_Confidence_0.599_Length_6392	FORWARD	54.93	35	ACATATTCGGCGGTATAAAA	141	418	418	TC	TCTCTCTCTCTCTCTCTC	18
Locus_2200_Transcript_12/21_Confidence_0.599_Length_6392	REVERSE	55.54	42.86	AAGGAATGTAAGAGCGAAGAG						
Locus_2246_Transcript_54/74_Confidence_0.464_Length_9675	FORWARD	55.1	27.27	TGCATTAGTTTGAAATGTGTTT	149	424	424	CT	CTCTCTCTCTCTCTCTCTCTCTCT	24
Locus_2246_Transcript_54/74_Confidence_0.464_Length_9675	REVERSE	55.09	42.86	AGATGGTATAGGGGATTGGTA						
Locus_2263_Transcript_151/275_Confidence_0.095_Length_5794	FORWARD	54.14	38.1	TCCTAAAAAGGTTACAAGCTG	161	424	424	CT	CTCTCTCTCTCTCTCTCTCTCTCT	24
Locus_2263_Transcript_151/275_Confidence_0.095_Length_5794	REVERSE	55.74	33.33	CCCCAAATTACAAATCAAAGT						
Locus_2285_Transcript_1/6_Confidence_0.735_Length_3431	FORWARD	55.18	47.62	GGGAGTACCACCACTAAAATC	151	419	419	GA	GAGAGAGAGAGAGAGAGA	18
Locus_2285_Transcript_1/6_Confidence_0.735_Length_3431	REVERSE	57.51	50	CTTTGTCTCCTGGTTTCAGC						
Locus_2367_Transcript_4/16_Confidence_0.536_Length_3739	FORWARD	54.96	38.1	TCTGTGCTTTTCTTAACCTTG	135	419	419	TC	TCTCTCTCTCTCTCTCTC	18
Locus_2367_Transcript_4/16_Confidence_0.536_Length_3739	REVERSE	54.78	35	ATTTTTGACAGGCTGAATGT						
Locus_2428_Transcript_52/101_Confidence_0.350_Length_2863	FORWARD	55.33	42.86	TCCCGTAGATAGAAAAAGAGG	146	426	426	GT	GTGTGTGTGTGTGTGTGTGTGTGTGT	26
Locus_2428_Transcript_52/101_Confidence_0.350_Length_2863	REVERSE	54.64	47.62	CTATCACTGCTATCTCCTCCA						
Locus_2545_Transcript_28/48_Confidence_0.590_Length_2456	FORWARD	55.15	38.1	TCACTGGATTCAAATCGATAC	153	419	419	AG	AGAGAGAGAGAGAGAGAG	18
Locus_2545_Transcript_28/48_Confidence_0.590_Length_2456	REVERSE	54	33.33	GTTGCTCTGAAAAGGATTTTA						
Locus_2577_Transcript_16/31_Confidence_0.608_Length_3830	FORWARD	55.09	40	CACCATTCGTCACCTAATTT	212	424	424	TC	TCTCTCTCTCTCTCTCTCTCTCTC	24
Locus_2577_Transcript_16/31_Confidence_0.608_Length_3830	REVERSE	56.36	55	CTACTCCTGCACTCAAGCAG						
Locus_2641_Transcript_39/55_Confidence_0.330_Length_3582	FORWARD	55.43	42.86	TACCATCTCGTACATCTTTGC	142	424	424	CT	CTCTCTCTCTCTCTCTCTCTCTCT	24
Locus_2641_Transcript_39/55_Confidence_0.330_Length_3582	REVERSE	54.47	42.86	GATGGTATAGGGGATTGGTAT						
Locus_2641_Transcript_39/55_Confidence_0.330_Length_3582	FORWARD	55.5	47.62	TAGAAAACCAGCTCCTCTCTC	153	424	424	CT	CTCTCTCTCTCTCTCTCTCTCTCT	24
Locus_2641_Transcript_39/55_Confidence_0.330_Length_3582	REVERSE	55.09	42.86	AGATGGTATAGGGGATTGGTA						
Locus_2641_Transcript_39/55_Confidence_0.330_Length_3582	FORWARD	55.01	50	TACAGTCCCTGAATGGAGAC	147	424	424	CT	CTCTCTCTCTCTCTCTCTCTCTCT	24
Locus_2641_Transcript_39/55_Confidence_0.330_Length_3582	REVERSE	55.63	33.33	TGTAAGAGCGAAAGCAAATTA						
Locus_2685_Transcript_3/22_Confidence_0.679_Length_2573	FORWARD	53.11	38.1	ATAAGCAGAGGATTGAGTCAT	155	427	427	TA	TATATATATATATATATATATATA	24
Locus_2685_Transcript_3/22_Confidence_0.679_Length_2573	REVERSE	55.16	42.86	GCAGGTATGTCTGTTTGATGT						
Locus_2685_Transcript_3/22_Confidence_0.679_Length_2573	FORWARD	55.34	42.86	ATCAAACAGACATACCTGCTG	170	427	427	AT	ATATATATATATATATATATATATAT	26
Locus_2685_Transcript_3/22_Confidence_0.679_Length_2573	REVERSE	54.96	38.1	GGCATGTCGTTTATTGATTAG						
Locus_2694_Transcript_7/20_Confidence_0.541_Length_2193	FORWARD	55.09	35	AACTTTGCTTCTCAAATCCA	135	424	424	CT	CTCTCTCTCTCTCTCTCTCTCTCT	24
Locus_2694_Transcript_7/20_Confidence_0.541_Length_2193	REVERSE	54.83	38.1	AAAAGATGGTATAGGGGATTG						
Locus_2694_Transcript_7/20_Confidence_0.541_Length_2193	FORWARD	55.45	52.38	CAGCCTCTCCTACAACTCTCT	143	424	424	CT	CTCTCTCTCTCTCTCTCTCTCTCT	24

Locus_2694_Transcript_7/20_Confidence_0.541_Length_2193	REVERSE	55.5	47.62	GAGAGAGGAGCTGGTTTTCTA						
Locus_2715_Transcript_25/35_Confidence_0.500_Length_4014	FORWARD	55.37	33.33	TTGGAAACAGAAATCTGAATG	179	421	421	AG	AGAGAGAGAGAGAGAGAGAG	20
Locus_2715_Transcript_25/35_Confidence_0.500_Length_4014	REVERSE	54.79	42.86	AGAGGGTGAGGAAGAAGTAAA						
Locus_2725_Transcript_4/12_Confidence_0.599_Length_2797	FORWARD	54.94	42.86	ATATTCCCTATGCTCCCTATG	156	424	424	CT	CTCTCTCTCTCTCTCTCTCTCTCT	24
Locus_2725_Transcript_4/12_Confidence_0.599_Length_2797	REVERSE	55.26	38.1	GAGCAGAAAATGGAAAGAGAT						
Locus_2725_Transcript_4/12_Confidence_0.599_Length_2797	FORWARD	55.26	38.1	ATCTCTTTCCATTTTCTGCTC	150	424	424	CT	CTCTCTCTCTCTCTCTCTCT	20
Locus_2725_Transcript_4/12_Confidence_0.599_Length_2797	REVERSE	55.07	40	ATCAAGCTCTCCAAAGTTCA						
Locus_2755_Transcript_52/65_Confidence_0.583_Length_2972	FORWARD	55.04	42.86	TGACAGGAGAGAGAAATCAGA	151	423	423	CT	CTCTCTCTCTCTCTCTCTCTCT	22
Locus_2755_Transcript_52/65_Confidence_0.583_Length_2972	REVERSE	55.24	28.57	AAATAGAAAGCGAAAGCAAAT						
Locus_2767_Transcript_36/42_Confidence_0.591_Length_3879	FORWARD	54.87	38.1	TCAGCTCCTCCCTTTATTTAT	163	419	419	TA	TATATATATATATATATA	18
Locus_2767_Transcript_36/42_Confidence_0.591_Length_3879	REVERSE	55.58	47.62	GAGGACCTCACTGAATACCAT						
Locus_2837_Transcript_9/73_Confidence_0.422_Length_4896	FORWARD	54.5	33.33	TATACAACGAACAGCAATGAA	154	420	420	AG	AGAGAGAGAGAGAGAGAGAG	20
Locus_2837_Transcript_9/73_Confidence_0.422_Length_4896	REVERSE	54.51	38.1	TATATGCCAACCGTATTTCTC						
Locus_2862_Transcript_47/68_Confidence_0.389_Length_2908	FORWARD	56.32	45	ACCTTTCTCCCGATACAGAA	145	420	420	AG	AGAGAGAGAGAGAGAGAGAG	20
Locus_2862_Transcript_47/68_Confidence_0.389_Length_2908	REVERSE	55	42.86	TTCTCCTTCCTTCTCCTCTTA						
Locus_2888_Transcript_3/41_Confidence_0.517_Length_1679	FORWARD	54.9	31.82	AAGCCATATCTCCAGAAAAATA	145	420	420	CT	CTCTCTCTCTCTCTCTCTCT	20
Locus_2888_Transcript_3/41_Confidence_0.517_Length_1679	REVERSE	55.23	38.1	GCCTTTTGTAAATCCTCTTGT						
Locus_2951_Transcript_8/19_Confidence_0.597_Length_2512	FORWARD	55.48	42.86	TAAGAATTGAGGAAGCTAGGG	143	420	420	GA	GAGAGAGAGAGAGAGAGAGA	20
Locus_2951_Transcript_8/19_Confidence_0.597_Length_2512	REVERSE	54.99	38.1	AAACGCTTAAACCATCTCTCT						
Locus_2979_Transcript_2/13_Confidence_0.689_Length_1583	FORWARD	55.02	57.14	CCGACTCTCTCTCTCTCTCTC	149	419	419	TC	TCTCTCTCTCTCTCTCTC	18
Locus_2979_Transcript_2/13_Confidence_0.689_Length_1583	REVERSE	54.38	38.1	ATTAAATCTTGCTAGCTGCTG						
Locus_2982_Transcript_17/50_Confidence_0.248_Length_1021	FORWARD	54.97	33.33	TTGAATCCAAAGTTGTCCTTA	99	419	419	GA	GAGAGAGAGAGAGAGAGA	18
Locus_2982_Transcript_17/50_Confidence_0.248_Length_1021	REVERSE	56.62	33.33	CAAAGACCAATTTTGAAAAGC						
Locus_2993_Transcript_10/23_Confidence_0.690_Length_1442	FORWARD	54.76	38.1	GAGTTGGAGGGAATTAAAAAG	165	424	424	GA	GAGAGAGAGAGAGAGAGAGAGAGA	24
Locus_2993_Transcript_10/23_Confidence_0.690_Length_1442	REVERSE	55.36	57.14	CGCTCTCTCTCTCTCTCTCTC						
Locus_2993_Transcript_10/23_Confidence_0.690_Length_1442	FORWARD	55.53	47.62	GAGGAGCAAAGCTAGATCACT	179	424	424	AG	AGAGAGAGAGAGAGAGAG	18
Locus_2993_Transcript_10/23_Confidence_0.690_Length_1442	REVERSE	53.88	33.33	CTGTCAACGATTTTCTCTTTT						
Locus_2993_Transcript_10/23_Confidence_0.690_Length_1442	FORWARD	54.67	33.33	GAAAAGAGAAAATCGTTGACA	143	424	424	AG	AGAGAGAGAGAGAGAGAGAGAG	22
Locus_2993_Transcript_10/23_Confidence_0.690_Length_1442	REVERSE	56.01	47.62	CGAACCTCGACCTGTAATACT						
Locus_2993_Transcript_10/23_Confidence_0.690_Length_1442	FORWARD	55.53	47.62	GAGGAGCAAAGCTAGATCACT	155	422	422	AG	AGAGAGAGAGAGAGAGAGAGAGAG	24
Locus_2993_Transcript_10/23_Confidence_0.690_Length_1442	REVERSE	55.39	50	TCTCTCTCTCTCTCTTGTGACC						
Locus_3019_Transcript_9/140_Confidence_0.258_Length_6728	FORWARD	55.26	47.62	GAGAAGGAACGGATAGAGAGA	148	422	422	CT	CTCTCTCTCTCTCTCTCTCTCT	22
Locus_3019_Transcript_9/140_Confidence_0.258_Length_6728	REVERSE	54.61	47.62	CAGAAGCACAGAGAGAAAGAG						
Locus_3066_Transcript_35/44_Confidence_0.511_Length_2504	FORWARD	55.04	47.62	TCTCGTTCTCTCTCTCTCCTT	156	420	420	CT	CTCTCTCTCTCTCTCTCTCT	20
Locus_3066_Transcript_35/44_Confidence_0.511_Length_2504	REVERSE	55.5	47.62	GAGAGAGGAGCTGGTTTTCTA						
Locus_3066_Transcript_35/44_Confidence_0.511_Length_2504	FORWARD	54.85	38.1	CGCTCTTACATTCCTTCAATA	142	424	424	CT	CTCTCTCTCTCTCTCTCTCT	20
Locus_3066_Transcript_35/44_Confidence_0.511_Length_2504	REVERSE	55.19	57.14	GGCAGAGAGAGAGAGAGAGAG						
Locus_3066_Transcript_35/44_Confidence_0.511_Length_2504	FORWARD	55.45	31.82	TCCTTCAATAATTTGCTTTCTC	158	424	424	CT	CTCTCTCTCTCTCTCTCTCTCTCT	24
Locus_3066_Transcript_35/44_Confidence_0.511_Length_2504	REVERSE	58.3	55	AGGGCTGATATGGAGGAGAG						
Locus_3066_Transcript_35/44_Confidence_0.511_Length_2504	FORWARD	54.14	38.1	TCCTAAAAAGGTTACAAGCTG	148	420	420	CT	CTCTCTCTCTCTCTCTCTCTCTCT	24
Locus_3066_Transcript_35/44_Confidence_0.511_Length_2504	REVERSE	55.63	52.38	GCCAGAGAGAGAGAGAGAGAA						
Locus_3066_Transcript_35/44_Confidence_0.511_Length_2504	FORWARD	56	57.14	CCCCTCTCTCTCTCTCTCTCT	135	420	420	CT	CTCTCTCTCTCTCTCTCTCTCTCT	24
Locus_3066_Transcript_35/44_Confidence_0.511_Length_2504	REVERSE	55.03	47.62	AAGTAGAAGGTGTACCCAAGG						
Locus_3129_Transcript_23/61_Confidence_0.364_Length_1390	FORWARD	55.12	33.33	AGCATGAGGCAATTACAAATA	171	421	421	AT	ATATATATATATATATATATATATAT	26
Locus_3129_Transcript_23/61_Confidence_0.364_Length_1390	REVERSE	54.85	42.86	TATCACATATGACCCCTGTTC						

Locus_3129_Transcript_23/61_Confidence_0.364_Length_1390	FORWARD	55.11	33.33	GAGATTGAATTTCATGTTGGA	145	421	421	TA	TATATATATATATATATATA	20
Locus_3129_Transcript_23/61_Confidence_0.364_Length_1390	REVERSE	55.53	33.33	CATTGCACACATTTTATCACA						
Locus_3217_Transcript_10/34_Confidence_0.511_Length_2127	FORWARD	55.23	47.62	TTCTACCGTCTTCTACCTTCC	155	423	423	TC	TCTCTCTCTCTCTCTCTCTCTC	22
Locus_3217_Transcript_10/34_Confidence_0.511_Length_2127	REVERSE	55.01	40.91	AAGGTAGTCCTTGAGCATTAGA						
Locus_3250_Transcript_12/26_Confidence_0.673_Length_2536	FORWARD	54.73	40	CTTACTTCACCTTTCGCAAT	146	422	422	TC	TCTCTCTCTCTCTCTCTCTCTC	22
Locus_3250_Transcript_12/26_Confidence_0.673_Length_2536	REVERSE	55.09	42.86	AGATGGTATAGGGGATTGGTA						
Locus_3278_Transcript_15/23_Confidence_0.595_Length_3630	FORWARD	55.04	42.86	TGGACAGAAGCAACAGATACT	152	418	418	CT	CTCTCTCTCTCTCTCTCT	18
Locus_3278_Transcript_15/23_Confidence_0.595_Length_3630	REVERSE	55.08	38.1	AGTACAATTTTCTTCCCGAAC						
Locus_3323_Transcript_8/85_Confidence_0.539_Length_2616	FORWARD	53.96	28.57	GAAAGCAAATTATTGAAGGAA	151	423	423	GA	GAGAGAGAGAGAGAGAGAGAGA	22
Locus_3323_Transcript_8/85_Confidence_0.539_Length_2616	REVERSE	55.14	52.38	CCGTTAGCTTACTCTCCTCTC						
Locus_3323_Transcript_8/85_Confidence_0.539_Length_2616	FORWARD	54.6	52.38	AGAGAGAGAGGAGAGAACTCG	153	423	423	AG	AGAGAGAGAGAGAGAGAG	18
Locus_3323_Transcript_8/85_Confidence_0.539_Length_2616	REVERSE	55.41	42.86	AACAAGGAGTTACGTCCAGAT						
Locus_3349_Transcript_10/12_Confidence_0.618_Length_1410	FORWARD	54.79	42.86	GCCTGATTGTGTCATAAACTC	135	423	423	TA	TATATATATATATATATATATA	22
Locus_3349_Transcript_10/12_Confidence_0.618_Length_1410	REVERSE	54.3	38.1	TAATCGACAGACAACAAGTGA						
Locus_3366_Transcript_8/31_Confidence_0.577_Length_4065	FORWARD	55.73	33.33	TGATGCTGAAGAATGATTGAT	152	419	419	TA	TATATATATATATATATA	18
Locus_3366_Transcript_8/31_Confidence_0.577_Length_4065	REVERSE	54.91	33.33	TCATCAGGTTCTATGCAAAAT						
Locus_3376_Transcript_101/115_Confidence_0.336_Length_5494	FORWARD	56.01	42.86	GATATGGAGGATGAATGGAAG	150	422	422	GA	GAGAGAGAGAGAGAGAGAGAGA	22
Locus_3376_Transcript_101/115_Confidence_0.336_Length_5494	REVERSE	55.44	52.38	ACACTGTCTCTCCACCTCTCT						
Locus_3376_Transcript_101/115_Confidence_0.336_Length_5494	FORWARD	55.37	47.62	AGAGAGAGAGAAGAGCTCGAA	153	422	422	AG	AGAGAGAGAGAGAGAGAGAGAG	22
Locus_3376_Transcript_101/115_Confidence_0.336_Length_5494	REVERSE	54.98	52.38	CAGGTTTCTCTCTACCTAGCC						
Locus_3377_Transcript_36/83_Confidence_0.389_Length_4097	FORWARD	55.27	42.86	CTAGCTGATTATGCCAAGATG	154	423	423	GA	GAGAGAGAGAGAGAGAGAGAGA	22
Locus_3377_Transcript_36/83_Confidence_0.389_Length_4097	REVERSE	54.67	33.33	TGTCAACGATTTTCTCTTTTC						
Locus_3377_Transcript_36/83_Confidence_0.389_Length_4097	FORWARD	54.48	52.38	GGAGAAGGAGAGAGAGAGAGA	166	423	423	AG	AGAGAGAGAGAGAGAGAGAGAG	22
Locus_3377_Transcript_36/83_Confidence_0.389_Length_4097	REVERSE	55.98	42.86	GGACTTAGGAGGCAATGAATA						
Locus_3377_Transcript_36/83_Confidence_0.389_Length_4097	FORWARD	54.67	33.33	GAAAAGAGAAAATCGTTGACA	169	424	424	AG	AGAGAGAGAGAGAGAGAGAGAG	22
Locus_3377_Transcript_36/83_Confidence_0.389_Length_4097	REVERSE	54.63	42.86	AGCACCAATGAAGACTGTTAG						
Locus_3386_Transcript_60/69_Confidence_0.579_Length_3950	FORWARD	55.06	38.1	CCGTATTAAAATCAACCTCCT	147	424	424	TA	TATATATATATATATATATATATA	24
Locus_3386_Transcript_60/69_Confidence_0.579_Length_3950	REVERSE	54.86	38.1	TGATGAGGAAGAAGAATACCA						
Locus_3429_Transcript_28/38_Confidence_0.582_Length_2249	FORWARD	55.09	42.86	AGATGGTATAGGGGATTGGTA	158	419	419	AG	AGAGAGAGAGAGAGAGAG	18
Locus_3429_Transcript_28/38_Confidence_0.582_Length_2249	REVERSE	54.98	50	ACAGAGGTTTCCTTCTCCTC						
Locus_3493_Transcript_10/41_Confidence_0.314_Length_1674	FORWARD	55.24	47.62	CACAGACAAAGCAGAAGAGTC	165	421	421	GA	GAGAGAGAGAGAGAGAGAGA	20
Locus_3493_Transcript_10/41_Confidence_0.314_Length_1674	REVERSE	54.7	42.86	GCTGTTGCATCTCAATAGTCT						
Locus_3493_Transcript_10/41_Confidence_0.314_Length_1674	FORWARD	55.03	57.14	CTGGAGAGGAGAGAGAGAGAG	140	421	421	GA	GAGAGAGAGAGAGAGAGAGAGAGA	24
Locus_3493_Transcript_10/41_Confidence_0.314_Length_1674	REVERSE	54.7	42.86	AGCTGTTGCATCTCAATAGTC						
Locus_3503_Transcript_23/26_Confidence_0.571_Length_1262	FORWARD	54.9	42.86	CATTTTACAACAGAGGGACAG	227	419	419	GA	GAGAGAGAGAGAGAGAGA	18
Locus_3503_Transcript_23/26_Confidence_0.571_Length_1262	REVERSE	55.84	45	AATGAGCTCGACCACATCTA						
Locus_3514_Transcript_7/31_Confidence_0.588_Length_2145	FORWARD	54.95	50	GTATGGTGTGGAGTTCTCGT	155	424	424	TC	TCTCTCTCTCTCTCTCTCTC	20
Locus_3514_Transcript_7/31_Confidence_0.588_Length_2145	REVERSE	55.2	42.86	ATAGGGGATTGGTATCAGAGA						
Locus_3514_Transcript_7/31_Confidence_0.588_Length_2145	FORWARD	54.5	52.38	GCCATCTCTCTCTCTCTCTCT	162	424	424	CT	CTCTCTCTCTCTCTCTCTCTCTCT	24
Locus_3514_Transcript_7/31_Confidence_0.588_Length_2145	REVERSE	54.57	42.86	CGACTAGGGTTTTCTTCTTCT						
Locus_3552_Transcript_133/133_Confidence_0.495_Length_4716	FORWARD	54.91	33.33	AAGCATGAAATCCTCAATACA	146	421	421	AG	AGAGAGAGAGAGAGAGAGAG	20
Locus_3552_Transcript_133/133_Confidence_0.495_Length_4716	REVERSE	55.68	38.1	AGCTTGGTGGCATATAGTTTT						
Locus_3567_Transcript_11/41_Confidence_0.437_Length_1143	FORWARD	55.18	33.33	AAATTTCTAGGGTGAATTTCG	142	424	424	GA	GAGAGAGAGAGAGAGAGAGAGAGA	24
Locus_3567_Transcript_11/41_Confidence_0.437_Length_1143	REVERSE	55.36	57.14	CGCTCTCTCTCTCTCTCTCTC						
Locus_3567_Transcript_11/41_Confidence_0.437_Length_1143	FORWARD	54.52	42.86	CCAGTAGAACCTTGAATCTGA	145	424	424	AG	AGAGAGAGAGAGAGAGAGAGAGAG	24

Locus_3567_Transcript_11/41_Confidence_0.437_Length_1143	REVERSE	55.39	50	TCTCTCTCTCTCTCTTGTGACC						
Locus_3567_Transcript_11/41_Confidence_0.437_Length_1143	FORWARD	54.77	52.17	AGAGAGAGAGAGAGAGAGAGAGC	167	424	424	AG	AGAGAGAGAGAGAGAGAG	18
Locus_3567_Transcript_11/41_Confidence_0.437_Length_1143	REVERSE	55.05	38.1	TTGTTGCCACTTGACTATTCT						
Locus_3599_Transcript_11/22_Confidence_0.635_Length_1716	FORWARD	55.58	42.86	ACCAGAAATCCTAACACATCC	163	427	427	AG	AGAGAGAGAGAGAGAGAGAGAGAGAG	26
Locus_3599_Transcript_11/22_Confidence_0.635_Length_1716	REVERSE	55.36	57.14	CGCTCTCTCTCTCTCTCTCTC						
Locus_3599_Transcript_11/22_Confidence_0.635_Length_1716	FORWARD	54.59	38.1	AAAAACTGTTGACGTACAAGG	168	427	427	AG	AGAGAGAGAGAGAGAGAGAGAGAG	24
Locus_3599_Transcript_11/22_Confidence_0.635_Length_1716	REVERSE	54.67	33.33	TGTCAACGATTTTCTCTTTTC						
Locus_3599_Transcript_11/22_Confidence_0.635_Length_1716	FORWARD	54.16	52.38	CAGCAGAGAGAGAGAGAGAGA	164	427	427	AG	AGAGAGAGAGAGAGAGAG	18
Locus_3599_Transcript_11/22_Confidence_0.635_Length_1716	REVERSE	55.31	38.1	GCCAAATCTACGCCTATTTAT						
Locus_3599_Transcript_11/22_Confidence_0.635_Length_1716	FORWARD	54.94	55	GTCGGTCACAAGAGAGAGAG	151	427	427	AG	AGAGAGAGAGAGAGAGAGAGAG	22
Locus_3599_Transcript_11/22_Confidence_0.635_Length_1716	REVERSE	54.3	42.86	CTGTCAACGATTTTCTCTAGG						
Locus_3599_Transcript_11/22_Confidence_0.635_Length_1716	FORWARD	54.84	38.1	GGATTTGACCTCTTTGGTATT	125	419	419	AG	AGAGAGAGAGAGAGAGAGAGAG	22
Locus_3599_Transcript_11/22_Confidence_0.635_Length_1716	REVERSE	55.31	38.1	GCCAAATCTACGCCTATTTAT						
Locus_3599_Transcript_11/22_Confidence_0.635_Length_1716	FORWARD	55.11	38.1	TCCTAGAGAAAATCGTTGACA	146	422	422	AG	AGAGAGAGAGAGAGAGAGAGAG	22
Locus_3599_Transcript_11/22_Confidence_0.635_Length_1716	REVERSE	55.31	42.86	TGAACTGAGCGAGTATTAGGA						
Locus_3647_Transcript_17/28_Confidence_0.605_Length_1346	FORWARD	54.45	42.86	GCTGATTGACTGTCTTCAGAT	157	421	421	TA	TATATATATATATATATATA	20
Locus_3647_Transcript_17/28_Confidence_0.605_Length_1346	REVERSE	55.02	35	CGAAAATGTGCATAGCAATA						
Locus_3707_Transcript_29/31_Confidence_0.653_Length_4149	FORWARD	55.57	42.86	ATCTAGTTGCAGTTTCCCACT	158	418	418	AG	AGAGAGAGAGAGAGAGAG	18
Locus_3707_Transcript_29/31_Confidence_0.653_Length_4149	REVERSE	54.93	47.62	TAAGTCGCTGCACCTATACTC						
Locus_3707_Transcript_29/31_Confidence_0.653_Length_4149	FORWARD	54.27	42.86	ACGGAGTACATAAATCGACAC	138	427	427	AG	AGAGAGAGAGAGAGAGAGAGAGAGAG	26
Locus_3707_Transcript_29/31_Confidence_0.653_Length_4149	REVERSE	55.36	57.14	CGCTCTCTCTCTCTCTCTCTC						
Locus_3707_Transcript_29/31_Confidence_0.653_Length_4149	FORWARD	54.57	33.33	TGAGCTTTAAAAAGCAAAGAG	151	427	427	AG	AGAGAGAGAGAGAGAGAGAGAGAG	24
Locus_3707_Transcript_29/31_Confidence_0.653_Length_4149	REVERSE	54.99	27.27	TTGGGATAAGCTAATTTTTGTT						
Locus_3738_Transcript_17/26_Confidence_0.610_Length_2600	FORWARD	53.96	28.57	GAAAGCAAATTATTGAAGGAA	133	423	423	GA	GAGAGAGAGAGAGAGAGAGAGA	22
Locus_3738_Transcript_17/26_Confidence_0.610_Length_2600	REVERSE	54.67	33.33	TGTCAACGATTTTCTCTTTTC						
Locus_3738_Transcript_17/26_Confidence_0.610_Length_2600	FORWARD	54.86	38.1	TGAAGGAATGTAATGGAGAGA	173	423	423	AG	AGAGAGAGAGAGAGAGAGAGAGAG	24
Locus_3738_Transcript_17/26_Confidence_0.610_Length_2600	REVERSE	55.31	38.1	GCCAAATCTACGCCTATTTAT						
Locus_3738_Transcript_17/26_Confidence_0.610_Length_2600	FORWARD	54.67	33.33	GAAAAGAGAAAATCGTTGACA	145	424	424	AG	AGAGAGAGAGAGAGAGAGAGAG	22
Locus_3738_Transcript_17/26_Confidence_0.610_Length_2600	REVERSE	55.14	47.62	AGGGAGATGTAGATCGAGAAG						
Locus_3751_Transcript_11/22_Confidence_0.652_Length_3704	FORWARD	55.06	42.86	AATCACGGAGAAGTAGAAACC	161	421	421	CT	CTCTCTCTCTCTCTCTCTCT	20
Locus_3751_Transcript_11/22_Confidence_0.652_Length_3704	REVERSE	55.2	42.86	ATAGGGGATTGGTATCAGAGA						
Locus_3804_Transcript_9/13_Confidence_0.714_Length_834	FORWARD	54.75	33.33	TTAACTCCACCAACACAATTT	158	422	422	AG	AGAGAGAGAGAGAGAGAGAGAG	22
Locus_3804_Transcript_9/13_Confidence_0.714_Length_834	REVERSE	55.2	38.1	CTTCCCAAGTGATTCTTCTTT						
Locus_3833_Transcript_28/31_Confidence_0.563_Length_2262	FORWARD	55.24	50	AAAGAGTAAGCCGTCCCTAC	177	427	427	GA	GAGAGAGAGAGAGAGAGAGAGAGAGA	26
Locus_3833_Transcript_28/31_Confidence_0.563_Length_2262	REVERSE	55.36	57.14	CGCTCTCTCTCTCTCTCTCTC						
Locus_3841_Transcript_11/17_Confidence_0.621_Length_1296	FORWARD	54.98	42.86	GAAAGTTAACACCTGGGACTT	150	420	420	AT	ATATATATATATATATATAT	20
Locus_3841_Transcript_11/17_Confidence_0.621_Length_1296	REVERSE	54.43	42.86	ACTGCACATAAAGACCGATAC						
Locus_3884_Transcript_4/26_Confidence_0.601_Length_1985	FORWARD	55.45	42.86	GTTTCTCTCTCGCTCGATATT	152	421	421	GA	GAGAGAGAGAGAGAGAGAGA	20
Locus_3884_Transcript_4/26_Confidence_0.601_Length_1985	REVERSE	57.48	55	CTCGCCTGTGATCTCTTACC						
Locus_3889_Transcript_15/25_Confidence_0.516_Length_1200	FORWARD	54.95	47.62	GTGTTGACAAGGTAGAAGACG	166	424	424	CT	CTCTCTCTCTCTCTCTCTCTCTCT	24
Locus_3889_Transcript_15/25_Confidence_0.516_Length_1200	REVERSE	54.69	47.62	TGTAAGAGCGAGAGAGAGAGA						
Locus_3912_Transcript_6/19_Confidence_0.588_Length_2575	FORWARD	55.2	47.62	TCCCTGTTCTCACTGTGTTAC	164	418	418	TC	TCTCTCTCTCTCTCTCTC	18
Locus_3912_Transcript_6/19_Confidence_0.588_Length_2575	REVERSE	54.86	33.33	ACTTTCAAGGTTAAGCCATTT						
Locus_3913_Transcript_15/41_Confidence_0.601_Length_1484	FORWARD	55.08	42.86	CAGTCAAACACCCACTAATGT	151	424	424	TC	TCTCTCTCTCTCTCTCTCTC	20
Locus_3913_Transcript_15/41_Confidence_0.601_Length_1484	REVERSE	55.09	47.62	GGTATAGGGGATTGGTATCAG						

Locus_3913_Transcript_15/41_Confidence_0.601_Length_1484	FORWARD	55.08	42.86	CAGTCAAACACCCACTAATGT	155	424	424	CT	CTCTCTCTCTCTCTCTCTCTCTCT	24
Locus_3913_Transcript_15/41_Confidence_0.601_Length_1484	REVERSE	55.09	42.86	AGATGGTATAGGGGATTGGTA						
Locus_3925_Transcript_14/26_Confidence_0.340_Length_1799	FORWARD	56.1	45	GAAGGCAGTTCTGGTTTCTT	152	424	424	CT	CTCTCTCTCTCTCTCTCTCTCTCT	24
Locus_3925_Transcript_14/26_Confidence_0.340_Length_1799	REVERSE	55.5	47.62	GAGAGAGGAGCTGGTTTTCTA						
Locus_3925_Transcript_14/26_Confidence_0.340_Length_1799	FORWARD	55.02	57.14	CGGACTCTCTCTCTCTCTCTC	169	424	424	CT	CTCTCTCTCTCTCTCTCTCTCTCT	24
Locus_3925_Transcript_14/26_Confidence_0.340_Length_1799	REVERSE	54.44	33.33	GTCGAAAGCAAATTATTGAAG						
Locus_3928_Transcript_117/135_Confidence_0.428_Length_6430	FORWARD	54.9	42.86	GATACTCAACTTTCGAGCGTA	148	420	420	CT	CTCTCTCTCTCTCTCTCTCT	20
Locus_3928_Transcript_117/135_Confidence_0.428_Length_6430	REVERSE	55.7	42.86	ATATTAGGAGTGGTTGGATGC						
Locus_3949_Transcript_61/161_Confidence_0.215_Length_7763	FORWARD	54.97	45.45	AGAAAGTAATCCCTCTCTCTCC	137	425	425	AG	AGAGAGAGAGAGAGAGAGAGAGAG	24
Locus_3949_Transcript_61/161_Confidence_0.215_Length_7763	REVERSE	55.36	57.14	CGCTCTCTCTCTCTCTCTCTC						
Locus_3949_Transcript_61/161_Confidence_0.215_Length_7763	FORWARD	55.28	40	AGCACTTGATTCCAACAAAC	140	425	425	AG	AGAGAGAGAGAGAGAGAGAGAGAG	24
Locus_3949_Transcript_61/161_Confidence_0.215_Length_7763	REVERSE	54.67	33.33	TGTCAACGATTTTCTCTTTTC						
Locus_3949_Transcript_61/161_Confidence_0.215_Length_7763	FORWARD	54.67	33.33	GAAAAGAGAAAATCGTTGACA	136	425	425	AG	AGAGAGAGAGAGAGAGAGAGAG	22
Locus_3949_Transcript_61/161_Confidence_0.215_Length_7763	REVERSE	54.65	42.86	ATCTTTCGGACTAAGTGGAGT						
Locus_3964_Transcript_42/46_Confidence_0.543_Length_1667	FORWARD	54.95	40	CACGACAAATACAGCAGAAA	230	420	420	GA	GAGAGAGAGAGAGAGAGAGA	20
Locus_3964_Transcript_42/46_Confidence_0.543_Length_1667	REVERSE	56.44	42.86	ATCATTCACCTCCACATTCTC						
Locus_3997_Transcript_26/50_Confidence_0.520_Length_3952	FORWARD	55.16	35	TTTTGAGCTCGGGATATTTA	219	421	421	AT	ATATATATATATATATATAT	20
Locus_3997_Transcript_26/50_Confidence_0.520_Length_3952	REVERSE	54.97	33.33	ATCACAAAAACCAATCACAAC						
Locus_4045_Transcript_3/25_Confidence_0.571_Length_1040	FORWARD	55.09	42.86	ATCATCATCTCCCTAACCTGT	153	425	425	AG	AGAGAGAGAGAGAGAGAGAGAGAG	24
Locus_4045_Transcript_3/25_Confidence_0.571_Length_1040	REVERSE	54.67	33.33	TGTCAACGATTTTCTCTTTTC						
Locus_4045_Transcript_3/25_Confidence_0.571_Length_1040	FORWARD	54.94	55	GTCGGTCACAAGAGAGAGAG	131	425	425	AG	AGAGAGAGAGAGAGAGAGAGAG	22
Locus_4045_Transcript_3/25_Confidence_0.571_Length_1040	REVERSE	54.84	38.1	TCTCTAAGCACCATAAAATGC						
Locus_4149_Transcript_59/95_Confidence_0.482_Length_2275	FORWARD	55.21	55	ATCTCAGAGCTCTCCCTCTC	150	424	424	CT	CTCTCTCTCTCTCTCTCTCTCT	22
Locus_4149_Transcript_59/95_Confidence_0.482_Length_2275	REVERSE	55.09	47.62	GGTATAGGGGATTGGTATCAG						
Locus_4149_Transcript_59/95_Confidence_0.482_Length_2275	FORWARD	54.14	38.1	TCCTAAAAAGGTTACAAGCTG	148	424	424	CT	CTCTCTCTCTCTCTCTCTCTCTCT	24
Locus_4149_Transcript_59/95_Confidence_0.482_Length_2275	REVERSE	54.69	47.62	TGTAAGAGCGAGAGAGAGAGA						
Locus_4190_Transcript_40/57_Confidence_0.503_Length_2043	FORWARD	55.18	38.1	ATTGGATCCTAACCACAACTT	131	423	423	AG	AGAGAGAGAGAGAGAGAGAGAG	22
Locus_4190_Transcript_40/57_Confidence_0.503_Length_2043	REVERSE	55.36	57.14	CGCTCTCTCTCTCTCTCTCTC						
Locus_4236_Transcript_27/36_Confidence_0.528_Length_2128	FORWARD	54.24	38.1	GATGAAGTTTATGAAGCTTGG	154	423	423	TC	TCTCTCTCTCTCTCTCTCTCTC	22
Locus_4236_Transcript_27/36_Confidence_0.528_Length_2128	REVERSE	54.82	42.86	TAGACCTGAACCATTGGAGTA						
Locus_4261_Transcript_34/58_Confidence_0.436_Length_1469	FORWARD	55.03	33.33	CAGAATTTCATTGATTTCAGC	144	423	423	TC	TCTCTCTCTCTCTCTCTCTCTC	22
Locus_4261_Transcript_34/58_Confidence_0.436_Length_1469	REVERSE	54.83	38.1	CACTTCCCTTAATCCCATATT						
Locus_4262_Transcript_18/28_Confidence_0.488_Length_918	FORWARD	55.76	52.38	CGGAAGATCTCTCTCTCTCAC	141	424	424	CT	CTCTCTCTCTCTCTCTCTCTCTCT	24
Locus_4262_Transcript_18/28_Confidence_0.488_Length_918	REVERSE	55.08	47.62	CTAGTAGGGGCAAGATGGTAT						
Locus_4313_Transcript_39/40_Confidence_0.515_Length_3949	FORWARD	55.23	38.1	GTTTCTCCCTATTGTTTGCTT	165	424	424	CT	CTCTCTCTCTCTCTCTCTCTCTCT	24
Locus_4313_Transcript_39/40_Confidence_0.515_Length_3949	REVERSE	55.2	42.86	ATAGGGGATTGGTATCAGAGA						
Locus_4313_Transcript_39/40_Confidence_0.515_Length_3949	FORWARD	55.23	38.1	GTTTCTCCCTATTGTTTGCTT	172	424	424	CT	CTCTCTCTCTCTCTCTCTCTCTCT	24
Locus_4313_Transcript_39/40_Confidence_0.515_Length_3949	REVERSE	55.09	42.86	AGATGGTATAGGGGATTGGTA						
Locus_4316_Transcript_4/10_Confidence_0.661_Length_1547	FORWARD	55.27	38.1	CTGCAGCTGAAGAAAGTTAAA	142	419	419	TG	TGTGTGTGTGTGTGTGTG	18
Locus_4316_Transcript_4/10_Confidence_0.661_Length_1547	REVERSE	55.12	47.62	GGTTATCCCACATACACACAC						
Locus_4316_Transcript_4/10_Confidence_0.661_Length_1547	FORWARD	55.27	38.1	CTGCAGCTGAAGAAAGTTAAA	149	419	419	TG	TGTGTGTGTGTGTGTGTG	18
Locus_4316_Transcript_4/10_Confidence_0.661_Length_1547	REVERSE	53.84	33.33	AAAAACAGGTTATCCCACATA						
Locus_4326_Transcript_18/42_Confidence_0.439_Length_1827	FORWARD	54.79	31.82	CAAGCTTGTATTTCTTTTCCTT	163	421	421	TC	TCTCTCTCTCTCTCTCTCTC	20
Locus_4326_Transcript_18/42_Confidence_0.439_Length_1827	REVERSE	55.14	33.33	GTAATTCGGCCAATTCTTATT						
Locus_4354_Transcript_27/35_Confidence_0.646_Length_1571	FORWARD	54.89	33.33	TTAGATGGAACCAGAAAATCA	135	425	425	AT	ATATATATATATATATATATATAT	24

Locus_4354_Transcript_27/35_Confidence_0.646_Length_1571	REVERSE	55.25	42.86	TGTTCAACAGAGGATTGAGTC						
Locus_4431_Transcript_6/20_Confidence_0.656_Length_1078	FORWARD	55.28	38.1	AAACCCCCTCAATACTCATAA	139	427	427	GA	GAGAGAGAGAGAGAGAGAGAGAGA	26
Locus_4431_Transcript_6/20_Confidence_0.656_Length_1078	REVERSE	55.36	57.14	CGCTCTCTCTCTCTCTCTCTC						
Locus_4431_Transcript_6/20_Confidence_0.656_Length_1078	FORWARD	55.44	52.17	GAGAGAGAGAGAGAGAGGAGAGA	156	427	427	AG	AGAGAGAGAGAGAGAGAGAGAGAG	24
Locus_4431_Transcript_6/20_Confidence_0.656_Length_1078	REVERSE	56.25	38.1	GCTGTCAACGATTTTCTCTTT						
Locus_4431_Transcript_6/20_Confidence_0.656_Length_1078	FORWARD	55.28	38.1	ACCCCCTCAATACTCATAAAA	137	427	427	GA	GAGAGAGAGAGAGAGAGAGAGAGA	24
Locus_4431_Transcript_6/20_Confidence_0.656_Length_1078	REVERSE	54.28	47.62	GCTTTTTCTCCTCTCTCTCTC						
Locus_4431_Transcript_6/20_Confidence_0.656_Length_1078	FORWARD	55.28	42.86	CCCCCTCAATACTCATAAAAC	162	427	427	AG	AGAGAGAGAGAGAGAGAGAGAG	22
Locus_4431_Transcript_6/20_Confidence_0.656_Length_1078	REVERSE	55.86	33.33	GGGGTTTTTGAAGAAACATTA						
Locus_4431_Transcript_6/20_Confidence_0.656_Length_1078	FORWARD	54.42	43.48	CAAATTGAGAGAGAGAGAGAGAG	195	422	422	GA	GAGAGAGAGAGAGAGAGAGAGA	22
Locus_4431_Transcript_6/20_Confidence_0.656_Length_1078	REVERSE	54.65	40	GCTGTCAACGATTTTCTCTT						
Locus_4464_Transcript_5/19_Confidence_0.670_Length_1168	FORWARD	53.96	38.1	AAAGATGGTATAGGGGATTTC	153	422	422	AG	AGAGAGAGAGAGAGAGAG	18
Locus_4464_Transcript_5/19_Confidence_0.670_Length_1168	REVERSE	54.53	42.86	TCAATCTCTCAATCTCTCCAG						
Locus_4464_Transcript_5/19_Confidence_0.670_Length_1168	FORWARD	54.94	55	GTCGGTCACAAGAGAGAGAG	140	422	422	AG	AGAGAGAGAGAGAGAGAGAGAG	22
Locus_4464_Transcript_5/19_Confidence_0.670_Length_1168	REVERSE	55.03	38.1	TCGACCTTATCAATCTCTCAA						
Locus_4473_Transcript_17/24_Confidence_0.561_Length_4523	FORWARD	55.42	42.86	GAAGAATCTCCCTTTGAACAC	171	422	422	CT	CTCTCTCTCTCTCTCTCTCTCT	22
Locus_4473_Transcript_17/24_Confidence_0.561_Length_4523	REVERSE	55.2	42.86	ATAGGGGATTGGTATCAGAGA						
Locus_4473_Transcript_17/24_Confidence_0.561_Length_4523	FORWARD	55.58	52.38	TATCGCCTCTCTCTCTCTCTC	151	422	422	CT	CTCTCTCTCTCTCTCTCTCTCT	22
Locus_4473_Transcript_17/24_Confidence_0.561_Length_4523	REVERSE	55.09	47.62	GGTATAGGGGATTGGTATCAG						
Locus_4473_Transcript_17/24_Confidence_0.561_Length_4523	FORWARD	55.42	42.86	GAAGAATCTCCCTTTGAACAC	171	423	423	CT	CTCTCTCTCTCTCTCTCTCTCT	22
Locus_4473_Transcript_17/24_Confidence_0.561_Length_4523	REVERSE	55.09	42.86	AGATGGTATAGGGGATTGGTA						
Locus_4503_Transcript_3/8_Confidence_0.706_Length_3834	FORWARD	54.46	38.1	CCACCAAACATTACCTAGAAA	161	418	418	AT	ATATATATATATATATAT	18
Locus_4503_Transcript_3/8_Confidence_0.706_Length_3834	REVERSE	55.35	33.33	ACCAAGGTTTACGATGAATTT						
Locus_4590_Transcript_15/20_Confidence_0.677_Length_749	FORWARD	55.09	47.62	GGTATAGGGGATTGGTATCAG	182	419	419	AG	AGAGAGAGAGAGAGAGAG	18
Locus_4590_Transcript_15/20_Confidence_0.677_Length_749	REVERSE	54.02	42.86	TGAGTTATATGCGTGAGTGAG						
Locus_4605_Transcript_13/17_Confidence_0.628_Length_1567	FORWARD	54.62	42.86	TTTGACTGAAGATGGAGAGAG	159	419	419	CT	CTCTCTCTCTCTCTCTCT	18
Locus_4605_Transcript_13/17_Confidence_0.628_Length_1567	REVERSE	55.23	38.1	AAAGAACAAGTTATGCCTTCC						
Locus_4628_Transcript_23/40_Confidence_0.525_Length_923	FORWARD	54.79	33.33	AGTGAAGCATGACATGAAAAT	155	424	424	AG	AGAGAGAGAGAGAGAGAGAGAGAG	24
Locus_4628_Transcript_23/40_Confidence_0.525_Length_923	REVERSE	55.28	52.38	GTCTTCCACTCTCTACCTGCT						
Locus_4643_Transcript_16/20_Confidence_0.626_Length_1411	FORWARD	55.31	38.1	CGTAAAACACTGCAGAAAAAC	161	424	424	AT	ATATATATATATATATATATATAT	24
Locus_4643_Transcript_16/20_Confidence_0.626_Length_1411	REVERSE	54.79	38.1	CTGATTCAATCCAGCTCTAAA						
Locus_4645_Transcript_37/45_Confidence_0.537_Length_6396	FORWARD	55	42.86	AAGGTGACCATATTCTGTGTG	145	421	421	TC	TCTCTCTCTCTCTCTCTCTC	20
Locus_4645_Transcript_37/45_Confidence_0.537_Length_6396	REVERSE	55.28	38.1	ATTCAAATTAGCTCGGGATAG						
Locus_4738_Transcript_98/111_Confidence_0.419_Length_2232	FORWARD	55.06	42.86	AAATCTACCCTGTTCGTTCTC	156	425	425	GA	GAGAGAGAGAGAGAGAGAGAGAGA	24
Locus_4738_Transcript_98/111_Confidence_0.419_Length_2232	REVERSE	55.36	57.14	CGCTCTCTCTCTCTCTCTCTC						
Locus_4738_Transcript_98/111_Confidence_0.419_Length_2232	FORWARD	54.37	52.38	GAGATAGCGAGTGAGTGAGAG	150	425	425	AG	AGAGAGAGAGAGAGAGAGAGAGAG	24
Locus_4738_Transcript_98/111_Confidence_0.419_Length_2232	REVERSE	54.67	33.33	TGTCAACGATTTTCTCTTTTC						
Locus_4738_Transcript_98/111_Confidence_0.419_Length_2232	FORWARD	54.67	33.33	GAAAAGAGAAAATCGTTGACA	148	425	425	AG	AGAGAGAGAGAGAGAGAGAGAG	22
Locus_4738_Transcript_98/111_Confidence_0.419_Length_2232	REVERSE	55.26	45	GAACACAGCATGGTTCTTCT						
Locus_4738_Transcript_98/111_Confidence_0.419_Length_2232	FORWARD	54.37	52.38	GTGAGAGAGAGATAGCGAGTG	158	422	422	GA	GAGAGAGAGAGAGAGAGAGAGAGA	24
Locus_4738_Transcript_98/111_Confidence_0.419_Length_2232	REVERSE	54.67	33.33	TGTCAACGATTTTCTCTTTTC						
Locus_4775_Transcript_12/14_Confidence_0.411_Length_569	FORWARD	53.86	23.81	TTTTCTTGCTTGAAAGTTTTT	159	424	424	TA	TATATATATATATATATATATATA	24
Locus_4775_Transcript_12/14_Confidence_0.411_Length_569	REVERSE	56.55	45	AGTCCTCCCCCAGATAATTT						
Locus_4775_Transcript_12/14_Confidence_0.411_Length_569	FORWARD	54.1	26.09	TCTTTTACTTTGAACAATGTTTG	156	424	424	TA	TATATATATATATATATATATATATA	26
Locus_4775_Transcript_12/14_Confidence_0.411_Length_569	REVERSE	53.84	42.86	ATACACATATGACCCCTGTTC						

Locus_4775_Transcript_12/14_Confidence_0.411_Length_569	FORWARD	55.31	38.1	TATAAATTATCTGGGGGAGGA	156	424	424	TA	TATATATATATATATATA	18
Locus_4775_Transcript_12/14_Confidence_0.411_Length_569	REVERSE	55.56	38.1	TTCACTCTTCTTATGGCATTG						
Locus_4799_Transcript_10/13_Confidence_0.656_Length_1905	FORWARD	55.34	38.1	AATTTCCTCAAACAGAGCTTC	158	418	418	TC	TCTCTCTCTCTCTCTCTC	18
Locus_4799_Transcript_10/13_Confidence_0.656_Length_1905	REVERSE	55.38	47.62	AGCTGGAGGAGAAGACATTAG						
Locus_4803_Transcript_22/29_Confidence_0.592_Length_2489	FORWARD	54.97	38.1	AAGATGAAGTGCAATAGGTGA	151	422	422	AG	AGAGAGAGAGAGAGAGAGAGAG	22
Locus_4803_Transcript_22/29_Confidence_0.592_Length_2489	REVERSE	55.36	57.14	CGCTCTCTCTCTCTCTCTCTC						
Locus_4803_Transcript_22/29_Confidence_0.592_Length_2489	FORWARD	54.82	23.81	TGAACATTTTTGGATTTGTTT	151	422	422	AG	AGAGAGAGAGAGAGAGAGAGAGAG	24
Locus_4803_Transcript_22/29_Confidence_0.592_Length_2489	REVERSE	55.39	50	TCTCTCTCTCTCTCTTGTGACC						
Locus_4803_Transcript_22/29_Confidence_0.592_Length_2489	FORWARD	54.59	38.1	AGCTCGAGTAATTCTGCAATA	189	422	422	AG	AGAGAGAGAGAGAGAGAG	18
Locus_4803_Transcript_22/29_Confidence_0.592_Length_2489	REVERSE	54.82	38.1	TGATATCTAAATGCCGCTAAC						
Locus_4829_Transcript_2/9_Confidence_0.692_Length_2752	FORWARD	54.74	33.33	AATCTGAATTGGACGTGAATA	144	421	421	AT	ATATATATATATATATATAT	20
Locus_4829_Transcript_2/9_Confidence_0.692_Length_2752	REVERSE	55.1	42.86	CATGGCTGAGCTCATAATAAC						
Locus_4872_Transcript_10/26_Confidence_0.577_Length_1460	FORWARD	55.07	42.86	ACAGTGCCACATCATATTCTC	148	426	426	CT	CTCTCTCTCTCTCTCTCTCTCTCTCT	26
Locus_4872_Transcript_10/26_Confidence_0.577_Length_1460	REVERSE	54.35	38.1	GTAGAGAGCGAAAGCAAATTA						
Locus_4903_Transcript_12/82_Confidence_0.411_Length_1608	FORWARD	54.94	42.86	TCTAACTGGCAAGAACAAGAG	144	423	423	CT	CTCTCTCTCTCTCTCTCTCTCT	22
Locus_4903_Transcript_12/82_Confidence_0.411_Length_1608	REVERSE	55.42	42.86	CGTCGTCTGTATTGTGAAACT						
Locus_5030_Transcript_17/56_Confidence_0.535_Length_1214	FORWARD	54.99	35	AGAGCGAAAGCAAATTATTG	135	423	423	GA	GAGAGAGAGAGAGAGAGAGAGA	22
Locus_5030_Transcript_17/56_Confidence_0.535_Length_1214	REVERSE	56.1	38.1	AGGTCCGGATGAACAATATAA						
Locus_5030_Transcript_17/56_Confidence_0.535_Length_1214	FORWARD	54.6	52.38	AGAGAGAGAGGAGAGAACTCG	138	423	423	AG	AGAGAGAGAGAGAGAGAG	18
Locus_5030_Transcript_17/56_Confidence_0.535_Length_1214	REVERSE	55.08	40	AACTTTGGGTTTTAGGGAAG						
Locus_5086_Transcript_68/93_Confidence_0.310_Length_2248	FORWARD	55	42.86	TGTTAGGAGGGATAATTAGGG	154	425	425	AG	AGAGAGAGAGAGAGAGAGAGAGAG	24
Locus_5086_Transcript_68/93_Confidence_0.310_Length_2248	REVERSE	54.68	33.33	TTTTTGAGGAACTTGAGAATG						
Locus_5108_Transcript_7/49_Confidence_0.566_Length_1833	FORWARD	55.4	38.1	TTCCCAACCTCATCTTCTTAT	163	424	424	TC	TCTCTCTCTCTCTCTCTCTC	20
Locus_5108_Transcript_7/49_Confidence_0.566_Length_1833	REVERSE	55.09	47.62	GGTATAGGGGATTGGTATCAG						
Locus_5108_Transcript_7/49_Confidence_0.566_Length_1833	FORWARD	55.4	42.86	CCCAACCTCATCTTCTTATTC	165	424	424	CT	CTCTCTCTCTCTCTCTCTCTCTCT	24
Locus_5108_Transcript_7/49_Confidence_0.566_Length_1833	REVERSE	55.09	42.86	AGATGGTATAGGGGATTGGTA						
Locus_5122_Transcript_4/27_Confidence_0.659_Length_2886	FORWARD	54.82	42.86	GGACAAGGAATTCTATCCTGT	147	419	419	AC	ACACACACACACACACAC	18
Locus_5122_Transcript_4/27_Confidence_0.659_Length_2886	REVERSE	55.04	33.33	TTTAATCTGCTTGTCTTTTGC						
Locus_5264_Transcript_8/13_Confidence_0.664_Length_2616	FORWARD	55.05	38.1	AGCAGGTGAACAGATTAACAA	147	420	420	AT	ATATATATATATATATATAT	20
Locus_5264_Transcript_8/13_Confidence_0.664_Length_2616	REVERSE	55.85	42.86	ATAAGTGGGAGCGATGATTAC						
Locus_5334_Transcript_22/44_Confidence_0.604_Length_2169	FORWARD	54.99	45	ATCTCAGGGCTGCATATAGA	150	421	421	GT	GTGTGTGTGTGTGTGTGTGT	20
Locus_5334_Transcript_22/44_Confidence_0.604_Length_2169	REVERSE	54.98	42.86	TTCGGAGGATAGTCAGTAACA						
Locus_5353_Transcript_9/10_Confidence_0.725_Length_2275	FORWARD	54.81	38.1	GCAGCAAACAGATTAACACTT	122	419	419	GA	GAGAGAGAGAGAGAGAGAGAGA	22
Locus_5353_Transcript_9/10_Confidence_0.725_Length_2275	REVERSE	54.86	47.83	TCTCTCTCTCTCTCTCTCTTGTG						
Locus_5372_Transcript_22/29_Confidence_0.505_Length_3049	FORWARD	55.13	42.86	TCTCTCTCCTCTTCACACAAA	154	418	418	CT	CTCTCTCTCTCTCTCTCT	18
Locus_5372_Transcript_22/29_Confidence_0.505_Length_3049	REVERSE	55.1	45	CCAAACCCTAACCACTGATA						
Locus_5426_Transcript_22/26_Confidence_0.571_Length_799	FORWARD	54.98	36.36	AAATATATCTCAATGCCCTGTC	131	423	423	GA	GAGAGAGAGAGAGAGAGAGAGA	22
Locus_5426_Transcript_22/26_Confidence_0.571_Length_799	REVERSE	56.1	38.1	CTTTCCACTTCTCGTCATTTT						
Locus_5493_Transcript_69/82_Confidence_0.534_Length_5620	FORWARD	54.62	38.1	GCTCTTGGTGAAAATATCAGA	150	419	419	AG	AGAGAGAGAGAGAGAGAG	18
Locus_5493_Transcript_69/82_Confidence_0.534_Length_5620	REVERSE	54.8	47.62	CAAGGCTCTCTTTCTCTCTCT						
Locus_5493_Transcript_69/82_Confidence_0.534_Length_5620	FORWARD	55.65	47.62	GTAAGGAGGTTCATTCGCTAC	152	419	419	AG	AGAGAGAGAGAGAGAGAG	18
Locus_5493_Transcript_69/82_Confidence_0.534_Length_5620	REVERSE	55.13	40.91	TCTCTATCAATCCTGAACTTCC						
Locus_5493_Transcript_69/82_Confidence_0.534_Length_5620	FORWARD	54.8	47.62	AGAGAGAGAAAGAGAGCCTTG	138	419	419	AG	AGAGAGAGAGAGAGAGAG	18
Locus_5493_Transcript_69/82_Confidence_0.534_Length_5620	REVERSE	55.21	40.91	TTCTTCTTCCTCCAAGTACATC						
Locus_5499_Transcript_18/24_Confidence_0.563_Length_2811	FORWARD	54.93	42.86	TCAAACCCTAATCTCCCTATC	152	419	419	CT	CTCTCTCTCTCTCTCTCT	18

Locus_5499_Transcript_18/24_Confidence_0.563_Length_2811	REVERSE	55.09	35	AGAAAAATCAGTTTGCTGGA						
Locus_5504_Transcript_5/23_Confidence_0.597_Length_2864	FORWARD	54.87	33.33	GGAAAACCAAAGATTACCATT	143	420	420	AT	ATATATATATATATATATAT	20
Locus_5504_Transcript_5/23_Confidence_0.597_Length_2864	REVERSE	54.02	38.1	TCTTGTAGTGACCAATCATCA						
Locus_5524_Transcript_28/34_Confidence_0.559_Length_1654	FORWARD	53.96	28.57	GAAAGCAAATTATTGAAGGAA	133	423	423	GA	GAGAGAGAGAGAGAGAGAGAGA	22
Locus_5524_Transcript_28/34_Confidence_0.559_Length_1654	REVERSE	54.67	33.33	TGTCAACGATTTTCTCTTTTC						
Locus_5524_Transcript_28/34_Confidence_0.559_Length_1654	FORWARD	53.96	28.57	GAAAGCAAATTATTGAAGGAA	178	423	423	AG	AGAGAGAGAGAGAGAGAG	18
Locus_5524_Transcript_28/34_Confidence_0.559_Length_1654	REVERSE	55.63	52.38	CGGATCTACAACTCTCCTCTC						
Locus_5524_Transcript_28/34_Confidence_0.559_Length_1654	FORWARD	54.67	33.33	GAAAAGAGAAAATCGTTGACA	137	423	423	AG	AGAGAGAGAGAGAGAGAGAGAG	22
Locus_5524_Transcript_28/34_Confidence_0.559_Length_1654	REVERSE	55.44	42.86	CTGAGTCAATGGTGAGAGAAA						
Locus_5599_Transcript_8/16_Confidence_0.544_Length_537	FORWARD	56.3	42.86	GCCAAATTCTGATCCAGTAAC	150	418	418	GA	GAGAGAGAGAGAGAGAGA	18
Locus_5599_Transcript_8/16_Confidence_0.544_Length_537	REVERSE	55.45	42.86	AGATTCAGTACCATCCGCTAT						
Locus_5657_Transcript_52/60_Confidence_0.469_Length_1878	FORWARD	54.24	42.86	AATGAAAGTCTTCTCCCTCTC	151	422	422	CT	CTCTCTCTCTCTCTCTCTCTCT	22
Locus_5657_Transcript_52/60_Confidence_0.469_Length_1878	REVERSE	55.09	42.86	AGATGGTATAGGGGATTGGTA						
Locus_5666_Transcript_13/30_Confidence_0.506_Length_2588	FORWARD	54.8	38.1	TTCTTTGATCTACACGTCCAT	160	421	421	TC	TCTCTCTCTCTCTCTCTCTC	20
Locus_5666_Transcript_13/30_Confidence_0.506_Length_2588	REVERSE	55.5	47.62	GAGAGAGGAGCTGGTTTTCTA						
Locus_5780_Transcript_23/33_Confidence_0.563_Length_1962	FORWARD	54.52	33.33	CTGCATAAATTGTCCTGATTT	136	419	419	AC	ACACACACACACACACAC	18
Locus_5780_Transcript_23/33_Confidence_0.563_Length_1962	REVERSE	54.76	28.57	GCTTCATTTGGTTTTGTTTTA						
Locus_5867_Transcript_12/24_Confidence_0.429_Length_1461	FORWARD	55.39	38.1	ATAAGCAAAAGGGAGTTCAAG	150	423	423	GA	GAGAGAGAGAGAGAGAGAGAGA	22
Locus_5867_Transcript_12/24_Confidence_0.429_Length_1461	REVERSE	55.36	57.14	CGCTCTCTCTCTCTCTCTCTC						
Locus_5867_Transcript_12/24_Confidence_0.429_Length_1461	FORWARD	55.49	42.86	AGCGAGAAATCTAAGCCATAC	175	423	423	AG	AGAGAGAGAGAGAGAGAGAGAGAG	24
Locus_5867_Transcript_12/24_Confidence_0.429_Length_1461	REVERSE	55.22	47.62	TGGGATCTCTCTTTCTCTCTC						
Locus_5867_Transcript_12/24_Confidence_0.429_Length_1461	FORWARD	54.25	38.1	GAAAGAGAAAATCGTTGACAG	153	423	423	AG	AGAGAGAGAGAGAGAGAGAGAG	22
Locus_5867_Transcript_12/24_Confidence_0.429_Length_1461	REVERSE	55.34	47.62	TGACTCAGCAGTCCTCTCTTA						
Locus_5931_Transcript_12/22_Confidence_0.654_Length_1580	FORWARD	54.99	40	TGTTTCGTCCTCAGAAGTTT	148	419	419	TC	TCTCTCTCTCTCTCTCTC	18
Locus_5931_Transcript_12/22_Confidence_0.654_Length_1580	REVERSE	54.89	36.36	CAAGTAAACGAAGAAGACAACA						
Locus_6033_Transcript_8/11_Confidence_0.660_Length_977	FORWARD	54.55	38.1	TAATTAAGCACACGTCTCACA	149	419	419	TA	TATATATATATATATATA	18
Locus_6033_Transcript_8/11_Confidence_0.660_Length_977	REVERSE	54.05	33.33	TCCCGATTAGGAACATATAAA						
Locus_6093_Transcript_12/19_Confidence_0.648_Length_1495	FORWARD	55.91	42.86	CCGAATAGGAAGATTCTGTGT	161	418	418	AG	AGAGAGAGAGAGAGAGAG	18
Locus_6093_Transcript_12/19_Confidence_0.648_Length_1495	REVERSE	56.3	50	GCTAGAGATCGGATCAGCTT						
Locus_6256_Transcript_39/47_Confidence_0.548_Length_1587	FORWARD	54.84	57.14	GAGAGTGGGAGAGAGAGAGAG	146	424	424	GA	GAGAGAGAGAGAGAGAGAGAGAGA	24
Locus_6256_Transcript_39/47_Confidence_0.548_Length_1587	REVERSE	55.3	28.57	TTTTTCCTTCAGCACAAATTA						
Locus_6274_Transcript_13/17_Confidence_0.642_Length_1149	FORWARD	55.06	38.1	TCCAAATCCTGTGTTGTATTC	142	422	422	AG	AGAGAGAGAGAGAGAGAGAGAG	22
Locus_6274_Transcript_13/17_Confidence_0.642_Length_1149	REVERSE	54.43	38.1	CACACATTCCAAACTTCTTCT						
Locus_6308_Transcript_28/31_Confidence_0.502_Length_1512	FORWARD	56.94	50	CACGAAGACTCCAGAAGGTT	148	421	421	TC	TCTCTCTCTCTCTCTCTCTC	20
Locus_6308_Transcript_28/31_Confidence_0.502_Length_1512	REVERSE	54.83	38.1	AAAAGATGGTATAGGGGATTG						
Locus_6308_Transcript_28/31_Confidence_0.502_Length_1512	FORWARD	55.39	47.62	CCTGTAAATCCCATCTCTCTC	148	421	421	CT	CTCTCTCTCTCTCTCTCTCTCTCT	24
Locus_6308_Transcript_28/31_Confidence_0.502_Length_1512	REVERSE	55.23	42.86	CATCTACAACAAGAAGGCAAG						
Locus_6531_Transcript_1/15_Confidence_0.540_Length_913	FORWARD	54.46	38.1	GGAAACCAACAGTAAACAAGA	132	425	425	AG	AGAGAGAGAGAGAGAGAGAGAGAG	24
Locus_6531_Transcript_1/15_Confidence_0.540_Length_913	REVERSE	54.67	33.33	TGTCAACGATTTTCTCTTTTC						
Locus_6531_Transcript_1/15_Confidence_0.540_Length_913	FORWARD	54.49	50	GGATCAGAGAGAGAGAGAGAGA	159	425	425	AG	AGAGAGAGAGAGAGAGAGAGAG	22
Locus_6531_Transcript_1/15_Confidence_0.540_Length_913	REVERSE	55.31	38.1	GCCAAATCTACGCCTATTTAT						
Locus_6531_Transcript_1/15_Confidence_0.540_Length_913	FORWARD	54.46	38.1	GGAAACCAACAGTAAACAAGA	132	424	424	AG	AGAGAGAGAGAGAGAGAGAGAGAG	24
Locus_6531_Transcript_1/15_Confidence_0.540_Length_913	REVERSE	54.67	33.33	TGTCAACGATTTTCTCTTTTC						
Locus_6531_Transcript_1/15_Confidence_0.540_Length_913	FORWARD	54.46	38.1	GGAAACCAACAGTAAACAAGA	175	424	424	AG	AGAGAGAGAGAGAGAGAG	18
Locus_6531_Transcript_1/15_Confidence_0.540_Length_913	REVERSE	55	47.62	CGCCTATTTATCTCCTCTCTC						

Locus_6531_Transcript_1/15_Confidence_0.540_Length_913	FORWARD	54.49	50	GGATCAGAGAGAGAGAGAGAGA	159	424	424	AG	AGAGAGAGAGAGAGAGAGAGAG	22
Locus_6531_Transcript_1/15_Confidence_0.540_Length_913	REVERSE	55.31	38.1	GCCAAATCTACGCCTATTTAT						
Locus_6565_Transcript_3/20_Confidence_0.630_Length_1278	FORWARD	55.26	47.62	CCTTAGCCAGGTCAATAGTCT	210	424	424	TC	TCTCTCTCTCTCTCTCTCTC	20
Locus_6565_Transcript_3/20_Confidence_0.630_Length_1278	REVERSE	55.2	42.86	ATAGGGGATTGGTATCAGAGA						
Locus_6565_Transcript_3/20_Confidence_0.630_Length_1278	FORWARD	54.63	47.62	AGTCTCGATCCATCTCTCTCT	140	424	424	CT	CTCTCTCTCTCTCTCTCTCTCTCT	24
Locus_6565_Transcript_3/20_Confidence_0.630_Length_1278	REVERSE	54.17	33.33	CTTCCCTTAATCCCATATTTT						
Locus_6614_Transcript_5/6_Confidence_0.886_Length_2115	FORWARD	56.23	42.86	CCGAGTAGTCTTGCGATTTAT	137	424	424	CT	CTCTCTCTCTCTCTCTCTCTCTCT	24
Locus_6614_Transcript_5/6_Confidence_0.886_Length_2115	REVERSE	54.83	38.1	AAAAGATGGTATAGGGGATTG						
Locus_6632_Transcript_34/37_Confidence_0.605_Length_2723	FORWARD	55.5	42.86	AGCTTTGAATCTCTCTTCCTG	148	426	426	GA	GAGAGAGAGAGAGAGAGAGAGA	22
Locus_6632_Transcript_34/37_Confidence_0.605_Length_2723	REVERSE	55.36	57.14	CGCTCTCTCTCTCTCTCTCTC						
Locus_6632_Transcript_34/37_Confidence_0.605_Length_2723	FORWARD	55.52	38.1	TCGACTTGACTAAGCTTTGAA	154	426	426	AG	AGAGAGAGAGAGAGAGAGAGAGAG	24
Locus_6632_Transcript_34/37_Confidence_0.605_Length_2723	REVERSE	54.67	33.33	TGTCAACGATTTTCTCTTTTC						
Locus_6632_Transcript_34/37_Confidence_0.605_Length_2723	FORWARD	55.14	55	CGGTCACAAGAGAGAGAGAG	131	426	426	AG	AGAGAGAGAGAGAGAGAGAGAG	22
Locus_6632_Transcript_34/37_Confidence_0.605_Length_2723	REVERSE	54.75	38.1	CCATTTATTTAGGGAAGAAGG						
Locus_6632_Transcript_34/37_Confidence_0.605_Length_2723	FORWARD	54.67	33.33	GAAAAGAGAAAATCGTTGACA	130	423	423	AG	AGAGAGAGAGAGAGAGAGAGAG	22
Locus_6632_Transcript_34/37_Confidence_0.605_Length_2723	REVERSE	55.2	38.1	GGCGGATTTTACTATTGTCTT						
Locus_6651_Transcript_10/22_Confidence_0.628_Length_4410	FORWARD	55.18	47.62	CATGGCTAAGTATCTCACTCG	147	421	421	TC	TCTCTCTCTCTCTCTCTCTC	20
Locus_6651_Transcript_10/22_Confidence_0.628_Length_4410	REVERSE	54.71	33.33	GAGTTACAAATGGCGAAAATA						
Locus_6680_Transcript_3/3_Confidence_0.600_Length_340	FORWARD	54.4	31.82	AAATACATAAGAATTGGGTGGT	152	340	340	AG	AGAGAGAGAGAGAGAGAG	18
Locus_6680_Transcript_3/3_Confidence_0.600_Length_340	REVERSE	54.96	42.86	TCTCAGCTCTTCAGAATCTTG						
Locus_6978_Transcript_10/30_Confidence_0.526_Length_757	FORWARD	55.55	47.62	GACTTGTAGGGTTCTTTGAGG	175	423	423	CT	CTCTCTCTCTCTCTCTCTCTCT	22
Locus_6978_Transcript_10/30_Confidence_0.526_Length_757	REVERSE	55.09	42.86	AGATGGTATAGGGGATTGGTA						
Locus_7044_Transcript_24/25_Confidence_0.599_Length_1748	FORWARD	56.14	42.86	ATCAGCTGAGGCAAATATCTC	136	419	419	GA	GAGAGAGAGAGAGAGAGAGAGA	22
Locus_7044_Transcript_24/25_Confidence_0.599_Length_1748	REVERSE	54.74	33.33	AACGGATCAAATCTAATCACA						
Locus_7044_Transcript_24/25_Confidence_0.599_Length_1748	FORWARD	55.2	57.14	CCGAGAGAGAGAGAGAGAGAG	152	419	419	AG	AGAGAGAGAGAGAGAGAG	18
Locus_7044_Transcript_24/25_Confidence_0.599_Length_1748	REVERSE	54.8	33.33	CTAAACCTAATGCAAACCAAA						
Locus_7232_Transcript_6/7_Confidence_0.556_Length_844	FORWARD	55.34	52.38	GTAGAGGGGTCAATGTAGAGG	153	418	418	GA	GAGAGAGAGAGAGAGAGA	18
Locus_7232_Transcript_6/7_Confidence_0.556_Length_844	REVERSE	54.97	38.1	GGGTAGAGCCAATGATTTTAT						
Locus_7321_Transcript_22/28_Confidence_0.609_Length_1856	FORWARD	54.91	38.1	AACTCTGCGTCAATACAACAT	150	424	424	TC	TCTCTCTCTCTCTCTCTCTCTC	22
Locus_7321_Transcript_22/28_Confidence_0.609_Length_1856	REVERSE	55.09	47.62	GGTATAGGGGATTGGTATCAG						
Locus_7321_Transcript_22/28_Confidence_0.609_Length_1856	FORWARD	54.91	38.1	AACTCTGCGTCAATACAACAT	154	424	424	CT	CTCTCTCTCTCTCTCTCTCTCTCT	24
Locus_7321_Transcript_22/28_Confidence_0.609_Length_1856	REVERSE	55.09	42.86	AGATGGTATAGGGGATTGGTA						
Locus_7441_Transcript_11/33_Confidence_0.602_Length_1929	FORWARD	54.89	45	CAAACCCTAACCTTTCACTG	179	423	423	CT	CTCTCTCTCTCTCTCTCTCTCT	22
Locus_7441_Transcript_11/33_Confidence_0.602_Length_1929	REVERSE	54.03	57.14	GGGAGAGAGAGAGAGAGAGAG						
Locus_7441_Transcript_11/33_Confidence_0.602_Length_1929	FORWARD	55.36	57.14	CTCGCTCTCTCTCTCTCTCTC	150	423	423	CT	CTCTCTCTCTCTCTCTCTCTCT	22
Locus_7441_Transcript_11/33_Confidence_0.602_Length_1929	REVERSE	55.09	47.62	GGTATAGGGGATTGGTATCAG						
Locus_7441_Transcript_11/33_Confidence_0.602_Length_1929	FORWARD	55.36	57.14	CGCTCTCTCTCTCTCTCTCTC	152	423	423	CT	CTCTCTCTCTCTCTCTCTCTCTCT	24
Locus_7441_Transcript_11/33_Confidence_0.602_Length_1929	REVERSE	55.09	42.86	AGATGGTATAGGGGATTGGTA						
Locus_7441_Transcript_11/33_Confidence_0.602_Length_1929	FORWARD	55.08	47.37	GTGGTTTTCAGGTCGAAGT	188	424	424	CT	CTCTCTCTCTCTCTCTCTCTCT	22
Locus_7441_Transcript_11/33_Confidence_0.602_Length_1929	REVERSE	55.2	42.86	ATAGGGGATTGGTATCAGAGA						
Locus_7518_Transcript_13/14_Confidence_0.680_Length_7512	FORWARD	54.92	31.82	TTGTCGTGTAAAGTTTTTGAAG	151	424	424	AT	ATATATATATATATATATATATAT	24
Locus_7518_Transcript_13/14_Confidence_0.680_Length_7512	REVERSE	54.64	52.38	CTCTCTCTCTCTCTCCACACA						
Locus_7560_Transcript_20/20_Confidence_0.400_Length_1869	FORWARD	55.25	33.33	TTTCATTTCCTCTTTCCTTTC	141	420	420	AG	AGAGAGAGAGAGAGAGAGAG	20
Locus_7560_Transcript_20/20_Confidence_0.400_Length_1869	REVERSE	56	57.14	CTCTCTCTCTCTCTCCCCTCT						
Locus_7560_Transcript_20/20_Confidence_0.400_Length_1869	FORWARD	55.03	45	AGAGCAAAGAATACGCAGAG	157	420	420	GA	GAGAGAGAGAGAGAGAGAGAGAGA	24

Locus_7560_Transcript_20/20_Confidence_0.400_Length_1869	REVERSE	54.67	33.33	TGTCAACGATTTTCTCTTTTC						
Locus_7560_Transcript_20/20_Confidence_0.400_Length_1869	FORWARD	54.25	52.38	TACGCAGAGAGAGAGAGAGAG	147	420	420	AG	AGAGAGAGAGAGAGAGAG	18
Locus_7560_Transcript_20/20_Confidence_0.400_Length_1869	REVERSE	53.88	33.33	CTGTCAACGATTTTCTCTTTT						
Locus_7560_Transcript_20/20_Confidence_0.400_Length_1869	FORWARD	55.14	55	CGGTCACAAGAGAGAGAGAG	153	420	420	AG	AGAGAGAGAGAGAGAGAGAGAG	22
Locus_7560_Transcript_20/20_Confidence_0.400_Length_1869	REVERSE	55.59	45	CCTTCAATGCATCCAGTATC						
Locus_7560_Transcript_20/20_Confidence_0.400_Length_1869	FORWARD	53.68	42.86	GAGAAGCAGAGCAAAGAATAC	164	422	422	AG	AGAGAGAGAGAGAGAGAGAG	20
Locus_7560_Transcript_20/20_Confidence_0.400_Length_1869	REVERSE	54.67	33.33	TGTCAACGATTTTCTCTTTTC						
Locus_7603_Transcript_52/55_Confidence_0.389_Length_3224	FORWARD	55.97	61.11	GGAGAGCGAAGTGAGAGG	151	424	424	GA	GAGAGAGAGAGAGAGAGAGAGAGA	24
Locus_7603_Transcript_52/55_Confidence_0.389_Length_3224	REVERSE	55.38	47.62	ATTCGTTAGTCTCGGAGAGAG						
Locus_7603_Transcript_52/55_Confidence_0.389_Length_3224	FORWARD	55.14	55	AAGTGAGAGGGGAGAGAGAG	143	424	424	AG	AGAGAGAGAGAGAGAGAGAGAGAG	24
Locus_7603_Transcript_52/55_Confidence_0.389_Length_3224	REVERSE	55.38	47.62	ATTCGTTAGTCTCGGAGAGAG						
Locus_7603_Transcript_52/55_Confidence_0.389_Length_3224	FORWARD	54.25	38.1	GAAAGAGAAAATCGTTGACAG	160	424	424	AG	AGAGAGAGAGAGAGAGAGAGAG	22
Locus_7603_Transcript_52/55_Confidence_0.389_Length_3224	REVERSE	54.57	38.1	CTATAACAAACGCCAAAAGAG						
Locus_7642_Transcript_3/10_Confidence_0.727_Length_2178	FORWARD	55.33	33.33	ATGGGAAGGATGATAAAGAAA	143	419	419	TC	TCTCTCTCTCTCTCTCTC	18
Locus_7642_Transcript_3/10_Confidence_0.727_Length_2178	REVERSE	54.56	33.33	AAAACCAAACCTACCATCTTT						
Locus_7849_Transcript_10/14_Confidence_0.618_Length_3090	FORWARD	54.76	38.1	AGGTTTGATTCAGAGGCTAAT	157	422	422	AT	ATATATATATATATATATATAT	22
Locus_7849_Transcript_10/14_Confidence_0.618_Length_3090	REVERSE	54.03	33.33	CCCATCATCATTATTAAAAGC						
Locus_7857_Transcript_9/28_Confidence_0.611_Length_1752	FORWARD	53.94	47.37	CTACGGTGGAAAAACACAC	124	421	421	CT	CTCTCTCTCTCTCTCTCTCT	20
Locus_7857_Transcript_9/28_Confidence_0.611_Length_1752	REVERSE	55.52	38.1	TATAATGCGGGTATAATGCAG						
Locus_8024_Transcript_27/51_Confidence_0.617_Length_2131	FORWARD	54.96	47.62	CACAGTCGAAGGATGATAGAG	137	422	422	AG	AGAGAGAGAGAGAGAGAG	18
Locus_8024_Transcript_27/51_Confidence_0.617_Length_2131	REVERSE	55.78	38.1	ATCGCAGAGAAGTTCAAATCT						
Locus_8193_Transcript_11/13_Confidence_0.635_Length_2403	FORWARD	55.25	36.36	CGAAAATACCAACTCTCTCATT	213	423	423	CT	CTCTCTCTCTCTCTCTCTCTCT	22
Locus_8193_Transcript_11/13_Confidence_0.635_Length_2403	REVERSE	55.15	47.62	GAGGATCTAGGGTTTCTACGA						
Locus_8193_Transcript_11/13_Confidence_0.635_Length_2403	FORWARD	54.99	47.62	ACCAACTCTCTCATTCCTCTC	206	423	423	CT	CTCTCTCTCTCTCTCTCTCT	20
Locus_8193_Transcript_11/13_Confidence_0.635_Length_2403	REVERSE	55.15	47.62	GAGGATCTAGGGTTTCTACGA						
Locus_8193_Transcript_11/13_Confidence_0.635_Length_2403	FORWARD	53.76	52.38	CTCTCTCTCTCTCTCTTGCTG	149	423	423	CT	CTCTCTCTCTCTCTCTCTCTCTCT	24
Locus_8193_Transcript_11/13_Confidence_0.635_Length_2403	REVERSE	54.98	42.86	GGTTTCTACGAAGAGATGGAT						
Locus_8217_Transcript_4/10_Confidence_0.652_Length_1065	FORWARD	55.17	47.62	CTCTCACAATCTCCCTCTTCT	145	418	418	TC	TCTCTCTCTCTCTCTCTC	18
Locus_8217_Transcript_4/10_Confidence_0.652_Length_1065	REVERSE	55.28	38.1	CGTTTTAGTTTCCGATTAGGT						
Locus_8244_Transcript_58/64_Confidence_0.480_Length_4087	FORWARD	54.52	45	GAATGGTATCGGAGAAACAC	155	420	420	AG	AGAGAGAGAGAGAGAGAGAG	20
Locus_8244_Transcript_58/64_Confidence_0.480_Length_4087	REVERSE	54.58	38.1	CTTCTTAAAGCTTTGTTGTGG						
Locus_8440_Transcript_21/37_Confidence_0.491_Length_1568	FORWARD	55.32	47.62	ATCTATCACACTCCTCCTCGT	145	422	422	AG	AGAGAGAGAGAGAGAGAGAGAG	22
Locus_8440_Transcript_21/37_Confidence_0.491_Length_1568	REVERSE	55.36	57.14	CGCTCTCTCTCTCTCTCTCTC						
Locus_8440_Transcript_21/37_Confidence_0.491_Length_1568	FORWARD	54.03	57.14	GAGAGAGAGAGAGGAGAGAGG	129	422	422	AG	AGAGAGAGAGAGAGAGAGAGAGAG	24
Locus_8440_Transcript_21/37_Confidence_0.491_Length_1568	REVERSE	54.67	33.33	TGTCAACGATTTTCTCTTTTC						
Locus_8440_Transcript_21/37_Confidence_0.491_Length_1568	FORWARD	54.03	57.14	GAGAGAGAGAGAGGAGAGAGG	145	423	423	AG	AGAGAGAGAGAGAGAGAG	18
Locus_8440_Transcript_21/37_Confidence_0.491_Length_1568	REVERSE	55.16	45	CTCCAGAAGTTTTTGCTGTC						
Locus_8487_Transcript_14/18_Confidence_0.643_Length_3378	FORWARD	55.39	42.86	CGTGGCTAATATGCAGTAAAG	153	418	418	AT	ATATATATATATATATAT	18
Locus_8487_Transcript_14/18_Confidence_0.643_Length_3378	REVERSE	54.35	33.33	ACACAAATCCATAAGAATTGC						
Locus_8496_Transcript_15/18_Confidence_0.588_Length_2531	FORWARD	54.74	42.86	TCGGTGTGACTTAAATCTAGC	157	418	418	AC	ACACACACACACACACAC	18
Locus_8496_Transcript_15/18_Confidence_0.588_Length_2531	REVERSE	54.91	35	GCAACGGTTCATTTTATTTC						
Locus_8570_Transcript_11/17_Confidence_0.682_Length_4518	FORWARD	55.48	38.1	CATCATGATTAGCTCTTTCCA	131	418	418	TC	TCTCTCTCTCTCTCTCTC	18
Locus_8570_Transcript_11/17_Confidence_0.682_Length_4518	REVERSE	54.31	42.86	GACAAAGAGCGAGCTATAAGA						
Locus_8699_Transcript_5/16_Confidence_0.632_Length_1707	FORWARD	55.47	47.62	GGAAGAAGATGACCAAGGTAG	163	428	428	GT	GTGTGTGTGTGTGTGTGTGTGTGTGTGT	28
Locus_8699_Transcript_5/16_Confidence_0.632_Length_1707	REVERSE	55.03	38.1	TTCCACCCCTCTAATTTTTAC						

Locus_8704_Transcript_7/12_Confidence_0.615_Length_1013	FORWARD	54.94	38.1	GCAGAAGAGAATGCATAAAGA	151	419	419	GA	GAGAGAGAGAGAGAGAGA	18
Locus_8704_Transcript_7/12_Confidence_0.615_Length_1013	REVERSE	54.85	33.33	AGAAGACGATGATGATTCAAA						
Locus_8710_Transcript_11/15_Confidence_0.509_Length_1052	FORWARD	54.95	38.1	ACCAAGATTTTCATTCCTCTC	159	418	418	GA	GAGAGAGAGAGAGAGAGA	18
Locus_8710_Transcript_11/15_Confidence_0.509_Length_1052	REVERSE	55.34	47.37	GCTGTGACGATTTTCCTCT						
Locus_8720_Transcript_12/51_Confidence_0.558_Length_2071	FORWARD	54.97	47.62	GAGGAATCGAGAAAGAGAGAG	167	423	423	CT	CTCTCTCTCTCTCTCTCTCTCT	22
Locus_8720_Transcript_12/51_Confidence_0.558_Length_2071	REVERSE	55.09	42.86	AGATGGTATAGGGGATTGGTA						
Locus_8762_Transcript_22/25_Confidence_0.610_Length_3370	FORWARD	54.91	38.1	ATTCATCTCGGGGAATATTAG	149	418	418	TA	TATATATATATATATATA	18
Locus_8762_Transcript_22/25_Confidence_0.610_Length_3370	REVERSE	54.65	42.86	TAATCAAGAGATCCGTGTAGC						
Locus_8771_Transcript_9/20_Confidence_0.504_Length_705	FORWARD	54.47	42.86	TCATATACTCCATACGGCTTC	154	424	424	CT	CTCTCTCTCTCTCTCTCTCTCTCT	24
Locus_8771_Transcript_9/20_Confidence_0.504_Length_705	REVERSE	55.09	42.86	AGATGGTATAGGGGATTGGTA						
Locus_8898_Transcript_13/18_Confidence_0.625_Length_798	FORWARD	54.99	42.86	ACTTGGATTTGAGGAGGATAG	138	418	418	AG	AGAGAGAGAGAGAGAGAG	18
Locus_8898_Transcript_13/18_Confidence_0.625_Length_798	REVERSE	54.31	33.33	ATCAATCTTCGAATCTGCTTA						
Locus_8935_Transcript_1/19_Confidence_0.488_Length_1408	FORWARD	54.92	50	TCTCTCTCTCTCTCTCTTCTGC	138	424	424	CT	CTCTCTCTCTCTCTCTCTCTCTCT	24
Locus_8935_Transcript_1/19_Confidence_0.488_Length_1408	REVERSE	54.82	38.1	AATGTGTGTTTAGAGCGAAAG						
Locus_9071_Transcript_14/15_Confidence_0.559_Length_1189	FORWARD	55.58	50	CGTACATTGTGAACCTCCTC	145	424	424	CT	CTCTCTCTCTCTCTCTCT	18
Locus_9071_Transcript_14/15_Confidence_0.559_Length_1189	REVERSE	54.83	38.1	AAAAGATGGTATAGGGGATTG						
Locus_9071_Transcript_14/15_Confidence_0.559_Length_1189	FORWARD	56	57.14	CTCTCCCTCCTCTCTCTCTCT	146	424	424	CT	CTCTCTCTCTCTCTCTCTCTCTCT	24
Locus_9071_Transcript_14/15_Confidence_0.559_Length_1189	REVERSE	54.35	33.33	TTTCAAACACTGTGAAACAGA						
Locus_9192_Transcript_5/16_Confidence_0.500_Length_2375	FORWARD	55.26	38.1	ATGACATAACTTGAGCATTGG	148	424	424	TC	TCTCTCTCTCTCTCTCTCTCTCTC	24
Locus_9192_Transcript_5/16_Confidence_0.500_Length_2375	REVERSE	55.58	42.86	TTAGGGTTTACAGAACGAGGT						
Locus_9385_Transcript_5/19_Confidence_0.503_Length_1396	FORWARD	55.7	47.62	GTAACAAGTCTTCGGAGAAGG	137	424	424	GA	GAGAGAGAGAGAGAGAGAGAGAGA	24
Locus_9385_Transcript_5/19_Confidence_0.503_Length_1396	REVERSE	54.67	33.33	TGTCAACGATTTTCTCTTTTC						
Locus_9385_Transcript_5/19_Confidence_0.503_Length_1396	FORWARD	54.67	33.33	GAAAAGAGAAAATCGTTGACA	156	424	424	AG	AGAGAGAGAGAGAGAGAGAGAG	22
Locus_9385_Transcript_5/19_Confidence_0.503_Length_1396	REVERSE	55.27	42.86	TATGCTGCTCCCTCACTATAA						
Locus_9385_Transcript_5/19_Confidence_0.503_Length_1396	FORWARD	55.7	47.62	GTAACAAGTCTTCGGAGAAGG	138	419	419	AG	AGAGAGAGAGAGAGAGAG	18
Locus_9385_Transcript_5/19_Confidence_0.503_Length_1396	REVERSE	53.88	33.33	CTGTCAACGATTTTCTCTTTT						
Locus_9396_Transcript_6/9_Confidence_0.755_Length_1345	FORWARD	55.1	47.62	GAATGGAGTAGAGGGAGAAGA	151	420	420	CT	CTCTCTCTCTCTCTCTCTCT	20
Locus_9396_Transcript_6/9_Confidence_0.755_Length_1345	REVERSE	55.93	42.86	GGAAAAGCTTCGATTACAGAG						
Locus_9460_Transcript_17/20_Confidence_0.553_Length_1704	FORWARD	55.08	33.33	TCAGAAAGTGAATGAAAGGAA	160	420	420	GA	GAGAGAGAGAGAGAGAGAGA	20
Locus_9460_Transcript_17/20_Confidence_0.553_Length_1704	REVERSE	53.58	42.86	AAGACCTTCTCCTCTTTTCTC						
Locus_9478_Transcript_4/12_Confidence_0.788_Length_862	FORWARD	54.16	52.38	TCTCTCTCTCTCTCTCTGCTG	150	424	424	CT	CTCTCTCTCTCTCTCTCTCTCTCT	24
Locus_9478_Transcript_4/12_Confidence_0.788_Length_862	REVERSE	54.77	45	GTAAGGGAAACGATCATACG						
Locus_9502_Transcript_8/19_Confidence_0.651_Length_1315	FORWARD	55.03	38.1	AAAATCTTCTCTTGGAACCAC	143	426	426	CT	CTCTCTCTCTCTCTCTCTCTCTCTCT	26
Locus_9502_Transcript_8/19_Confidence_0.651_Length_1315	REVERSE	55.54	47.62	AGGAATGTAAGAGCGAGAGAG						
Locus_9557_Transcript_13/16_Confidence_0.576_Length_1988	FORWARD	54.61	42.86	ACCTCTATAGGGTTCCTCAAA	156	418	418	CT	CTCTCTCTCTCTCTCTCT	18
Locus_9557_Transcript_13/16_Confidence_0.576_Length_1988	REVERSE	55.35	52.63	GGGGTTACAGAGAAGCAGA						
Locus_9595_Transcript_11/19_Confidence_0.580_Length_1244	FORWARD	55.07	42.86	CACTTGAAGACATACCTTTCG	155	418	418	TA	TATATATATATATATATA	18
Locus_9595_Transcript_11/19_Confidence_0.580_Length_1244	REVERSE	54.62	47.62	TAGAGAGGGATAGGGGATAGA						
Locus_9666_Transcript_26/38_Confidence_0.460_Length_1864	FORWARD	54.93	33.33	AAGCCAAATATCGTTTTCTCT	156	422	422	GA	GAGAGAGAGAGAGAGAGAGAGA	22
Locus_9666_Transcript_26/38_Confidence_0.460_Length_1864	REVERSE	54.67	33.33	TGTCAACGATTTTCTCTTTTC						
Locus_9666_Transcript_26/38_Confidence_0.460_Length_1864	FORWARD	54.67	33.33	GAAAAGAGAAAATCGTTGACA	114	422	422	AG	AGAGAGAGAGAGAGAGAGAGAG	22
Locus_9666_Transcript_26/38_Confidence_0.460_Length_1864	REVERSE	54.52	47.62	TCTCTCTCTAGCTGTCTTCCA						
Locus_9666_Transcript_26/38_Confidence_0.460_Length_1864	FORWARD	54.93	33.33	AAGCCAAATATCGTTTTCTCT	157	419	419	AG	AGAGAGAGAGAGAGAGAG	18
Locus_9666_Transcript_26/38_Confidence_0.460_Length_1864	REVERSE	53.88	33.33	CTGTCAACGATTTTCTCTTTT						
Locus_9854_Transcript_10/22_Confidence_0.673_Length_2868	FORWARD	55.05	38.1	GCTTTGTTTATACCTCCCATT	165	422	422	TC	TCTCTCTCTCTCTCTCTCTCTC	22

Locus_9854_Transcript_10/22_Confidence_0.673_Length_2868	REVERSE	55.2	42.86	ATAGGGGATTGGTATCAGAGA						
Locus_9854_Transcript_10/22_Confidence_0.673_Length_2868	FORWARD	55.08	47.62	CCCATTGATCTCTCTCTCTCT	153	422	422	CT	CTCTCTCTCTCTCTCTCTCTCT	22
Locus_9854_Transcript_10/22_Confidence_0.673_Length_2868	REVERSE	55.09	47.62	GGTATAGGGGATTGGTATCAG						
Locus_9854_Transcript_10/22_Confidence_0.673_Length_2868	FORWARD	55.08	47.62	CCCATTGATCTCTCTCTCTCT	157	422	422	CT	CTCTCTCTCTCTCTCTCTCTCTCT	24
Locus_9854_Transcript_10/22_Confidence_0.673_Length_2868	REVERSE	55.09	42.86	AGATGGTATAGGGGATTGGTA						
Locus_9903_Transcript_11/17_Confidence_0.555_Length_2770	FORWARD	55.08	47.62	CTCTCTGTGTCCAGTTTCTTG	143	423	423	CT	CTCTCTCTCTCTCTCTCTCTCT	22
Locus_9903_Transcript_11/17_Confidence_0.555_Length_2770	REVERSE	56	57.14	AGAGAGAGAGAGGAGGGAGAG						
Locus_9903_Transcript_11/17_Confidence_0.555_Length_2770	FORWARD	54.53	47.62	GGATTTGCTCTCTCTCTCTCT	157	423	423	CT	CTCTCTCTCTCTCTCTCTCTCTCT	24
Locus_9903_Transcript_11/17_Confidence_0.555_Length_2770	REVERSE	55.09	42.86	AGATGGTATAGGGGATTGGTA						
Locus_10178_Transcript_9/12_Confidence_0.702_Length_1760	FORWARD	55.08	38.1	TTCATGCTCTCAGATTGAACT	156	422	422	CT	CTCTCTCTCTCTCTCTCTCT	20
Locus_10178_Transcript_9/12_Confidence_0.702_Length_1760	REVERSE	55.09	47.62	GGTATAGGGGATTGGTATCAG						
Locus_10178_Transcript_9/12_Confidence_0.702_Length_1760	FORWARD	55.08	38.1	TTCATGCTCTCAGATTGAACT	160	422	422	CT	CTCTCTCTCTCTCTCTCTCTCTCT	24
Locus_10178_Transcript_9/12_Confidence_0.702_Length_1760	REVERSE	55.09	42.86	AGATGGTATAGGGGATTGGTA						
Locus_10469_Transcript_14/30_Confidence_0.474_Length_903	FORWARD	54.03	57.14	CCTCTCTCTCTCTCTCTCTCC	135	424	424	CT	CTCTCTCTCTCTCTCTCTCTCTCT	24
Locus_10469_Transcript_14/30_Confidence_0.474_Length_903	REVERSE	55.5	47.62	GAGAGAGGAGCTGGTTTTCTA						
Locus_10523_Transcript_3/12_Confidence_0.694_Length_2224	FORWARD	55.31	42.86	TGACCAATGAAGTGTCCTTAG	161	418	418	TA	TATATATATATATATATA	18
Locus_10523_Transcript_3/12_Confidence_0.694_Length_2224	REVERSE	55.65	47.62	CGCTGGCTCTATTCTCTCTAT						
Locus_10568_Transcript_20/23_Confidence_0.608_Length_702	FORWARD	52.74	36.36	TCCAATATTAGAGGATTGAGTC	147	426	426	TA	TATATATATATATATATATATATATA	26
Locus_10568_Transcript_20/23_Confidence_0.608_Length_702	REVERSE	54.25	42.86	AGGGTAAATAGGGTTCTTCTG						
Locus_10576_Transcript_22/24_Confidence_0.639_Length_1307	FORWARD	54.72	47.37	GATGACTTTGGGACCTGAT	166	427	427	GA	GAGAGAGAGAGAGAGAGAGAGAGAGA	26
Locus_10576_Transcript_22/24_Confidence_0.639_Length_1307	REVERSE	55.36	57.14	CGCTCTCTCTCTCTCTCTCTC						
Locus_10576_Transcript_22/24_Confidence_0.639_Length_1307	FORWARD	56.01	52.38	GGAGCTTGTTGAGAGAGAGAG	151	427	427	GA	GAGAGAGAGAGAGAGAGAGAGA	22
Locus_10576_Transcript_22/24_Confidence_0.639_Length_1307	REVERSE	53.88	33.33	CTGTCAACGATTTTCTCTTTT						
Locus_10576_Transcript_22/24_Confidence_0.639_Length_1307	FORWARD	54.94	55	GTCGGTCACAAGAGAGAGAG	133	427	427	AG	AGAGAGAGAGAGAGAGAGAGAG	22
Locus_10576_Transcript_22/24_Confidence_0.639_Length_1307	REVERSE	54.91	38.1	CCCATTTATAACCACTTCCTT						
Locus_10576_Transcript_22/24_Confidence_0.639_Length_1307	FORWARD	56.01	52.38	GGAGCTTGTTGAGAGAGAGAG	151	419	419	GA	GAGAGAGAGAGAGAGAGAGAGA	22
Locus_10576_Transcript_22/24_Confidence_0.639_Length_1307	REVERSE	53.88	33.33	CTGTCAACGATTTTCTCTTTT						
Locus_10578_Transcript_8/9_Confidence_0.514_Length_273	FORWARD	54.62	42.86	CCGTTCAGATCAACATATACC	147	273	273	GA	GAGAGAGAGAGAGAGAGA	18
Locus_10578_Transcript_8/9_Confidence_0.514_Length_273	REVERSE	55.02	47.62	CCGGTGTACAGTAAAGTCTTG						
Locus_10606_Transcript_8/16_Confidence_0.579_Length_1578	FORWARD	55.23	33.33	TGCAGATCAAAGAATTAGCAT	159	425	425	CT	CTCTCTCTCTCTCTCTCTCTCTCT	24
Locus_10606_Transcript_8/16_Confidence_0.579_Length_1578	REVERSE	54.62	52.17	AGAGAGAGAGAGAGAGAGAGAGG						
Locus_10610_Transcript_15/24_Confidence_0.631_Length_1898	FORWARD	55.24	40.91	TCCTACTTAAGCTCAACTTCGT	135	421	421	GA	GAGAGAGAGAGAGAGAGAGA	20
Locus_10610_Transcript_15/24_Confidence_0.631_Length_1898	REVERSE	54.95	50	GTGCTTGGAGTTGAGAAGAC						
Locus_10629_Transcript_4/18_Confidence_0.607_Length_2251	FORWARD	54.88	38.1	TCATCTTCTCCACATTATGCT	164	419	419	CT	CTCTCTCTCTCTCTCTCT	18
Locus_10629_Transcript_4/18_Confidence_0.607_Length_2251	REVERSE	55.23	33.33	AAATCCTCACAATCTCCAAAT						
Locus_10748_Transcript_33/42_Confidence_0.589_Length_2562	FORWARD	55.4	42.86	CGATGATCACTAGTTTGGAGA	204	424	424	TC	TCTCTCTCTCTCTCTCTCTCTC	22
Locus_10748_Transcript_33/42_Confidence_0.589_Length_2562	REVERSE	55.2	42.86	ATAGGGGATTGGTATCAGAGA						
Locus_10748_Transcript_33/42_Confidence_0.589_Length_2562	FORWARD	56.16	52.38	GGGATTCCTTCTCTCTCTCTC	156	424	424	CT	CTCTCTCTCTCTCTCTCTCTCTCT	24
Locus_10748_Transcript_33/42_Confidence_0.589_Length_2562	REVERSE	54.85	38.1	CTTCTCCAGCTTTCTTTTTCT						
Locus_10835_Transcript_19/26_Confidence_0.210_Length_682	FORWARD	55.5	47.62	GAGAGAGGAGCTGGTTTTCTA	150	424	424	AG	AGAGAGAGAGAGAGAGAGAGAGAG	24
Locus_10835_Transcript_19/26_Confidence_0.210_Length_682	REVERSE	55.31	38.1	GCCAAATCTACGCCTATTTAT						
Locus_10835_Transcript_19/26_Confidence_0.210_Length_682	FORWARD	54.67	33.33	GAAAAGAGAAAATCGTTGACA	149	424	424	AG	AGAGAGAGAGAGAGAGAGAGAG	22
Locus_10835_Transcript_19/26_Confidence_0.210_Length_682	REVERSE	55.35	38.1	TATACAACATCTGCATCAGCA						
Locus_11095_Transcript_1/11_Confidence_0.307_Length_1224	FORWARD	54.64	47.62	CCGAGTTTAGAGAGTGTGGTA	155	426	426	AT	ATATATATATATATATATATATATAT	26
Locus_11095_Transcript_1/11_Confidence_0.307_Length_1224	REVERSE	54.58	38.1	GCTCGAAAGAGGAAATAGAAT						

Locus_11220_Transcript_1/23_Confidence_0.573_Length_858	FORWARD	54.99	38.1	ATTAGAAGTCTCCGTTGCTTT	149	420	420	CT	CTCTCTCTCTCTCTCTCTCT	20
Locus_11220_Transcript_1/23_Confidence_0.573_Length_858	REVERSE	55.06	42.86	CTATCATTGCAGGAGAAGATG						
Locus_11236_Transcript_4/6_Confidence_0.720_Length_1185	FORWARD	55.05	33.33	GATCGTGATTTTGATGTTGTT	157	418	418	CT	CTCTCTCTCTCTCTCTCT	18
Locus_11236_Transcript_4/6_Confidence_0.720_Length_1185	REVERSE	55.03	47.62	CCGGAGACATTAGAGACCTAT						
Locus_11288_Transcript_14/29_Confidence_0.484_Length_2142	FORWARD	55	45.45	CTTGCCTATATACACACACCAG	154	423	423	CT	CTCTCTCTCTCTCTCTCTCTCT	22
Locus_11288_Transcript_14/29_Confidence_0.484_Length_2142	REVERSE	54.14	38.1	CAGCTTGTAACCTTTTTAGGA						
Locus_11288_Transcript_14/29_Confidence_0.484_Length_2142	FORWARD	54.98	42.86	ACCCATCGAATTTACTCTCTC	157	423	423	CT	CTCTCTCTCTCTCTCTCTCTCTCT	24
Locus_11288_Transcript_14/29_Confidence_0.484_Length_2142	REVERSE	55.09	42.86	AGATGGTATAGGGGATTGGTA						
Locus_11288_Transcript_14/29_Confidence_0.484_Length_2142	FORWARD	55.09	42.86	TACCAATCCCCTATACCATCT	166	424	424	TC	TCTCTCTCTCTCTCTCTCTCTC	22
Locus_11288_Transcript_14/29_Confidence_0.484_Length_2142	REVERSE	54.47	38.1	TTATGGAAGAAGAGGAATGTG						
Locus_11288_Transcript_14/29_Confidence_0.484_Length_2142	FORWARD	55.24	52.38	TCTCCTCGCTCTCTACTCTCT	153	423	423	CT	CTCTCTCTCTCTCTCTCTCTCTCT	24
Locus_11288_Transcript_14/29_Confidence_0.484_Length_2142	REVERSE	55.5	47.62	GAGAGAGGAGCTGGTTTTCTA						
Locus_11288_Transcript_14/29_Confidence_0.484_Length_2142	FORWARD	54.85	38.1	CGCTCTTACATTCCTTCAATA	156	423	423	TC	TCTCTCTCTCTCTCTCTCTCTC	22
Locus_11288_Transcript_14/29_Confidence_0.484_Length_2142	REVERSE	55.07	42.86	AGGAGATGATTTAGGATCGAG						
Locus_11344_Transcript_14/21_Confidence_0.531_Length_1825	FORWARD	54.59	42.86	CGCTAGCATCTTAATTCTCAC	118	424	424	AG	AGAGAGAGAGAGAGAGAGAGAGAG	24
Locus_11344_Transcript_14/21_Confidence_0.531_Length_1825	REVERSE	54.67	33.33	TGTCAACGATTTTCTCTTTTC						
Locus_11344_Transcript_14/21_Confidence_0.531_Length_1825	FORWARD	54.59	42.86	CGCTAGCATCTTAATTCTCAC	189	424	424	AG	AGAGAGAGAGAGAGAGAG	18
Locus_11344_Transcript_14/21_Confidence_0.531_Length_1825	REVERSE	54.81	47.62	CAAGAGATCCCTCTCTTTCTC						
Locus_11344_Transcript_14/21_Confidence_0.531_Length_1825	FORWARD	54.67	33.33	GAAAAGAGAAAATCGTTGACA	148	424	424	AG	AGAGAGAGAGAGAGAGAG	18
Locus_11344_Transcript_14/21_Confidence_0.531_Length_1825	REVERSE	54.85	38.1	CGATAGAAAGAATGCTGGTAA						
Locus_11427_Transcript_4/11_Confidence_0.667_Length_526	FORWARD	54.91	42.86	CATAACCGAGTAGAGCTTTGA	145	421	421	AG	AGAGAGAGAGAGAGAGAGAG	20
Locus_11427_Transcript_4/11_Confidence_0.667_Length_526	REVERSE	55.14	50	CTTCTGTTCCTTGCTCTGTC						
Locus_11465_Transcript_7/12_Confidence_0.811_Length_2205	FORWARD	54.51	47.62	GCACACACATTCTCTCTCTCT	179	423	423	CT	CTCTCTCTCTCTCTCTCTCTCT	22
Locus_11465_Transcript_7/12_Confidence_0.811_Length_2205	REVERSE	55.09	47.62	GGTATAGGGGATTGGTATCAG						
Locus_11465_Transcript_7/12_Confidence_0.811_Length_2205	FORWARD	54.92	50	TTGCTCTCTCTCTCTCTCTCTC	153	423	423	CT	CTCTCTCTCTCTCTCTCTCTCTCT	24
Locus_11465_Transcript_7/12_Confidence_0.811_Length_2205	REVERSE	55.09	42.86	AGATGGTATAGGGGATTGGTA						
Locus_11469_Transcript_29/30_Confidence_0.612_Length_1367	FORWARD	54.13	38.1	ATGAGAGATCAATCGTCAATC	155	424	424	GA	GAGAGAGAGAGAGAGAGAGAGAGA	24
Locus_11469_Transcript_29/30_Confidence_0.612_Length_1367	REVERSE	54.77	47.62	CCGGTCCTAAATTAGTCTCTC						
Locus_11469_Transcript_29/30_Confidence_0.612_Length_1367	FORWARD	54.93	50	TTCGAGAGAGAGAGAGAGAGAG	157	424	424	AG	AGAGAGAGAGAGAGAGAG	18
Locus_11469_Transcript_29/30_Confidence_0.612_Length_1367	REVERSE	54.83	38.1	TAATATTCTTCTACGGCATCG						
Locus_11581_Transcript_15/16_Confidence_0.567_Length_1002	FORWARD	54.22	38.1	CTCAAAGTTGCCTCTTTTCTA	157	419	419	CT	CTCTCTCTCTCTCTCTCT	18
Locus_11581_Transcript_15/16_Confidence_0.567_Length_1002	REVERSE	55.04	47.62	GCCACTCTTCTTTCTTCTCTC						
Locus_11651_Transcript_1/9_Confidence_0.460_Length_310	FORWARD	52.3	30	GAAAGCAAATTATTGAAGGA	143	310	310	GA	GAGAGAGAGAGAGAGAGAGAGA	22
Locus_11651_Transcript_1/9_Confidence_0.460_Length_310	REVERSE	54.73	42.86	TCACCGCTAGGATTATAACTG						
Locus_11651_Transcript_1/9_Confidence_0.460_Length_310	FORWARD	54.17	38.1	TGAAGGAATGTAAAGGAGAGA	130	310	310	AG	AGAGAGAGAGAGAGAGAG	18
Locus_11651_Transcript_1/9_Confidence_0.460_Length_310	REVERSE	54.73	42.86	TCACCGCTAGGATTATAACTG						
Locus_11663_Transcript_1/11_Confidence_0.523_Length_515	FORWARD	55.5	47.62	GAGAGAGGAGCTGGTTTTCTA	143	424	424	AG	AGAGAGAGAGAGAGAGAGAGAGAG	24
Locus_11663_Transcript_1/11_Confidence_0.523_Length_515	REVERSE	55.04	42.86	CTGATTTCTATCCCGGTACTT						
Locus_11695_Transcript_5/22_Confidence_0.589_Length_711	FORWARD	55.15	33.33	ATATTTGCCCTTATCTTTTGG	158	423	423	AG	AGAGAGAGAGAGAGAGAGAGAG	22
Locus_11695_Transcript_5/22_Confidence_0.589_Length_711	REVERSE	54.48	52.38	TCCTTCTCCTCTCTCTCTCTC						
Locus_12228_Transcript_3/12_Confidence_0.444_Length_433	FORWARD	55.09	31.82	GAAAGCAAATTATTGAAGGAAG	149	423	423	GA	GAGAGAGAGAGAGAGAGAGAGA	22
Locus_12228_Transcript_3/12_Confidence_0.444_Length_433	REVERSE	55.69	50	GCACAGTAAGCTGACTCTCTCT						
Locus_12228_Transcript_3/12_Confidence_0.444_Length_433	FORWARD	54.6	52.38	AGAGAGAGAGGAGAGAACTCG	142	423	423	AG	AGAGAGAGAGAGAGAGAG	18
Locus_12228_Transcript_3/12_Confidence_0.444_Length_433	REVERSE	54.26	38.1	TCTTTCCTTCTGAAGATCTGA						
Locus_12228_Transcript_3/12_Confidence_0.444_Length_433	FORWARD	54.67	33.33	GAAAAGAGAAAATCGTTGACA	150	423	423	AG	AGAGAGAGAGAGAGAGAGAGAG	22

Locus_12228_Transcript_3/12_Confidence_0.444_Length_433	REVERSE	55.34	47.62	ACCTCATAGTAGTCCCCTGAA						
Locus_12231_Transcript_19/22_Confidence_0.623_Length_2627	FORWARD	54.27	36.36	TGGAGTTTCAGATGTTAGAGAA	148	419	419	AG	AGAGAGAGAGAGAGAGAG	18
Locus_12231_Transcript_19/22_Confidence_0.623_Length_2627	REVERSE	54.44	38.1	GCAGGATAATGGAATAACTCA						
Locus_12278_Transcript_6/14_Confidence_0.591_Length_1324	FORWARD	55.24	38.1	TGGATCAAAGATATCATCTCG	190	424	424	CT	CTCTCTCTCTCTCTCTCTCTCT	22
Locus_12278_Transcript_6/14_Confidence_0.591_Length_1324	REVERSE	55.2	42.86	ATAGGGGATTGGTATCAGAGA						
Locus_12278_Transcript_6/14_Confidence_0.591_Length_1324	FORWARD	55.24	38.1	TGGATCAAAGATATCATCTCG	197	424	424	CT	CTCTCTCTCTCTCTCTCTCTCTCT	24
Locus_12278_Transcript_6/14_Confidence_0.591_Length_1324	REVERSE	55.09	42.86	AGATGGTATAGGGGATTGGTA						
Locus_12348_Transcript_27/41_Confidence_0.534_Length_1122	FORWARD	55.17	38.1	GATACGGTATAAAAGGCGAAT	146	425	425	AG	AGAGAGAGAGAGAGAGAGAGAGAG	24
Locus_12348_Transcript_27/41_Confidence_0.534_Length_1122	REVERSE	55.1	31.82	TCAACGATTTTCTCTTTTCTCT						
Locus_12348_Transcript_27/41_Confidence_0.534_Length_1122	FORWARD	56.36	47.62	CGGAGAAATCAGAGAGAGAGA	162	425	425	AG	AGAGAGAGAGAGAGAGAGAGAG	22
Locus_12348_Transcript_27/41_Confidence_0.534_Length_1122	REVERSE	54.92	42.86	ATACCATATCCGCTAAGCTCT						
Locus_12488_Transcript_3/4_Confidence_0.875_Length_448	FORWARD	54.68	38.1	GGATGATTTGAGGGAGATAAT	156	420	420	CT	CTCTCTCTCTCTCTCTCTCTCT	22
Locus_12488_Transcript_3/4_Confidence_0.875_Length_448	REVERSE	54.92	50	GCAAGAGAGAGAGAGAGAGAGA						
Locus_12488_Transcript_3/4_Confidence_0.875_Length_448	FORWARD	55.03	33.33	ATAATTGGGAGTTTTCCTTTG	147	420	420	CT	CTCTCTCTCTCTCTCTCTCT	20
Locus_12488_Transcript_3/4_Confidence_0.875_Length_448	REVERSE	64.1	63.16	CTTCCGCACCGGCAGTTAG						
Locus_12756_Transcript_12/13_Confidence_0.725_Length_879	FORWARD	54.78	33.33	TTTGTTTATACCTCCCATTGA	163	422	422	TC	TCTCTCTCTCTCTCTCTCTCTC	22
Locus_12756_Transcript_12/13_Confidence_0.725_Length_879	REVERSE	55.2	42.86	ATAGGGGATTGGTATCAGAGA						
Locus_12756_Transcript_12/13_Confidence_0.725_Length_879	FORWARD	55.08	47.62	CCCATTGATCTCTCTCTCTCT	157	422	422	CT	CTCTCTCTCTCTCTCTCTCTCTCT	24
Locus_12756_Transcript_12/13_Confidence_0.725_Length_879	REVERSE	55.09	42.86	AGATGGTATAGGGGATTGGTA						
Locus_12810_Transcript_6/7_Confidence_0.674_Length_1939	FORWARD	54.89	42.86	CCGACTACACTTGATTCAAAC	148	420	420	CT	CTCTCTCTCTCTCTCTCTCT	20
Locus_12810_Transcript_6/7_Confidence_0.674_Length_1939	REVERSE	54.9	45	CGATCTCCAACAGTTTCTTC						
Locus_13064_Transcript_2/5_Confidence_0.455_Length_254	FORWARD	55.1	47.62	CGAATAGTTAGACACGTGAGG	110	254	254	AT	ATATATATATATATATATAT	20
Locus_13064_Transcript_2/5_Confidence_0.455_Length_254	REVERSE	55.44	45	CACACACACACGCATATTCT						
Locus_13139_Transcript_2/3_Confidence_0.600_Length_354	FORWARD	55.06	38.1	TCCTTCATGTAAACATTGGAC	143	354	354	TC	TCTCTCTCTCTCTCTCTCTC	20
Locus_13139_Transcript_2/3_Confidence_0.600_Length_354	REVERSE	55.56	42.86	TACCAAGTTTTGAGACAGACG						
Locus_13166_Transcript_2/5_Confidence_0.882_Length_621	FORWARD	54.85	38.1	ATGCTGAAACTCTGATGTTGT	147	425	425	GA	GAGAGAGAGAGAGAGAGAGAGAGA	24
Locus_13166_Transcript_2/5_Confidence_0.882_Length_621	REVERSE	55.36	57.14	CGCTCTCTCTCTCTCTCTCTC						
Locus_13166_Transcript_2/5_Confidence_0.882_Length_621	FORWARD	55.02	57.14	GACGGAGAGAGAGAGAGAGAG	147	425	425	AG	AGAGAGAGAGAGAGAGAGAGAGAG	24
Locus_13166_Transcript_2/5_Confidence_0.882_Length_621	REVERSE	53.88	33.33	CTGTCAACGATTTTCTCTTTT						
Locus_13337_Transcript_1/2_Confidence_0.750_Length_596	FORWARD	54.7	38.1	GGAGCTTTCTTTATGTGTCAA	170	421	421	AT	ATATATATATATATATATAT	20
Locus_13337_Transcript_1/2_Confidence_0.750_Length_596	REVERSE	55.02	47.62	CGATGTCGTATAGTCCTTCTG						
Locus_13387_Transcript_11/12_Confidence_0.618_Length_1220	FORWARD	55.2	42.86	CGCTTCCTTCTTTCTATTCTC	168	418	418	TC	TCTCTCTCTCTCTCTCTC	18
Locus_13387_Transcript_11/12_Confidence_0.618_Length_1220	REVERSE	56.31	38.1	TGATCTTAAAGACACCGGAAT						
Locus_13609_Transcript_8/11_Confidence_0.610_Length_536	FORWARD	56.09	42.11	GAATTCACACGAGCCAAAT	153	423	423	CT	CTCTCTCTCTCTCTCTCTCTCT	22
Locus_13609_Transcript_8/11_Confidence_0.610_Length_536	REVERSE	55.09	42.86	AGATGGTATAGGGGATTGGTA						
Locus_13778_Transcript_3/5_Confidence_0.722_Length_371	FORWARD	55.04	31.82	AAACTGATGGATGAAATTAAGG	149	371	371	AG	AGAGAGAGAGAGAGAGAG	18
Locus_13778_Transcript_3/5_Confidence_0.722_Length_371	REVERSE	55.32	47.62	GCATTGAACCTCTCTCTCTCT						
Locus_13822_Transcript_9/14_Confidence_0.295_Length_323	FORWARD	55.22	42.86	AGCATCTCTCCAAAACTAACC	151	323	323	AG	AGAGAGAGAGAGAGAGAGAGAG	22
Locus_13822_Transcript_9/14_Confidence_0.295_Length_323	REVERSE	54.29	47.62	CCTCCCTACAATCTTCTCTCT						
Locus_13822_Transcript_9/14_Confidence_0.295_Length_323	FORWARD	55.61	52.38	AGCCGTTAGACAGAGAGAGAG	152	323	323	AG	AGAGAGAGAGAGAGAGAG	18
Locus_13822_Transcript_9/14_Confidence_0.295_Length_323	REVERSE	54.98	42.86	TGATCTGTCGTCTACCCTAAA						
Locus_14154_Transcript_20/27_Confidence_0.583_Length_880	FORWARD	54.75	38.1	TGACATTGCAATATGTGTAGC	151	424	424	GT	GTGTGTGTGTGTGTGTGTGTGTGT	24
Locus_14154_Transcript_20/27_Confidence_0.583_Length_880	REVERSE	55.3	38.1	CCTCTCCAATTTTTCCTCTAA						
Locus_14543_Transcript_1/1_Confidence_0.996_Length_2557	FORWARD	54.97	38.1	ATTGGAGATGAGCACAGTTTA	145	425	425	AG	AGAGAGAGAGAGAGAGAGAGAGAG	24
Locus_14543_Transcript_1/1_Confidence_0.996_Length_2557	REVERSE	55.36	57.14	CGCTCTCTCTCTCTCTCTCTC						

Locus_14543_Transcript_1/1_Confidence_0.996_Length_2557	FORWARD	55	33.33	GAACATGATTTGAATTGGGTA	159	425	425	AG	AGAGAGAGAGAGAGAGAGAGAGAG	24
Locus_14543_Transcript_1/1_Confidence_0.996_Length_2557	REVERSE	55.36	57.14	CGCTCTCTCTCTCTCTCTCTC						
Locus_14547_Transcript_1/1_Confidence_0.971_Length_443	FORWARD	54.97	52.38	GAGAGAGAGAGAGTGGTGGAT	145	419	419	GA	GAGAGAGAGAGAGAGAGA	18
Locus_14547_Transcript_1/1_Confidence_0.971_Length_443	REVERSE	54.99	33.33	ACTCAAAATTGTCCTGCAATA						
Locus_15091_Transcript_1/1_Confidence_1.000_Length_545	FORWARD	54.23	50	CAGAAGTAGAACCAGCTTCC	153	426	426	TC	TCTCTCTCTCTCTCTCTCTCTCTCTC	26
Locus_15091_Transcript_1/1_Confidence_1.000_Length_545	REVERSE	55.09	42.86	AGATGGTATAGGGGATTGGTA						
Locus_15140_Transcript_1/1_Confidence_1.000_Length_411	FORWARD	55.66	42.86	GGCCTTGTGTTTCTATTTAGG	158	411	411	AT	ATATATATATATATATATATAT	22
Locus_15140_Transcript_1/1_Confidence_1.000_Length_411	REVERSE	55.35	42.86	CAAATGCTGGAAGCTATAGTG						
Locus_15223_Transcript_1/1_Confidence_1.000_Length_690	FORWARD	55.36	33.33	TAAAGGCTTTGAAAGACAACA	142	422	422	AG	AGAGAGAGAGAGAGAGAGAGAG	22
Locus_15223_Transcript_1/1_Confidence_1.000_Length_690	REVERSE	57.98	55.56	CTTTCATCCCTCCCCTTG						
Locus_15225_Transcript_1/1_Confidence_1.000_Length_437	FORWARD	55.5	47.62	TAGAAAACCAGCTCCTCTCTC	147	424	424	CT	CTCTCTCTCTCTCTCTCTCTCTCT	24
Locus_15225_Transcript_1/1_Confidence_1.000_Length_437	REVERSE	54.83	38.1	AAAAGATGGTATAGGGGATTG						
Locus_15226_Transcript_1/1_Confidence_1.000_Length_337	FORWARD	54.99	33.33	ACTCAAAATTGTCCTGCAATA	155	337	337	CT	CTCTCTCTCTCTCTCTCTCTCTCT	24
Locus_15226_Transcript_1/1_Confidence_1.000_Length_337	REVERSE	54.69	47.62	TGTAAGAGCGAGAGAGAGAGA						
Locus_15226_Transcript_1/1_Confidence_1.000_Length_337	FORWARD	54.97	52.38	AGATCCACCACTCTCTCTCTC	150	337	337	CT	CTCTCTCTCTCTCTCTCTCTCTCTCT	26
Locus_15226_Transcript_1/1_Confidence_1.000_Length_337	REVERSE	54.93	33.33	GTGGATGAAAGCAAATTATTG						
Locus_15227_Transcript_1/1_Confidence_1.000_Length_925	FORWARD	55.11	38.1	AACCTTTCTGAAACCTGAAAC	155	423	423	TC	TCTCTCTCTCTCTCTCTCTCTC	22
Locus_15227_Transcript_1/1_Confidence_1.000_Length_925	REVERSE	55.07	52.38	TAGAGAGAGAGAGGAGCTGGT						
Locus_15227_Transcript_1/1_Confidence_1.000_Length_925	FORWARD	54.35	47.62	GAGGCCATAATCTCTCTCTCT	148	423	423	TC	TCTCTCTCTCTCTCTCTCTCTC	22
Locus_15227_Transcript_1/1_Confidence_1.000_Length_925	REVERSE	54.83	38.1	AAAAGATGGTATAGGGGATTG						
Locus_15228_Transcript_1/1_Confidence_1.000_Length_921	FORWARD	55.11	38.1	AACCTTTCTGAAACCTGAAAC	153	423	423	TC	TCTCTCTCTCTCTCTCTCTCTC	22
Locus_15228_Transcript_1/1_Confidence_1.000_Length_921	REVERSE	55.5	47.62	GAGAGAGGAGCTGGTTTTCTA						
Locus_15228_Transcript_1/1_Confidence_1.000_Length_921	FORWARD	54.35	47.62	GGCCATAATCTCTCTCTCTCT	173	423	423	TC	TCTCTCTCTCTCTCTCTCTCTC	22
Locus_15228_Transcript_1/1_Confidence_1.000_Length_921	REVERSE	55.21	38.1	ATAGGGGATTGGTATCAGAAA						
Locus_15231_Transcript_1/1_Confidence_1.000_Length_390	FORWARD	54.14	38.1	TCCTAAAAAGGTTACAAGCTG	145	390	390	CT	CTCTCTCTCTCTCTCTCTCTCTCT	24
Locus_15231_Transcript_1/1_Confidence_1.000_Length_390	REVERSE	55.54	47.62	AGGAATGTAAGAGCGAGAGAG						
Locus_15232_Transcript_1/1_Confidence_1.000_Length_421	FORWARD	54.14	38.1	TCCTAAAAAGGTTACAAGCTG	145	421	421	CT	CTCTCTCTCTCTCTCTCTCTCTCT	24
Locus_15232_Transcript_1/1_Confidence_1.000_Length_421	REVERSE	55.54	47.62	AGGAATGTAAGAGCGAGAGAG						
Locus_15233_Transcript_1/1_Confidence_1.000_Length_382	FORWARD	55.11	38.1	AACCTTTCTGAAACCTGAAAC	155	382	382	TC	TCTCTCTCTCTCTCTCTCTCTC	22
Locus_15233_Transcript_1/1_Confidence_1.000_Length_382	REVERSE	55.07	52.38	TAGAGAGAGAGAGGAGCTGGT						
Locus_15233_Transcript_1/1_Confidence_1.000_Length_382	FORWARD	54.35	47.62	GAGGCCATAATCTCTCTCTCT	148	382	382	TC	TCTCTCTCTCTCTCTCTCTCTC	22
Locus_15233_Transcript_1/1_Confidence_1.000_Length_382	REVERSE	54.83	38.1	AAAAGATGGTATAGGGGATTG						
Locus_15234_Transcript_1/1_Confidence_1.000_Length_415	FORWARD	55.11	38.1	AACCTTTCTGAAACCTGAAAC	155	415	415	TC	TCTCTCTCTCTCTCTCTCTCTC	22
Locus_15234_Transcript_1/1_Confidence_1.000_Length_415	REVERSE	55.07	52.38	TAGAGAGAGAGAGGAGCTGGT						
Locus_15234_Transcript_1/1_Confidence_1.000_Length_415	FORWARD	54.35	47.62	GAGGCCATAATCTCTCTCTCT	148	415	415	TC	TCTCTCTCTCTCTCTCTCTCTC	22
Locus_15234_Transcript_1/1_Confidence_1.000_Length_415	REVERSE	54.83	38.1	AAAAGATGGTATAGGGGATTG						
Locus_15235_Transcript_1/1_Confidence_1.000_Length_415	FORWARD	55.11	38.1	AACCTTTCTGAAACCTGAAAC	153	415	415	TC	TCTCTCTCTCTCTCTCTCTCTC	22
Locus_15235_Transcript_1/1_Confidence_1.000_Length_415	REVERSE	55.5	47.62	GAGAGAGGAGCTGGTTTTCTA						
Locus_15235_Transcript_1/1_Confidence_1.000_Length_415	FORWARD	54.35	47.62	GGCCATAATCTCTCTCTCTCT	173	415	415	TC	TCTCTCTCTCTCTCTCTCTCTC	22
Locus_15235_Transcript_1/1_Confidence_1.000_Length_415	REVERSE	55.21	38.1	ATAGGGGATTGGTATCAGAAA						
Locus_15236_Transcript_1/1_Confidence_1.000_Length_448	FORWARD	55.11	38.1	AACCTTTCTGAAACCTGAAAC	153	423	423	TC	TCTCTCTCTCTCTCTCTCTCTC	22
Locus_15236_Transcript_1/1_Confidence_1.000_Length_448	REVERSE	55.5	47.62	GAGAGAGGAGCTGGTTTTCTA						
Locus_15236_Transcript_1/1_Confidence_1.000_Length_448	FORWARD	54.35	47.62	GGCCATAATCTCTCTCTCTCT	173	423	423	TC	TCTCTCTCTCTCTCTCTCTCTC	22
Locus_15236_Transcript_1/1_Confidence_1.000_Length_448	REVERSE	55.21	38.1	ATAGGGGATTGGTATCAGAAA						
Locus_15237_Transcript_1/1_Confidence_1.000_Length_1380	FORWARD	54.14	38.1	TCCTAAAAAGGTTACAAGCTG	141	424	424	CT	CTCTCTCTCTCTCTCTCTCTCTCT	24

Locus_15237_Transcript_1/1_Confidence_1.000_Length_1380	REVERSE	55.54	47.62	AGGAATGTAAGAGCGAGAGAG						
Locus_15238_Transcript_1/1_Confidence_1.000_Length_619	FORWARD	55.29	40	ACGATGAAGAAACCAAACTG	167	421	421	TC	TCTCTCTCTCTCTCTCTCTC	20
Locus_15238_Transcript_1/1_Confidence_1.000_Length_619	REVERSE	54.69	47.62	TGTAAGAGCGAGAGAGAGAGA						
Locus_15238_Transcript_1/1_Confidence_1.000_Length_619	FORWARD	55.14	50	TCTCTCTCTTCTGCGCTAAC	154	421	421	CT	CTCTCTCTCTCTCTCTCTCTCTCT	24
Locus_15238_Transcript_1/1_Confidence_1.000_Length_619	REVERSE	54.99	35	AGAGCGAAAGCAAATTATTG						
Locus_15238_Transcript_1/1_Confidence_1.000_Length_619	FORWARD	55.29	40	ACGATGAAGAAACCAAACTG	167	424	424	TC	TCTCTCTCTCTCTCTCTCTC	20
Locus_15238_Transcript_1/1_Confidence_1.000_Length_619	REVERSE	54.69	47.62	TGTAAGAGCGAGAGAGAGAGA						
Locus_15239_Transcript_1/1_Confidence_1.000_Length_724	FORWARD	55.29	40	ACGATGAAGAAACCAAACTG	167	424	424	TC	TCTCTCTCTCTCTCTCTCTC	20
Locus_15239_Transcript_1/1_Confidence_1.000_Length_724	REVERSE	54.69	47.62	TGTAAGAGCGAGAGAGAGAGA						
Locus_15239_Transcript_1/1_Confidence_1.000_Length_724	FORWARD	55.14	50	TCTCTCTCTTCTGCGCTAAC	154	424	424	CT	CTCTCTCTCTCTCTCTCTCTCTCT	24
Locus_15239_Transcript_1/1_Confidence_1.000_Length_724	REVERSE	54.99	35	AGAGCGAAAGCAAATTATTG						
Locus_15308_Transcript_1/1_Confidence_1.000_Length_522	FORWARD	64.39	66.67	GTCGGTCGGTCGGTCACA	145	419	419	AG	AGAGAGAGAGAGAGAGAG	18
Locus_15308_Transcript_1/1_Confidence_1.000_Length_522	REVERSE	55.11	38.1	AACCTTTCTGAAACCTGAAAC						
Locus_15309_Transcript_1/1_Confidence_1.000_Length_550	FORWARD	64.39	66.67	GTCGGTCGGTCGGTCACA	155	419	419	AG	AGAGAGAGAGAGAGAGAG	18
Locus_15309_Transcript_1/1_Confidence_1.000_Length_550	REVERSE	54.92	38.1	AAGTCTTTGCTGGTAACCTTT						
Locus_15310_Transcript_1/1_Confidence_1.000_Length_812	FORWARD	64.39	66.67	GTCGGTCGGTCGGTCACA	145	419	419	AG	AGAGAGAGAGAGAGAGAG	18
Locus_15310_Transcript_1/1_Confidence_1.000_Length_812	REVERSE	55.11	38.1	AACCTTTCTGAAACCTGAAAC						
Locus_15311_Transcript_1/1_Confidence_1.000_Length_580	FORWARD	64.39	66.67	GTCGGTCGGTCGGTCACA	145	419	419	AG	AGAGAGAGAGAGAGAGAG	18
Locus_15311_Transcript_1/1_Confidence_1.000_Length_580	REVERSE	55.11	38.1	AACCTTTCTGAAACCTGAAAC						
Locus_15312_Transcript_1/1_Confidence_1.000_Length_349	FORWARD	64.39	66.67	GTCGGTCGGTCGGTCACA	147	349	349	AG	AGAGAGAGAGAGAGAGAG	18
Locus_15312_Transcript_1/1_Confidence_1.000_Length_349	REVERSE	54.9	42.86	AGAAGTCTTTGCTGGTAACCT						
Locus_15313_Transcript_1/1_Confidence_1.000_Length_353	FORWARD	64.39	66.67	GTCGGTCGGTCGGTCACA	147	353	353	AG	AGAGAGAGAGAGAGAGAG	18
Locus_15313_Transcript_1/1_Confidence_1.000_Length_353	REVERSE	54.9	42.86	AGAAGTCTTTGCTGGTAACCT						
Locus_15314_Transcript_1/1_Confidence_1.000_Length_308	FORWARD	54.72	47.37	GATGACTTTGGGACCTGAT	157	308	308	AG	AGAGAGAGAGAGAGAGAGAGAG	22
Locus_15314_Transcript_1/1_Confidence_1.000_Length_308	REVERSE	54.67	33.33	TGTCAACGATTTTCTCTTTTC						
Locus_15314_Transcript_1/1_Confidence_1.000_Length_308	FORWARD	54.72	47.37	GATGACTTTGGGACCTGAT	158	308	308	AG	AGAGAGAGAGAGAGAGAG	18
Locus_15314_Transcript_1/1_Confidence_1.000_Length_308	REVERSE	53.88	33.33	CTGTCAACGATTTTCTCTTTT						
Locus_15315_Transcript_1/1_Confidence_1.000_Length_367	FORWARD	54.59	50	CTTCTACTTCGAGAGAGAGAGC	109	367	367	GA	GAGAGAGAGAGAGAGAGAGAGAGA	24
Locus_15315_Transcript_1/1_Confidence_1.000_Length_367	REVERSE	54.67	33.33	TGTCAACGATTTTCTCTTTTC						
Locus_15315_Transcript_1/1_Confidence_1.000_Length_367	FORWARD	55.36	57.14	GCGAGAGAGAGAGAGAGAGAG	152	367	367	AG	AGAGAGAGAGAGAGAGAG	18
Locus_15315_Transcript_1/1_Confidence_1.000_Length_367	REVERSE	54.61	47.37	ATCTGGATCACCAGCTTCT						
Locus_15315_Transcript_1/1_Confidence_1.000_Length_367	FORWARD	54.67	33.33	GAAAAGAGAAAATCGTTGACA	152	367	367	AG	AGAGAGAGAGAGAGAGAGAGAG	22
Locus_15315_Transcript_1/1_Confidence_1.000_Length_367	REVERSE	54.69	47.37	ATGATGGTGGTGGTGTATG						
Locus_15316_Transcript_1/1_Confidence_1.000_Length_1355	FORWARD	55.05	57.89	GTATAGACGCGGAGGAGAG	180	424	424	GA	GAGAGAGAGAGAGAGAGAGAGAGA	24
Locus_15316_Transcript_1/1_Confidence_1.000_Length_1355	REVERSE	53.88	33.33	CTGTCAACGATTTTCTCTTTT						
Locus_15316_Transcript_1/1_Confidence_1.000_Length_1355	FORWARD	55.36	57.14	GCGAGAGAGAGAGAGAGAGAG	156	424	424	AG	AGAGAGAGAGAGAGAGAG	18
Locus_15316_Transcript_1/1_Confidence_1.000_Length_1355	REVERSE	54.69	50	ATCACCATGGATCACCAG						
Locus_15316_Transcript_1/1_Confidence_1.000_Length_1355	FORWARD	54.67	33.33	GAAAAGAGAAAATCGTTGACA	180	424	424	AG	AGAGAGAGAGAGAGAGAGAGAG	22
Locus_15316_Transcript_1/1_Confidence_1.000_Length_1355	REVERSE	54.97	47.37	CAGGCTGATATGCTTCGTA						
Locus_15317_Transcript_1/1_Confidence_1.000_Length_529	FORWARD	54.25	38.1	GAAAGAGAAAATCGTTGACAG	175	422	422	AG	AGAGAGAGAGAGAGAGAGAGAG	22
Locus_15317_Transcript_1/1_Confidence_1.000_Length_529	REVERSE	54.8	47.62	CTGCTCTTTTCTCTCCTCTCT						
Locus_15317_Transcript_1/1_Confidence_1.000_Length_529	FORWARD	54.67	33.33	GAAAAGAGAAAATCGTTGACA	161	422	422	AG	AGAGAGAGAGAGAGAGAGAGAG	22
Locus_15317_Transcript_1/1_Confidence_1.000_Length_529	REVERSE	54.85	42.86	GCTGATATGGTGATATGCTTC						
Locus_15318_Transcript_1/1_Confidence_1.000_Length_228	FORWARD	55.8	52.38	GAGCGAGAGAGAGAGAGAGAA	118	228	228	AG	AGAGAGAGAGAGAGAGAG	18
Locus_15318_Transcript_1/1_Confidence_1.000_Length_228	REVERSE	54.04	55.56	CCACTGCTCTTCGCTAGT						

Locus_15319_Transcript_1/1_Confidence_1.000_Length_539	FORWARD	54.94	52.38	AGCGAGAGAGAGAGAGAGAAG	153	419	419	AG	AGAGAGAGAGAGAGAGAG	18
Locus_15319_Transcript_1/1_Confidence_1.000_Length_539	REVERSE	55.16	52.63	CTCTTGTGACCGATCCTCT						
Locus_15319_Transcript_1/1_Confidence_1.000_Length_539	FORWARD	54.67	33.33	GAAAAGAGAAAATCGTTGACA	174	419	419	AG	AGAGAGAGAGAGAGAGAGAGAG	22
Locus_15319_Transcript_1/1_Confidence_1.000_Length_539	REVERSE	54.77	52.38	CTGCTCTTCTCTCCTCTCTCT						
Locus_15319_Transcript_1/1_Confidence_1.000_Length_539	FORWARD	54.67	33.33	GAAAAGAGAAAATCGTTGACA	140	419	419	AG	AGAGAGAGAGAGAGAGAGAGAG	22
Locus_15319_Transcript_1/1_Confidence_1.000_Length_539	REVERSE	56.38	45	GCTGATATGGATGCTTCGTA						
Locus_15320_Transcript_1/1_Confidence_1.000_Length_701	FORWARD	51.8	30.43	AAGTAAGTAAAAGATGGTATTCG	114	422	422	AG	AGAGAGAGAGAGAGAGAG	18
Locus_15320_Transcript_1/1_Confidence_1.000_Length_701	REVERSE	55.19	57.14	CTGCTCCTCTCTCTCTCTCTC						
Locus_15320_Transcript_1/1_Confidence_1.000_Length_701	FORWARD	54.67	33.33	GAAAAGAGAAAATCGTTGACA	187	422	422	AG	AGAGAGAGAGAGAGAGAGAGAG	22
Locus_15320_Transcript_1/1_Confidence_1.000_Length_701	REVERSE	55.18	33.33	TGTTTTTAGCCATAACCTTGA						
Locus_15321_Transcript_1/1_Confidence_1.000_Length_524	FORWARD	54.9	57.89	GGAGCAGGAGAGGTAGTTG	131	424	424	AG	AGAGAGAGAGAGAGAGAGAGAGAG	24
Locus_15321_Transcript_1/1_Confidence_1.000_Length_524	REVERSE	54.67	33.33	TGTCAACGATTTTCTCTTTTC						
Locus_15321_Transcript_1/1_Confidence_1.000_Length_524	FORWARD	54.9	57.89	GGAGCAGGAGAGGTAGTTG	132	424	424	AG	AGAGAGAGAGAGAGAGAG	18
Locus_15321_Transcript_1/1_Confidence_1.000_Length_524	REVERSE	53.88	33.33	CTGTCAACGATTTTCTCTTTT						
Locus_15322_Transcript_1/1_Confidence_1.000_Length_368	FORWARD	55.09	42.86	AGATGGTATAGGGGATTGGTA	121	368	368	AG	AGAGAGAGAGAGAGAGAG	18
Locus_15322_Transcript_1/1_Confidence_1.000_Length_368	REVERSE	54.78	38.1	AAAGATTCTGAAACGAGGACT						
Locus_15323_Transcript_1/1_Confidence_1.000_Length_518	FORWARD	55.18	38.1	GAGGTGAAAGAGAAAATCGTT	166	422	422	AG	AGAGAGAGAGAGAGAGAGAGAG	22
Locus_15323_Transcript_1/1_Confidence_1.000_Length_518	REVERSE	54.95	50	CGAGAAAGTCCCATACTGAG						
Locus_15324_Transcript_1/1_Confidence_1.000_Length_335	FORWARD	55.09	47.62	GGTATAGGGGATTGGTATCAG	151	335	335	AG	AGAGAGAGAGAGAGAGAG	18
Locus_15324_Transcript_1/1_Confidence_1.000_Length_335	REVERSE	55.08	42.86	CAGTCAAACACCCACTAATGT						
Locus_15325_Transcript_1/1_Confidence_1.000_Length_302	FORWARD	55.09	47.62	GGTATAGGGGATTGGTATCAG	151	302	302	AG	AGAGAGAGAGAGAGAGAG	18
Locus_15325_Transcript_1/1_Confidence_1.000_Length_302	REVERSE	55.08	42.86	CAGTCAAACACCCACTAATGT						
Locus_15326_Transcript_1/1_Confidence_1.000_Length_650	FORWARD	55.19	38.1	CAAAGAGAAAATCGTTGACAG	147	422	422	AG	AGAGAGAGAGAGAGAGAGAGAG	22
Locus_15326_Transcript_1/1_Confidence_1.000_Length_650	REVERSE	55.26	45	GAACACAGCATGGTTCTTCT						
Locus_15897_Transcript_1/1_Confidence_1.000_Length_804	FORWARD	55.47	38.1	GCCTCTGTTGTCACAAAATTA	163	423	423	AG	AGAGAGAGAGAGAGAGAGAGAG	22
Locus_15897_Transcript_1/1_Confidence_1.000_Length_804	REVERSE	55.15	42.86	AAAGTGTTCTAAAGGGGAGTG						
Locus_15898_Transcript_1/1_Confidence_1.000_Length_803	FORWARD	54.69	42.86	TTAGTTGGTCAGTCTGGACAT	150	421	421	GA	GAGAGAGAGAGAGAGAGAGA	20
Locus_15898_Transcript_1/1_Confidence_1.000_Length_803	REVERSE	54.59	38.1	GAAGGGGGATAATTAAAAGTG						
Locus_15899_Transcript_1/1_Confidence_1.000_Length_804	FORWARD	54.69	42.86	TTAGTTGGTCAGTCTGGACAT	151	423	423	GA	GAGAGAGAGAGAGAGAGAGAGA	22
Locus_15899_Transcript_1/1_Confidence_1.000_Length_804	REVERSE	54.59	38.1	GAAGGGGGATAATTAAAAGTG						
Locus_15900_Transcript_1/1_Confidence_1.000_Length_831	FORWARD	54.69	42.86	TTAGTTGGTCAGTCTGGACAT	163	423	423	GA	GAGAGAGAGAGAGAGAGAGAGA	22
Locus_15900_Transcript_1/1_Confidence_1.000_Length_831	REVERSE	55.15	42.86	AAAGTGTTCTAAAGGGGAGTG						
Locus_16624_Transcript_1/1_Confidence_1.000_Length_472	FORWARD	54.85	42.86	TATCACATATGACCCCTGTTC	171	419	419	AT	ATATATATATATATATAT	18
Locus_16624_Transcript_1/1_Confidence_1.000_Length_472	REVERSE	54.1	33.33	GAGATTGAATTTCATGTTGGT						
Locus_16625_Transcript_1/1_Confidence_1.000_Length_431	FORWARD	54.85	42.86	TATCACATATGACCCCTGTTC	148	425	425	AT	ATATATATATATATATATATATAT	24
Locus_16625_Transcript_1/1_Confidence_1.000_Length_431	REVERSE	55.11	33.33	GAGATTGAATTTCATGTTGGA						
Locus_16626_Transcript_1/1_Confidence_1.000_Length_397	FORWARD	55.56	38.1	TTCACTCTTCTTATGGCATTG	153	397	397	AT	ATATATATATATATATAT	18
Locus_16626_Transcript_1/1_Confidence_1.000_Length_397	REVERSE	55.11	33.33	GAGATTGAATTTCATGTTGGA						
Locus_16627_Transcript_1/1_Confidence_1.000_Length_399	FORWARD	55.56	38.1	TTCACTCTTCTTATGGCATTG	155	399	399	AT	ATATATATATATATATATAT	20
Locus_16627_Transcript_1/1_Confidence_1.000_Length_399	REVERSE	55.11	33.33	GAGATTGAATTTCATGTTGGA						
Locus_16628_Transcript_1/1_Confidence_1.000_Length_305	FORWARD	55.71	33.33	CAGGCAAAACAAGTTAAACAA	149	305	305	AT	ATATATATATATATATAT	18
Locus_16628_Transcript_1/1_Confidence_1.000_Length_305	REVERSE	55.24	38.1	CACACACAACACACACATTTT						
Locus_16644_Transcript_1/1_Confidence_1.000_Length_426	FORWARD	55.54	47.62	AGAAGGAAGATCTGCGAGTAG	151	418	418	TC	TCTCTCTCTCTCTCTCTC	18
Locus_16644_Transcript_1/1_Confidence_1.000_Length_426	REVERSE	55.25	47.62	AGCGTAGAACCCTACAGAATC						
Locus_16645_Transcript_1/1_Confidence_1.000_Length_424	FORWARD	55.54	47.62	AGAAGGAAGATCTGCGAGTAG	149	420	420	CT	CTCTCTCTCTCTCTCTCTCT	20

Locus_16645_Transcript_1/1_Confidence_1.000_Length_424	REVERSE	55.25	47.62	AGCGTAGAACCCTACAGAATC						
Locus_16903_Transcript_1/1_Confidence_1.000_Length_551	FORWARD	54.99	33.33	ACTCAAAATTGTCCTGCAATA	145	419	419	CT	CTCTCTCTCTCTCTCTCT	18
Locus_16903_Transcript_1/1_Confidence_1.000_Length_551	REVERSE	54.97	52.38	GAGAGAGAGAGAGTGGTGGAT						
Locus_16904_Transcript_1/1_Confidence_1.000_Length_284	FORWARD	54.99	33.33	ACTCAAAATTGTCCTGCAATA	145	284	284	CT	CTCTCTCTCTCTCTCTCT	18
Locus_16904_Transcript_1/1_Confidence_1.000_Length_284	REVERSE	54.97	52.38	GAGAGAGAGAGAGTGGTGGAT						
Locus_16905_Transcript_1/1_Confidence_1.000_Length_250	FORWARD	54.99	33.33	ACTCAAAATTGTCCTGCAATA	145	250	250	CT	CTCTCTCTCTCTCTCTCT	18
Locus_16905_Transcript_1/1_Confidence_1.000_Length_250	REVERSE	54.97	52.38	GAGAGAGAGAGAGTGGTGGAT						
Locus_17089_Transcript_1/1_Confidence_1.000_Length_298	FORWARD	55.08	38.1	TGACACAACGAAGAAGAAGTT	141	298	298	AG	AGAGAGAGAGAGAGAGAGAG	20
Locus_17089_Transcript_1/1_Confidence_1.000_Length_298	REVERSE	54.5	42.86	ATGTTCTCTTCTCCTCCATCT						
Locus_17313_Transcript_1/1_Confidence_1.000_Length_334	FORWARD	54.28	33.33	AAAACTCAACATTTGGTTCAC	150	334	334	AT	ATATATATATATATATATATAT	22
Locus_17313_Transcript_1/1_Confidence_1.000_Length_334	REVERSE	52.61	33.33	TAAGGTCAGTTTGAGATTGAA						
Locus_19085_Transcript_1/1_Confidence_1.000_Length_316	FORWARD	52.61	33.33	TAAGGTCAGTTTGAGATTGAA	151	316	316	AT	ATATATATATATATATATATAT	22
Locus_19085_Transcript_1/1_Confidence_1.000_Length_316	REVERSE	55.71	33.33	CAGGCAAAACAAGTTAAACAA						
Locus_19086_Transcript_1/1_Confidence_1.000_Length_336	FORWARD	52.61	33.33	TAAGGTCAGTTTGAGATTGAA	152	336	336	AT	ATATATATATATATATATATAT	22
Locus_19086_Transcript_1/1_Confidence_1.000_Length_336	REVERSE	54.28	33.33	AAAACTCAACATTTGGTTCAC						
Locus_19086_Transcript_1/1_Confidence_1.000_Length_336	FORWARD	55.11	33.33	GAGATTGAATTTCATGTTGGA	159	336	336	TA	TATATATATATATATATA	18
Locus_19086_Transcript_1/1_Confidence_1.000_Length_336	REVERSE	55.71	33.33	CAGGCAAAACAAGTTAAACAA						
Locus_19087_Transcript_1/1_Confidence_1.000_Length_314	FORWARD	52.61	33.33	TAAGGTCAGTTTGAGATTGAA	149	314	314	AT	ATATATATATATATATATAT	20
Locus_19087_Transcript_1/1_Confidence_1.000_Length_314	REVERSE	55.71	33.33	CAGGCAAAACAAGTTAAACAA						
Locus_19088_Transcript_1/1_Confidence_1.000_Length_318	FORWARD	55.11	33.33	GAGATTGAATTTCATGTTGGA	141	318	318	AT	ATATATATATATATATATATAT	22
Locus_19088_Transcript_1/1_Confidence_1.000_Length_318	REVERSE	55.71	33.33	CAGGCAAAACAAGTTAAACAA						
Locus_21552_Transcript_1/1_Confidence_1.000_Length_249	FORWARD	53.94	47.37	CTACGGTGGAAAAACACAC	176	249	249	CT	CTCTCTCTCTCTCTCTCTCT	20
Locus_21552_Transcript_1/1_Confidence_1.000_Length_249	REVERSE	53.91	38.1	GGATTATCCAAAGAGTTGTTG						
Locus_21553_Transcript_1/1_Confidence_1.000_Length_323	FORWARD	53.94	47.37	CTACGGTGGAAAAACACAC	124	323	323	CT	CTCTCTCTCTCTCTCTCTCT	20
Locus_21553_Transcript_1/1_Confidence_1.000_Length_323	REVERSE	55.52	38.1	TATAATGCGGGTATAATGCAG						
Locus_21555_Transcript_1/1_Confidence_1.000_Length_230	FORWARD	53.94	47.37	CTACGGTGGAAAAACACAC	169	230	230	CT	CTCTCTCTCTCTCTCTCTCT	20
Locus_21555_Transcript_1/1_Confidence_1.000_Length_230	REVERSE	55.39	38.1	TTATCCAAAGAGTTGTGATGC						

(B) Trinucleotide repeat more than 6 times.

Seq ID	Orientation	tm	GC%	Seq	Prod size	Motif	SSR	SSRLen
Locus_25_Transcript_59/302_Confidence_0.084_Length_2575	FORWARD	54.9	40.91	CTGGTAGATGGATCTATTTGGT	139	TTC	TTCTTCTTCTTCTTCTTCTTCTTC	24
Locus_25_Transcript_59/302_Confidence_0.084_Length_2575	REVERSE	54.98	36.36	GAAGAAGAAGAAGAAGAAGCAA				
Locus_27_Transcript_125/143_Confidence_0.269_Length_5400	FORWARD	54.81	36.36	CGTGTTGTGAAGAAAGATCTAA	153	TGA	TGATGATGATGATGATGATGATGA	24
Locus_27_Transcript_125/143_Confidence_0.269_Length_5400	REVERSE	54.95	38.1	TAACTTCCTCCAACTCATCAA				
Locus_29_Transcript_121/307_Confidence_0.050_Length_3068	FORWARD	56.4	52.63	GAAAGCTCGAGAGCAACAAC	264	ACA	ACAACAACAACAACAACAACAACAACAAC[illegible]	33
Locus_29_Transcript_121/307_Confidence_0.050_Length_3068	REVERSE	57.64	55.56	GCTGTGGGAGTATTTTGTCG				
Locus_36_Transcript_19/23_Confidence_0.589_Length_956	FORWARD	54.62	28.57	CAATAAGGTTTGGTTGAAAAA	152	GAT	GATGATGATGATGATGATGAT	21
Locus_36_Transcript_19/23_Confidence_0.589_Length_956	REVERSE	55.08	38.1	GGAGCTTTATTGATCTTCCAT				
Locus_163_Transcript_23/42_Confidence_0.552_Length_2405	FORWARD	54.92	42.86	GAGGAATCAAAGGAGATGACT	148	GAA	GAAGAAGAAGAAGAAGAAGAAGAA	24
Locus_163_Transcript_23/42_Confidence_0.552_Length_2405	REVERSE	54.63	42.86	CCTTCTTCCTTCTCCTTCTTA				
Locus_168_Transcript_15/35_Confidence_0.595_Length_2203	FORWARD	54.34	52.38	AGAGAGAGAGGGAGAGAGATG	114	GAG	GAGGAGGAGGAGGAGGAGGAG	21
Locus_168_Transcript_15/35_Confidence_0.595_Length_2203	REVERSE	54.77	38.1	CTTCTTTGATTCAATCCACAC				
Locus_174_Transcript_3/31_Confidence_0.373_Length_3861	FORWARD	54.04	50	GTACAAGCTGGGAGTTCTTC	167	GAG	GAGGAGGAGGAGGAGGAG	18
Locus_174_Transcript_3/31_Confidence_0.373_Length_3861	REVERSE	54.83	38.1	AATGAGAAGAAAAGGAGGAGA				
Locus_177_Transcript_49/62_Confidence_0.280_Length_2285	FORWARD	54.54	42.86	ACTCACTCTGAAAATGCTCAC	147	TCT	TCTTCTTCTTCTTCTTCTTCTTCTTCTTCT	30
Locus_177_Transcript_49/62_Confidence_0.280_Length_2285	REVERSE	55.28	38.1	TTAGAGGAGATGCAAACAAGA				
Locus_177_Transcript_49/62_Confidence_0.280_Length_2285	FORWARD	54.54	42.86	ACTCACTCTGAAAATGCTCAC	149	TTC	TTCTTCTTCTTCTTCTTCTTCTTC	24
Locus_177_Transcript_49/62_Confidence_0.280_Length_2285	REVERSE	54.62	38.1	GATTAGAGGAGATGCAAACAA				
Locus_178_Transcript_7/13_Confidence_0.627_Length_1991	FORWARD	55.62	40	TTTGTTACCCGTACGTGATT	142	GCC	GCCGCCGCCGCCGCCGCCGCC	21
Locus_178_Transcript_7/13_Confidence_0.627_Length_1991	REVERSE	54.97	42.86	AGCCTCTCACGACTTATTTCT				
Locus_206_Transcript_69/94_Confidence_0.185_Length_7868	FORWARD	55.39	47.62	CCCCCTATGTATATCAAGAGC	170	AGA	AGAAGAAGAAGAAGAAGA	18
Locus_206_Transcript_69/94_Confidence_0.185_Length_7868	REVERSE	54.88	38.1	CTCAAGCCTATTTGTTCTCAA				
Locus_252_Transcript_33/65_Confidence_0.587_Length_5610	FORWARD	55.26	42.86	GTGATACAGGCCAATCTTGTA	151	GCA	GCAGCAGCAGCAGCAGCA	18
Locus_252_Transcript_33/65_Confidence_0.587_Length_5610	REVERSE	55.03	40	CTGATTTTGGTGTTGGATCT				
Locus_262_Transcript_104/104_Confidence_0.386_Length_4827	FORWARD	54.94	33.33	GTATTTCAATTCCCGAAATCT	150	AAG	AAGAAGAAGAAGAAGAAG	18
Locus_262_Transcript_104/104_Confidence_0.386_Length_4827	REVERSE	54.87	42.86	TTCTTCTTCTGCTGTGACTTC				
Locus_293_Transcript_30/33_Confidence_0.617_Length_6683	FORWARD	55.04	38.1	AACAACAACAACATCAACCTC	142	AAC	AACAACAACAACAACAAC	18
Locus_293_Transcript_30/33_Confidence_0.617_Length_6683	REVERSE	54.53	33.33	AAAAAGATTCATGCAACTGAC				
Locus_293_Transcript_30/33_Confidence_0.617_Length_6683	FORWARD	55.3	42.86	GAGCAGCTGTATTTTGAGATG	154	TCA	TCATCATCATCATCATCATCATCA	24
Locus_293_Transcript_30/33_Confidence_0.617_Length_6683	REVERSE	56.03	33.33	ATCAATTGCATGAATCAGATG				
Locus_317_Transcript_60/81_Confidence_0.581_Length_4655	FORWARD	54.6	42.86	TCTTCATCGGAGTCTATCTTG	144	ATG	ATGATGATGATGATGATGATG	21
Locus_317_Transcript_60/81_Confidence_0.581_Length_4655	REVERSE	55.31	38.1	ACCCTAGCCAAAATACATCAT				
Locus_372_Transcript_29/37_Confidence_0.298_Length_1455	FORWARD	57.14	45	ACCCAAAAGCTCTCCTCTTT	151	CTG	CTGCTGCTGCTGCTGCTG	18
Locus_372_Transcript_29/37_Confidence_0.298_Length_1455	REVERSE	54.85	42.86	TGCACATAAGTCAGACAAGTG				
Locus_418_Transcript_33/61_Confidence_0.206_Length_1216	FORWARD	55.16	42.86	ACTATTAACAAGGGCGTAACC	128	ATA	ATAATAATAATAATAATAATA	21
Locus_418_Transcript_33/61_Confidence_0.206_Length_1216	REVERSE	54.57	33.33	TAAGTAATTTTGTCCCAATCG				
Locus_432_Transcript_70/100_Confidence_0.356_Length_2066	FORWARD	56.19	34.78	TGTTCTGAGAGGTTTGTATTTTG	162	AAG	AAGAAGAAGAAGAAGAAG	18
Locus_432_Transcript_70/100_Confidence_0.356_Length_2066	REVERSE	59.06	55	GGATCTCACCGGATCTCACT				

Locus_448_Transcript_388/463_Confidence_0.042_Length_4620	FORWARD	55.09	38.1	CGTATCTTAAGTCGCAGAAAA	155	TCC	TCCTCCTCCTCCTCCTCC	18
Locus_448_Transcript_388/463_Confidence_0.042_Length_4620	REVERSE	55.12	47.62	CTTTAGACGAGGAAGAGGAAG				
Locus_463_Transcript_26/45_Confidence_0.435_Length_1525	FORWARD	54.92	42.86	TCAGGACCTTGAAGATGTAGA	150	CTT	CTTCTTCTTCTTCTTCTT	18
Locus_463_Transcript_26/45_Confidence_0.435_Length_1525	REVERSE	55.17	35	AAAAGAAACCAGTTGCAGAA				
Locus_470_Transcript_37/42_Confidence_0.474_Length_1552	FORWARD	53	28.57	CATCCAAACATTAACTTTTCA	145	AAT	AATAATAATAATAATAATAAT	21
Locus_470_Transcript_37/42_Confidence_0.474_Length_1552	REVERSE	54.99	40.91	GTATTGACTGTTTTGAGTGTCG				
Locus_504_Transcript_8/30_Confidence_0.623_Length_1266	FORWARD	54.71	40	GAATTAATGAGATCGCTGCT	150	GAA	GAAGAAGAAGAAGAAGAA	18
Locus_504_Transcript_8/30_Confidence_0.623_Length_1266	REVERSE	54.47	33.33	TCGGTAAATCGACATTTAATC				
Locus_516_Transcript_115/164_Confidence_0.244_Length_9547	FORWARD	54.39	31.82	AACATCAATAGGGAAAATAAGC	152	TGC	TGCTGCTGCTGCTGCTGCTGCTGC	24
Locus_516_Transcript_115/164_Confidence_0.244_Length_9547	REVERSE	55.02	42.86	GACATAGCCTACATGAAAACG				
Locus_559_Transcript_12/14_Confidence_0.669_Length_1496	FORWARD	54.51	38.1	TGAGAGCGAGAAATATAGCAT	155	TTG	TTGTTGTTGTTGTTGTTGTTGTTGTTG	27
Locus_559_Transcript_12/14_Confidence_0.669_Length_1496	REVERSE	54.94	33.33	ACAACAACAACAACAACAACA				
Locus_559_Transcript_12/14_Confidence_0.669_Length_1496	FORWARD	54.94	33.33	TGTTGTTGTTGTTGTTGTTGT	150	TTG	TTGTTGTTGTTGTTGTTGTTGTTGTTG	27
Locus_559_Transcript_12/14_Confidence_0.669_Length_1496	REVERSE	54.57	33.33	CATAAATACGCCGAACATAAT				
Locus_593_Transcript_18/25_Confidence_0.574_Length_2205	FORWARD	54.98	33.33	TGATATGGATGGTAAAAATGG	147	GGC	GGCGGCGGCGGCGGCGGCGGC	21
Locus_593_Transcript_18/25_Confidence_0.574_Length_2205	REVERSE	55.3	42.86	TCTCCACACTCGTAACATTTC				
Locus_609_Transcript_20/23_Confidence_0.590_Length_1511	FORWARD	54.93	42.86	GTCATCATCAACTTCACCACT	162	GCC	GCCGCCGCCGCCGCCGCC	18
Locus_609_Transcript_20/23_Confidence_0.590_Length_1511	REVERSE	54.91	28.57	TGAAACCAATTGGAATCTAAA				
Locus_615_Transcript_28/58_Confidence_0.419_Length_1952	FORWARD	54.9	45	CCGAACAACTCATCTTCTTC	162	CCT	CCTCCTCCTCCTCCTCCT	18
Locus_615_Transcript_28/58_Confidence_0.419_Length_1952	REVERSE	55.93	42.86	TGCCCAAGTCTGATGTTACTA				
Locus_632_Transcript_4/8_Confidence_0.679_Length_1474	FORWARD	54.1	42.86	GTTCTAAGCAAACTCCACATC	167	CCT	CCTCCTCCTCCTCCTCCTCCT	21
Locus_632_Transcript_4/8_Confidence_0.679_Length_1474	REVERSE	54.77	47.62	GAATAGGAGGAAGATGAGAGC				
Locus_699_Transcript_37/53_Confidence_0.525_Length_2441	FORWARD	55.27	38.1	GAGGATTCTTTCCATGAAATC	171	TTC	TTCTTCTTCTTCTTCTTC	18
Locus_699_Transcript_37/53_Confidence_0.525_Length_2441	REVERSE	55.36	33.33	GTGTTCACCAAAGAAACAAAA				
Locus_774_Transcript_17/24_Confidence_0.648_Length_4742	FORWARD	54.84	38.1	AAGCTGTATGGTTTCCTTCTT	143	AGA	AGAAGAAGAAGAAGAAGAAGAAGAAGA	27
Locus_774_Transcript_17/24_Confidence_0.648_Length_4742	REVERSE	55.01	42.86	TCAGCACAGATTTAAGCTCTC				
Locus_793_Transcript_11/15_Confidence_0.589_Length_956	FORWARD	54.93	42.86	AATACACAGGTCTGGTTAGCA	169	CAG	CAGCAGCAGCAGCAGCAG	18
Locus_793_Transcript_11/15_Confidence_0.589_Length_956	REVERSE	54.9	42.86	TTGGCTCTCTAACAACATAGC				
Locus_861_Transcript_27/41_Confidence_0.301_Length_1385	FORWARD	54.86	38.1	TTAATCCAATGTCTGAGGAGA	150	AGG	AGGAGGAGGAGGAGGAGG	18
Locus_861_Transcript_27/41_Confidence_0.301_Length_1385	REVERSE	55.95	38.1	CGGCGGATTTATAATATGTCT				
Locus_925_Transcript_4/14_Confidence_0.620_Length_3080	FORWARD	54.91	38.1	AATCGACTGACTTCATCTTCA	146	TCA	TCATCATCATCATCATCATCA	21
Locus_925_Transcript_4/14_Confidence_0.620_Length_3080	REVERSE	54.19	42.86	CTACCGAGTAATGACATGGAT				
Locus_925_Transcript_4/14_Confidence_0.620_Length_3080	FORWARD	54.7	33.33	CATCTCTTTTGTTTTGCAGAT	157	TCA	TCATCATCATCATCATCATCA	21
Locus_925_Transcript_4/14_Confidence_0.620_Length_3080	REVERSE	55.33	52.38	GAGTCGGGGACACTTACTATC				
Locus_959_Transcript_15/21_Confidence_0.597_Length_1216	FORWARD	55.49	50	GGGGATACAGAAGATGGAGT	142	AGG	AGGAGGAGGAGGAGGAGG	18
Locus_959_Transcript_15/21_Confidence_0.597_Length_1216	REVERSE	55.3	42.86	AGGATCGGTCGAATACACTAT				
Locus_1059_Transcript_25/31_Confidence_0.640_Length_1966	FORWARD	54.36	38.1	AGAAGTTCGATTCTGCTATGA	153	CGC	CGCCGCCGCCGCCGCCGCCGCCGC	24
Locus_1059_Transcript_25/31_Confidence_0.640_Length_1966	REVERSE	55.12	42.86	GGGAAGGAAAGAGAGATGTAA				
Locus_1069_Transcript_41/64_Confidence_0.406_Length_2993	FORWARD	54.5	47.62	GAGGTGTTGAGTGTAGCTGTT	156	TCT	TCTTCTTCTTCTTCTTCT	18
Locus_1069_Transcript_41/64_Confidence_0.406_Length_2993	REVERSE	55.33	38.1	ACCGGAATATAACAAACCCTA				
Locus_1090_Transcript_13/25_Confidence_0.630_Length_2059	FORWARD	55.34	42.86	ATCATACAAGGCAGACAACAG	176	AGC	AGCAGCAGCAGCAGCAGC	18

Locus_1090_Transcript_13/25_Confidence_0.630_Length_2059	REVERSE	55.62	47.62	GAGTTGGCTCTTAGACATTCC				
Locus_1112_Transcript_10/44_Confidence_0.590_Length_3701	FORWARD	55.18	42.86	GGGATTTAGGTATATGGATCG	172	GGT	GGTGGTGGTGGTGGTGGT	18
Locus_1112_Transcript_10/44_Confidence_0.590_Length_3701	REVERSE	54.94	50	CATGCTCAACCTTCTCCTAC				
Locus_1114_Transcript_6/30_Confidence_0.590_Length_2022	FORWARD	53.11	34.78	ACATATCATAACTTACCCACTCA	146	AAT	AATAATAATAATAATAAT	18
Locus_1114_Transcript_6/30_Confidence_0.590_Length_2022	REVERSE	54.04	30.43	AGTTGGTGTTATAATTGTTGTCA				
Locus_1117_Transcript_10/11_Confidence_0.647_Length_907	FORWARD	55.22	38.1	ATCATAGAAAGATGAGCAGCA	256	GCC	GCCGCCGCCGCCGCCGCCGCC	21
Locus_1117_Transcript_10/11_Confidence_0.647_Length_907	REVERSE	54.9	42.86	AGAGGCTGTTAAGTTTCCACT				
Locus_1157_Transcript_7/12_Confidence_0.685_Length_1449	FORWARD	54.04	27.27	AAATTGGACCCTATAAGAAAAA	147	TTG	TTGTTGTTGTTGTTGTTGTTGTTGTTGTTGT	33
Locus_1157_Transcript_7/12_Confidence_0.685_Length_1449	REVERSE	54.57	33.33	CATAAATACGCCGAACATAAT				
Locus_1158_Transcript_2/11_Confidence_0.688_Length_3223	FORWARD	54.22	38.1	GAAAGAAGAAAAGGAAGAAGG	154	AGG	AGGAGGAGGAGGAGGAGGAGGAGGAGG	27
Locus_1158_Transcript_2/11_Confidence_0.688_Length_3223	REVERSE	54.79	47.62	TACTACCCTCGTCAGCTATTG				
Locus_1158_Transcript_2/11_Confidence_0.688_Length_3223	FORWARD	54.79	42.86	CAGCAGCAGAAGAAGAATAAG	151	AGG	AGGAGGAGGAGGAGGAGGAGGAGGAGG	27
Locus_1158_Transcript_2/11_Confidence_0.688_Length_3223	REVERSE	55.05	33.33	TTAGCATCATCATCATTCACA				
Locus_1161_Transcript_55/102_Confidence_0.536_Length_2614	FORWARD	55.63	42.86	ACCGAGGATCTGAGAGTTTTA	145	CTT	CTTCTTCTTCTTCTTCTT	18
Locus_1161_Transcript_55/102_Confidence_0.536_Length_2614	REVERSE	55.07	33.33	AAACAGTCCAATTAAACAGCA				
Locus_1195_Transcript_51/74_Confidence_0.470_Length_2688	FORWARD	54.74	33.33	GATTGATTCTTTGCTTGAGAA	151	CTT	CTTCTTCTTCTTCTTCTT	18
Locus_1195_Transcript_51/74_Confidence_0.470_Length_2688	REVERSE	55	38.1	AGATGCTGATGAAGATGAAGA				
Locus_1195_Transcript_51/74_Confidence_0.470_Length_2688	FORWARD	54.97	47.62	CCTCATCTCCCTCTAACTCAT	144	CTT	CTTCTTCTTCTTCTTCTT	18
Locus_1195_Transcript_51/74_Confidence_0.470_Length_2688	REVERSE	55.06	47.62	GAGGAAGAGAAGAAGAGAACG				
Locus_1220_Transcript_16/18_Confidence_0.543_Length_2694	FORWARD	57.51	50	CTATGAGCCCGGTGATGTAT	156	AAG	AAGAAGAAGAAGAAGAAG	18
Locus_1220_Transcript_16/18_Confidence_0.543_Length_2694	REVERSE	54.76	42.86	TTTGCTCTCTGACCTTAACAC				
Locus_1276_Transcript_3/10_Confidence_0.638_Length_1556	FORWARD	54.71	42.86	CTTTTCACTTCTCTCCACTCA	153	ATC	ATCATCATCATCATCATCATC	21
Locus_1276_Transcript_3/10_Confidence_0.638_Length_1556	REVERSE	55.34	42.86	GAGGACTTGTTGTTTCTGACA				
Locus_1338_Transcript_4/11_Confidence_0.671_Length_3687	FORWARD	55.12	33.33	AAATTATGAAGGGTGGAACAT	141	TTA	TTATTATTATTATTATTATTA	21
Locus_1338_Transcript_4/11_Confidence_0.671_Length_3687	REVERSE	56.08	35	TTTTTAAAGCAGCAAACAGC				
Locus_1358_Transcript_12/26_Confidence_0.596_Length_1705	FORWARD	54.66	33.33	CTGACGGAAGATTGAAAATTA	165	TCA	TCATCATCATCATCATCATCATCA	24
Locus_1358_Transcript_12/26_Confidence_0.596_Length_1705	REVERSE	54.59	42.86	TGGTCTTCGGTAAGAAGTATG				
Locus_1382_Transcript_31/44_Confidence_0.498_Length_2303	FORWARD	54.88	42.86	CAGATGCTGATAAAGATGGAC	151	TGA	TGATGATGATGATGATGA	18
Locus_1382_Transcript_31/44_Confidence_0.498_Length_2303	REVERSE	54.82	38.1	AAACTTCTAACCTGGAAAGGA				
Locus_1385_Transcript_32/36_Confidence_0.455_Length_1324	FORWARD	54.77	33.33	TGAATCAGTGTTTTCTGGAAT	143	GAA	GAAGAAGAAGAAGAAGAA	18
Locus_1385_Transcript_32/36_Confidence_0.455_Length_1324	REVERSE	55.01	38.1	CAAATACAATTCCCTCTCCTT				
Locus_1390_Transcript_27/33_Confidence_0.586_Length_1949	FORWARD	54.98	38.1	TATCCAAGAATCATCACAACC	169	TTA	TTATTATTATTATTATTATTA	21
Locus_1390_Transcript_27/33_Confidence_0.586_Length_1949	REVERSE	55.14	42.86	GAGGAGAGAATGCCTGTTTAT				
Locus_1433_Transcript_149/150_Confidence_0.287_Length_3713	FORWARD	54.82	38.1	GCGACTATAATCTGCCAAATA	156	GAA	GAAGAAGAAGAAGAAGAA	18
Locus_1433_Transcript_149/150_Confidence_0.287_Length_3713	REVERSE	55.24	52.38	CTCTCTCTGTCTCCCTCTGTT				
Locus_1435_Transcript_6/11_Confidence_0.708_Length_2457	FORWARD	55.38	42.86	AGCTGGAGAAGGTTTAATCAG	151	CAT	CATCATCATCATCATCAT	18
Locus_1435_Transcript_6/11_Confidence_0.708_Length_2457	REVERSE	54.75	38.1	TATTGTACAACCACCACATGA				
Locus_1437_Transcript_8/18_Confidence_0.614_Length_2264	FORWARD	54.65	38.1	TCAGTTTTCCTTCATTCTCTG	148	AGC	AGCAGCAGCAGCAGCAGC	18
Locus_1437_Transcript_8/18_Confidence_0.614_Length_2264	REVERSE	54.4	47.62	GAAGTAGTGCAGATCTCAAGC				
Locus_1461_Transcript_31/88_Confidence_0.333_Length_3170	FORWARD	54.44	38.1	CGTAATTTAGGGTTTCCTTCT	149	TCT	TCTTCTTCTTCTTCTTCT	18
Locus_1461_Transcript_31/88_Confidence_0.333_Length_3170	REVERSE	55.16	28.57	ATCAAACGCATCAAAACTAAA				

Locus_1461_Transcript_31/88_Confidence_0.333_Length_3170	FORWARD	55.05	38.1	TGGGTAATTATGGTTCTGATG	153	GAT	GATGATGATGATGATGATGAT	21
Locus_1461_Transcript_31/88_Confidence_0.333_Length_3170	REVERSE	54.34	42.86	TCCTTTACTGTCTCAACATCC				
Locus_1514_Transcript_7/39_Confidence_0.529_Length_2012	FORWARD	54.61	33.33	AGGCTTTCATATACCCAAAAT	150	TAT	TATTATTATTATTATTATTATTAT	24
Locus_1514_Transcript_7/39_Confidence_0.529_Length_2012	REVERSE	54.95	31.82	TGTGACTTGAAATGTTGTTTCT				
Locus_1520_Transcript_36/42_Confidence_0.490_Length_1086	FORWARD	55.54	38.1	ATCAGCCTTCATGATCTTCTT	152	GCT	GCTGCTGCTGCTGCTGCTGCTGCT	24
Locus_1520_Transcript_36/42_Confidence_0.490_Length_1086	REVERSE	55.31	50	AACTCTACCACCATCAGCAG				
Locus_1569_Transcript_22/26_Confidence_0.686_Length_3296	FORWARD	54.77	45.45	GACGTTATAAAGGGTACTGAGG	179	TTC	TTCTTCTTCTTCTTCTTCTTCTTC	24
Locus_1569_Transcript_22/26_Confidence_0.686_Length_3296	REVERSE	54.71	47.62	CCTTCTTCTTCCTCTATGTCC				
Locus_1636_Transcript_37/46_Confidence_0.591_Length_2024	FORWARD	57.57	42.86	ATGTCAAGGTGAAGGAGGAAT	209	GAG	GAGGAGGAGGAGGAGGAG	18
Locus_1636_Transcript_37/46_Confidence_0.591_Length_2024	REVERSE	55.21	40.91	GTATCCAATCATCCGAACTATC				
Locus_1705_Transcript_37/42_Confidence_0.546_Length_1219	FORWARD	54.62	45	CGATTCCATAAGATCTCCAC	162	CCT	CCTCCTCCTCCTCCTCCTCCT	21
Locus_1705_Transcript_37/42_Confidence_0.546_Length_1219	REVERSE	55.03	47.62	CCACCACCTCCTAGTTTTACT				
Locus_1733_Transcript_6/15_Confidence_0.594_Length_1614	FORWARD	54.88	38.1	TGTCATCTTCATTAGCTCCAT	145	CCT	CCTCCTCCTCCTCCTCCT	18
Locus_1733_Transcript_6/15_Confidence_0.594_Length_1614	REVERSE	55.2	42.86	GAGAGGAAGAATCGGATAAGA				
Locus_1737_Transcript_8/27_Confidence_0.494_Length_2012	FORWARD	55.39	42.86	GGATTGGTCATGGGTATAGTT	130	GAT	GATGATGATGATGATGAT	18
Locus_1737_Transcript_8/27_Confidence_0.494_Length_2012	REVERSE	54.91	33.33	TCATTCAGAGCATACCAAAAT				
Locus_1756_Transcript_103/107_Confidence_0.282_Length_2877	FORWARD	54.58	38.1	CCAATAAAGCTTCTGTGTCAT	139	TCA	TCATCATCATCATCATCATCA	21
Locus_1756_Transcript_103/107_Confidence_0.282_Length_2877	REVERSE	54.46	38.1	CTTGAAGGAGGAAAAAGAGAT				
Locus_1756_Transcript_103/107_Confidence_0.282_Length_2877	FORWARD	55.14	40	GCGTTTCCTTAGAATTTGTG	160	TCA	TCATCATCATCATCATCATCATCA	24
Locus_1756_Transcript_103/107_Confidence_0.282_Length_2877	REVERSE	54.97	33.33	TTGGACCTGAAATCTTAAACA				
Locus_1792_Transcript_40/42_Confidence_0.485_Length_1340	FORWARD	54.37	42.86	CGTCATCTTTGGTCTGTCTAT	140	TCT	TCTTCTTCTTCTTCTTCTTCTTCT	24
Locus_1792_Transcript_40/42_Confidence_0.485_Length_1340	REVERSE	54.94	36.36	AGCAAACAAGAAGAAGAAGAAG				
Locus_1792_Transcript_40/42_Confidence_0.485_Length_1340	FORWARD	54.98	42.86	CTCGCTCTTCTTCTTCTTCTT	149	TTC	TTCTTCTTCTTCTTCTTCTTCTTC	24
Locus_1792_Transcript_40/42_Confidence_0.485_Length_1340	REVERSE	55.3	42.86	ATCTGTACGGTAACACAAACG				
Locus_1831_Transcript_13/38_Confidence_0.546_Length_2408	FORWARD	55.3	47.62	GTCTCCATTTAAGTGGTCTCC	135	TCT	TCTTCTTCTTCTTCTTCTTCTTCT	24
Locus_1831_Transcript_13/38_Confidence_0.546_Length_2408	REVERSE	55.27	33.33	CGAACAACGTAATTGATTGAT				
Locus_1904_Transcript_18/32_Confidence_0.594_Length_9055	FORWARD	55.4	33.33	GGTTGCATTTGAACTTGAATA	180	GCG	GCGGCGGCGGCGGCGGCGGCGGCG	24
Locus_1904_Transcript_18/32_Confidence_0.594_Length_9055	REVERSE	54.95	33.33	CCCCAATTCTCAAATTTCTAT				
Locus_1914_Transcript_14/23_Confidence_0.495_Length_3518	FORWARD	55.14	50	GCAGAAACAGAGGAAGACAG	147	GAT	GATGATGATGATGATGATGATGATGATGA	33
Locus_1914_Transcript_14/23_Confidence_0.495_Length_3518	REVERSE	54.19	38.1	AGCCAACAGAATCTTCTATCA				
Locus_1915_Transcript_12/38_Confidence_0.608_Length_1915	FORWARD	54.75	33.33	ATGAGTTTGTTTTGGTTGGTA	153	ATG	ATGATGATGATGATGATG	18
Locus_1915_Transcript_12/38_Confidence_0.608_Length_1915	REVERSE	55.09	38.1	GTATGTAAATGCCCCATAACA				
Locus_1926_Transcript_14/28_Confidence_0.611_Length_1072	FORWARD	55.1	33.33	TCTGAAGCATTCTCAATTGTT	150	TCT	TCTTCTTCTTCTTCTTCT	18
Locus_1926_Transcript_14/28_Confidence_0.611_Length_1072	REVERSE	55.15	42.86	AGAGAGAGCAACATGAACAGA				
Locus_2029_Transcript_32/44_Confidence_0.599_Length_2413	FORWARD	54.49	38.1	GCTAACCAGTTCACAAACAAT	155	CGG	CGGCGGCGGCGGCGGCGG	18
Locus_2029_Transcript_32/44_Confidence_0.599_Length_2413	REVERSE	55.2	38.1	AATTTTCTTCCAGTTCTGAGG				
Locus_2075_Transcript_7/14_Confidence_0.652_Length_1348	FORWARD	55.75	38.1	GGATGAATCAGCTATGGATTT	151	TTC	TTCTTCTTCTTCTTCTTCTTC	21
Locus_2075_Transcript_7/14_Confidence_0.652_Length_1348	REVERSE	54.94	36.36	AGCAAACAAGAAGAAGAAGAAG				
Locus_2075_Transcript_7/14_Confidence_0.652_Length_1348	FORWARD	54.56	42.86	TGTGCTTCTAGAGAGGAAGAA	167	TTC	TTCTTCTTCTTCTTCTTCTTCTTC	24
Locus_2075_Transcript_7/14_Confidence_0.652_Length_1348	REVERSE	55.67	40.91	GGAGCAACTATACATTTCCTTG				
Locus_2078_Transcript_22/29_Confidence_0.598_Length_3107	FORWARD	55.72	52.38	CACCTATCACACGACCAGTAG	150	GGT	GGTGGTGGTGGTGGTGGT	18

Locus_2078_Transcript_22/29_Confidence_0.598_Length_3107	REVERSE	55.43	42.86	CAAGTCTTCAAGCTCAAACAG				
Locus_2093_Transcript_28/37_Confidence_0.576_Length_994	FORWARD	55.58	42.86	CTCTTCTTCTGTTTGGCTTCT	149	CTG	CTGCTGCTGCTGCTGCTGCTGCTGCTG	27
Locus_2093_Transcript_28/37_Confidence_0.576_Length_994	REVERSE	55.47	47.62	TCAGCATAGTATCCACTACGG				
Locus_2319_Transcript_15/30_Confidence_0.625_Length_3196	FORWARD	55.04	38.1	AAAACACAAACATGGTCTCAC	160	ATT	ATTATTATTATTATTATT	18
Locus_2319_Transcript_15/30_Confidence_0.625_Length_3196	REVERSE	55.63	45	CCTTGTTGCCTGAACTTCTA				
Locus_2350_Transcript_8/10_Confidence_0.683_Length_2253	FORWARD	54.2	42.86	CCATTAGTTGTACTCCAAAGC	151	GAT	GATGATGATGATGATGAT	18
Locus_2350_Transcript_8/10_Confidence_0.683_Length_2253	REVERSE	55.57	33.33	TCATCATCATCATCTTCGTTT				
Locus_2350_Transcript_8/10_Confidence_0.683_Length_2253	FORWARD	54.92	38.1	CTCCATTAAACGAAGATGATG	149	GAT	GATGATGATGATGATGATGAT	21
Locus_2350_Transcript_8/10_Confidence_0.683_Length_2253	REVERSE	55.04	38.1	ATGTTGTGTGTTTGGTTCTTC				
Locus_2400_Transcript_10/26_Confidence_0.607_Length_2492	FORWARD	54.89	42.86	TCCTCCTCACTTCTTCTTCTT	117	TTC	TTCTTCTTCTTCTTCTTCTTC	21
Locus_2400_Transcript_10/26_Confidence_0.607_Length_2492	REVERSE	54.77	38.1	TCGCAGTTGTATTTTAGTTCC				
Locus_2434_Transcript_15/28_Confidence_0.545_Length_2352	FORWARD	54.99	42.86	AAGTCCTATCATCTTCCCAAG	143	TTG	TTGTTGTTGTTGTTGTTGTTG	21
Locus_2434_Transcript_15/28_Confidence_0.545_Length_2352	REVERSE	55.25	42.86	TCACAGACTCACGCATACATA				
Locus_2444_Transcript_13/49_Confidence_0.611_Length_2252	FORWARD	56.33	50	CTTCCCTTCACCTCCATAAG	148	TCC	TCCTCCTCCTCCTCCTCC	18
Locus_2444_Transcript_13/49_Confidence_0.611_Length_2252	REVERSE	54.24	42.86	TGTGAGCTTGGTAGAGAGATT				
Locus_2496_Transcript_18/28_Confidence_0.538_Length_1997	FORWARD	54.82	42.86	CATATGGTTGACAAGGAAGAG	209	GAA	GAAGAAGAAGAAGAAGAA	18
Locus_2496_Transcript_18/28_Confidence_0.538_Length_1997	REVERSE	54.23	42.86	GATCATTACCTCATCGGAGTA				
Locus_2592_Transcript_68/83_Confidence_0.594_Length_4085	FORWARD	54.73	30	TTTTTGGGTTAAAAATACGC	149	TAA	TAATAATAATAATAATAATAA	21
Locus_2592_Transcript_68/83_Confidence_0.594_Length_4085	REVERSE	54.73	38.1	TCTCTGAATTCTTCTGATTCG				
Locus_2610_Transcript_27/50_Confidence_0.610_Length_15561	FORWARD	55.36	42.86	TGGTACTTGTACGAAATCCAG	158	TGA	TGATGATGATGATGATGA	18
Locus_2610_Transcript_27/50_Confidence_0.610_Length_15561	REVERSE	54.11	47.62	AGAGGCTAGTTCCTTCTCACT				
Locus_2726_Transcript_5/13_Confidence_0.618_Length_2238	FORWARD	55.22	38.1	CTCGATCATCATGAAAACTGT	144	CAT	CATCATCATCATCATCAT	18
Locus_2726_Transcript_5/13_Confidence_0.618_Length_2238	REVERSE	55.22	42.86	GGCTTTACCTCAGAAACTGAT				
Locus_2726_Transcript_5/13_Confidence_0.618_Length_2238	FORWARD	55.22	38.1	CTCGATCATCATGAAAACTGT	144	TCT	TCTTCTTCTTCTTCTTCTTCT	21
Locus_2726_Transcript_5/13_Confidence_0.618_Length_2238	REVERSE	55.22	42.86	GGCTTTACCTCAGAAACTGAT				
Locus_2774_Transcript_13/23_Confidence_0.437_Length_2369	FORWARD	55.31	42.86	ATGGAAGAGGATAAGCTTGAG	157	AGA	AGAAGAAGAAGAAGAAGA	18
Locus_2774_Transcript_13/23_Confidence_0.437_Length_2369	REVERSE	54.96	33.33	GGTTTTTCTTTTTCTTCTTGG				
Locus_2776_Transcript_4/11_Confidence_0.677_Length_1835	FORWARD	54.71	47.62	GAAGAGTATTTGGAGGAGGAG	147	GGA	GGAGGAGGAGGAGGAGGAGGA	21
Locus_2776_Transcript_4/11_Confidence_0.677_Length_1835	REVERSE	55.76	42.86	CCTTTGGAGAAGAGATTGAAG				
Locus_2776_Transcript_4/11_Confidence_0.677_Length_1835	FORWARD	54.76	33.33	ATTAATGGCGGAAGAGTATTT	157	GGA	GGAGGAGGAGGAGGAGGAGGA	21
Locus_2776_Transcript_4/11_Confidence_0.677_Length_1835	REVERSE	55.76	42.86	CCTTTGGAGAAGAGATTGAAG				
Locus_2818_Transcript_29/73_Confidence_0.469_Length_3397	FORWARD	55.14	33.33	CCACATAACCCAAACAAATTA	150	AAT	AATAATAATAATAATAAT	18
Locus_2818_Transcript_29/73_Confidence_0.469_Length_3397	REVERSE	55.09	42.86	GTTTGAGGTTTCTGAAGGACT				
Locus_2857_Transcript_15/16_Confidence_0.649_Length_6622	FORWARD	55.09	38.1	GACATGGATAGTGTTGCAAAT	170	ATG	ATGATGATGATGATGATGATG	21
Locus_2857_Transcript_15/16_Confidence_0.649_Length_6622	REVERSE	55.36	28.57	ATCTTAAATTGCCCATTCATT				
Locus_2865_Transcript_1/6_Confidence_0.636_Length_833	FORWARD	54.39	42.86	AAGGGTTACTGTTTCTGTGTG	103	GGT	GGTGGTGGTGGTGGTGGT	18
Locus_2865_Transcript_1/6_Confidence_0.636_Length_833	REVERSE	55.19	38.1	TGCAAATATCTACACCAATCC				
Locus_2923_Transcript_33/44_Confidence_0.436_Length_1186	FORWARD	54.81	47.62	GATGAAGAGGAAGAAGAGGAG	152	GAT	GATGATGATGATGATGATGAT	21
Locus_2923_Transcript_33/44_Confidence_0.436_Length_1186	REVERSE	55.57	50	AGGTACTCTGTCCCCATTTC				
Locus_2960_Transcript_12/17_Confidence_0.578_Length_6370	FORWARD	54.58	42.86	CTATGCCTTCTCATCCTGATA	151	CAG	CAGCAGCAGCAGCAGCAG	18
Locus_2960_Transcript_12/17_Confidence_0.578_Length_6370	REVERSE	54.49	28.57	ATGTTTGTTGGTGATTGATTT				

Locus_2971_Transcript_15/22_Confidence_0.580_Length_1720	FORWARD	54.83	38.1	TTCTGAGGAAAGAGGAGAAAT	150	GCG	GCGGCGGCGGCGGCGGCGGCG	21
Locus_2971_Transcript_15/22_Confidence_0.580_Length_1720	REVERSE	54.4	33.33	TTGTTCTCTATTTCGAAGCAT				
Locus_3006_Transcript_114/121_Confidence_0.164_Length_3674	FORWARD	54.89	33.33	CAATTCTGGATTTTGATTCTG	154	TTA	TTATTATTATTATTATTATTA	21
Locus_3006_Transcript_114/121_Confidence_0.164_Length_3674	REVERSE	54.93	28.57	TAATTTGAATCACCAATGCTT				
Locus_3024_Transcript_6/10_Confidence_0.664_Length_3980	FORWARD	55.09	40	TAACGAACGATTGGCTCTAT	133	GCC	GCCGCCGCCGCCGCCGCCGCC	21
Locus_3024_Transcript_6/10_Confidence_0.664_Length_3980	REVERSE	54.04	50	GTTCCTCGACGAGAGTTTAC				
Locus_3116_Transcript_11/19_Confidence_0.570_Length_2489	FORWARD	54.92	42.86	TTCCTCATCTCTACAACCTCA	151	CCG	CCGCCGCCGCCGCCGCCGCCG	21
Locus_3116_Transcript_11/19_Confidence_0.570_Length_2489	REVERSE	55.49	33.33	TAGATCGTGTTTTTGTTTTGG				
Locus_3222_Transcript_15/39_Confidence_0.689_Length_2347	FORWARD	54.84	38.1	GAGTATTGAATCCCCAAGTTT	150	GAG	GAGGAGGAGGAGGAGGAGGAGGAG	24
Locus_3222_Transcript_15/39_Confidence_0.689_Length_2347	REVERSE	54.56	38.1	TTCAGAATCATCTTTCTCCTG				
Locus_3243_Transcript_22/41_Confidence_0.493_Length_3919	FORWARD	55	38.1	TCTTACGGGATTTCACAGTTA	135	AAG	AAGAAGAAGAAGAAGAAG	18
Locus_3243_Transcript_22/41_Confidence_0.493_Length_3919	REVERSE	55.24	38.1	TCTCATGGAATTCGCTACTTA				
Locus_3268_Transcript_16/27_Confidence_0.381_Length_1825	FORWARD	55.46	45	TAACTCGCCTTCTCGTCTTA	166	AAC	AACAACAACAACAACAACAACAACAAC	27
Locus_3268_Transcript_16/27_Confidence_0.381_Length_1825	REVERSE	55	27.27	TTTTTCCATTACTGTTTGATGA				
Locus_3298_Transcript_21/30_Confidence_0.628_Length_1363	FORWARD	55.22	47.62	CAGAGAAGGAGAAGAAGAAGC	169	AGA	AGAAGAAGAAGAAGAAGAAGAAGA	24
Locus_3298_Transcript_21/30_Confidence_0.628_Length_1363	REVERSE	55.17	42.86	TAACAACTTACACCTGCTCGT				
Locus_3327_Transcript_38/48_Confidence_0.421_Length_1690	FORWARD	54.48	42.86	AATCTAACTCAAAGCCCTCTC	160	GAA	GAAGAAGAAGAAGAAGAA	18
Locus_3327_Transcript_38/48_Confidence_0.421_Length_1690	REVERSE	54.72	42.86	CATATCCAGAACTCGTCATTC				
Locus_3355_Transcript_3/18_Confidence_0.579_Length_2015	FORWARD	55.18	47.62	CCTAATCCAGTGGTGTCTGTA	133	TGC	TGCTGCTGCTGCTGCTGC	18
Locus_3355_Transcript_3/18_Confidence_0.579_Length_2015	REVERSE	55.08	45	AGCAGCAGTCTCTGATGATT				
Locus_3376_Transcript_101/115_Confidence_0.336_Length_5494	FORWARD	54.98	52.38	GGCTAGGTAGAGAGAAACCTG	180	CTC	CTCCTCCTCCTCCTCCTC	18
Locus_3376_Transcript_101/115_Confidence_0.336_Length_5494	REVERSE	55.32	38.1	TGGCGAGACAATAAGTAAGAA				
Locus_3421_Transcript_8/29_Confidence_0.429_Length_1397	FORWARD	54.35	33.33	GTCATCCAAGTTGCATATTTT	143	AAG	AAGAAGAAGAAGAAGAAGAAG	21
Locus_3421_Transcript_8/29_Confidence_0.429_Length_1397	REVERSE	54.81	42.86	AGTTTGGCATCTTCCTCTAGT				
Locus_3428_Transcript_25/50_Confidence_0.458_Length_1306	FORWARD	54.79	47.62	CCTCTTCTTCCCAGAAACTAC	149	CTC	CTCCTCCTCCTCCTCCTCCTC	21
Locus_3428_Transcript_25/50_Confidence_0.458_Length_1306	REVERSE	54.16	33.33	GAAGCAAACAGAAGAAAAGAA				
Locus_3428_Transcript_25/50_Confidence_0.458_Length_1306	FORWARD	55.26	42.86	CACACAGCTGTTAATTTAGCC	150	CTT	CTTCTTCTTCTTCTTCTT	18
Locus_3428_Transcript_25/50_Confidence_0.458_Length_1306	REVERSE	54.08	38.1	TGGATTCCATAGAAGAGAGAA				
Locus_3428_Transcript_25/50_Confidence_0.458_Length_1306	FORWARD	55.56	33.33	ACCATCAATGTTGAAGAACAA	162	TCT	TCTTCTTCTTCTTCTTCTTCTTCT	24
Locus_3428_Transcript_25/50_Confidence_0.458_Length_1306	REVERSE	54.08	38.1	TGGATTCCATAGAAGAGAGAA				
Locus_3489_Transcript_1/8_Confidence_0.632_Length_2642	FORWARD	54.55	38.1	TCTTGAACATAAACAGGGAAG	148	AAC	AACAACAACAACAACAAC	18
Locus_3489_Transcript_1/8_Confidence_0.632_Length_2642	REVERSE	55.12	40	ACCTTCACATCACCAAAAAG				
Locus_3510_Transcript_22/30_Confidence_0.617_Length_1724	FORWARD	55.15	33.33	TAATGCGAAACCCTATGTAAA	145	AGA	AGAAGAAGAAGAAGAAGA	18
Locus_3510_Transcript_22/30_Confidence_0.617_Length_1724	REVERSE	53.84	28.57	AAAATTTTCTTCTCGAATGTG				
Locus_3575_Transcript_34/54_Confidence_0.581_Length_1836	FORWARD	56.95	50	GTATCCCTGATCTGCTGCTT	147	GCA	GCAGCAGCAGCAGCAGCAGCA	21
Locus_3575_Transcript_34/54_Confidence_0.581_Length_1836	REVERSE	55.34	38.1	ACCGTTGAGATTATTGGAAGT				
Locus_3600_Transcript_10/15_Confidence_0.646_Length_1477	FORWARD	55.65	42.86	ATCTCTGTCGCCTTCACTATT	147	CCT	CCTCCTCCTCCTCCTCCT	18
Locus_3600_Transcript_10/15_Confidence_0.646_Length_1477	REVERSE	54.88	38.1	TTTTGTTCTTCTGATGCTAGG				
Locus_3601_Transcript_12/13_Confidence_0.607_Length_2230	FORWARD	53.66	42.86	CTTCAGGTGTTGTGTGAGATA	152	TAT	TATTATTATTATTATTATTATTAT	24
Locus_3601_Transcript_12/13_Confidence_0.607_Length_2230	REVERSE	55.17	42.86	CACCGAATACACTCTTCACAT				
Locus_3601_Transcript_12/13_Confidence_0.607_Length_2230	FORWARD	54.24	33.33	TCCTTGCTGATTTCTGTTATT	153	TAT	TATTATTATTATTATTATTATTAT	24

Locus_3601_Transcript_12/13_Confidence_0.607_Length_2230	REVERSE	54.7	28.57	ACCAACATCAATTTACCAAAA				
Locus_3647_Transcript_17/28_Confidence_0.605_Length_1346	FORWARD	56.28	33.33	TTTTTCTTGAATCCCATGAGT	155	TGC	TGCTGCTGCTGCTGCTGC	18
Locus_3647_Transcript_17/28_Confidence_0.605_Length_1346	REVERSE	58.54	47.62	ATGAGGAACTTGCAGGAAGAG				
Locus_3661_Transcript_5/6_Confidence_0.609_Length_1175	FORWARD	54.39	45.45	GAAGTGTCTTCCTCTTCTCAGT	148	GAG	GAGGAGGAGGAGGAGGAGGAG	21
Locus_3661_Transcript_5/6_Confidence_0.609_Length_1175	REVERSE	54.63	28.57	CTCAAAATCTCCATTGAAAAA				
Locus_3725_Transcript_3/7_Confidence_0.750_Length_2829	FORWARD	56.47	52.38	GAGAGAGATGGAGAGGGAGAT	156	GGA	GGAGGAGGAGGAGGAGGA	18
Locus_3725_Transcript_3/7_Confidence_0.750_Length_2829	REVERSE	55.05	38.1	TGTGCCTTGTAGTGATTTTCT				
Locus_3752_Transcript_31/48_Confidence_0.360_Length_3874	FORWARD	56.16	33.33	AACTATTTGCCAATGAACCAT	151	GAA	GAAGAAGAAGAAGAAGAA	18
Locus_3752_Transcript_31/48_Confidence_0.360_Length_3874	REVERSE	55.66	42.86	GTTTAGTGGGATAAAGGCTTG				
Locus_3754_Transcript_6/19_Confidence_0.573_Length_909	FORWARD	55.32	42.86	AGTCTGAAGCTTCTTCTTGCT	154	TCC	TCCTCCTCCTCCTCCTCCTCCTCC	24
Locus_3754_Transcript_6/19_Confidence_0.573_Length_909	REVERSE	55.18	47.62	CAATATAGAGGAGGAGCAGGT				
Locus_3771_Transcript_26/28_Confidence_0.588_Length_1545	FORWARD	54.96	36.36	TCATGTAGAATGAGACCTTGAA	148	AAC	AACAACAACAACAACAACAACAAC	24
Locus_3771_Transcript_26/28_Confidence_0.588_Length_1545	REVERSE	55.18	47.62	CGCTGTATTATCTCTGGTGAG				
Locus_3817_Transcript_10/24_Confidence_0.607_Length_1400	FORWARD	55.67	47.62	GAAGTGAAGGTGGAGAAAGAG	140	AGG	AGGAGGAGGAGGAGGAGGAGG	21
Locus_3817_Transcript_10/24_Confidence_0.607_Length_1400	REVERSE	54.81	52.38	GCTGTTACTAGAACCCTCCTC				
Locus_3821_Transcript_71/75_Confidence_0.285_Length_2379	FORWARD	54.76	50	AGTCGCCTTTAGACAGTCAG	173	TCC	TCCTCCTCCTCCTCCTCC	18
Locus_3821_Transcript_71/75_Confidence_0.285_Length_2379	REVERSE	55	33.33	GGAACCAATAAATCACAATGA				
Locus_3828_Transcript_8/18_Confidence_0.621_Length_1816	FORWARD	55.3	42.86	AAGGGAAGTACTTGTTGAAGC	153	CAA	CAACAACAACAACAACAA	18
Locus_3828_Transcript_8/18_Confidence_0.621_Length_1816	REVERSE	55.41	42.86	GAGAGCTTGGTTGAAGTCTTT				
Locus_3853_Transcript_27/28_Confidence_0.562_Length_2467	FORWARD	55.81	60	GAGTAGCGTAGGAGGAGGAG	284	GGA	GGAGGAGGAGGAGGAGGAGGA	21
Locus_3853_Transcript_27/28_Confidence_0.562_Length_2467	REVERSE	54.38	38.1	AGCAAACACATATCGATTACC				
Locus_3858_Transcript_35/49_Confidence_0.508_Length_1774	FORWARD	54.77	33.33	TCATTTGACATTTCAGAGGTT	149	TCT	TCTTCTTCTTCTTCTTCTTCT	21
Locus_3858_Transcript_35/49_Confidence_0.508_Length_1774	REVERSE	55.58	52.38	GTCTCGGGGAAGTAGTTAGTG				
Locus_3858_Transcript_35/49_Confidence_0.508_Length_1774	FORWARD	54.77	33.33	TCATTTGACATTTCAGAGGTT	149	TCT	TCTTCTTCTTCTTCTTCTTCT	21
Locus_3858_Transcript_35/49_Confidence_0.508_Length_1774	REVERSE	55.58	52.38	GTCTCGGGGAAGTAGTTAGTG				
Locus_3897_Transcript_20/27_Confidence_0.605_Length_3408	FORWARD	55.73	42.86	TCATGAAGAAGAGTCGCATAG	151	GAG	GAGGAGGAGGAGGAGGAG	18
Locus_3897_Transcript_20/27_Confidence_0.605_Length_3408	REVERSE	55.14	42.86	AATTGGAGCTCCTCAGTTATC				
Locus_3897_Transcript_20/27_Confidence_0.605_Length_3408	FORWARD	55.73	42.86	TCATGAAGAAGAGTCGCATAG	151	GAG	GAGGAGGAGGAGGAGGAG	18
Locus_3897_Transcript_20/27_Confidence_0.605_Length_3408	REVERSE	55.14	42.86	AATTGGAGCTCCTCAGTTATC				
Locus_3897_Transcript_20/27_Confidence_0.605_Length_3408	FORWARD	55.47	42.86	AATTGACCTCCTCCTCTAACA	169	TCC	TCCTCCTCCTCCTCCTCCTCC	21
Locus_3897_Transcript_20/27_Confidence_0.605_Length_3408	REVERSE	55	47.62	CGATCAGAGAAGCTGAGAGTA				
Locus_3909_Transcript_9/23_Confidence_0.516_Length_854	FORWARD	54.84	28.57	CAGCAAAATTTAAGGAAATGA	155	TTC	TTCTTCTTCTTCTTCTTCTTCTTC	24
Locus_3909_Transcript_9/23_Confidence_0.516_Length_854	REVERSE	55.22	33.33	TTATAAAACATGGTGGAATCG				
Locus_3979_Transcript_10/13_Confidence_0.619_Length_2188	FORWARD	55.19	52.38	GCTGGAGTACTCTCTCCATCT	153	TGC	TGCTGCTGCTGCTGCTGC	18
Locus_3979_Transcript_10/13_Confidence_0.619_Length_2188	REVERSE	55.54	33.33	ATGATGAAGCAGAAGGATTTT				
Locus_4028_Transcript_50/58_Confidence_0.579_Length_5799	FORWARD	55.18	42.86	CCTTCCCTTCTAGGATTAACA	151	TGG	TGGTGGTGGTGGTGGTGG	18
Locus_4028_Transcript_50/58_Confidence_0.579_Length_5799	REVERSE	55.05	38.1	ACCAGAGCACAAAAATCTGTA				
Locus_4093_Transcript_41/96_Confidence_0.356_Length_1989	FORWARD	55.09	33.33	TCACTTTTTGATTGACACACA	147	CTT	CTTCTTCTTCTTCTTCTT	18
Locus_4093_Transcript_41/96_Confidence_0.356_Length_1989	REVERSE	55.13	33.33	AGAAAATTGAAGAATCCAAGG				
Locus_4186_Transcript_21/73_Confidence_0.612_Length_3860	FORWARD	54.82	28.57	AAATTGTCAAGCAATTGTGAT	149	TTA	TTATTATTATTATTATTATTA	21
Locus_4186_Transcript_21/73_Confidence_0.612_Length_3860	REVERSE	55.08	38.1	TCTGGCATGTAAGGAAATCTA				

Locus_4186_Transcript_21/73_Confidence_0.612_Length_3860	FORWARD	54.82	28.57	AAATTGTCAAGCAATTGTGAT	149	TTA	TTATTATTATTATTATTATTA	21
Locus_4186_Transcript_21/73_Confidence_0.612_Length_3860	REVERSE	55.08	38.1	TCTGGCATGTAAGGAAATCTA				
Locus_4282_Transcript_17/34_Confidence_0.623_Length_3440	FORWARD	57.27	50	CTATGCTTCCCACAGAAACC	213	AGA	AGAAGAAGAAGAAGAAGAAGAAGA	24
Locus_4282_Transcript_17/34_Confidence_0.623_Length_3440	REVERSE	55.33	50	CTGTGATCCACCATCCTTAC				
Locus_4282_Transcript_17/34_Confidence_0.623_Length_3440	FORWARD	55.45	38.1	TGGATCACAGGGTGATATAAA	152	AGA	AGAAGAAGAAGAAGAAGAAGA	21
Locus_4282_Transcript_17/34_Confidence_0.623_Length_3440	REVERSE	57.2	50	CCATCTCCATTCCTTGCTAC				
Locus_4321_Transcript_8/25_Confidence_0.544_Length_2935	FORWARD	55.32	38.1	AATAATTCTGAAGGGCAGAAG	166	TTA	TTATTATTATTATTATTATTATTA	24
Locus_4321_Transcript_8/25_Confidence_0.544_Length_2935	REVERSE	54.99	28.57	TGGAACCAAACAAAGATTAAA				
Locus_4321_Transcript_8/25_Confidence_0.544_Length_2935	FORWARD	55.47	38.1	CTCAATTGTTGTGATGCTCTT	151	ATT	ATTATTATTATTATTATTATTATT	24
Locus_4321_Transcript_8/25_Confidence_0.544_Length_2935	REVERSE	55.1	42.86	TGATCGTAAGTTCAGGAGAGA				
Locus_4323_Transcript_6/9_Confidence_0.619_Length_2915	FORWARD	55.03	42.86	AAATTGGGGACTCTACATAGC	158	TCA	TCATCATCATCATCATCATCA	21
Locus_4323_Transcript_6/9_Confidence_0.619_Length_2915	REVERSE	54.92	42.86	TAGAGTCCATCAGTCCAGAAA				
Locus_4323_Transcript_6/9_Confidence_0.619_Length_2915	FORWARD	54.92	42.86	TTTCTGGACTGATGGACTCTA	135	TCA	TCATCATCATCATCATCATCA	21
Locus_4323_Transcript_6/9_Confidence_0.619_Length_2915	REVERSE	54.86	38.1	TAATGAAGATGAAGGGAGTGA				
Locus_4323_Transcript_6/9_Confidence_0.619_Length_2915	FORWARD	55.03	42.86	AAATTGGGGACTCTACATAGC	158	TCA	TCATCATCATCATCATCATCA	21
Locus_4323_Transcript_6/9_Confidence_0.619_Length_2915	REVERSE	54.92	42.86	TAGAGTCCATCAGTCCAGAAA				
Locus_4342_Transcript_36/45_Confidence_0.611_Length_2646	FORWARD	55.55	38.1	CTTCCTCCGTTATTTCAATTC	173	CCG	CCGCCGCCGCCGCCGCCG	18
Locus_4342_Transcript_36/45_Confidence_0.611_Length_2646	REVERSE	54.89	45	AACTCCGGATCCTTATTCTC				
Locus_4356_Transcript_27/35_Confidence_0.594_Length_3718	FORWARD	54.52	42.86	CTATTGTGAAGAAGGAGGTGA	152	AAG	AAGAAGAAGAAGAAGAAGAAGAAG	24
Locus_4356_Transcript_27/35_Confidence_0.594_Length_3718	REVERSE	54.7	42.86	CTGTTACTAACCTGCGTTGAT				
Locus_4375_Transcript_4/9_Confidence_0.675_Length_1663	FORWARD	54.89	47.62	GGAAAGAAGAAGAGAGTGAGG	170	GAG	GAGGAGGAGGAGGAGGAGGAG	21
Locus_4375_Transcript_4/9_Confidence_0.675_Length_1663	REVERSE	55.63	38.1	TAGCAGCGATTAACTTCTCAA				
Locus_4403_Transcript_23/24_Confidence_0.558_Length_1213	FORWARD	55.26	38.1	GACGAAGAAGATGATGATGAA	190	GGA	GGAGGAGGAGGAGGAGGA	18
Locus_4403_Transcript_23/24_Confidence_0.558_Length_1213	REVERSE	54.78	38.1	TGGGCAAGTACATAAGAAAAG				
Locus_4410_Transcript_32/60_Confidence_0.374_Length_4715	FORWARD	54.54	47.62	TTCTATAGTCAGCCTCCACAC	148	TAT	TATTATTATTATTATTAT	18
Locus_4410_Transcript_32/60_Confidence_0.374_Length_4715	REVERSE	55.18	33.33	TTATTGGGCTACAAAGTCAAA				
Locus_4444_Transcript_8/16_Confidence_0.536_Length_2224	FORWARD	54.96	38.1	AGATTGATGACATGAGAATGG	165	AGA	AGAAGAAGAAGAAGAAGA	18
Locus_4444_Transcript_8/16_Confidence_0.536_Length_2224	REVERSE	55.75	42.86	CCTCGAAGAATGAAAGAAGAC				
Locus_4585_Transcript_24/28_Confidence_0.622_Length_3769	FORWARD	54.51	42.86	TATCATCATCACTGTCCTCCT	149	TCC	TCCTCCTCCTCCTCCTCC	18
Locus_4585_Transcript_24/28_Confidence_0.622_Length_3769	REVERSE	55.42	42.86	AGAAATTGGCGGTAGAGTAAG				
Locus_4585_Transcript_24/28_Confidence_0.622_Length_3769	FORWARD	55.01	42.86	ATCCTTTCTGTCTTCCTTGTC	160	TCC	TCCTCCTCCTCCTCCTCCTCCTCC	24
Locus_4585_Transcript_24/28_Confidence_0.622_Length_3769	REVERSE	54.63	42.86	CCAATTCTTGCTGTACAGAGT				
Locus_4644_Transcript_20/25_Confidence_0.604_Length_2555	FORWARD	55.46	52.38	GGAGACCTAGTCTCTCAATGG	140	TCT	TCTTCTTCTTCTTCTTCT	18
Locus_4644_Transcript_20/25_Confidence_0.604_Length_2555	REVERSE	55.5	38.1	GATCTCGTGCTTTTTCTTCTT				
Locus_4738_Transcript_98/111_Confidence_0.419_Length_2232	FORWARD	55.62	47.62	TACCTCGCCTATCTCCACTAT	131	AAT	AATAATAATAATAATAATAAT	21
Locus_4738_Transcript_98/111_Confidence_0.419_Length_2232	REVERSE	54.01	42.86	GATGATCTAGTACGCAAATCC				
Locus_4746_Transcript_21/30_Confidence_0.599_Length_2853	FORWARD	55.22	38.1	AACTGATGTCATCGAATTGAG	175	TAT	TATTATTATTATTATTATTATTAT	24
Locus_4746_Transcript_21/30_Confidence_0.599_Length_2853	REVERSE	54.95	36.36	AGGGTCATAAGCAAGATAAAGA				
Locus_4754_Transcript_34/54_Confidence_0.536_Length_1939	FORWARD	55.16	42.86	ATACTAGTGGGTGGGGATAAA	152	TTA	TTATTATTATTATTATTATTA	21
Locus_4754_Transcript_34/54_Confidence_0.536_Length_1939	REVERSE	54.93	42.86	GGTATGGAGGAAGGAGAATTA				
Locus_4766_Transcript_9/14_Confidence_0.698_Length_4334	FORWARD	54.98	50	GCAAGTATACCACCAAGCTC	220	CTC	CTCCTCCTCCTCCTCCTCCTC	21

Locus_4766_Transcript_9/14_Confidence_0.698_Length_4334	REVERSE	54.41	47.62	CGCTACAGTCTTAGGTTTCAC				
Locus_4825_Transcript_13/24_Confidence_0.607_Length_1069	FORWARD	55.5	38.1	ACCAGCTTCTACATTTCCAAT	153	CTC	CTCCTCCTCCTCCTCCTCCTCCTC	24
Locus_4825_Transcript_13/24_Confidence_0.607_Length_1069	REVERSE	55.14	31.82	AAGATCTTCATTGATCCTTTTG				
Locus_5009_Transcript_45/50_Confidence_0.587_Length_2313	FORWARD	55.85	38.1	CAATTCGATACGATTTGAGTG	167	ACG	ACGACGACGACGACGACG	18
Locus_5009_Transcript_45/50_Confidence_0.587_Length_2313	REVERSE	54.97	42.86	GCAACCATACATGAGAAGAAG				
Locus_5019_Transcript_11/106_Confidence_0.346_Length_2575	FORWARD	54.46	38.1	TGTGCGACATCTGTATTAGAA	155	AAG	AAGAAGAAGAAGAAGAAGAAGAAG	24
Locus_5019_Transcript_11/106_Confidence_0.346_Length_2575	REVERSE	54.2	38.1	TCGTTTAACAGCTTCTTCTTC				
Locus_5026_Transcript_17/40_Confidence_0.512_Length_2477	FORWARD	55.01	38.1	CCTCAAAACCTTGGATATTCT	173	CCT	CCTCCTCCTCCTCCTCCTCCT	21
Locus_5026_Transcript_17/40_Confidence_0.512_Length_2477	REVERSE	54.67	33.33	AATTAAGAATGTGGTGGTGAA				
Locus_5026_Transcript_17/40_Confidence_0.512_Length_2477	FORWARD	54.97	33.33	TTCTTAATTGGGACTGTTTCA	151	CTT	CTTCTTCTTCTTCTTCTT	18
Locus_5026_Transcript_17/40_Confidence_0.512_Length_2477	REVERSE	56.6	45	CCGTGGAGGATAATTTGAGT				
Locus_5026_Transcript_17/40_Confidence_0.512_Length_2477	FORWARD	55.23	38.1	TTCTTCTTTCTCTCGACATCA	169	CTT	CTTCTTCTTCTTCTTCTT	18
Locus_5026_Transcript_17/40_Confidence_0.512_Length_2477	REVERSE	55.03	33.33	CTGCATAAATCCTGCTTAAAA				
Locus_5112_Transcript_18/57_Confidence_0.343_Length_3924	FORWARD	55.32	38.1	TGTAACCAATGAAGTTCCAAG	171	TGA	TGATGATGATGATGATGA	18
Locus_5112_Transcript_18/57_Confidence_0.343_Length_3924	REVERSE	54.82	38.1	TCTCCAAGTCCTTTACCTTTT				
Locus_5135_Transcript_7/12_Confidence_0.640_Length_1727	FORWARD	54.36	33.33	ATGGTATTTTCGTCATTTCAG	168	GGC	GGCGGCGGCGGCGGCGGC	18
Locus_5135_Transcript_7/12_Confidence_0.640_Length_1727	REVERSE	55.2	42.86	ATGAGCCAGAACTAAATAGGG				
Locus_5195_Transcript_7/8_Confidence_0.705_Length_2732	FORWARD	54.68	47.62	CACAGAAGAGGAGTCAGAAGA	150	ATG	ATGATGATGATGATGATGATG	21
Locus_5195_Transcript_7/8_Confidence_0.705_Length_2732	REVERSE	55.1	33.33	TTTCCAGGCAATATTTACAGA				
Locus_5207_Transcript_5/15_Confidence_0.614_Length_1161	FORWARD	54.81	38.1	GTATGCCATTTGTATTCCTTG	150	TGA	TGATGATGATGATGATGA	18
Locus_5207_Transcript_5/15_Confidence_0.614_Length_1161	REVERSE	55.59	42.86	TGTCCAGATTGTAAGCTATGC				
Locus_5313_Transcript_7/12_Confidence_0.736_Length_3452	FORWARD	54.58	38.1	AACATGTGCTTAATCCTCTTG	158	CCT	CCTCCTCCTCCTCCTCCT	18
Locus_5313_Transcript_7/12_Confidence_0.736_Length_3452	REVERSE	54.86	38.1	TGACTTTAATCTCCATCCTGA				
Locus_5343_Transcript_4/7_Confidence_0.643_Length_1275	FORWARD	55.25	42.86	AGGAACAGCAGCACAATACTA	177	TAC	TACTACTACTACTACTAC	18
Locus_5343_Transcript_4/7_Confidence_0.643_Length_1275	REVERSE	54.67	52.38	GAGTAGGAAACCCAGGAGTAG				
Locus_5362_Transcript_31/46_Confidence_0.596_Length_6554	FORWARD	54.93	42.86	TGTGTGTCATCAGGTACAGAA	161	CCA	CCACCACCACCACCACCACCACCA	24
Locus_5362_Transcript_31/46_Confidence_0.596_Length_6554	REVERSE	54.92	42.86	GTAATGCTGCTGAGTAATGCT				
Locus_5364_Transcript_17/29_Confidence_0.592_Length_6988	FORWARD	55.07	38.1	CCTCCATTTCATCATCATCTA	147	CTC	CTCCTCCTCCTCCTCCTC	18
Locus_5364_Transcript_17/29_Confidence_0.592_Length_6988	REVERSE	54.78	40	GTGTTGCTGTTGAGTTTGAA				
Locus_5370_Transcript_25/48_Confidence_0.579_Length_2130	FORWARD	55	42.86	ACTTGCAGTCTCCTTCTTCTT	158	TCT	TCTTCTTCTTCTTCTTCT	18
Locus_5370_Transcript_25/48_Confidence_0.579_Length_2130	REVERSE	55.1	42.86	CAAACTTGTCTCAGAAACCTG				
Locus_5442_Transcript_13/39_Confidence_0.553_Length_2271	FORWARD	55.8	47.62	ATCGAGATGAGGAGATCTGAC	156	CTC	CTCCTCCTCCTCCTCCTCCTC	21
Locus_5442_Transcript_13/39_Confidence_0.553_Length_2271	REVERSE	56.02	42.86	AAAGAGATGGGAGGAAGAGAT				
Locus_5512_Transcript_29/57_Confidence_0.534_Length_2884	FORWARD	55.77	38.1	AACTGAAACCAAAGCAGAGTT	160	TCT	TCTTCTTCTTCTTCTTCTTCTTCT	24
Locus_5512_Transcript_29/57_Confidence_0.534_Length_2884	REVERSE	53.86	42.86	CGACCCAATATCTCTGATAAC				
Locus_5512_Transcript_29/57_Confidence_0.534_Length_2884	FORWARD	55.16	40	TGAAACCAAAGCAGAGTCTT	173	CTT	CTTCTTCTTCTTCTTCTTCTTCTT	24
Locus_5512_Transcript_29/57_Confidence_0.534_Length_2884	REVERSE	55.04	38.1	CTTTCGTCTGCAAATCTCTAA				
Locus_5594_Transcript_10/24_Confidence_0.455_Length_1278	FORWARD	55.44	33.33	ATGATTAAACGGAAACCCTAA	146	TTC	TTCTTCTTCTTCTTCTTCTTC	21
Locus_5594_Transcript_10/24_Confidence_0.455_Length_1278	REVERSE	54.22	42.86	TTGGTAGAGAGAAAGACAGGA				
Locus_5594_Transcript_10/24_Confidence_0.455_Length_1278	FORWARD	55.57	38.1	ACTGAACTTAGGGTTTTGCAT	171	GAA	GAAGAAGAAGAAGAAGAAGAA	21
Locus_5594_Transcript_10/24_Confidence_0.455_Length_1278	REVERSE	55.34	47.62	CAGCCACGTTACTTCTGTAAG				

Locus_5613_Transcript_10/13_Confidence_0.358_Length_1444	FORWARD	54.95	38.1	ATCAAAACTCCATCTTTCTCC	146	TCT	TCTTCTTCTTCTTCTTCTTCTTCTTCTTCTTC	36
Locus_5613_Transcript_10/13_Confidence_0.358_Length_1444	REVERSE	54.37	38.1	TCTAATATACACACGGTGCAA				
Locus_5622_Transcript_14/22_Confidence_0.630_Length_1717	FORWARD	55.36	38.1	TGCAGGTAAAAAGAGTTCAGA	150	CGC	CGCCGCCGCCGCCGCCGC	18
Locus_5622_Transcript_14/22_Confidence_0.630_Length_1717	REVERSE	55.26	38.1	AAGACGACTTTTTCCTTTGTC				
Locus_5627_Transcript_11/24_Confidence_0.629_Length_2835	FORWARD	54.99	33.33	TGCAATCTTTCACAAACCTAT	154	CAA	CAACAACAACAACAACAA	18
Locus_5627_Transcript_11/24_Confidence_0.629_Length_2835	REVERSE	54.75	28.57	TTTGCATGATTTTGTTGTATG				
Locus_5680_Transcript_11/53_Confidence_0.475_Length_600	FORWARD	54.93	47.62	CAGATGGACCTATGCTATGAG	149	CTT	CTTCTTCTTCTTCTTCTTCTT	21
Locus_5680_Transcript_11/53_Confidence_0.475_Length_600	REVERSE	54.96	38.1	TCTTAGTTTGTTCTGCTTTGG				
Locus_5746_Transcript_38/47_Confidence_0.645_Length_4236	FORWARD	54.52	38.1	GATTATTGGGCTACAAAGTCA	147	AAT	AATAATAATAATAATAATAATAAT	24
Locus_5746_Transcript_38/47_Confidence_0.645_Length_4236	REVERSE	54.96	38.1	AGCGTGATATAGTTTTTGCTG				
Locus_5810_Transcript_16/22_Confidence_0.621_Length_1695	FORWARD	51.72	40	AGGTATACAAGGAATCATGC	129	AAT	AATAATAATAATAATAAT	18
Locus_5810_Transcript_16/22_Confidence_0.621_Length_1695	REVERSE	54.54	47.62	GTGAGGAGAGCTTGTATGGTA				
Locus_5836_Transcript_9/16_Confidence_0.635_Length_2136	FORWARD	54.95	38.1	CCTTTGTTCTCATTTCATCAG	198	AAG	AAGAAGAAGAAGAAGAAG	18
Locus_5836_Transcript_9/16_Confidence_0.635_Length_2136	REVERSE	55.48	38.1	GATGAAGAAATGTTCCATGTG				
Locus_5843_Transcript_4/15_Confidence_0.656_Length_1635	FORWARD	54.28	38.1	AGTGTCCAGATTAAAGAGCAA	145	TCA	TCATCATCATCATCATCATCA	21
Locus_5843_Transcript_4/15_Confidence_0.656_Length_1635	REVERSE	54.48	42.86	CTTAAAGCTAGGGGATGATCT				
Locus_5844_Transcript_7/23_Confidence_0.598_Length_2089	FORWARD	54.86	38.1	TAATTTGAAGCACTCTTCGTC	149	AGA	AGAAGAAGAAGAAGAAGAAGAAGA	24
Locus_5844_Transcript_7/23_Confidence_0.598_Length_2089	REVERSE	54.67	38.1	CAGACAATAAAAACACCCATC				
Locus_5853_Transcript_10/28_Confidence_0.664_Length_2945	FORWARD	55	38.1	CACATCCAAATCTTCGTAAAG	147	TCT	TCTTCTTCTTCTTCTTCT	18
Locus_5853_Transcript_10/28_Confidence_0.664_Length_2945	REVERSE	55.02	38.1	ATGATATTGGTCTCCCTTCAT				
Locus_5891_Transcript_20/28_Confidence_0.603_Length_2255	FORWARD	56.38	38.1	TGCTGAAGCAGAAAGTTCATA	191	GGC	GGCGGCGGCGGCGGCGGCGGC	21
Locus_5891_Transcript_20/28_Confidence_0.603_Length_2255	REVERSE	55.29	47.62	GTGGAAGCCTTGTCTCTACTT				
Locus_5931_Transcript_12/22_Confidence_0.654_Length_1580	FORWARD	54.99	40	TGTTTCGTCCTCAGAAGTTT	148	TCT	TCTTCTTCTTCTTCTTCT	18
Locus_5931_Transcript_12/22_Confidence_0.654_Length_1580	REVERSE	54.89	36.36	CAAGTAAACGAAGAAGACAACA				
Locus_5949_Transcript_14/56_Confidence_0.585_Length_5646	FORWARD	55.01	38.1	CCCATCTTCAGGTTTCTTATT	139	CTG	CTGCTGCTGCTGCTGCTGCTGCTG	24
Locus_5949_Transcript_14/56_Confidence_0.585_Length_5646	REVERSE	54.93	35	ACAAAATCATGCCATATTCC				
Locus_5985_Transcript_6/7_Confidence_0.762_Length_1791	FORWARD	55.53	47.62	GTATTTTAGTGGCGGACCTAC	134	GGC	GGCGGCGGCGGCGGCGGCGGC	21
Locus_5985_Transcript_6/7_Confidence_0.762_Length_1791	REVERSE	54.81	38.1	AAAGGATTTAGTACCGCTCAT				
Locus_5994_Transcript_13/31_Confidence_0.555_Length_2021	FORWARD	55.08	38.1	TGAGTAGGAGAATATGGCAAA	178	AGA	AGAAGAAGAAGAAGAAGAAGAAGA	24
Locus_5994_Transcript_13/31_Confidence_0.555_Length_2021	REVERSE	55.06	38.1	TGATTCTTCTTCTTCTGGTGA				
Locus_6009_Transcript_5/12_Confidence_0.655_Length_1634	FORWARD	55.04	47.62	GACTTCCTGCTCTTTCTCTTC	152	ATC	ATCATCATCATCATCATC	18
Locus_6009_Transcript_5/12_Confidence_0.655_Length_1634	REVERSE	54.32	38.1	GACTTTGAATCTCGTTGTGAT				
Locus_6048_Transcript_2/8_Confidence_0.724_Length_6756	FORWARD	54.92	42.86	ATGGAAGAGTTTCCTGATCTC	153	CTT	CTTCTTCTTCTTCTTCTT	18
Locus_6048_Transcript_2/8_Confidence_0.724_Length_6756	REVERSE	55.3	38.1	ACAGGAACCAATTTCTCTCAT				
Locus_6052_Transcript_17/31_Confidence_0.578_Length_2060	FORWARD	54.78	47.62	GTGTACATCTCTTCCTCATCG	146	GAA	GAAGAAGAAGAAGAAGAA	18
Locus_6052_Transcript_17/31_Confidence_0.578_Length_2060	REVERSE	55.18	42.86	CCCTTTCTGTCTCATCTTCTT				
Locus_6071_Transcript_4/6_Confidence_0.690_Length_899	FORWARD	55.63	38.1	AGATTTGGGTTCAATCATCTC	164	CAC	CACCACCACCACCACCAC	18
Locus_6071_Transcript_4/6_Confidence_0.690_Length_899	REVERSE	55.18	47.62	TGGTGGAGTTGGTAGTGATAG				
Locus_6095_Transcript_18/40_Confidence_0.498_Length_1644	FORWARD	54.75	42.86	CTGTATTTAGCAGGCTTTACG	122	GAA	GAAGAAGAAGAAGAAGAAGAAGAA	24
Locus_6095_Transcript_18/40_Confidence_0.498_Length_1644	REVERSE	55.6	42.86	GCTGGTTTAACAGCTTCTTCT				
Locus_6138_Transcript_12/33_Confidence_0.633_Length_2617	FORWARD	55.18	42.86	CAATTCCTTCCTCTTCACTCT	151	CCA	CCACCACCACCACCACCACCACCA	24

Locus_6138_Transcript_12/33_Confidence_0.633_Length_2617	REVERSE	54.67	33.33	GAATGAAAGTTTTCGAACAGA				
Locus_6175_Transcript_13/15_Confidence_0.605_Length_1116	FORWARD	55.62	38.1	TTTTACTATCCTGGCGGTTAT	139	GGC	GGCGGCGGCGGCGGCGGCGGC	21
Locus_6175_Transcript_13/15_Confidence_0.605_Length_1116	REVERSE	55.46	47.62	GACAGGTTCGAGAGGTAATTC				
Locus_6222_Transcript_28/90_Confidence_0.394_Length_1403	FORWARD	55.04	42.86	CGACCCTTCTATTTGGTCTAT	181	CTC	CTCCTCCTCCTCCTCCTC	18
Locus_6222_Transcript_28/90_Confidence_0.394_Length_1403	REVERSE	55	45	GATGCCATCATCATCTCTCT				
Locus_6223_Transcript_2/4_Confidence_0.846_Length_1056	FORWARD	55.72	47.62	TTCTCACAGTCGTTACTCTCG	198	GCA	GCAGCAGCAGCAGCAGCAGCA	21
Locus_6223_Transcript_2/4_Confidence_0.846_Length_1056	REVERSE	55.27	42.86	AAAGGAGGTTATGTAGCAAGG				
Locus_6223_Transcript_2/4_Confidence_0.846_Length_1056	FORWARD	54.16	42.86	TCCTTTCTCACAGTCGTTACT	150	GCA	GCAGCAGCAGCAGCAGCAGCA	21
Locus_6223_Transcript_2/4_Confidence_0.846_Length_1056	REVERSE	55.09	33.33	CAATTATTCGAAATCAACTGC				
Locus_6238_Transcript_2/12_Confidence_0.598_Length_1434	FORWARD	55.04	47.62	GTCTTGAAGGTAGTCGGAGAT	129	CCG	CCGCCGCCGCCGCCGCCGCCG	21
Locus_6238_Transcript_2/12_Confidence_0.598_Length_1434	REVERSE	55.11	50	GACGCAGCCGATATACTAAC				
Locus_6266_Transcript_6/12_Confidence_0.642_Length_1038	FORWARD	55.45	50	TCTTACTCCACACCTGAAGC	220	CCG	CCGCCGCCGCCGCCGCCGCCG	21
Locus_6266_Transcript_6/12_Confidence_0.642_Length_1038	REVERSE	54.23	38.1	ATGACGATATTTCATCCTCTG				
Locus_6315_Transcript_24/36_Confidence_0.555_Length_1383	FORWARD	55.18	47.62	GAGGAGGAAGAAGAAGTGGTA	150	GAA	GAAGAAGAAGAAGAAGAAGAA	21
Locus_6315_Transcript_24/36_Confidence_0.555_Length_1383	REVERSE	55.1	42.86	GAGCTATGGCATCAATGTAAG				
Locus_6363_Transcript_13/22_Confidence_0.619_Length_4555	FORWARD	54.61	45	ACCTTTGTTAACACGCTCTC	153	TTC	TTCTTCTTCTTCTTCTTC	18
Locus_6363_Transcript_13/22_Confidence_0.619_Length_4555	REVERSE	54.93	28.57	AATTCAAGCAAAATATGGTGA				
Locus_6408_Transcript_32/64_Confidence_0.513_Length_3008	FORWARD	54.82	42.86	GTCGGTGAAGCAAGTAACTAA	165	CTC	CTCCTCCTCCTCCTCCTC	18
Locus_6408_Transcript_32/64_Confidence_0.513_Length_3008	REVERSE	55.15	42.86	GTATCAAAGGGGAGAATCATC				
Locus_6472_Transcript_22/39_Confidence_0.527_Length_3761	FORWARD	55.52	42.86	CTCTTGTTACCACGAACGATA	179	AAC	AACAACAACAACAACAAC	18
Locus_6472_Transcript_22/39_Confidence_0.527_Length_3761	REVERSE	54.93	38.1	GGTATTGTGGTTGTTGTTGTT				
Locus_6547_Transcript_16/21_Confidence_0.646_Length_2117	FORWARD	55.06	42.86	GCTTGAAGAATACGCTGTAGA	146	GAG	GAGGAGGAGGAGGAGGAGGAG	21
Locus_6547_Transcript_16/21_Confidence_0.646_Length_2117	REVERSE	55.23	42.86	TTCTTCATCTTTCCCTCTCTC				
Locus_6559_Transcript_48/60_Confidence_0.470_Length_8761	FORWARD	54.45	33.33	ATTGAACAACACCTTGATGAT	146	TGA	TGATGATGATGATGATGA	18
Locus_6559_Transcript_48/60_Confidence_0.470_Length_8761	REVERSE	54.72	33.33	CCAGCTAAAATCATTAAACCA				
Locus_6604_Transcript_6/7_Confidence_0.724_Length_1387	FORWARD	55.14	38.1	TCAAAGTTCTCCTTGTCTTCA	161	TTC	TTCTTCTTCTTCTTCTTC	18
Locus_6604_Transcript_6/7_Confidence_0.724_Length_1387	REVERSE	54.8	42.86	GTGGTAATCCGAGATCCTAAT				
Locus_6635_Transcript_24/32_Confidence_0.585_Length_1789	FORWARD	55.25	47.62	AGCTACAGTTACCGAGGATTC	140	GAT	GATGATGATGATGATGAT	18
Locus_6635_Transcript_24/32_Confidence_0.585_Length_1789	REVERSE	54.82	42.86	GTCGATTTCTTCTTCTTCCTC				
Locus_6635_Transcript_24/32_Confidence_0.585_Length_1789	FORWARD	55.01	33.33	AAAAATCGAGGAAGAAGAAGA	129	GAT	GATGATGATGATGATGAT	18
Locus_6635_Transcript_24/32_Confidence_0.585_Length_1789	REVERSE	54.68	33.33	TTTTAGATTTTGGATCCCTCT				
Locus_6635_Transcript_24/32_Confidence_0.585_Length_1789	FORWARD	55.25	47.62	AGCTACAGTTACCGAGGATTC	140	GAT	GATGATGATGATGATGAT	18
Locus_6635_Transcript_24/32_Confidence_0.585_Length_1789	REVERSE	54.82	42.86	GTCGATTTCTTCTTCTTCCTC				
Locus_6681_Transcript_60/94_Confidence_0.567_Length_2134	FORWARD	55.04	33.33	GATTTGCTTGAAACAGCTTTA	158	ATC	ATCATCATCATCATCATC	18
Locus_6681_Transcript_60/94_Confidence_0.567_Length_2134	REVERSE	54.94	33.33	AGGTTGAAAGGAAGAAGAAAA				
Locus_6745_Transcript_8/8_Confidence_0.561_Length_631	FORWARD	55.05	42.86	CTCAAACAAATAGAGCACACC	219	GAG	GAGGAGGAGGAGGAGGAGGAGGAG	24
Locus_6745_Transcript_8/8_Confidence_0.561_Length_631	REVERSE	56.3	55	ATAACCTCCTCCTCCTCCTC				
Locus_6745_Transcript_8/8_Confidence_0.561_Length_631	FORWARD	55.49	50	AAGAAGAAGCAGAGGAGGAG	102	GAG	GAGGAGGAGGAGGAGGAG	18
Locus_6745_Transcript_8/8_Confidence_0.561_Length_631	REVERSE	56.17	38.1	AACTCGACGACAAAAACTTTC				
Locus_6785_Transcript_16/34_Confidence_0.602_Length_1881	FORWARD	55.07	47.62	TCGGATCTCTCTCCTATTACC	144	TGC	TGCTGCTGCTGCTGCTGCTGC	21
Locus_6785_Transcript_16/34_Confidence_0.602_Length_1881	REVERSE	55.17	38.1	AAAGTGGGTAAGGAAAACAAG				

Locus_6786_Transcript_3/26_Confidence_0.534_Length_1862	FORWARD	55.39	38.1	ATCCAATACCCAACCATTTAC	153	TCT	TCTTCTTCTTCTTCTTCT	18
Locus_6786_Transcript_3/26_Confidence_0.534_Length_1862	REVERSE	55.16	38.1	AATTAAACCAGAATCGAGAGG				
Locus_6799_Transcript_8/24_Confidence_0.595_Length_2930	FORWARD	55.05	33.33	TTAGAAAAGATGGTTCGATGA	152	TGT	TGTTGTTGTTGTTGTTGTTGT	21
Locus_6799_Transcript_8/24_Confidence_0.595_Length_2930	REVERSE	54.86	38.1	ACCATTACCAATAACAGAGCA				
Locus_6808_Transcript_11/23_Confidence_0.609_Length_2763	FORWARD	55.22	42.86	AGCATAGAAGGAAAGTCCAAC	162	TGA	TGATGATGATGATGATGA	18
Locus_6808_Transcript_11/23_Confidence_0.609_Length_2763	REVERSE	55.27	38.1	CACCTTTTCTTCCTTTCAACT				
Locus_6841_Transcript_15/25_Confidence_0.523_Length_4256	FORWARD	56.38	47.62	CCTCTCACCCCCTAACTAAAT	172	CCT	CCTCCTCCTCCTCCTCCT	18
Locus_6841_Transcript_15/25_Confidence_0.523_Length_4256	REVERSE	55.6	40.91	CCATGAACTGAGGAATATAAGC				
Locus_6852_Transcript_8/13_Confidence_0.633_Length_5223	FORWARD	54.91	42.86	AGAGATGATAGAGGGATTTGG	154	GAG	GAGGAGGAGGAGGAGGAGGAG	21
Locus_6852_Transcript_8/13_Confidence_0.633_Length_5223	REVERSE	54.38	42.86	ACCTAAGGCGGTATATTTCTC				
Locus_6878_Transcript_14/16_Confidence_0.641_Length_2274	FORWARD	54.62	38.1	TCTACAATTTCTGAGGATTGC	152	CCT	CCTCCTCCTCCTCCTCCT	18
Locus_6878_Transcript_14/16_Confidence_0.641_Length_2274	REVERSE	54.99	33.33	ACAGAAGCAAATGGTGAAATA				
Locus_6879_Transcript_28/29_Confidence_0.631_Length_2738	FORWARD	55.37	47.62	GTAACTGACGAACCTGTGAGA	161	GCC	GCCGCCGCCGCCGCCGCC	18
Locus_6879_Transcript_28/29_Confidence_0.631_Length_2738	REVERSE	54.14	40	CCAAATCCTCTCATAAAAGC				
Locus_6947_Transcript_8/14_Confidence_0.633_Length_1806	FORWARD	55.51	38.1	GGAAAAACAGAAAACAGGAAC	149	TAA	TAATAATAATAATAATAA	18
Locus_6947_Transcript_8/14_Confidence_0.633_Length_1806	REVERSE	55.39	38.1	GAAGCCATCACTTTAATCACA				
Locus_6964_Transcript_13/48_Confidence_0.635_Length_1667	FORWARD	54.7	38.1	GTAGCCTTGTGAGAAAATCAA	153	AAG	AAGAAGAAGAAGAAGAAGAAGAAGAAG	27
Locus_6964_Transcript_13/48_Confidence_0.635_Length_1667	REVERSE	54.93	38.1	CCATCGTTTAACAGCTAAGAA				
Locus_7086_Transcript_4/9_Confidence_0.686_Length_596	FORWARD	55.29	45.45	CGTTCTCTCTTCTTATCTCTCG	157	TTA	TTATTATTATTATTATTATTATTA	24
Locus_7086_Transcript_4/9_Confidence_0.686_Length_596	REVERSE	54.59	38.1	AGGGGGATTCAATGTAATATC				
Locus_7119_Transcript_6/9_Confidence_0.706_Length_1661	FORWARD	54.77	38.1	TTAACTTTTAGGCACGTCATC	153	TTG	TTGTTGTTGTTGTTGTTG	18
Locus_7119_Transcript_6/9_Confidence_0.706_Length_1661	REVERSE	55.05	33.33	AACAACAACAACAACAAATCC				
Locus_7119_Transcript_6/9_Confidence_0.706_Length_1661	FORWARD	55.11	42.86	CACACATAGTGGGATTAAGGA	146	TTG	TTGTTGTTGTTGTTGTTG	18
Locus_7119_Transcript_6/9_Confidence_0.706_Length_1661	REVERSE	54.74	33.33	TTGAAGAAATACACCTTTTCG				
Locus_7139_Transcript_12/14_Confidence_0.600_Length_1290	FORWARD	55.42	38.1	AAGAAGAAGAAGAGGGGTTTT	155	GAG	GAGGAGGAGGAGGAGGAG	18
Locus_7139_Transcript_12/14_Confidence_0.600_Length_1290	REVERSE	55.29	28.57	AAAAATCAAATCGGAAAAGAG				
Locus_7183_Transcript_2/8_Confidence_0.571_Length_665	FORWARD	54.1	45	CAGCATCCTTAGGGTTTTAC	152	CGG	CGGCGGCGGCGGCGGCGG	18
Locus_7183_Transcript_2/8_Confidence_0.571_Length_665	REVERSE	55.78	47.62	CTTCCCTACTGAGAGCAAATC				
Locus_7251_Transcript_12/29_Confidence_0.433_Length_1559	FORWARD	55.59	38.1	GGCTTGCTTAATGTTGATGTA	132	GAT	GATGATGATGATGATGATGAT	21
Locus_7251_Transcript_12/29_Confidence_0.433_Length_1559	REVERSE	55.77	38.1	ATCAACAACATCATCATCGTC				
Locus_7279_Transcript_15/40_Confidence_0.289_Length_1748	FORWARD	54.56	38.1	CTTGATAACGCCATCATAGTT	160	TTC	TTCTTCTTCTTCTTCTTC	18
Locus_7279_Transcript_15/40_Confidence_0.289_Length_1748	REVERSE	55.51	38.1	CAAGCCAAAGAAGAAGAAGAT				
Locus_7280_Transcript_3/5_Confidence_0.875_Length_1604	FORWARD	59.79	40	ACAAACCGCAAAGCAAAACT	175	TTC	TTCTTCTTCTTCTTCTTC	18
Locus_7280_Transcript_3/5_Confidence_0.875_Length_1604	REVERSE	57	40.91	GGTTTGTGGATATTGTTGAGTG				
Locus_7282_Transcript_33/41_Confidence_0.525_Length_2349	FORWARD	54.82	42.86	CTACATTGAGAATCACCCTTG	159	ATA	ATAATAATAATAATAATA	18
Locus_7282_Transcript_33/41_Confidence_0.525_Length_2349	REVERSE	54.65	28.57	TGCTTCTATAAAATTGCGTTT				
Locus_7291_Transcript_13/15_Confidence_0.589_Length_1688	FORWARD	54.39	47.62	TGTGTCTCCTACTCTTCAACC	157	TTC	TTCTTCTTCTTCTTCTTC	18
Locus_7291_Transcript_13/15_Confidence_0.589_Length_1688	REVERSE	55.18	33.33	AGCAAAAAGTGAATCAGTCAA				
Locus_7304_Transcript_13/31_Confidence_0.457_Length_719	FORWARD	54.76	42.86	TGTAGCTTCTTTTCAGGTGAC	187	GCG	GCGGCGGCGGCGGCGGCGGCGGCG	24
Locus_7304_Transcript_13/31_Confidence_0.457_Length_719	REVERSE	53.75	31.82	CTTTGTCAAATTCTAAAACTGC				
Locus_7330_Transcript_23/24_Confidence_0.638_Length_1973	FORWARD	56.93	33.33	ATCCATCATTGCGTTATCAAT	145	GAC	GACGACGACGACGACGAC	18

Locus_7330_Transcript_23/24_Confidence_0.638_Length_1973	REVERSE	55.07	42.86	TAGGGTTACCTTTGGATTAGG				
Locus_7358_Transcript_11/19_Confidence_0.361_Length_2039	FORWARD	54.08	42.86	GCCCTTTTTCCTCTTCTATAC	197	CCT	CCTCCTCCTCCTCCTCCTCCT	21
Locus_7358_Transcript_11/19_Confidence_0.361_Length_2039	REVERSE	55.12	47.62	TAGAGGAGGCCATAGGAATAC				
Locus_7381_Transcript_10/26_Confidence_0.615_Length_2685	FORWARD	55.65	40	CTCAAAAGAACATGGGAATG	140	TTC	TTCTTCTTCTTCTTCTTC	18
Locus_7381_Transcript_10/26_Confidence_0.615_Length_2685	REVERSE	53.93	42.86	CATGAGAAGAAGAGGAATCAC				
Locus_7414_Transcript_36/37_Confidence_0.586_Length_2435	FORWARD	54.96	47.62	GGCTCTATAATCGGCTCTATC	137	CTG	CTGCTGCTGCTGCTGCTGCTG	21
Locus_7414_Transcript_36/37_Confidence_0.586_Length_2435	REVERSE	57.42	45	CTAAAGCAGCAGGCTTTGAT				
Locus_7426_Transcript_9/20_Confidence_0.638_Length_2976	FORWARD	55.92	42.86	ACTCTTACCCATCGCAAATAC	151	AGA	AGAAGAAGAAGAAGAAGA	18
Locus_7426_Transcript_9/20_Confidence_0.638_Length_2976	REVERSE	54.67	42.86	GCACTAACCTCATCTTCTTCA				
Locus_7426_Transcript_9/20_Confidence_0.638_Length_2976	FORWARD	55.92	42.86	ACTCTTACCCATCGCAAATAC	151	AGA	AGAAGAAGAAGAAGAAGA	18
Locus_7426_Transcript_9/20_Confidence_0.638_Length_2976	REVERSE	54.67	42.86	GCACTAACCTCATCTTCTTCA				
Locus_7519_Transcript_15/46_Confidence_0.603_Length_1387	FORWARD	55	47.62	GAAGGTAAGGTAAAGCAGGAG	141	TTC	TTCTTCTTCTTCTTCTTC	18
Locus_7519_Transcript_15/46_Confidence_0.603_Length_1387	REVERSE	55.36	38.1	TGGCTACAAGTTGAAGAAGAA				
Locus_7679_Transcript_20/21_Confidence_0.586_Length_1272	FORWARD	59.08	50	ACGGCGTCTAAAGAAGAGGA	172	GAG	GAGGAGGAGGAGGAGGAGGAG	21
Locus_7679_Transcript_20/21_Confidence_0.586_Length_1272	REVERSE	56.76	42.86	GCGATCATCTTAAAACAGAGC				
Locus_7693_Transcript_21/31_Confidence_0.602_Length_3346	FORWARD	55.33	33.33	TTGAAGAAATAGGGGATGAAT	150	GAA	GAAGAAGAAGAAGAAGAA	18
Locus_7693_Transcript_21/31_Confidence_0.602_Length_3346	REVERSE	54.66	33.33	TGTTATTTGCTGTGCAATCTA				
Locus_7717_Transcript_7/13_Confidence_0.597_Length_1519	FORWARD	55.01	50	GAGGAGTTGATGTGGAGGTA	168	GAG	GAGGAGGAGGAGGAGGAGGAG	21
Locus_7717_Transcript_7/13_Confidence_0.597_Length_1519	REVERSE	55.37	50	GTAAATCTCCGTCCTCTTCC				
Locus_7769_Transcript_17/58_Confidence_0.528_Length_1302	FORWARD	54.31	38.1	AGTGAATGAAGGTAATCATGC	148	CAG	CAGCAGCAGCAGCAGCAG	18
Locus_7769_Transcript_17/58_Confidence_0.528_Length_1302	REVERSE	54.91	45	GAGCATAGGAAGAAGAAGCA				
Locus_7797_Transcript_5/12_Confidence_0.530_Length_1068	FORWARD	55.72	45	ACCTACTTCGCCAAAACTCT	158	CCT	CCTCCTCCTCCTCCTCCTCCT	21
Locus_7797_Transcript_5/12_Confidence_0.530_Length_1068	REVERSE	54.17	38.1	AAGAAGAGGAAAGAAGAGGAA				
Locus_7798_Transcript_16/33_Confidence_0.327_Length_1309	FORWARD	54.86	47.62	CAGCGTCTACTTCTCAACTGT	150	CAG	CAGCAGCAGCAGCAGCAG	18
Locus_7798_Transcript_16/33_Confidence_0.327_Length_1309	REVERSE	55.87	38.1	TGCTATGCATGTAATTGTCCT				
Locus_7807_Transcript_17/19_Confidence_0.603_Length_1588	FORWARD	54.67	33.33	ATCAAACAAACCTTACATCCA	142	AAT	AATAATAATAATAATAATAAT	21
Locus_7807_Transcript_17/19_Confidence_0.603_Length_1588	REVERSE	55.08	33.33	TCTGAAGAAATTTCAGTTCCA				
Locus_7846_Transcript_21/42_Confidence_0.541_Length_1987	FORWARD	55.27	47.62	CACTTCCCTATCCCATTCTAC	169	GGA	GGAGGAGGAGGAGGAGGAGGAGGA	24
Locus_7846_Transcript_21/42_Confidence_0.541_Length_1987	REVERSE	54.64	38.1	TCACCACATATCTTTCCACTT				
Locus_7848_Transcript_6/22_Confidence_0.462_Length_1523	FORWARD	54.87	42.86	TCTCTCTCCCTATTTTCGTCT	151	AAG	AAGAAGAAGAAGAAGAAGAAG	21
Locus_7848_Transcript_6/22_Confidence_0.462_Length_1523	REVERSE	54.94	33.33	TCTATTTCCCATCGTTTAACA				
Locus_7852_Transcript_9/29_Confidence_0.503_Length_936	FORWARD	55.1	42.86	CTGGATCTCCTCAGATTTTCT	153	GAA	GAAGAAGAAGAAGAAGAAGAAGAA	24
Locus_7852_Transcript_9/29_Confidence_0.503_Length_936	REVERSE	54.67	38.1	CAGACAATAAAAACACCCATC				
Locus_7862_Transcript_17/29_Confidence_0.592_Length_2303	FORWARD	56.26	45	CAAAGCTTTCTTCCTCAGGT	200	CGC	CGCCGCCGCCGCCGCCGC	18
Locus_7862_Transcript_17/29_Confidence_0.592_Length_2303	REVERSE	54.62	38.1	GAAGGAAATTCAGACATAGCA				
Locus_7879_Transcript_6/15_Confidence_0.627_Length_2840	FORWARD	55.92	42.86	GCCTGAACCGTTAGTATTCAT	146	CGG	CGGCGGCGGCGGCGGCGGCGG	21
Locus_7879_Transcript_6/15_Confidence_0.627_Length_2840	REVERSE	55	38.1	AATCTCAACTCGAAACACTCA				
Locus_7907_Transcript_33/39_Confidence_0.489_Length_2945	FORWARD	54.75	38.1	CCCACAAACAAAAGTGTATTC	153	GCC	GCCGCCGCCGCCGCCGCC	18
Locus_7907_Transcript_33/39_Confidence_0.489_Length_2945	REVERSE	55.05	42.86	TAGATCTTTGATGGGTGTGTC				
Locus_7909_Transcript_15/21_Confidence_0.538_Length_2474	FORWARD	57.35	55	CTCCACCTCATCGAACTCTC	120	CTC	CTCCTCCTCCTCCTCCTC	18
Locus_7909_Transcript_15/21_Confidence_0.538_Length_2474	REVERSE	54.82	42.86	GGATTGGTCTTCATAGGACTT				

Locus_7928_Transcript_14/15_Confidence_0.590_Length_1182	FORWARD	54.65	42.86	ATAAGAGGAAGAAGGCTCAAG	151	TCC	TCCTCCTCCTCCTCCTCCTCC	21
Locus_7928_Transcript_14/15_Confidence_0.590_Length_1182	REVERSE	55.65	42.86	CATGATGTCGAGATTGAGTTC				
Locus_7956_Transcript_23/40_Confidence_0.444_Length_1735	FORWARD	55.01	38.1	AAGAATCCCTCATTAAACCAG	166	CTT	CTTCTTCTTCTTCTTCTTCTT	21
Locus_7956_Transcript_23/40_Confidence_0.444_Length_1735	REVERSE	55.01	38.1	TCATAATCTGCTTTAGGCTTG				
Locus_8081_Transcript_1/9_Confidence_0.644_Length_2237	FORWARD	54.67	50	CCTAAGAGTCTGACGATGCT	223	AGG	AGGAGGAGGAGGAGGAGG	18
Locus_8081_Transcript_1/9_Confidence_0.644_Length_2237	REVERSE	55.43	33.33	AGCATAAACCCATCATTTTCT				
Locus_8081_Transcript_1/9_Confidence_0.644_Length_2237	FORWARD	55.43	33.33	AGAAAATGATGGGTTTATGCT	286	AGG	AGGAGGAGGAGGAGGAGG	18
Locus_8081_Transcript_1/9_Confidence_0.644_Length_2237	REVERSE	56.18	50	TACAACCTCCTCCTCCTCAT				
Locus_8104_Transcript_15/28_Confidence_0.557_Length_1784	FORWARD	54.3	28.57	AAGCAAATTTCTGCACATTAT	155	TCT	TCTTCTTCTTCTTCTTCT	18
Locus_8104_Transcript_15/28_Confidence_0.557_Length_1784	REVERSE	54.92	38.1	GATTTAAAGTTTGGGGACAGT				
Locus_8119_Transcript_23/30_Confidence_0.497_Length_787	FORWARD	54.51	47.62	GGTCTCCTTGTTCACATACAC	138	CTT	CTTCTTCTTCTTCTTCTTCTTCTT	24
Locus_8119_Transcript_23/30_Confidence_0.497_Length_787	REVERSE	55.01	42.86	CAAGGACAGAAGAACATTGAG				
Locus_8126_Transcript_16/21_Confidence_0.643_Length_916	FORWARD	54.86	33.33	GAAGCACCTAAAATAATGCAA	160	AAT	AATAATAATAATAATAATAAT	21
Locus_8126_Transcript_16/21_Confidence_0.643_Length_916	REVERSE	54.92	38.1	TCCATCCTCGTAAACTCATTA				
Locus_8130_Transcript_17/28_Confidence_0.593_Length_2985	FORWARD	55.24	40	ATCTCTCTGCAACGAAAGAA	149	CTT	CTTCTTCTTCTTCTTCTTCTT	21
Locus_8130_Transcript_17/28_Confidence_0.593_Length_2985	REVERSE	55.04	33.33	ACAATACGCAAAATACCAAGA				
Locus_8225_Transcript_5/9_Confidence_0.758_Length_2188	FORWARD	55.01	38.1	CAAATCTCCTAAAGGTTGGAT	149	TCC	TCCTCCTCCTCCTCCTCC	18
Locus_8225_Transcript_5/9_Confidence_0.758_Length_2188	REVERSE	55	38.1	GAAAAGGATGTAACTCGGATT				
Locus_8236_Transcript_2/10_Confidence_0.609_Length_619	FORWARD	55.19	38.1	TCATCTATTCCTGCTCTTTGA	140	CCT	CCTCCTCCTCCTCCTCCT	18
Locus_8236_Transcript_2/10_Confidence_0.609_Length_619	REVERSE	54.92	42.86	AGGAGGAGAAATGAATCAGAC				
Locus_8236_Transcript_2/10_Confidence_0.609_Length_619	FORWARD	54.16	47.62	GATAAACCCTAGTGAGTGCTG	154	CTC	CTCCTCCTCCTCCTCCTCCTC	21
Locus_8236_Transcript_2/10_Confidence_0.609_Length_619	REVERSE	54.92	42.86	AGGAGGAGAAATGAATCAGAC				
Locus_8347_Transcript_17/25_Confidence_0.595_Length_1741	FORWARD	55.23	42.86	GGGACTAAAAATCATCACCTC	168	TCA	TCATCATCATCATCATCA	18
Locus_8347_Transcript_17/25_Confidence_0.595_Length_1741	REVERSE	55.11	42.86	ACAGATTCAAGACACCTGATG				
Locus_8425_Transcript_6/17_Confidence_0.542_Length_5486	FORWARD	54.59	38.1	TGAAGAGCTTACTGAAAGGAA	144	TTC	TTCTTCTTCTTCTTCTTCTTCTTC	24
Locus_8425_Transcript_6/17_Confidence_0.542_Length_5486	REVERSE	55.22	42.86	GAAGAAGAAGAAGAACGAAGC				
Locus_8425_Transcript_6/17_Confidence_0.542_Length_5486	FORWARD	54.59	38.1	TGAAGAGCTTACTGAAAGGAA	135	TTC	TTCTTCTTCTTCTTCTTCTTCTTC	24
Locus_8425_Transcript_6/17_Confidence_0.542_Length_5486	REVERSE	55.7	33.33	TAACAACAACAAAAACGAAGC				
Locus_8425_Transcript_6/17_Confidence_0.542_Length_5486	FORWARD	55.7	33.33	GCTTCGTTTTTGTTGTTGTTA	159	TTC	TTCTTCTTCTTCTTCTTC	18
Locus_8425_Transcript_6/17_Confidence_0.542_Length_5486	REVERSE	56.75	35	AATGAGCTTTCGCAAGAAAT				
Locus_8425_Transcript_6/17_Confidence_0.542_Length_5486	FORWARD	54.34	31.82	TTTGTTGAGATTAATGCTCTGT	149	TTC	TTCTTCTTCTTCTTCTTC	18
Locus_8425_Transcript_6/17_Confidence_0.542_Length_5486	REVERSE	55.67	45.45	CCCTCTCTCCATCCTTATAACT				
Locus_8704_Transcript_7/12_Confidence_0.615_Length_1013	FORWARD	55.87	38.1	GGGGTTGTTGAAAAGATTAGA	150	TCT	TCTTCTTCTTCTTCTTCT	18
Locus_8704_Transcript_7/12_Confidence_0.615_Length_1013	REVERSE	55.01	42.86	AGAACAAGAACAGGGAGATTC				
Locus_8728_Transcript_2/8_Confidence_0.690_Length_2219	FORWARD	55.02	42.86	AGTTGACACACAGAACCAATC	148	CGG	CGGCGGCGGCGGCGGCGG	18
Locus_8728_Transcript_2/8_Confidence_0.690_Length_2219	REVERSE	55.36	38.1	AATAAGCTCGGGAGTATTTTG				
Locus_8740_Transcript_2/14_Confidence_0.492_Length_789	FORWARD	54.73	38.1	GCCACCTTTATTAGAAAATCC	159	GAA	GAAGAAGAAGAAGAAGAAGAAGAA	24
Locus_8740_Transcript_2/14_Confidence_0.492_Length_789	REVERSE	55.09	47.62	TTAAGCTCTCCTCACTCCTCT				
Locus_8789_Transcript_2/13_Confidence_0.588_Length_1083	FORWARD	54.62	38.1	ACGGAATATCCACTCATGTTA	140	GCG	GCGGCGGCGGCGGCGGCG	18
Locus_8789_Transcript_2/13_Confidence_0.588_Length_1083	REVERSE	55.23	33.33	ATCTCCATTCTTTGTCCATTT				
Locus_8793_Transcript_15/15_Confidence_0.553_Length_2240	FORWARD	54.84	33.33	CTGAAGAAAATCTTCGAAACA	159	CCT	CCTCCTCCTCCTCCTCCTCCT	21

Locus_8793_Transcript_15/15_Confidence_0.553_Length_2240	REVERSE	54.32	33.33	AAAGAAACCAGCTTGTGATTA				
Locus_8826_Transcript_7/17_Confidence_0.597_Length_817	FORWARD	54.91	38.1	ATATCGTTTCAACTGCTGTGT	158	TTA	TTATTATTATTATTATTA	18
Locus_8826_Transcript_7/17_Confidence_0.597_Length_817	REVERSE	54.75	24	TGCAAATAAAAAGTAAACTACAAGA				
Locus_8843_Transcript_4/26_Confidence_0.654_Length_2211	FORWARD	54.97	42.86	AACCACTAACCTTATCCCAAG	154	TCC	TCCTCCTCCTCCTCCTCCTCCTCC	24
Locus_8843_Transcript_4/26_Confidence_0.654_Length_2211	REVERSE	55.67	47.62	TAAGCTTCTTCAGAGGAGGAG				
Locus_8843_Transcript_4/26_Confidence_0.654_Length_2211	FORWARD	54.64	33.33	CCAAAATCTCCAATAGCATTA	154	TCC	TCCTCCTCCTCCTCCTCCTCCTCC	24
Locus_8843_Transcript_4/26_Confidence_0.654_Length_2211	REVERSE	54.58	33.33	TTGGTGAATGTTTCCTATGAT				
Locus_8959_Transcript_22/28_Confidence_0.622_Length_2579	FORWARD	54.94	45.45	CCGTACTCCTTACTCTTTTGTC	147	CCT	CCTCCTCCTCCTCCTCCT	18
Locus_8959_Transcript_22/28_Confidence_0.622_Length_2579	REVERSE	54.91	42.86	TGAATGAGCTCGTAGTAAAGG				
Locus_8964_Transcript_22/39_Confidence_0.597_Length_2344	FORWARD	54.8	38.1	GTTCAGATGAGAAGAAAGCAA	147	AAG	AAGAAGAAGAAGAAGAAGAAGAAGAAG	27
Locus_8964_Transcript_22/39_Confidence_0.597_Length_2344	REVERSE	55.07	33.33	TTGGTTGCACAGTTTTTCTAT				
Locus_8964_Transcript_22/39_Confidence_0.597_Length_2344	FORWARD	55.35	42.86	TCAGGATTCTGTTCAGATGAG	147	GAA	GAAGAAGAAGAAGAAGAAGAAGAA	24
Locus_8964_Transcript_22/39_Confidence_0.597_Length_2344	REVERSE	55.07	33.33	TTGGTTGCACAGTTTTTCTAT				
Locus_9032_Transcript_32/34_Confidence_0.557_Length_2832	FORWARD	55.15	42.86	CCATGAAACAGATAAGCAGAG	148	AGA	AGAAGAAGAAGAAGAAGA	18
Locus_9032_Transcript_32/34_Confidence_0.557_Length_2832	REVERSE	55.49	42.86	AGCGTTACTTGTTCACACAAC				
Locus_9057_Transcript_4/10_Confidence_0.729_Length_3372	FORWARD	54.96	42.86	AAAGAGCATACTGCACAGAAG	148	ACC	ACCACCACCACCACCACC	18
Locus_9057_Transcript_4/10_Confidence_0.729_Length_3372	REVERSE	54.85	33.33	ACCAGTTCATCAAAGTTGAAA				
Locus_9096_Transcript_17/19_Confidence_0.670_Length_1388	FORWARD	55.12	28.57	CCCAAAAAGGTTTTAATTTGT	162	ATT	ATTATTATTATTATTATT	18
Locus_9096_Transcript_17/19_Confidence_0.670_Length_1388	REVERSE	54.94	42.86	CAGAGAGCTTGAAGTTGGTAA				
Locus_9101_Transcript_29/42_Confidence_0.592_Length_1655	FORWARD	55.59	38.1	ACTCCAAATATCATCGGATTC	202	GGC	GGCGGCGGCGGCGGCGGC	18
Locus_9101_Transcript_29/42_Confidence_0.592_Length_1655	REVERSE	55.64	42.86	TTCTCTTGATGTCGAAGTACG				
Locus_9197_Transcript_5/7_Confidence_0.435_Length_693	FORWARD	54.82	38.1	GAATTAGCCAATTTCTCTTCC	149	CTC	CTCCTCCTCCTCCTCCTC	18
Locus_9197_Transcript_5/7_Confidence_0.435_Length_693	REVERSE	55.92	47.62	GAGAAGGAGATGAGCAAGAAG				
Locus_9197_Transcript_5/7_Confidence_0.435_Length_693	FORWARD	55.18	42.86	CTTCTTCCTTCTGGTTCATCT	148	CTC	CTCCTCCTCCTCCTCCTC	18
Locus_9197_Transcript_5/7_Confidence_0.435_Length_693	REVERSE	55.23	42.86	TATGATGAAGTGGAGGTTGAG				
Locus_9197_Transcript_5/7_Confidence_0.435_Length_693	FORWARD	53.8	38.1	TCGTCTACATTCTGAACATTG	151	CTC	CTCCTCCTCCTCCTCCTC	18
Locus_9197_Transcript_5/7_Confidence_0.435_Length_693	REVERSE	55.71	42.86	ATGAACCAGAAGGAGATGAAC				
Locus_9245_Transcript_28/53_Confidence_0.583_Length_1841	FORWARD	54.57	42.86	GGGTTACTCCTCAATTTCTTC	117	CAG	CAGCAGCAGCAGCAGCAG	18
Locus_9245_Transcript_28/53_Confidence_0.583_Length_1841	REVERSE	54.76	38.1	TTCATCTGGTTAACCTCCATA				
Locus_9397_Transcript_19/28_Confidence_0.594_Length_1395	FORWARD	54.67	38.1	AAGAGATCAAAGCATTGACAG	138	TTC	TTCTTCTTCTTCTTCTTC	18
Locus_9397_Transcript_19/28_Confidence_0.594_Length_1395	REVERSE	55.21	33.33	GCCAATGATGTAAATTTGGTA				
Locus_9502_Transcript_8/19_Confidence_0.651_Length_1315	FORWARD	54.72	33.33	TCATCCACCATAGCAATAAAT	152	TAT	TATTATTATTATTATTAT	18
Locus_9502_Transcript_8/19_Confidence_0.651_Length_1315	REVERSE	56.1	42.86	AAATAAAGAGAGGTCGGGAGT				
Locus_9647_Transcript_11/15_Confidence_0.600_Length_602	FORWARD	56.87	50	ATATAGTCAGGCTGCGATCC	151	CCG	CCGCCGCCGCCGCCGCCGCCGCCG	24
Locus_9647_Transcript_11/15_Confidence_0.600_Length_602	REVERSE	59.5	40	TTCCCAATGGCTCTTGAAAT				
Locus_9706_Transcript_4/11_Confidence_0.667_Length_594	FORWARD	54.59	45	AAGGATGCTTTACGTACGAC	219	CGC	CGCCGCCGCCGCCGCCGC	18
Locus_9706_Transcript_4/11_Confidence_0.667_Length_594	REVERSE	54.81	45	GTCATCATCTTCTTCCTTCG				
Locus_9706_Transcript_4/11_Confidence_0.667_Length_594	FORWARD	56.96	50	GAAGGAAGAATCACCACCAC	131	CGC	CGCCGCCGCCGCCGCCGC	18
Locus_9706_Transcript_4/11_Confidence_0.667_Length_594	REVERSE	56.44	38.1	GAAAACCAGCCGTTAATTATG				
Locus_9722_Transcript_13/14_Confidence_0.704_Length_515	FORWARD	55.35	42.86	TGAAGATGAGATGGAGAGTTG	144	TTC	TTCTTCTTCTTCTTCTTCTTCTTC	24
Locus_9722_Transcript_13/14_Confidence_0.704_Length_515	REVERSE	54.9	33.33	CACCTCAAAGCAATCTAAAAA				

Locus_9722_Transcript_13/14_Confidence_0.704_Length_515	FORWARD	55.08	38.1	AATCCTCAGATTCCTATTTGC	153	CTT	CTTCTTCTTCTTCTTCTTCTTCTT	24
Locus_9722_Transcript_13/14_Confidence_0.704_Length_515	REVERSE	54.94	47.62	GAAAGGGAAGAGTAGAAGCAC				
Locus_9769_Transcript_12/24_Confidence_0.556_Length_1914	FORWARD	55.63	42.86	ACCAGATCCAATCTCTGATTC	150	GAA	GAAGAAGAAGAAGAAGAA	18
Locus_9769_Transcript_12/24_Confidence_0.556_Length_1914	REVERSE	54.37	38.1	GTTTTCGCATTCTATATGCTC				
Locus_9835_Transcript_2/6_Confidence_0.667_Length_1538	FORWARD	55.07	45	ATGAAGGTATTCGGTGAGTG	155	GGT	GGTGGTGGTGGTGGTGGT	18
Locus_9835_Transcript_2/6_Confidence_0.667_Length_1538	REVERSE	54.86	47.62	GCACCTTATCTCTCTTCCTTC				
Locus_10098_Transcript_9/10_Confidence_0.607_Length_2122	FORWARD	55.05	38.1	AGACACCTTAATGTTTGCTGA	204	TAA	TAATAATAATAATAATAA	18
Locus_10098_Transcript_9/10_Confidence_0.607_Length_2122	REVERSE	55.05	38.1	AAGTTTGTAGCAGTTCCATCA				
Locus_10151_Transcript_4/7_Confidence_0.776_Length_1997	FORWARD	54.43	42.86	TATTAGTGGGGTGAACTCTTG	150	GCC	GCCGCCGCCGCCGCCGCC	18
Locus_10151_Transcript_4/7_Confidence_0.776_Length_1997	REVERSE	55.06	45	GTGCCCATCAGAAATAGTGT				
Locus_10204_Transcript_4/6_Confidence_0.775_Length_1334	FORWARD	55.46	42.86	CTCCCTCTGCTTGTTTACTTT	164	ATT	ATTATTATTATTATTATT	18
Locus_10204_Transcript_4/6_Confidence_0.775_Length_1334	REVERSE	54.72	33.33	TAAGCCCCCATAATATTTCTT				
Locus_10236_Transcript_8/13_Confidence_0.579_Length_854	FORWARD	54.84	33.33	AATCGGTACAGGCTTTAAAAT	150	AAG	AAGAAGAAGAAGAAGAAG	18
Locus_10236_Transcript_8/13_Confidence_0.579_Length_854	REVERSE	55.32	42.86	CATCTTCCCCAATCTCTTATC				
Locus_10311_Transcript_5/8_Confidence_0.615_Length_1555	FORWARD	54.88	42.86	GCTCATCAGAACTATCATTGG	158	CAT	CATCATCATCATCATCAT	18
Locus_10311_Transcript_5/8_Confidence_0.615_Length_1555	REVERSE	54.86	38.1	TTAAAAAGGAGGAGCTAAAGG				
Locus_10349_Transcript_13/16_Confidence_0.647_Length_1660	FORWARD	54.79	47.62	GGGATCACTAGACTCTCCAAT	144	GAG	GAGGAGGAGGAGGAGGAG	18
Locus_10349_Transcript_13/16_Confidence_0.647_Length_1660	REVERSE	55.04	47.62	ATCCTCGTACTCTTCTTCCAC				
Locus_10349_Transcript_13/16_Confidence_0.647_Length_1660	FORWARD	54.72	33.33	CATATTTGCCAAAGAAGGTTA	148	ATG	ATGATGATGATGATGATG	18
Locus_10349_Transcript_13/16_Confidence_0.647_Length_1660	REVERSE	54.76	35	ATTTCTTCTTCATTGCAAGC				
Locus_10415_Transcript_7/11_Confidence_0.548_Length_1005	FORWARD	54.19	36.36	ACCAAGAAGGTTCTAGAAGAAA	140	AGA	AGAAGAAGAAGAAGAAGAAGAAGA	24
Locus_10415_Transcript_7/11_Confidence_0.548_Length_1005	REVERSE	54.88	42.86	CTATCAGCATCATTGTCCTTC				
Locus_10454_Transcript_27/28_Confidence_0.568_Length_1300	FORWARD	55.08	47.62	AGAGGGGTATGGTAATAGCTG	165	AAT	AATAATAATAATAATAAT	18
Locus_10454_Transcript_27/28_Confidence_0.568_Length_1300	REVERSE	54.67	38.1	CATTCCTTGGTTATTTGTGTC				
Locus_10519_Transcript_2/3_Confidence_0.778_Length_1629	FORWARD	55.66	47.62	TCTCTATTCGTCGGTAGTCCT	148	TTA	TTATTATTATTATTATTATTA	21
Locus_10519_Transcript_2/3_Confidence_0.778_Length_1629	REVERSE	54.03	33.33	TAAGATGTTCATTTGCTTGTG				
Locus_10585_Transcript_9/10_Confidence_0.634_Length_1058	FORWARD	54.99	33.33	TCTTGATCAGAAAAATCCTCA	149	TCT	TCTTCTTCTTCTTCTTCTTCTTCT	24
Locus_10585_Transcript_9/10_Confidence_0.634_Length_1058	REVERSE	54.61	33.33	AAAAAGGTTTGCAGTATGATG				
Locus_10594_Transcript_29/32_Confidence_0.576_Length_1473	FORWARD	54.98	50	CTTCAGTTCCTGTTCTCCTG	150	AAG	AAGAAGAAGAAGAAGAAGAAG	21
Locus_10594_Transcript_29/32_Confidence_0.576_Length_1473	REVERSE	54.93	38.1	CCATCGTTTAACAGCTAAGAA				
Locus_10627_Transcript_9/11_Confidence_0.625_Length_2738	FORWARD	55.42	38.1	ATGCTGATTATGTTGCAGAAC	152	AGA	AGAAGAAGAAGAAGAAGA	18
Locus_10627_Transcript_9/11_Confidence_0.625_Length_2738	REVERSE	54.9	42.86	CACATTTCTTCTGGTTAGTGG				
Locus_10665_Transcript_14/16_Confidence_0.592_Length_1869	FORWARD	55.37	42.86	AGATCTCCGTCTCATCTTCAT	165	GGC	GGCGGCGGCGGCGGCGGC	18
Locus_10665_Transcript_14/16_Confidence_0.592_Length_1869	REVERSE	54.75	42.86	TACATGCCTTACCTTGAGAAG				
Locus_10668_Transcript_3/25_Confidence_0.531_Length_2174	FORWARD	55.02	38.1	TGCTCAGCAAATCTTAACTTC	160	AAG	AAGAAGAAGAAGAAGAAGAAGAAG	24
Locus_10668_Transcript_3/25_Confidence_0.531_Length_2174	REVERSE	54.67	38.1	CAGACAATAAAAACACCCATC				
Locus_10694_Transcript_11/16_Confidence_0.595_Length_1920	FORWARD	55.35	38.1	GAATTTGTTGATGACGAGAGA	145	CTT	CTTCTTCTTCTTCTTCTT	18
Locus_10694_Transcript_11/16_Confidence_0.595_Length_1920	REVERSE	54.65	42.86	ACCAGAAGAAGAAGAAGAAGC				
Locus_10694_Transcript_11/16_Confidence_0.595_Length_1920	FORWARD	55.08	50	CACTATGGTTCCCCTGTCTA	151	CTT	CTTCTTCTTCTTCTTCTT	18
Locus_10694_Transcript_11/16_Confidence_0.595_Length_1920	REVERSE	54.99	47.62	ACTCTCCTTCATCAACTCCTC				
Locus_10710_Transcript_16/17_Confidence_0.578_Length_2306	FORWARD	55.26	45	AGTTGAGCACAATCTTGACC	143	GCG	GCGGCGGCGGCGGCGGCGGCGGCG	24

Locus_10710_Transcript_16/17_Confidence_0.578_Length_2306	REVERSE	55.47	42.86	TCATGGCAAGTAGTCAGAAAC				
Locus_10737_Transcript_14/32_Confidence_0.552_Length_1297	FORWARD	55.07	38.1	TGAGGATGATGATCTTAATGG	151	GAT	GATGATGATGATGATGAT	18
Locus_10737_Transcript_14/32_Confidence_0.552_Length_1297	REVERSE	54.47	33.33	ACAATCCTAAATGCTTTGCTA				
Locus_10816_Transcript_8/16_Confidence_0.671_Length_1024	FORWARD	55.6	42.86	GTAGAGCTTCCAAAGCTGTTT	175	CTT	CTTCTTCTTCTTCTTCTTCTT	21
Locus_10816_Transcript_8/16_Confidence_0.671_Length_1024	REVERSE	55.31	47.62	CTCAGAGCCTTTTACTTAGCC				
Locus_10874_Transcript_1/6_Confidence_0.632_Length_920	FORWARD	55.73	42.86	AAGAAGTAAGGGGTGTTGCTA	159	GTG	GTGGTGGTGGTGGTGGTG	18
Locus_10874_Transcript_1/6_Confidence_0.632_Length_920	REVERSE	55.62	33.33	ATCCAAAAGAAAGCTCATGTT				
Locus_10936_Transcript_11/12_Confidence_0.649_Length_2074	FORWARD	54.44	38.1	TTGATATTACCAGGAATCTGC	164	AGC	AGCAGCAGCAGCAGCAGC	18
Locus_10936_Transcript_11/12_Confidence_0.649_Length_2074	REVERSE	54.11	47.62	AGTAATCCTCTCCTCCTTCAC				
Locus_11004_Transcript_4/7_Confidence_0.667_Length_877	FORWARD	55.11	22.73	AAAATGTTGTGCAATTTTGTAA	143	ATA	ATAATAATAATAATAATA	18
Locus_11004_Transcript_4/7_Confidence_0.667_Length_877	REVERSE	55.16	36.36	TGAGATTGAAGAAGACTGTGAA				
Locus_11033_Transcript_29/35_Confidence_0.407_Length_2500	FORWARD	54.75	42.86	AGAGAAAGGACTGATCGAAGT	148	CTT	CTTCTTCTTCTTCTTCTT	18
Locus_11033_Transcript_29/35_Confidence_0.407_Length_2500	REVERSE	55.12	42.86	GCAGCTTCAATGTCTGTTATC				
Locus_11132_Transcript_11/12_Confidence_0.652_Length_1500	FORWARD	54.6	38.1	ATCATAACCGAAAAGAGAGGT	163	GGT	GGTGGTGGTGGTGGTGGT	18
Locus_11132_Transcript_11/12_Confidence_0.652_Length_1500	REVERSE	54.79	45	AACAGATCTGGCACGTAGAT				
Locus_11388_Transcript_2/12_Confidence_0.573_Length_2001	FORWARD	54.97	42.86	CTTACAGATCAGCAATCCAAC	147	AGA	AGAAGAAGAAGAAGAAGAAGA	21
Locus_11388_Transcript_2/12_Confidence_0.573_Length_2001	REVERSE	54.78	33.33	TTTACCTCCAATTATGTTCCA				
Locus_11388_Transcript_2/12_Confidence_0.573_Length_2001	FORWARD	55.42	38.1	AGAATCTGGCTGGAACATAAT	149	AGA	AGAAGAAGAAGAAGAAGAAGAAGA	24
Locus_11388_Transcript_2/12_Confidence_0.573_Length_2001	REVERSE	54.2	42.86	ACAAACACTTTGGTGTAGGTC				
Locus_11397_Transcript_2/6_Confidence_0.625_Length_287	FORWARD	54.55	42.86	CTAAAAGAACTGCTTCTGGTG	149	TTA	TTATTATTATTATTATTA	18
Locus_11397_Transcript_2/6_Confidence_0.625_Length_287	REVERSE	55.29	47.62	AGGCACTCAAACCTCTCTTAC				
Locus_11442_Transcript_1/9_Confidence_0.648_Length_1057	FORWARD	53.8	38.1	TTTGTGTTGAGATCTGAAGTG	170	CGG	CGGCGGCGGCGGCGGCGG	18
Locus_11442_Transcript_1/9_Confidence_0.648_Length_1057	REVERSE	55.48	52.38	AGTCTCTACGCTTACGACTCC				
Locus_11474_Transcript_1/4_Confidence_0.833_Length_912	FORWARD	54.57	33.33	TCACACAATTCAATTTCACAG	144	CAA	CAACAACAACAACAACAACAACAA	24
Locus_11474_Transcript_1/4_Confidence_0.833_Length_912	REVERSE	55.43	38.1	CATCGTAGATTGCAAGTGATT				
Locus_11539_Transcript_3/13_Confidence_0.706_Length_850	FORWARD	55.32	35	ATCCCATCTTTCCCTAAAAA	150	CCT	CCTCCTCCTCCTCCTCCTCCT	21
Locus_11539_Transcript_3/13_Confidence_0.706_Length_850	REVERSE	55.05	42.86	TTGCAGAGAGAAAAGAGTCTG				
Locus_11539_Transcript_3/13_Confidence_0.706_Length_850	FORWARD	54.07	42.86	TTTTCTCTCTGCAACTCTCTC	155	CCT	CCTCCTCCTCCTCCTCCT	18
Locus_11539_Transcript_3/13_Confidence_0.706_Length_850	REVERSE	55.09	42.86	AAGAGTCTGTTTTGGGAGAAC				
Locus_11539_Transcript_3/13_Confidence_0.706_Length_850	FORWARD	55.12	35	AAAATGCAGATCCAATCAAC	132	CCT	CCTCCTCCTCCTCCTCCTCCT	21
Locus_11539_Transcript_3/13_Confidence_0.706_Length_850	REVERSE	55.05	42.86	TTGCAGAGAGAAAAGAGTCTG				
Locus_11592_Transcript_3/4_Confidence_0.700_Length_651	FORWARD	59.09	50	AAGATGGACGGAGATGGAAG	170	GAG	GAGGAGGAGGAGGAGGAG	18
Locus_11592_Transcript_3/4_Confidence_0.700_Length_651	REVERSE	56.19	38.1	AAAATGGAGCTCATTCTGAAG				
Locus_11613_Transcript_21/21_Confidence_0.375_Length_1689	FORWARD	55.47	38.1	AGTTTAACCGAAACGAAAGAG	142	TCC	TCCTCCTCCTCCTCCTCC	18
Locus_11613_Transcript_21/21_Confidence_0.375_Length_1689	REVERSE	54.72	42.86	GACGGAAGAAGATTAGGTTTC				
Locus_11613_Transcript_21/21_Confidence_0.375_Length_1689	FORWARD	54.72	42.86	GTCGGAAACCTAATCTTCTTC	152	TCC	TCCTCCTCCTCCTCCTCC	18
Locus_11613_Transcript_21/21_Confidence_0.375_Length_1689	REVERSE	54.81	47.62	CTTCTCCATCTCTCTTTCCTC				
Locus_11774_Transcript_16/21_Confidence_0.636_Length_4369	FORWARD	54.57	38.1	TCAGTAGAAGTTGCACAAACA	136	AAC	AACAACAACAACAACAAC	18
Locus_11774_Transcript_16/21_Confidence_0.636_Length_4369	REVERSE	54.89	33.33	ATGTATGGGCTTTTGTTGTTA				
Locus_11778_Transcript_4/5_Confidence_0.667_Length_847	FORWARD	55.08	38.1	AACATTCTGTAACAGCAGCAT	146	TTG	TTGTTGTTGTTGTTGTTG	18
Locus_11778_Transcript_4/5_Confidence_0.667_Length_847	REVERSE	54.42	33.33	GCAATTTTATCACAGCTTTTC				

Locus_11819_Transcript_6/9_Confidence_0.545_Length_1400	FORWARD	55.5	42.86	AAGGAGAAGAGCAATTGAGAG	196	AGG	AGGAGGAGGAGGAGGAGG	18
Locus_11819_Transcript_6/9_Confidence_0.545_Length_1400	REVERSE	54.79	42.86	GCAACATTCTATGGAGTCAAC				
Locus_11840_Transcript_8/11_Confidence_0.647_Length_1299	FORWARD	58.03	45	AAGTTGGTGAGTGGGTTGAA	163	GAG	GAGGAGGAGGAGGAGGAGGAG	21
Locus_11840_Transcript_8/11_Confidence_0.647_Length_1299	REVERSE	55.54	40	TCTGCGGAAAACTATCAAAC				
Locus_11877_Transcript_7/9_Confidence_0.583_Length_1045	FORWARD	54.91	42.86	CGGCTAAAGAGTGAAGATACA	150	CTT	CTTCTTCTTCTTCTTCTT	18
Locus_11877_Transcript_7/9_Confidence_0.583_Length_1045	REVERSE	55.18	38.1	CTTGTTTTTCTCAATGCAGTC				
Locus_11877_Transcript_7/9_Confidence_0.583_Length_1045	FORWARD	55.36	33.33	TAAAAGCCTCAAGTGTTTCAA	158	AAG	AAGAAGAAGAAGAAGAAGAAG	21
Locus_11877_Transcript_7/9_Confidence_0.583_Length_1045	REVERSE	55.15	42.86	TCATACTCCTTGTCCTGCTTA				
Locus_12123_Transcript_6/7_Confidence_0.647_Length_621	FORWARD	54.28	28.57	AAAAGGAATGAATAACAAGCA	143	GAA	GAAGAAGAAGAAGAAGAAGAA	21
Locus_12123_Transcript_6/7_Confidence_0.647_Length_621	REVERSE	55.04	42.86	ACATGCTCTGGTCTTTCTACA				
Locus_12157_Transcript_2/3_Confidence_0.769_Length_339	FORWARD	54.91	28.57	TTTCAAATTAACCATTCCTCA	176	AGA	AGAAGAAGAAGAAGAAGAAGAAGA	24
Locus_12157_Transcript_2/3_Confidence_0.769_Length_339	REVERSE	53.76	42.86	AATAGATGTTCTCGACTCTCG				
Locus_12277_Transcript_9/12_Confidence_0.664_Length_1200	FORWARD	55.81	42.86	AAGAGGATGAGGAGAATGATG	157	CCG	CCGCCGCCGCCGCCGCCGCCG	21
Locus_12277_Transcript_9/12_Confidence_0.664_Length_1200	REVERSE	55.15	42.86	CTAATCCGACCCTAATCCTTA				
Locus_12287_Transcript_1/6_Confidence_0.636_Length_705	FORWARD	54.8	38.1	GCTTTCTCACTTTCAGAATCA	152	CTT	CTTCTTCTTCTTCTTCTT	18
Locus_12287_Transcript_1/6_Confidence_0.636_Length_705	REVERSE	54.98	45	GAAGCTCAATCAATGGAGAG				
Locus_12287_Transcript_1/6_Confidence_0.636_Length_705	FORWARD	54.58	38.1	CTTTGTTTCAGCATGATACCT	149	CTG	CTGCTGCTGCTGCTGCTG	18
Locus_12287_Transcript_1/6_Confidence_0.636_Length_705	REVERSE	55.24	38.1	GAAGAGAGGGATTTCTCAAAA				
Locus_12348_Transcript_27/41_Confidence_0.534_Length_1122	FORWARD	55.51	45	GAAAGGAAGATTGAGGGAAG	196	CGG	CGGCGGCGGCGGCGGCGGCGG	21
Locus_12348_Transcript_27/41_Confidence_0.534_Length_1122	REVERSE	56.06	42.86	ATCTTTGCCACTTCGATCTAC				
Locus_12392_Transcript_8/14_Confidence_0.645_Length_766	FORWARD	55.89	45	GTGGAATTTCAGTTGCTCAG	139	GAA	GAAGAAGAAGAAGAAGAAGAAGAAGAAG	30
Locus_12392_Transcript_8/14_Confidence_0.645_Length_766	REVERSE	55.17	42.86	AAATACTCATCCACCTCACCT				
Locus_12647_Transcript_5/8_Confidence_0.711_Length_1078	FORWARD	54.65	42.86	GTCTCAGATGCGGATTAAGTA	141	CGG	CGGCGGCGGCGGCGGCGG	18
Locus_12647_Transcript_5/8_Confidence_0.711_Length_1078	REVERSE	56.86	50	GAGACAGACGGTTCTCGAAT				
Locus_12647_Transcript_5/8_Confidence_0.711_Length_1078	FORWARD	54.65	42.86	GTCTCAGATGCGGATTAAGTA	109	CGG	CGGCGGCGGCGGCGGCGG	18
Locus_12647_Transcript_5/8_Confidence_0.711_Length_1078	REVERSE	54.98	45	CATAACTCCCTTTCCCTTCT				
Locus_12880_Transcript_3/7_Confidence_0.778_Length_1955	FORWARD	55.31	42.86	TTCCTCCTCTTCTTCTTCAAC	203	GGA	GGAGGAGGAGGAGGAGGAGGAGGA	24
Locus_12880_Transcript_3/7_Confidence_0.778_Length_1955	REVERSE	56.01	55	CACTCGTCCACTCTGCTAAC				
Locus_12919_Transcript_4/10_Confidence_0.710_Length_776	FORWARD	55.04	36.36	TCTTCTTCTCCATACAAACACA	122	CCT	CCTCCTCCTCCTCCTCCTCCT	21
Locus_12919_Transcript_4/10_Confidence_0.710_Length_776	REVERSE	55.34	47.62	GGAGAAGATGGAGAGAACATC				
Locus_12982_Transcript_11/19_Confidence_0.585_Length_1877	FORWARD	54.9	42.86	GCTATACGTTGAAAGAGACGA	163	AGA	AGAAGAAGAAGAAGAAGAAGAAGA	24
Locus_12982_Transcript_11/19_Confidence_0.585_Length_1877	REVERSE	55.44	38.1	TTACTTTGCTGTATTGGATGG				
Locus_12995_Transcript_5/5_Confidence_0.500_Length_443	FORWARD	56.41	40	ACCTTCAAATCCCAATTCAC	159	CTC	CTCCTCCTCCTCCTCCTCCTC	21
Locus_12995_Transcript_5/5_Confidence_0.500_Length_443	REVERSE	54.95	38.1	TAGGAGTGATTGGAAAACTCA				
Locus_13273_Transcript_8/10_Confidence_0.644_Length_758	FORWARD	54.99	33.33	ACCAATCTCATAAACAAGCAA	131	CCT	CCTCCTCCTCCTCCTCCT	18
Locus_13273_Transcript_8/10_Confidence_0.644_Length_758	REVERSE	54.51	52.38	AAGTAGGAGAGGAGAGTCGAG				
Locus_13395_Transcript_1/2_Confidence_0.750_Length_388	FORWARD	55.62	42.86	GTGGGTTAGGTGAACGATTAT	137	GGA	GGAGGAGGAGGAGGAGGA	18
Locus_13395_Transcript_1/2_Confidence_0.750_Length_388	REVERSE	54.78	50	GAGATCAATGGAGTCTCTCG				
Locus_13476_Transcript_6/8_Confidence_0.630_Length_974	FORWARD	54.71	42.86	ATCTGCTTTACATGAGAGTCG	150	GAA	GAAGAAGAAGAAGAAGAA	18
Locus_13476_Transcript_6/8_Confidence_0.630_Length_974	REVERSE	54.91	38.1	GCAACAATTTCATATGGAGAG				
Locus_13690_Transcript_4/6_Confidence_0.762_Length_427	FORWARD	54.13	38.1	TCTTGCTTTTCTTCTATGGAG	140	CTG	CTGCTGCTGCTGCTGCTGCTGCTGCTG	27

Locus_13690_Transcript_4/6_Confidence_0.762_Length_427	REVERSE	55.05	42.86	TCCATCACTTTCACATACCTC				
Locus_13809_Transcript_1/1_Confidence_0.000_Length_250	FORWARD	55.91	42.86	ATTTCCTTACGACATGAGAGG	151	GAA	GAAGAAGAAGAAGAAGAA	18
Locus_13809_Transcript_1/1_Confidence_0.000_Length_250	REVERSE	55.03	42.86	AGTGGCTGGACCTTATATTTC				
Locus_14274_Transcript_3/3_Confidence_0.956_Length_634	FORWARD	54.44	38.1	CGTAATTTAGGGTTTCCTTCT	149	TCT	TCTTCTTCTTCTTCTTCT	18
Locus_14274_Transcript_3/3_Confidence_0.956_Length_634	REVERSE	55.16	28.57	ATCAAACGCATCAAAACTAAA				
Locus_14304_Transcript_5/6_Confidence_0.882_Length_879	FORWARD	55.04	33.33	GATTTGCTTGAAACAGCTTTA	158	ATC	ATCATCATCATCATCATC	18
Locus_14304_Transcript_5/6_Confidence_0.882_Length_879	REVERSE	54.94	33.33	AGGTTGAAAGGAAGAAGAAAA				
Locus_14344_Transcript_1/2_Confidence_0.884_Length_352	FORWARD	55.2	42.86	TTACTACGCTCAACCCATAGA	207	ATT	ATTATTATTATTATTATT	18
Locus_14344_Transcript_1/2_Confidence_0.884_Length_352	REVERSE	54.96	42.86	GGACACATCTCCATCAGAATA				
Locus_14525_Transcript_1/1_Confidence_0.970_Length_331	FORWARD	57.1	50	AGTGTTCAAGAAGGCAGGAG	130	GGT	GGTGGTGGTGGTGGTGGTGGT	21
Locus_14525_Transcript_1/1_Confidence_0.970_Length_331	REVERSE	55.08	38.1	CCACTTTAACAAATCTCAACG				
Locus_14759_Transcript_1/2_Confidence_0.980_Length_409	FORWARD	55.17	35	AAGAAACCAGTTGCAGAAAA	150	AGA	AGAAGAAGAAGAAGAAGA	18
Locus_14759_Transcript_1/2_Confidence_0.980_Length_409	REVERSE	54.3	43.48	AGATGTAGATCTTGTACGTCTCC				
Locus_14803_Transcript_2/3_Confidence_0.929_Length_1021	FORWARD	55.16	28.57	ATCAAACGCATCAAAACTAAA	149	AGA	AGAAGAAGAAGAAGAAGA	18
Locus_14803_Transcript_2/3_Confidence_0.929_Length_1021	REVERSE	54.44	38.1	CGTAATTTAGGGTTTCCTTCT				
Locus_14803_Transcript_2/3_Confidence_0.929_Length_1021	FORWARD	54.77	33.33	ACGCATCAAAACTAAATCAAG	151	AGA	AGAAGAAGAAGAAGAAGAAGAAGA	24
Locus_14803_Transcript_2/3_Confidence_0.929_Length_1021	REVERSE	54.2	38.1	TCGTTTAACAGCTTCTTCTTC				
Locus_14952_Transcript_1/1_Confidence_1.000_Length_733	FORWARD	54.17	38.1	CCTTCTCATTCTCAAATATCG	151	CTC	CTCCTCCTCCTCCTCCTC	18
Locus_14952_Transcript_1/1_Confidence_1.000_Length_733	REVERSE	55.3	33.33	TTCCCTTTCTTTTGAATTAGG				
Locus_15000_Transcript_1/1_Confidence_1.000_Length_271	FORWARD	54.83	42.86	CAAAAGTCTGATCTGAACACC	167	TCT	TCTTCTTCTTCTTCTTCTTCTTCT	24
Locus_15000_Transcript_1/1_Confidence_1.000_Length_271	REVERSE	54.37	38.1	TCTAATATACACACGGTGCAA				
Locus_15001_Transcript_1/1_Confidence_1.000_Length_277	FORWARD	55.26	38.1	TTCAAAAGTCTGATGAACACC	169	TCT	TCTTCTTCTTCTTCTTCTTCTTCT	24
Locus_15001_Transcript_1/1_Confidence_1.000_Length_277	REVERSE	54.37	38.1	TCTAATATACACACGGTGCAA				
Locus_15336_Transcript_1/1_Confidence_1.000_Length_372	FORWARD	55.31	42.86	ATTTCTGGAAGCTATGGAGAG	127	AGA	AGAAGAAGAAGAAGAAGAAGAAGA	24
Locus_15336_Transcript_1/1_Confidence_1.000_Length_372	REVERSE	55.03	38.1	TTTAGGGTTTCTTCACAGACA				
Locus_15337_Transcript_1/1_Confidence_1.000_Length_525	FORWARD	55.16	28.57	AACGCATCAAAACTAAATCAA	150	AGA	AGAAGAAGAAGAAGAAGAAGAAGA	24
Locus_15337_Transcript_1/1_Confidence_1.000_Length_525	REVERSE	54.08	38.1	CATCGTTTAACAGCTTCTTCT				
Locus_15339_Transcript_1/1_Confidence_1.000_Length_781	FORWARD	55.16	28.57	ATCAAACGCATCAAAACTAAA	149	AGA	AGAAGAAGAAGAAGAAGA	18
Locus_15339_Transcript_1/1_Confidence_1.000_Length_781	REVERSE	54.44	38.1	CGTAATTTAGGGTTTCCTTCT				
Locus_15339_Transcript_1/1_Confidence_1.000_Length_781	FORWARD	54.77	33.33	ACGCATCAAAACTAAATCAAG	151	AGA	AGAAGAAGAAGAAGAAGAAGAAGA	24
Locus_15339_Transcript_1/1_Confidence_1.000_Length_781	REVERSE	54.2	38.1	TCGTTTAACAGCTTCTTCTTC				
Locus_15340_Transcript_1/1_Confidence_1.000_Length_329	FORWARD	55.72	47.62	ACTCTCCATCTCATCTTCACC	154	AAG	AAGAAGAAGAAGAAGAAGAAG	21
Locus_15340_Transcript_1/1_Confidence_1.000_Length_329	REVERSE	55.25	47.62	GGCGGAGATAGTGTTCTTACT				
Locus_15344_Transcript_1/1_Confidence_1.000_Length_482	FORWARD	54.94	36.36	AGCAAACAAGAAGAAGAAGAAG	148	AAG	AAGAAGAAGAAGAAGAAGAAGAAG	24
Locus_15344_Transcript_1/1_Confidence_1.000_Length_482	REVERSE	54.55	38.1	TGTTCACATACACGCTTTCTA				
Locus_15975_Transcript_1/1_Confidence_1.000_Length_541	FORWARD	56.19	34.78	TGTTCTGAGAGGTTTGTATTTTG	162	AAG	AAGAAGAAGAAGAAGAAG	18
Locus_15975_Transcript_1/1_Confidence_1.000_Length_541	REVERSE	59.06	55	GGATCTCACCGGATCTCACT				
Locus_15976_Transcript_1/1_Confidence_1.000_Length_542	FORWARD	56.19	34.78	TGTTCTGAGAGGTTTGTATTTTG	162	AAG	AAGAAGAAGAAGAAGAAG	18
Locus_15976_Transcript_1/1_Confidence_1.000_Length_542	REVERSE	59.06	55	GGATCTCACCGGATCTCACT				
Locus_15977_Transcript_1/1_Confidence_1.000_Length_441	FORWARD	56.19	34.78	TGTTCTGAGAGGTTTGTATTTTG	162	AAG	AAGAAGAAGAAGAAGAAG	18
Locus_15977_Transcript_1/1_Confidence_1.000_Length_441	REVERSE	59.06	55	GGATCTCACCGGATCTCACT				

Locus_15978_Transcript_1/1_Confidence_1.000_Length_460	FORWARD	56.19	34.78	TGTTCTGAGAGGTTTGTATTTTG	162	AAG	AAGAAGAAGAAGAAGAAG	18
Locus_15978_Transcript_1/1_Confidence_1.000_Length_460	REVERSE	59.06	55	GGATCTCACCGGATCTCACT				
Locus_16041_Transcript_1/1_Confidence_1.000_Length_562	FORWARD	54.93	42.86	ATCTTGTACGTCTCCGTTGTA	155	CTT	CTTCTTCTTCTTCTTCTT	18
Locus_16041_Transcript_1/1_Confidence_1.000_Length_562	REVERSE	55.17	40	GAAACCAGTTGCAGAAAAAG				
Locus_16042_Transcript_1/1_Confidence_1.000_Length_413	FORWARD	55.57	47.62	AGATCTTGTACGTCTCCGTCT	146	CTT	CTTCTTCTTCTTCTTCTT	18
Locus_16042_Transcript_1/1_Confidence_1.000_Length_413	REVERSE	55.17	35	AAAAGAAACCAGTTGCAGAA				
Locus_16514_Transcript_1/1_Confidence_1.000_Length_259	FORWARD	55.42	42.86	AGGAAGAGTTCGAAGTTGAAG	104	GAA	GAAGAAGAAGAAGAAGAAGAA	21
Locus_16514_Transcript_1/1_Confidence_1.000_Length_259	REVERSE	55.09	47.62	TTAAGCTCTCCTCACTCCTCT				
Locus_17142_Transcript_1/1_Confidence_1.000_Length_457	FORWARD	55.39	38.1	ACTCTTTTGGCTTCTATTTGG	150	CTC	CTCCTCCTCCTCCTCCTC	18
Locus_17142_Transcript_1/1_Confidence_1.000_Length_457	REVERSE	54.52	33.33	GAAGAAAAGAAGAAGAAGAAGAAG				
Locus_17450_Transcript_1/1_Confidence_1.000_Length_700	FORWARD	55.27	42.86	CTTAGTGGGATAAAGGCTTGT	157	GTT	GTTGTTGTTGTTGTTGTT	18
Locus_17450_Transcript_1/1_Confidence_1.000_Length_700	REVERSE	54.7	38.1	ATCACGAAGTATGCAAATAGC				
Locus_17489_Transcript_1/1_Confidence_1.000_Length_483	FORWARD	54.44	38.1	CGTAATTTAGGGTTTCCTTCT	149	TCT	TCTTCTTCTTCTTCTTCT	18
Locus_17489_Transcript_1/1_Confidence_1.000_Length_483	REVERSE	55.16	28.57	ATCAAACGCATCAAAACTAAA				
Locus_17492_Transcript_1/1_Confidence_1.000_Length_501	FORWARD	54.85	33.33	ATGATAAAGGCGAAGAAGATT	149	TCT	TCTTCTTCTTCTTCTTCT	18
Locus_17492_Transcript_1/1_Confidence_1.000_Length_501	REVERSE	55.15	38.1	GTTTCCATTTCTGGAAGCTAT				
Locus_17493_Transcript_1/1_Confidence_1.000_Length_579	FORWARD	54.85	33.33	ATGATAAAGGCGAAGAAGATT	149	TCT	TCTTCTTCTTCTTCTTCT	18
Locus_17493_Transcript_1/1_Confidence_1.000_Length_579	REVERSE	55.15	38.1	GTTTCCATTTCTGGAAGCTAT				
Locus_17797_Transcript_1/1_Confidence_1.000_Length_244	FORWARD	55	38.1	CGCTCTTCTTCTTTCTTCTTT	157	TTC	TTCTTCTTCTTCTTCTTCTTC	21
Locus_17797_Transcript_1/1_Confidence_1.000_Length_244	REVERSE	54.96	42.86	CAAGGGTTAACAATCTGTACG				
Locus_17810_Transcript_1/1_Confidence_1.000_Length_335	FORWARD	54.92	38.1	TCCTCTCTCTTTTTGTCCTTT	174	TCT	TCTTCTTCTTCTTCTTCTTCTTCT	24
Locus_17810_Transcript_1/1_Confidence_1.000_Length_335	REVERSE	55.37	40	TAAACTCTCGTTCGCTTTGT				
Locus_17811_Transcript_1/1_Confidence_1.000_Length_311	FORWARD	54.92	38.1	TCCTCTCTCTTTTTGTCCTTT	174	TCT	TCTTCTTCTTCTTCTTCTTCTTCT	24
Locus_17811_Transcript_1/1_Confidence_1.000_Length_311	REVERSE	55.37	40	TAAACTCTCGTTCGCTTTGT				
Locus_17812_Transcript_1/1_Confidence_1.000_Length_315	FORWARD	54.92	38.1	TCCTCTCTCTTTTTGTCCTTT	178	TCT	TCTTCTTCTTCTTCTTCTTCT	21
Locus_17812_Transcript_1/1_Confidence_1.000_Length_315	REVERSE	55.37	40	TAAACTCTCGTTCGCTTTGT				
Locus_18813_Transcript_1/1_Confidence_1.000_Length_519	FORWARD	55.46	47.62	GATCTTGTAAGTCTCGGTTCC	150	TCT	TCTTCTTCTTCTTCTTCT	18
Locus_18813_Transcript_1/1_Confidence_1.000_Length_519	REVERSE	56	45	GTGGAGGAGAAGGAAAAGAA				
Locus_18814_Transcript_1/1_Confidence_1.000_Length_521	FORWARD	55.46	47.62	GATCTTGTAAGTCTCGGTTCC	150	TCT	TCTTCTTCTTCTTCTTCT	18
Locus_18814_Transcript_1/1_Confidence_1.000_Length_521	REVERSE	56	45	GTGGAGGAGAAGGAAAAGAA				
Locus_18815_Transcript_1/1_Confidence_1.000_Length_342	FORWARD	54.92	42.86	TCAGGACCTTGAAGATGTAGA	166	TCT	TCTTCTTCTTCTTCTTCT	18
Locus_18815_Transcript_1/1_Confidence_1.000_Length_342	REVERSE	56.21	47.62	GAGGAGAAGGAAAAGAAGACC				
Locus_19055_Transcript_1/1_Confidence_1.000_Length_235	FORWARD	54.84	42.86	GAGAATGATGGAGAAGAGGAT	148	CCG	CCGCCGCCGCCGCCGCCGCCG	21
Locus_19055_Transcript_1/1_Confidence_1.000_Length_235	REVERSE	55.15	42.86	CCTTATCGACCCTAATCCTTA				
Locus_19213_Transcript_1/1_Confidence_1.000_Length_656	FORWARD	54.55	42.86	GAAGGAGAAGAAAGAAGGAGA	152	AGA	AGAAGAAGAAGAAGAAGA	18
Locus_19213_Transcript_1/1_Confidence_1.000_Length_656	REVERSE	55.47	38.1	CTTGATCAAACAGAACAGCAT				
Locus_19912_Transcript_1/1_Confidence_1.000_Length_831	FORWARD	55.09	33.33	TCACTTTTTGATTGACACACA	147	CTT	CTTCTTCTTCTTCTTCTT	18
Locus_19912_Transcript_1/1_Confidence_1.000_Length_831	REVERSE	55.13	33.33	AGAAAATTGAAGAATCCAAGG				
Locus_20453_Transcript_1/1_Confidence_1.000_Length_405	FORWARD	57.12	47.62	GTGGAGGAGAAGGAAAAGAAG	144	AAG	AAGAAGAAGAAGAAGAAG	18
Locus_20453_Transcript_1/1_Confidence_1.000_Length_405	REVERSE	58.52	50	CTTCTCAGCCTTGGGTTTCT				
Locus_21135_Transcript_1/1_Confidence_1.000_Length_325	FORWARD	55.49	50	AAGAAGAAGCAGAGGAGGAG	160	GAG	GAGGAGGAGGAGGAGGAG	18

Locus_21135_Transcript_1/1_Confidence_1.000_Length_325	REVERSE	56.44	45	ATAACCTCCTCCCAACAATG				
Locus_21136_Transcript_1/1_Confidence_1.000_Length_280	FORWARD	55.49	50	AAGAAGAAGCAGAGGAGGAG	103	GAG	GAGGAGGAGGAGGAGGAG	18
Locus_21136_Transcript_1/1_Confidence_1.000_Length_280	REVERSE	56.17	38.1	AACTCGACGACAAAAACTTTC				
Locus_21137_Transcript_1/1_Confidence_1.000_Length_223	FORWARD	55.49	50	AAGAAGAAGCAGAGGAGGAG	198	GAG	GAGGAGGAGGAGGAGGAG	18
Locus_21137_Transcript_1/1_Confidence_1.000_Length_223	REVERSE	54.55	38.1	CAAAACCAGAGATGGAAGTAA				
Locus_21353_Transcript_1/1_Confidence_1.000_Length_278	FORWARD	57.58	45	CCACTCCCCACACTTAAAAA	159	CCT	CCTCCTCCTCCTCCTCCTCCT	21
Locus_21353_Transcript_1/1_Confidence_1.000_Length_278	REVERSE	55.18	45	AGTGAGAATCGTCGAACATC				
Locus_21436_Transcript_1/1_Confidence_1.000_Length_483	FORWARD	54.08	52.38	GAGGAGGAGAGAGAGAGAAAG	132	AGA	AGAAGAAGAAGAAGAAGA	18
Locus_21436_Transcript_1/1_Confidence_1.000_Length_483	REVERSE	54.31	42.86	CTGTTCTTCCTGTTTCTGAAG				
Locus_21437_Transcript_1/1_Confidence_1.000_Length_538	FORWARD	54.08	52.38	GAGGAGGAGAGAGAGAGAAAG	132	AGA	AGAAGAAGAAGAAGAAGA	18
Locus_21437_Transcript_1/1_Confidence_1.000_Length_538	REVERSE	54.31	42.86	CTGTTCTTCCTGTTTCTGAAG				
Locus_21438_Transcript_1/1_Confidence_1.000_Length_479	FORWARD	54.38	47.62	AGAAAGAGGGAGAGAGAGATG	165	AGA	AGAAGAAGAAGAAGAAGA	18
Locus_21438_Transcript_1/1_Confidence_1.000_Length_479	REVERSE	54.74	38.1	TGTTCTTCCTGTTTTCTGAAG				
Locus_21439_Transcript_1/1_Confidence_1.000_Length_534	FORWARD	54.38	47.62	AGAAAGAGGGAGAGAGAGATG	165	AGA	AGAAGAAGAAGAAGAAGA	18
Locus_21439_Transcript_1/1_Confidence_1.000_Length_534	REVERSE	54.74	38.1	TGTTCTTCCTGTTTTCTGAAG				
Locus_21440_Transcript_1/1_Confidence_1.000_Length_558	FORWARD	54.08	52.38	GAGGAGGAGAGAGAGAGAAAG	132	AGA	AGAAGAAGAAGAAGAAGA	18
Locus_21440_Transcript_1/1_Confidence_1.000_Length_558	REVERSE	54.31	42.86	CTGTTCTTCCTGTTTCTGAAG				
Locus_21441_Transcript_1/1_Confidence_1.000_Length_562	FORWARD	54.08	52.38	GAGGAGGAGAGAGAGAGAAAG	132	AGA	AGAAGAAGAAGAAGAAGA	18
Locus_21441_Transcript_1/1_Confidence_1.000_Length_562	REVERSE	54.31	42.86	CTGTTCTTCCTGTTTCTGAAG				
Locus_21858_Transcript_1/1_Confidence_1.000_Length_316	FORWARD	55.72	47.62	ACTCTCCATCTCATCTTCACC	135	AAG	AAGAAGAAGAAGAAGAAGAAGAAG	24
Locus_21858_Transcript_1/1_Confidence_1.000_Length_316	REVERSE	55.25	47.62	GGCGGAGATAGTGTTCTTACT				
Locus_21894_Transcript_1/1_Confidence_1.000_Length_394	FORWARD	54.92	45.45	GAGAAATAGACGAGCTCATACC	185	GAC	GACGACGACGACGACGACGACGACGAC	27
Locus_21894_Transcript_1/1_Confidence_1.000_Length_394	REVERSE	54.92	42.86	ATCTCTCTTGCTTCTTGTCCT				

(C) Tetranucleotide repeat more than 5 times.

Seq ID	Orientation	Start	tm	GC%	Seq	Prod size	Motif	SSR	SSRLen
Locus_361_Transcript_8/10_Confidence_0.667_Length_2265	FORWARD	140	54.15	38.1	CTCAGATTCCATAATTTCAGC	149	TTAT	TTATTTATTTATTTATTTAT	20
Locus_361_Transcript_8/10_Confidence_0.667_Length_2265	REVERSE	288	54.25	42.86	TTTCAGTAAGCTTAGGACTCG				
Locus_361_Transcript_8/10_Confidence_0.667_Length_2265	FORWARD	161	55.25	33.33	CTCAAATAACAAATCCACCAA	165	TTAT	TTATTTATTTATTTATTTAT	20
Locus_361_Transcript_8/10_Confidence_0.667_Length_2265	REVERSE	325	54.97	42.86	CTACAAAATGTCATCCTCTGC				
Locus_898_Transcript_89/133_Confidence_0.333_Length_3222	FORWARD	175	55.12	38.1	GTGGTAACGCAAAAACATAAG	156	TTTA	TTTATTTATTTATTTATTTATTTA	24
Locus_898_Transcript_89/133_Confidence_0.333_Length_3222	REVERSE	330	54.64	33.33	TCAAGCTTCAAAGTAAATGCT				
Locus_1156_Transcript_76/96_Confidence_0.242_Length_1523	FORWARD	134	54.03	57.14	GAGAGGAGAGAGGAGAGAGAG	150	CGGT	CGGTCGGTCGGTCGGTCGGT	20
Locus_1156_Transcript_76/96_Confidence_0.242_Length_1523	REVERSE	283	55.01	33.33	CACCATCACTTTTATTTTTGC				
Locus_1268_Transcript_4/12_Confidence_0.656_Length_1288	FORWARD	22	55.08	33.33	TTGCGCCTTAATTATTGTTAG	141	CCTG	CCTGCCTGCCTGCCTGCCTGCCTGCCTGCCT	32
Locus_1268_Transcript_4/12_Confidence_0.656_Length_1288	REVERSE	162	55.94	47.62	GCAGGAGAATCGTTAGAGAAG				
Locus_1633_Transcript_29/35_Confidence_0.365_Length_2130	FORWARD	119	54.03	57.14	GGGAGAGAGAGAGAGAGAGAG	153	CGGT	CGGTCGGTCGGTCGGTCGGTCGGTCGGT	28
Locus_1633_Transcript_29/35_Confidence_0.365_Length_2130	REVERSE	271	53.88	33.33	CTGTCAACGATTTTCTCTTTT				
Locus_2054_Transcript_31/32_Confidence_0.609_Length_1880	FORWARD	218	54.67	42.86	TATAGGCTTCTGAACATCTGG	154	TGTT	TGTTTGTTTGTTTGTTTGTT	20
Locus_2054_Transcript_31/32_Confidence_0.609_Length_1880	REVERSE	371	55.17	33.33	GAAAACCTTGGCATTTCTATT				
Locus_2641_Transcript_39/55_Confidence_0.330_Length_3582	FORWARD	172	55.2	42.86	TCTCTGATACCAATCCCCTAT	144	TTAC	TTACTTACTTACTTACTTACTTACTTACTTA	52
Locus_2641_Transcript_39/55_Confidence_0.330_Length_3582	REVERSE	315	55.43	38.1	CAATTGCACGAGATTATCAGT				
Locus_3522_Transcript_36/40_Confidence_0.569_Length_2763	FORWARD	148	54.25	42.86	TGCAAGTTTAGTAGTCCTGGT	152	TTTA	TTTATTTATTTATTTATTTA	20
Locus_3522_Transcript_36/40_Confidence_0.569_Length_2763	REVERSE	299	54.87	47.62	CTCCGATTGTACAGAGTTGTC				
Locus_3567_Transcript_11/41_Confidence_0.437_Length_1143	FORWARD	48	54.52	42.86	CCAGTAGAACCTTGAATCTGA	154	CGGT	CGGTCGGTCGGTCGGTCGGTCGGT	24
Locus_3567_Transcript_11/41_Confidence_0.437_Length_1143	REVERSE	201	54.44	50	CATGGTCTCTCTCTCTCTCTCT				
Locus_3707_Transcript_29/31_Confidence_0.653_Length_4149	FORWARD	131	54.57	33.33	TGAGCTTTAAAAAGCAAAGAG	151	CGGT	CGGTCGGTCGGTCGGTCGGTCGGTCGGT	28
Locus_3707_Transcript_29/31_Confidence_0.653_Length_4149	REVERSE	281	54.99	27.27	TTGGGATAAGCTAATTTTTGTT				
Locus_4245_Transcript_17/22_Confidence_0.632_Length_2667	FORWARD	204	55.24	42.86	AAGCTGTCACTTCAAGACAGA	159	GGAG	GGAGGGAGGGAGGGAGGGAG	20
Locus_4245_Transcript_17/22_Confidence_0.632_Length_2667	REVERSE	362	55.42	47.62	GTCTCCTTTGACTCCTCATTC				
Locus_4600_Transcript_27/48_Confidence_0.520_Length_1792	FORWARD	140	55.18	33.33	TGTTTTCTTTGTGTGTTGTGA	149	TTTA	TTTATTTATTTATTTATTTA	20
Locus_4600_Transcript_27/48_Confidence_0.520_Length_1792	REVERSE	288	54.92	38.1	GTGCAAGATTCATGTCTTTTC				
Locus_4824_Transcript_11/26_Confidence_0.577_Length_1571	FORWARD	284	54.35	42.86	GATTCATGAACTAGAGCTTGC	134	TATG	TATGTATGTATGTATGTATGTATGTATG	28
Locus_4824_Transcript_11/26_Confidence_0.577_Length_1571	REVERSE	417	54.76	38.1	CCTACGCAAATTTACATCCTA				
Locus_4824_Transcript_11/26_Confidence_0.577_Length_1571	FORWARD	286	54.35	42.86	GATTCATGAACTAGAGCTTGC	134	TATG	TATGTATGTATGTATGTATGTATGTATG	28
Locus_4824_Transcript_11/26_Confidence_0.577_Length_1571	REVERSE	419	54.76	38.1	CCTACGCAAATTTACATCCTA				
Locus_4992_Transcript_40/52_Confidence_0.508_Length_2753	FORWARD	133	54.56	33.33	GGAAATGATGAAGATTGCTAA	151	AAAT	AAATAAATAAATAAATAAAT	20
Locus_4992_Transcript_40/52_Confidence_0.508_Length_2753	REVERSE	283	55.12	42.86	GAGGTTAATGAAGGAGGAGAA				
Locus_5532_Transcript_5/19_Confidence_0.393_Length_2148	FORWARD	97	55.3	38.1	ATTCTTGGAAAGTGGATGTCT	152	AGAT	AGATAGATAGATAGATAGATAGAT	24
Locus_5532_Transcript_5/19_Confidence_0.393_Length_2148	REVERSE	248	54.22	42.86	GTAGTGATGATGTGTGTGCAT				
Locus_5532_Transcript_5/19_Confidence_0.393_Length_2148	FORWARD	230	54.22	42.86	GCACACACATCATCACTACAT	156	AGAT	AGATAGATAGATAGATAGATAGAT	24
Locus_5532_Transcript_5/19_Confidence_0.393_Length_2148	REVERSE	385	55.04	42.86	ATCATATGCGGATGACTACAC				
Locus_5626_Transcript_20/89_Confidence_0.574_Length_2090	FORWARD	238	54.7	38.1	TGTCAATTAGTTTTCCTGCTC	146	TTTA	TTTATTTATTTATTTATTTA	20
Locus_5626_Transcript_20/89_Confidence_0.574_Length_2090	REVERSE	383	54.82	33.33	ATTCATATGACAATGCACACA				
Locus_5657_Transcript_52/60_Confidence_0.469_Length_1878	FORWARD	93	55.19	52.38	TCTCTCTCTCTCTCTGCCTCT	150	TTAC	TTACTTACTTACTTACTTAC	24
Locus_5657_Transcript_52/60_Confidence_0.469_Length_1878	REVERSE	242	55.09	27.27	AGAAAGCAAATTATTGAAGGAA				

Locus_5757_Transcript_17/25_Confidence_0.584_Length_810	FORWARD	109	55.18	23.81	TTTGAAATTACAACGCAAAAT	164	AGAT	AGATAGATAGATAGATAGATAGAT	24
Locus_5757_Transcript_17/25_Confidence_0.584_Length_810	REVERSE	272	55.04	42.86	GGAAATGGGGAGTAATGTATC				
Locus_7547_Transcript_34/42_Confidence_0.591_Length_2987	FORWARD	81	54.91	28.57	TTTGGTTTATTTCCTCAATCA	166	ATAA	ATAAATAAATAAATAAATAA	20
Locus_7547_Transcript_34/42_Confidence_0.591_Length_2987	REVERSE	246	55.25	38.1	TTTGAGAGTTTCTGCTGTTGT				
Locus_7695_Transcript_17/38_Confidence_0.556_Length_3232	FORWARD	276	54.49	42.86	GTAATGCTATCTCCTGGTGAA	146	ATGT	ATGTATGTATGTATGTATGTATGT	24
Locus_7695_Transcript_17/38_Confidence_0.556_Length_3232	REVERSE	421	54.24	28.57	AAATGGTTATTTTTCATGGTG				
Locus_9903_Transcript_11/17_Confidence_0.555_Length_2770	FORWARD	149	54.19	57.14	GAGCCTCTCTCTCTCTCTCTC	145	TTAC	TTACTTACTTACTTACTTACTTAC	24
Locus_9903_Transcript_11/17_Confidence_0.555_Length_2770	REVERSE	293	54.9	33.33	CCAAGTTCTGAAAGCAAATTA				
Locus_11085_Transcript_7/18_Confidence_0.575_Length_1602	FORWARD	140	54.62	42.86	GTCTGCGAAGTACTCAACAAT	144	GAAG	GAAGGAAGGAAGGAAGGAAG	20
Locus_11085_Transcript_7/18_Confidence_0.575_Length_1602	REVERSE	283	54.65	33.33	GCTGATTTGACTAATTTGGAA				
Locus_13166_Transcript_2/5_Confidence_0.882_Length_621	FORWARD	273	55.02	57.14	GACGGAGAGAGAGAGAGAGAG	147	CGGT	CGGTCGGTCGGTCGGTCGGTCGGT	24
Locus_13166_Transcript_2/5_Confidence_0.882_Length_621	REVERSE	419	53.88	33.33	CTGTCAACGATTTTCTCTTTT				
Locus_13609_Transcript_8/11_Confidence_0.610_Length_536	FORWARD	107	55.36	57.14	CGCTCTCTCTCTCTCTCTCTC	148	TTAC	TTACTTACTTACTTACTTACTTACTTAC	28
Locus_13609_Transcript_8/11_Confidence_0.610_Length_536	REVERSE	254	55	42.86	AGCTAGGGTTTAGAGTTTCCA				
Locus_15225_Transcript_1/1_Confidence_1.000_Length_437	FORWARD	184	55.09	42.86	TACCAATCCCCTATACCATCT	155	TTAC	TTACTTACTTACTTACTTACTTACTTACTTA	32
Locus_15225_Transcript_1/1_Confidence_1.000_Length_437	REVERSE	338	54.68	33.33	TTCATCATCTCCAACTTCATT				
Locus_15321_Transcript_1/1_Confidence_1.000_Length_524	FORWARD	213	56.43	45	GATGACTTTGGGACCTGATT	176	GTCG	GTCGGTCGGTCGGTCGGTCG	20
Locus_15321_Transcript_1/1_Confidence_1.000_Length_524	REVERSE	388	54.67	33.33	TGTCAACGATTTTCTCTTTTC				

(D) Pentanucleotide repeat more than 5 times.

Seq ID	Orientation	tm	GC%	Seq	Prod size	Motif	SSR	SSRLen
Locus_925_Transcript_4/14_Confidence_0.620_Length_3080	FORWARD	54.92	42.86	GATCAGAAGGAGGACAAAGAT	146	TGAGG	TGAGGTGAGGTGAGGTGAGGTGAGG	25
Locus_925_Transcript_4/14_Confidence_0.620_Length_3080	REVERSE	54.67	33.33	AATAAAACCCTGAACCCTAAA				
Locus_976_Transcript_5/19_Confidence_0.611_Length_729	FORWARD	54.78	35	GTCATGATGCAAAAATGCTA	166	AATGT	AATGTAATGTAATGTAATGTAATGT	25
Locus_976_Transcript_5/19_Confidence_0.611_Length_729	REVERSE	54.97	38.1	GCAATAAGCAAGCATAGGATA				
Locus_1787_Transcript_3/7_Confidence_0.639_Length_1199	FORWARD	54.83	38.1	AATCTTCTTCCCTTCTTCTGA	152	GATCA	GATCAGATCAGATCAGATCAGATCA	25
Locus_1787_Transcript_3/7_Confidence_0.639_Length_1199	REVERSE	55	38.1	TACTCTGAATCCGTGGTTAAA				
Locus_8869_Transcript_5/9_Confidence_0.675_Length_413	FORWARD	54.92	38.1	CTTAAGGGAAATGGAAGAAAG	136	TTATT	TTATTTTATTTTATTTTATTTTATTTTATT	30
Locus_8869_Transcript_5/9_Confidence_0.675_Length_413	REVERSE	55.27	33.33	ATACAAAAATGCATGGTCAAG				
Locus_17818_Transcript_1/1_Confidence_1.000_Length_321	FORWARD	55.07	31.82	CAAATCCCATACATATTTGACA	157	AAAAC	AAAACAAAACAAAACAAAACAAAAC	25
Locus_17818_Transcript_1/1_Confidence_1.000_Length_321	REVERSE	54.75	38.1	ATACTTGTTGTTGCTGATGGT				
Locus_17819_Transcript_1/1_Confidence_1.000_Length_453	FORWARD	55.07	31.82	CAAATCCCATACATATTTGACA	157	AAAAC	AAAACAAAACAAAACAAAACAAAAC	25
Locus_17819_Transcript_1/1_Confidence_1.000_Length_453	REVERSE	54.75	38.1	ATACTTGTTGTTGCTGATGGT				

(E) Hexanucleotide repeat more than 4 times.

Seq ID	Orientation	tm	GC%	Seq	Prod size	Motif	Motif Len	SSR	SSRLen
Locus_17_Transcript_42/50_Confidence_0.593_Length_5741	FORWARD	55.24	42.86	TGTGCATAATAGGGGTGATAG	153	TTTATT	6	TTTATTTTTATTTTTATTTTTATTTTTATT	30
Locus_17_Transcript_42/50_Confidence_0.593_Length_5741	REVERSE	54.91	28.57	TCTCAAATCCACCAAAAATTA					
Locus_17_Transcript_42/50_Confidence_0.593_Length_5741	FORWARD	54.91	28.57	TAATTTTTGGTGGATTTGAGA	154	TTTATT	6	TTTATTTTTATTTTTATTTTTATT	24
Locus_17_Transcript_42/50_Confidence_0.593_Length_5741	REVERSE	54.28	33.33	CAGCAGTAATAAACATGCAAA					
Locus_164_Transcript_118/136_Confidence_0.289_Length_1757	FORWARD	55.12	52.38	CCTAGACCTCCAACTCTGTCT	148	TCTCTG	6	TCTCTGTCTCTGTCTCTGTCTCTG	24
Locus_164_Transcript_118/136_Confidence_0.289_Length_1757	REVERSE	54.65	33.33	TGCATTTCTTATTGTTTCTCC					
Locus_337_Transcript_20/86_Confidence_0.573_Length_3129	FORWARD	56.09	42.86	TTTGACGACTACTGCAACTGT	142	CACCAA	6	CACCAACACCAACACCAACACCAA	24
Locus_337_Transcript_20/86_Confidence_0.573_Length_3129	REVERSE	54.55	42.86	GAACTGGAATTGACATCAGTC					
Locus_391_Transcript_19/24_Confidence_0.710_Length_4275	FORWARD	54.18	42.86	ATAACCTCTACCATGTGCATC	151	CTCGAC	6	CTCGACCTCGACCTCGACCTCGAC	24
Locus_391_Transcript_19/24_Confidence_0.710_Length_4275	REVERSE	55.4	50	GTCGTATGGTGGAAACAGAC					
Locus_391_Transcript_19/24_Confidence_0.710_Length_4275	FORWARD	55.4	50	GTCTGTTTCCACCATACGAC	174	CTCGAC	6	CTCGACCTCGACCTCGACCTCGACCTCGAC	30
Locus_391_Transcript_19/24_Confidence_0.710_Length_4275	REVERSE	55.11	38.1	TTGTTTCGAGATAGGAGTCAA					
Locus_505_Transcript_23/47_Confidence_0.573_Length_3437	FORWARD	55.52	42.86	CTGAAGATCTCCCATTCTTTC	152	CGCCAC	6	CGCCACCGCCACCGCCACCGCCACCGCCA	30
Locus_505_Transcript_23/47_Confidence_0.573_Length_3437	REVERSE	55.19	50	TCAGAGTCCGGTGAAGATAC					
Locus_921_Transcript_42/65_Confidence_0.583_Length_4364	FORWARD	55.4	42.86	CGAAGTTGATGGAGATACAGA	152	GAGGAT	6	GAGGATGAGGATGAGGATGAGGAT	24
Locus_921_Transcript_42/65_Confidence_0.583_Length_4364	REVERSE	54.64	38.1	ATGTGGTGAAGAAACTCCATA					
Locus_1256_Transcript_17/51_Confidence_0.535_Length_2636	FORWARD	55.86	47.62	CTCTAAAAGGGCAGACTGTTC	141	TTCCCT	6	TTCCCTTTCCCTTTCCCTTTCCCT	24
Locus_1256_Transcript_17/51_Confidence_0.535_Length_2636	REVERSE	54.8	47.62	CTCTTCTGAGAGGCTCTTCTT					
Locus_1462_Transcript_14/19_Confidence_0.587_Length_1312	FORWARD	54.98	42.86	CCAAAGAGAGTACATGAATCG	153	CCTCTC	6	CCTCTCCCTCTCCCTCTCCCTCTCCCTCTC	30
Locus_1462_Transcript_14/19_Confidence_0.587_Length_1312	REVERSE	54.64	38.1	TTACAAAGAGGTTTCAGTTGG					
Locus_1949_Transcript_6/11_Confidence_0.716_Length_2361	FORWARD	55.02	38.1	GACAGATTTTAGCTTGCTTGA	169	CCTCTC	6	CCTCTCCCTCTCCCTCTCCCTCTC	24
Locus_1949_Transcript_6/11_Confidence_0.716_Length_2361	REVERSE	54.04	38.1	GCAAATTCGGAAGAAGTAGTA					
Locus_1990_Transcript_48/77_Confidence_0.570_Length_4809	FORWARD	54.71	28.57	AAAATTATCGCCATCCTTAAT	149	GGAAAA	6	GGAAAAGGAAAAGGAAAAGGAAAA	24
Locus_1990_Transcript_48/77_Confidence_0.570_Length_4809	REVERSE	54.68	38.1	AAGAGGTCGGAGTGTTTTAAT					
Locus_2143_Transcript_8/14_Confidence_0.767_Length_793	FORWARD	54.58	52.38	CTAACCCTCTCCCTCATACTC	146	AAACCC	6	AAACCCAAACCCAAACCCAAACCCAAACC	30
Locus_2143_Transcript_8/14_Confidence_0.767_Length_793	REVERSE	56.33	50	GGGCTGATAGGGTTTGTAAG					
Locus_2623_Transcript_2/14_Confidence_0.684_Length_3447	FORWARD	55.51	42.86	ATCAGGATATGTTGGGAGAAC	171	GTGAAG	6	GTGAAGGTGAAGGTGAAGGTGAAGGTGAA	30
Locus_2623_Transcript_2/14_Confidence_0.684_Length_3447	REVERSE	55.13	31.82	AAACAACTGTTCCAAATCTTTC					
Locus_2639_Transcript_56/106_Confidence_0.388_Length_4129	FORWARD	55.15	47.62	GTATAAGGAGAGGGTTCGAGA	153	AGGGAG	6	AGGGAGAGGGAGAGGGAGAGGGAGAGGG	30
Locus_2639_Transcript_56/106_Confidence_0.388_Length_4129	REVERSE	55.03	38.1	TACTTGTCCCTTTCACTTTCA					
Locus_2642_Transcript_124/133_Confidence_0.268_Length_6476	FORWARD	54.47	42.86	TTCTCTCAGCTTCTCTATCCA	181	TCTCCC	6	TCTCCCTCTCCCTCTCCCTCTCCC	24
Locus_2642_Transcript_124/133_Confidence_0.268_Length_6476	REVERSE	55.34	38.1	ATGGATTCTGATAATGCCTCT					
Locus_2671_Transcript_85/89_Confidence_0.352_Length_3069	FORWARD	54.74	38.1	CGAAGCTCTCATAATTTGTGT	145	ACGAAG	6	ACGAAGACGAAGACGAAGACGAAG	24
Locus_2671_Transcript_85/89_Confidence_0.352_Length_3069	REVERSE	54.83	38.1	AGATCAAGGCGAGATCTATTT					
Locus_2775_Transcript_11/13_Confidence_0.667_Length_3349	FORWARD	55.26	38.1	ATCTCGAATGGAGTTTATGGT	148	ATGGAT	6	ATGGATATGGATATGGATATGGAT	24
Locus_2775_Transcript_11/13_Confidence_0.667_Length_3349	REVERSE	53.54	38.1	ACTGGTAATGTTGAGATCCAT					
Locus_2846_Transcript_18/25_Confidence_0.629_Length_1641	FORWARD	54.4	38.1	AACAAGTTGAAGCATATCCAC	136	AACCCT	6	AACCCTAACCCTAACCCTAACCCT	24
Locus_2846_Transcript_18/25_Confidence_0.629_Length_1641	REVERSE	55.25	52.38	CTCGTCTGGTAGTATGCAGAG					
Locus_2895_Transcript_9/19_Confidence_0.656_Length_1416	FORWARD	56.63	50	AGAGGATGATGAGGTCCAAG	147	GAGGAT	6	GAGGATGAGGATGAGGATGAGGAT	24
Locus_2895_Transcript_9/19_Confidence_0.656_Length_1416	REVERSE	54.64	38.1	AGGTGAATTGACATGTAAGGA					
Locus_2920_Transcript_36/43_Confidence_0.586_Length_2898	FORWARD	56.33	50	GGCTGATAGGGTTTGTAAGG	184	TGGGTT	6	TGGGTTTGGGTTTGGGTTTGGGTTTGGGTT	36

Locus_2920_Transcript_36/43_Confidence_0.586_Length_2898	REVERSE	54.58	52.38	CTAACCCTCTCCCTCATACTC					
Locus_3052_Transcript_19/32_Confidence_0.614_Length_3892	FORWARD	55.2	33.33	ACTACCAAAACCCAATCAAAT	152	CAACTT	6	CAACTTCAACTTCAACTTCAACTT	24
Locus_3052_Transcript_19/32_Confidence_0.614_Length_3892	REVERSE	54.33	33.33	TGCTATCATCTGAATGTGAAA					
Locus_3171_Transcript_9/19_Confidence_0.657_Length_701	FORWARD	55.04	42.86	CGTAAACCTTGATCTATGACG	176	CTCCCT	6	CTCCCTCTCCCTCTCCCTCTCCCT	24
Locus_3171_Transcript_9/19_Confidence_0.657_Length_701	REVERSE	54.72	42.86	GGGTCAAATATGGACTAAGGT					
Locus_3213_Transcript_21/41_Confidence_0.368_Length_3819	FORWARD	54.96	42.86	CACAGATCATTATAGGCCAAG	156	GGAGAG	6	GGAGAGGGAGAGGGAGAGGGAGAGGGAG	30
Locus_3213_Transcript_21/41_Confidence_0.368_Length_3819	REVERSE	55.05	40	ATCATCATGGTCTGGTTCAT					
Locus_3449_Transcript_24/52_Confidence_0.614_Length_9259	FORWARD	55.16	42.86	TTCATCAGGAGAATCTGTCAC	150	CTCAAC	6	CTCAACCTCAACCTCAACCTCAAC	24
Locus_3449_Transcript_24/52_Confidence_0.614_Length_9259	REVERSE	55.28	47.62	GTGAAGCAGTAGTTGCAGAAC					
Locus_4147_Transcript_11/25_Confidence_0.456_Length_2080	FORWARD	55.38	38.1	AACTCACCTTCCTCAATGTTT	154	GGAGAA	6	GGAGAAGGAGAAGGAGAAGGAGAA	24
Locus_4147_Transcript_11/25_Confidence_0.456_Length_2080	REVERSE	55.22	40	ATAATTTCCGCTTCTCCTTC					
Locus_4451_Transcript_19/29_Confidence_0.662_Length_5917	FORWARD	55.37	42.86	AGGAAGATGAGGTCAACAACT	178	AAGTTT	6	AAGTTTAAGTTTAAGTTTAAGTTTAAGTTT	30
Locus_4451_Transcript_19/29_Confidence_0.662_Length_5917	REVERSE	54.88	38.1	CAGCCTAAACAGAAAGATGAA					
Locus_4451_Transcript_19/29_Confidence_0.662_Length_5917	FORWARD	55.37	42.86	AGGAAGATGAGGTCAACAACT	174	AAGTTT	6	AAGTTTAAGTTTAAGTTTAAGTTTAAGTTT	30
Locus_4451_Transcript_19/29_Confidence_0.662_Length_5917	REVERSE	55.06	38.1	TCCACATGGAGACATTTTTAC					
Locus_4473_Transcript_17/24_Confidence_0.561_Length_4523	FORWARD	56.43	45	AGCAGCTTTCCAAACCTTAG	138	TCTCCT	6	TCTCCTTCTCCTTCTCCTTCTCCT	24
Locus_4473_Transcript_17/24_Confidence_0.561_Length_4523	REVERSE	55.5	42.86	AAGATCCAGCAGAAGAAGAAG					
Locus_4627_Transcript_24/28_Confidence_0.656_Length_6324	FORWARD	55.84	47.62	CCCTAACCCTAATCCTATTCC	181	CATCGC	6	CATCGCCATCGCCATCGCCATCGC	24
Locus_4627_Transcript_24/28_Confidence_0.656_Length_6324	REVERSE	55.24	45	ATAGTGAAGGAAGGGTCGAT					
Locus_4864_Transcript_5/12_Confidence_0.655_Length_1238	FORWARD	55.07	42.86	GATCAGATGGTAGTGCATGTT	147	TAAAAA	6	TAAAAATAAAAATAAAAATAAAAA	24
Locus_4864_Transcript_5/12_Confidence_0.655_Length_1238	REVERSE	54.67	33.33	TGATTCGTTTTCAGACTTTTC					
Locus_5631_Transcript_8/18_Confidence_0.588_Length_2004	FORWARD	55.01	38.1	CCACCCATTCTATTTTCTCTT	175	TCCCCT	6	TCCCCTTCCCCTTCCCCTTCCCCT	24
Locus_5631_Transcript_8/18_Confidence_0.588_Length_2004	REVERSE	54.31	38.1	GCCTGATTTGTCATTGATACT					
Locus_5631_Transcript_8/18_Confidence_0.588_Length_2004	FORWARD	55.4	38.1	TTCCCCTTTCAGAGTATCAAT	153	TCCCCT	6	TCCCCTTCCCCTTCCCCTTCCCCT	24
Locus_5631_Transcript_8/18_Confidence_0.588_Length_2004	REVERSE	55.34	38.1	GGAAATGAGAGAGCTTTTGTT					
Locus_5631_Transcript_8/18_Confidence_0.588_Length_2004	FORWARD	55.47	47.62	CCTTCCATCACCTTCTCTAAC	148	CCAATT	6	CCAATTCCAATTCCAATTCCAATT	24
Locus_5631_Transcript_8/18_Confidence_0.588_Length_2004	REVERSE	56.08	45	AGGAGATTGGTTGTGGAGTT					
Locus_5900_Transcript_29/35_Confidence_0.596_Length_1811	FORWARD	54.99	42.86	CTCTGATAGGAAAATCCTGGT	150	CTCTCC	6	CTCTCCCTCTCCCTCTCCCTCTCC	24
Locus_5900_Transcript_29/35_Confidence_0.596_Length_1811	REVERSE	54.33	47.62	GGTAGTGCAGAGAACAGAAAG					
Locus_5967_Transcript_22/43_Confidence_0.508_Length_1364	FORWARD	55.03	42.86	GCCCTAAACCTCAATGTCTAT	163	TATGTC	6	TATGTCTATGTCTATGTCTATGTC	24
Locus_5967_Transcript_22/43_Confidence_0.508_Length_1364	REVERSE	55.08	50	CTCCTAATCGCGACTGTATC					
Locus_6009_Transcript_5/12_Confidence_0.655_Length_1634	FORWARD	55.04	47.62	GACTTCCTGCTCTTTCTCTTC	159	GATCAC	6	GATCACGATCACGATCACGATCACGATCA	30
Locus_6009_Transcript_5/12_Confidence_0.655_Length_1634	REVERSE	56.18	45	AGCTTCCGACTTTGAATCTC					
Locus_6071_Transcript_4/6_Confidence_0.690_Length_899	FORWARD	55.63	38.1	AGATTTGGGTTCAATCATCTC	164	ACCAAA	6	ACCAAAACCAAAACCAAAACCAAA	24
Locus_6071_Transcript_4/6_Confidence_0.690_Length_899	REVERSE	55.18	47.62	TGGTGGAGTTGGTAGTGATAG					
Locus_6071_Transcript_4/6_Confidence_0.690_Length_899	FORWARD	55.18	47.62	CTATCACTACCAACTCCACCA	179	ACCGCC	6	ACCGCCACCGCCACCGCCACCGCC	24
Locus_6071_Transcript_4/6_Confidence_0.690_Length_899	REVERSE	57.22	45	TGGGATTATAACTGGGGATG					
Locus_6725_Transcript_18/34_Confidence_0.601_Length_2029	FORWARD	55.31	33.33	AAATCAGGTGGTTCAAATTCT	148	TGTGTT	6	TGTGTTTGTGTTTGTGTTTGTGTT	24
Locus_6725_Transcript_18/34_Confidence_0.601_Length_2029	REVERSE	54.96	28.57	AAACAACTCAAAAACCAACAA					
Locus_7607_Transcript_9/11_Confidence_0.619_Length_1489	FORWARD	54.83	38.1	CCCATACAATCTCCCTTTAAT	151	CTCACC	6	CTCACCCTCACCCTCACCCTCACC	24
Locus_7607_Transcript_9/11_Confidence_0.619_Length_1489	REVERSE	54.9	38.1	CATTTAAGCTCAACTGCTCAT					
Locus_7616_Transcript_14/41_Confidence_0.520_Length_1640	FORWARD	54.94	38.1	ATTTCTCTGCTGACCTTCTTT	139	CTTCCT	6	CTTCCTCTTCCTCTTCCTCTTCCT	24
Locus_7616_Transcript_14/41_Confidence_0.520_Length_1640	REVERSE	54.97	47.62	ACAAGATCAGAGGAGGATAGG					
Locus_7907_Transcript_33/39_Confidence_0.489_Length_2945	FORWARD	54.82	42.86	ACCCATCAAAGATCTACCTTC	151	CACCTC	6	CACCTCCACCTCCACCTCCACCTCCACCTC	36

Locus_7907_Transcript_33/39_Confidence_0.489_Length_2945	REVERSE	55.19	50	GAGCTTTGTGGTAGTTCTCG					
Locus_8509_Transcript_4/5_Confidence_0.684_Length_1119	FORWARD	55.25	47.62	GTATTCTGCTCGGTACCTTCT	154	TAGGGT	6	TAGGGTTAGGGTTAGGGTTAGGGT	24
Locus_8509_Transcript_4/5_Confidence_0.684_Length_1119	REVERSE	54.98	38.1	GTTACGAAACCATAACCCTTT					
Locus_8615_Transcript_4/6_Confidence_0.706_Length_962	FORWARD	54.59	38.1	CTTCATTCTTCTGTTGTTCGT	148	AGAGGG	6	AGAGGGAGAGGGAGAGGGAGAGGG	24
Locus_8615_Transcript_4/6_Confidence_0.706_Length_962	REVERSE	55.38	47.62	AGCTGGAATTAGAGTGAGAGG					
Locus_8876_Transcript_24/35_Confidence_0.581_Length_1474	FORWARD	55.9	42.86	AGCTCAACCTGTATCCAATTC	125	CCCTAA	6	CCCTAACCCTAACCCTAACCCTAA	24
Locus_8876_Transcript_24/35_Confidence_0.581_Length_1474	REVERSE	54.02	45	GAAGGTGTTTTGAGAGGAAG					
Locus_9051_Transcript_2/5_Confidence_0.727_Length_698	FORWARD	55.69	30	TTGAGGATTTCATGGAAAAA	151	GGTTAG	6	GGTTAGGGTTAGGGTTAGGGTTAG	24
Locus_9051_Transcript_2/5_Confidence_0.727_Length_698	REVERSE	55.81	42.86	ACGAACTCTATCTGCTCGATT					
Locus_9432_Transcript_1/8_Confidence_0.321_Length_1244	FORWARD	54.99	47.62	CACAGATTGAAGAGACTGAGG	152	AACCCT	6	AACCCTAACCCTAACCCTAACCCT	24
Locus_9432_Transcript_1/8_Confidence_0.321_Length_1244	REVERSE	56.01	47.62	CGAGCTAGTTCAAGAAGAACC					
Locus_9432_Transcript_1/8_Confidence_0.321_Length_1244	FORWARD	54.99	47.62	CACAGATTGAAGAGACTGAGG	149	AACCCT	6	AACCCTAACCCTAACCCTAACCCT	24
Locus_9432_Transcript_1/8_Confidence_0.321_Length_1244	REVERSE	55.18	42.86	AAGAGGGAATGAAACTCTCTG					
Locus_9453_Transcript_15/34_Confidence_0.613_Length_2275	FORWARD	55.11	42.86	ACATCAGTGGAGAACATGAAG	147	GAGGTG	6	GAGGTGGAGGTGGAGGTGGAGGTG	24
Locus_9453_Transcript_15/34_Confidence_0.613_Length_2275	REVERSE	54.58	38.1	AAAAAGACATGCCATCTACAG					
Locus_9544_Transcript_11/29_Confidence_0.597_Length_2244	FORWARD	55.01	42.86	ACTTCCTCAACCATCTTCTTC	149	CTCTTC	6	CTCTTCCTCTTCCTCTTCCTCTTCCTCTTC	30
Locus_9544_Transcript_11/29_Confidence_0.597_Length_2244	REVERSE	54.46	33.33	AAGAATTGGTGAGTTTGTTTG					
Locus_9631_Transcript_11/14_Confidence_0.588_Length_2037	FORWARD	55.44	42.86	TGTGACCAGGCTTATATTCTG	150	TGCTGA	6	TGCTGATGCTGATGCTGATGCTGA	24
Locus_9631_Transcript_11/14_Confidence_0.588_Length_2037	REVERSE	55.22	38.1	AATCAAGTCCTCCTCATCAAT					
Locus_10042_Transcript_20/23_Confidence_0.591_Length_2368	FORWARD	55.11	38.1	GCAAACTCAGCTGTTTCTTTA	148	TCCACC	6	TCCACCTCCACCTCCACCTCCACC	24
Locus_10042_Transcript_20/23_Confidence_0.591_Length_2368	REVERSE	55.61	47.62	CTGACATTAGTGGATGAGGTG					
Locus_10275_Transcript_13/18_Confidence_0.569_Length_669	FORWARD	55.22	45	CCAAAGTTGCTTCTAGGTTG	136	CCGTCG	6	CCGTCGCCGTCGCCGTCGCCGTCG	24
Locus_10275_Transcript_13/18_Confidence_0.569_Length_669	REVERSE	55.08	52.38	GGTCCATAGGAGCTGTACTCT					
Locus_10299_Transcript_5/21_Confidence_0.619_Length_1332	FORWARD	55.05	42.86	TCAACTATGATCCAAGTGACC	155	CCCTAA	6	CCCTAACCCTAACCCTAACCCTAA	24
Locus_10299_Transcript_5/21_Confidence_0.619_Length_1332	REVERSE	54.98	38.1	TCCACCACTAAAGCCATAATA					
Locus_11178_Transcript_8/12_Confidence_0.603_Length_1430	FORWARD	54.52	28.57	TGTCATGTTTGATTTCATTTG	149	GGAAGG	6	GGAAGGGGAAGGGGAAGGGGAAGG	24
Locus_11178_Transcript_8/12_Confidence_0.603_Length_1430	REVERSE	55.18	42.86	CAAGATCCTCACTTTCTTCCT					
Locus_11179_Transcript_37/43_Confidence_0.556_Length_1834	FORWARD	55.46	52.38	CTATAGCTGCGACTACCACTG	138	CGGCTC	6	CGGCTCCGGCTCCGGCTCCGGCTC	24
Locus_11179_Transcript_37/43_Confidence_0.556_Length_1834	REVERSE	55.36	33.33	ATTTTCACGGGCTTTTATTAG					
Locus_11742_Transcript_4/9_Confidence_0.706_Length_1372	FORWARD	55.05	38.1	TACAAGCTTGAGTTGGAACAT	146	TGAGGA	6	TGAGGATGAGGATGAGGATGAGGA	24
Locus_11742_Transcript_4/9_Confidence_0.706_Length_1372	REVERSE	55.36	38.1	TAGTTAACGTTGATTGGTTGG					
Locus_11880_Transcript_11/15_Confidence_0.656_Length_2661	FORWARD	56.43	38.1	ATCCAATCATCATCGAATCTC	145	TCCTTC	6	TCCTTCTCCTTCTCCTTCTCCTTC	24
Locus_11880_Transcript_11/15_Confidence_0.656_Length_2661	REVERSE	55.6	42.86	CGGGATAGAAAGGAAATAGTG					
Locus_11884_Transcript_5/7_Confidence_0.647_Length_928	FORWARD	57.27	45	ACTCGTTCTATTTGCGCTTC	97	CGCTTT	6	CGCTTTCGCTTTCGCTTTCGCTTT	24
Locus_11884_Transcript_5/7_Confidence_0.647_Length_928	REVERSE	54.88	47.62	CACCTTCATCTCAACCAGTAG					
Locus_12099_Transcript_7/12_Confidence_0.595_Length_1017	FORWARD	55.11	38.1	TTAACTGACGAGGATTTCTCA	153	AGCCCG	6	AGCCCGAGCCCGAGCCCGAGCCCG	24
Locus_12099_Transcript_7/12_Confidence_0.595_Length_1017	REVERSE	55.94	47.62	CAGATGCATCCTCACTAACTG					
Locus_12099_Transcript_7/12_Confidence_0.595_Length_1017	FORWARD	55.11	38.1	TTAACTGACGAGGATTTCTCA	153	AGCCCG	6	AGCCCGAGCCCGAGCCCGAGCCCG	24
Locus_12099_Transcript_7/12_Confidence_0.595_Length_1017	REVERSE	55.94	47.62	CAGATGCATCCTCACTAACTG					
Locus_14565_Transcript_4/5_Confidence_0.915_Length_901	FORWARD	56.33	50	GGCTGATAGGGTTTGTAAGG	155	TGGGTT	6	TGGGTTTGGGTTTGGGTTTGGGTTTGGGTT	36
Locus_14565_Transcript_4/5_Confidence_0.915_Length_901	REVERSE	54.58	52.38	CTAACCCTCTCCCTCATACTC					
Locus_14701_Transcript_1/2_Confidence_0.984_Length_616	FORWARD	54.58	52.38	CTAACCCTCTCCCTCATACTC	178	AAACCC	6	AAACCCAAACCCAAACCCAAACCCAAAC[illegible]	30
Locus_14701_Transcript_1/2_Confidence_0.984_Length_616	REVERSE	56.33	50	GGCTGATAGGGTTTGTAAGG					
Locus_15532_Transcript_1/1_Confidence_1.000_Length_1056	FORWARD	54.1	42.86	GAGACAATGGTGATTCTCACT	120	TGCAAA	6	TGCAAATGCAAATGCAAATGCAAA	24

Locus_15532_Transcript_1/1_Confidence_1.000_Length_1056	REVERSE	55.32	28.57	TTTTGTCTGCTTTTGCATTAT					
Locus_15533_Transcript_1/1_Confidence_1.000_Length_1056	FORWARD	54.1	42.86	GAGACAATGGTGATTCTCACT	120	TGCAAA	6	TGCAAATGCAAATGCAAATGCAAA	24
Locus_15533_Transcript_1/1_Confidence_1.000_Length_1056	REVERSE	55.32	28.57	TTTTGTCTGCTTTTGCATTAT					
Locus_15535_Transcript_1/1_Confidence_1.000_Length_354	FORWARD	54.1	42.86	GAGACAATGGTGATTCTCACT	120	TGCAAA	6	TGCAAATGCAAATGCAAATGCAAA	24
Locus_15535_Transcript_1/1_Confidence_1.000_Length_354	REVERSE	55.32	28.57	TTTTGTCTGCTTTTGCATTAT					
Locus_15536_Transcript_1/1_Confidence_1.000_Length_354	FORWARD	54.1	42.86	GAGACAATGGTGATTCTCACT	120	TGCAAA	6	TGCAAATGCAAATGCAAATGCAAA	24
Locus_15536_Transcript_1/1_Confidence_1.000_Length_354	REVERSE	55.32	28.57	TTTTGTCTGCTTTTGCATTAT					
Locus_15540_Transcript_1/1_Confidence_1.000_Length_401	FORWARD	54.1	42.86	GAGACAATGGTGATTCTCACT	120	TGCAAA	6	TGCAAATGCAAATGCAAATGCAAA	24
Locus_15540_Transcript_1/1_Confidence_1.000_Length_401	REVERSE	55.32	28.57	TTTTGTCTGCTTTTGCATTAT					
Locus_15541_Transcript_1/1_Confidence_1.000_Length_401	FORWARD	54.1	42.86	GAGACAATGGTGATTCTCACT	120	TGCAAA	6	TGCAAATGCAAATGCAAATGCAAA	24
Locus_15541_Transcript_1/1_Confidence_1.000_Length_401	REVERSE	55.32	28.57	TTTTGTCTGCTTTTGCATTAT					
Locus_15767_Transcript_1/1_Confidence_1.000_Length_1603	FORWARD	56.1	38.1	GCTTCAACAATCCTTTCAAGT	163	CACCAA	6	CACCAACACCAACACCAACACCAA	24
Locus_15767_Transcript_1/1_Confidence_1.000_Length_1603	REVERSE	54.55	42.86	GAACTGGAATTGACATCAGTC					
Locus_15997_Transcript_1/1_Confidence_1.000_Length_1291	FORWARD	55.45	28.57	CCAAAAAGAACACAAAAATCA	171	CTTCCT	6	CTTCCTCTTCCTCTTCCTCTTCCT	24
Locus_15997_Transcript_1/1_Confidence_1.000_Length_1291	REVERSE	55.23	42.86	GAGGAAGAAGAGGATGAAGAA					
Locus_18109_Transcript_1/1_Confidence_1.000_Length_735	FORWARD	54.58	52.38	CTAACCCTCTCCCTCATACTC	151	AAACCC	6	AAACCCAAACCCAAACCCAAACCCAAACC	36
Locus_18109_Transcript_1/1_Confidence_1.000_Length_735	REVERSE	56.33	50	GGCTGATAGGGTTTGTAAGG					
Locus_18110_Transcript_1/1_Confidence_1.000_Length_761	FORWARD	54.58	52.38	CTAACCCTCTCCCTCATACTC	151	AAACCC	6	AAACCCAAACCCAAACCCAAACCCAAACC	36
Locus_18110_Transcript_1/1_Confidence_1.000_Length_761	REVERSE	56.33	50	GGCTGATAGGGTTTGTAAGG					
Locus_18111_Transcript_1/1_Confidence_1.000_Length_746	FORWARD	55.26	52.38	CTCATACCTCCCTCATACTCC	150	AAACCC	6	AAACCCAAACCCAAACCCAAACCCAAACC	36
Locus_18111_Transcript_1/1_Confidence_1.000_Length_746	REVERSE	56.33	50	GGCTGATAGGGTTTGTAAGG					
Locus_18112_Transcript_1/1_Confidence_1.000_Length_721	FORWARD	54.58	52.38	CTAACCCTCTCCCTCATACTC	151	AAACCC	6	AAACCCAAACCCAAACCCAAACCCAAACC	36
Locus_18112_Transcript_1/1_Confidence_1.000_Length_721	REVERSE	56.33	50	GGCTGATAGGGTTTGTAAGG					
Locus_18678_Transcript_1/1_Confidence_1.000_Length_459	FORWARD	55.38	42.86	AGTATTCCTTAGCCATTACGC	154	ACGAAG	6	ACGAAGACGAAGACGAAGACGAAG	24
Locus_18678_Transcript_1/1_Confidence_1.000_Length_459	REVERSE	54.7	42.86	CCACTGGATCAATATGATAGC					
Locus_18679_Transcript_1/1_Confidence_1.000_Length_458	FORWARD	55.38	42.86	AGTATTCCTTAGCCATTACGC	160	ACGAAG	6	ACGAAGACGAAGACGAAGACGAAG	24
Locus_18679_Transcript_1/1_Confidence_1.000_Length_458	REVERSE	54.7	42.86	CCACTGGATCAATATGATAGC					
Locus_18680_Transcript_1/1_Confidence_1.000_Length_452	FORWARD	55.38	42.86	AGTATTCCTTAGCCATTACGC	154	ACGAAG	6	ACGAAGACGAAGACGAAGACGAAG	24
Locus_18680_Transcript_1/1_Confidence_1.000_Length_452	REVERSE	54.7	42.86	CCACTGGATCAATATGATAGC					
Locus_20202_Transcript_1/1_Confidence_1.000_Length_219	FORWARD	54.88	38.1	TTCTTGACTGAGCTTATTTGG	152	AGGAAG	6	AGGAAGAGGAAGAGGAAGAGGAAG	24
Locus_20202_Transcript_1/1_Confidence_1.000_Length_219	REVERSE	55.45	28.57	TTTTTCATGGTTTTCTTTGTG					
Locus_20799_Transcript_1/1_Confidence_1.000_Length_318	FORWARD	54.99	38.1	ATATCACATGCTTGTCTGCTT	140	TTTGCA	6	TTTGCATTTGCATTTGCATTTGCA	24
Locus_20799_Transcript_1/1_Confidence_1.000_Length_318	REVERSE	56.09	42.86	AGACAATGGTGAGGTGATTCT					
Locus_20800_Transcript_1/1_Confidence_1.000_Length_322	FORWARD	55.32	28.57	TTTTGTCTGCTTTTGCATTAT	157	TTTGCA	6	TTTGCATTTGCATTTGCATTTGCA	24
Locus_20800_Transcript_1/1_Confidence_1.000_Length_322	REVERSE	56.09	42.86	AATGGTGAGGTGATTCTCACT					
Locus_20801_Transcript_1/1_Confidence_1.000_Length_324	FORWARD	55.32	28.57	TTTTGTCTGCTTTTGCATTAT	157	TTTGCA	6	TTTGCATTTGCATTTGCATTTGCA	24
Locus_20801_Transcript_1/1_Confidence_1.000_Length_324	REVERSE	56.09	42.86	AATGGTGAGGTGATTCTCACT					
Locus_20802_Transcript_1/1_Confidence_1.000_Length_342	FORWARD	55.32	28.57	TTTTGTCTGCTTTTGCATTAT	165	TTTGCA	6	TTTGCATTTGCATTTGCATTTGCA	24
Locus_20802_Transcript_1/1_Confidence_1.000_Length_342	REVERSE	54.88	47.62	CTAGTGAGAATCACCCAAGTG					
Locus_20803_Transcript_1/1_Confidence_1.000_Length_348	FORWARD	55.32	28.57	TTTTGTCTGCTTTTGCATTAT	165	TTTGCA	6	TTTGCATTTGCATTTGCATTTGCA	24
Locus_20803_Transcript_1/1_Confidence_1.000_Length_348	REVERSE	54.88	47.62	CTAGTGAGAATCACCCAAGTG					
Locus_20804_Transcript_1/1_Confidence_1.000_Length_318	FORWARD	54.69	33.33	TGCATTATATCACATGTTTGC	146	TTTGCA	6	TTTGCATTTGCATTTGCATTTGCA	24
Locus_20804_Transcript_1/1_Confidence_1.000_Length_318	REVERSE	55.18	38.1	TGACAATGGTGATTCTTTCTC					
Locus_20805_Transcript_1/1_Confidence_1.000_Length_224	FORWARD	55.32	28.57	TTTTGTCTGCTTTTGCATTAT	144	TTTGCA	6	TTTGCATTTGCATTTGCATTTGCA	24

Locus_20805_Transcript_1/1_Confidence_1.000_Length_224	REVERSE	55.09	38.1	TGAATGGTGATTCTCATTCTC					
Locus_21213_Transcript_1/1_Confidence_1.000_Length_202	FORWARD	55.45	28.57	TTTTTCATGGTTTTCTTTGTG	123	TCTTCC	6	TCTTCCTCTTCCTCTTCCTCTTCC	24
Locus_21213_Transcript_1/1_Confidence_1.000_Length_202	REVERSE	54.88	38.1	TTCTTGACTGAGCTTATTTGG					
